# Supplementary material for: Impact of the COVID-19 pandemic on incident diagnoses in German refugee centres 2018 to 2023
Source: Nat Commun. 2025 Jul 24;16:6808. doi: 10.1038/s41467-025-61876-x (PMC12290057; doi:10.1038/s41467-025-61876-x)
Supplement: Supplementary file 1 — Supplementary Information [file 41467_2025_61876_MOESM1_ESM.pdf]

Supplementary information

Supplement to: Bozorgmehr K, Erdmann S, Rohleder S, Jahn R. Impact of the COVID-19 pandemic on incident diagnoses in German refugee centres 2018 to 2023.

Content of supplementary information

1. MAIN ANALYSIS ... 1

1.1. DETAILED RESULTS OF THE MAIN ANALYSIS ... 1

1.1.1 Disabilities (short label: Disability) ... 1
1.1.2. Injury, poisoning and certain other consequences of external causes (short label: Cons.ext.causes)... 2
1.1.3. Diseases of the skin and subcutaneous tissue (short label: Skin) ... 3
1.1.4. Diseases of the digestive system (short label: Digestive syst.) ... 4
1.1.5. Diseases of the blood and blood-forming organs and certain disorders involving the immune mechanism (short label: Blood)... 5
1.1.6. Certain infectious and parasitic diseases (short label: Inf.diseases) ... 6
1.1.7. Notifiable infectious diseases (short label: Inf.notify) ... 7
1.1.8. Diseases of the circulatory system (short label: Circulatory syst.) ... 8
1.1.9. Hypertension (short label: Hypertension)... 9
1.1.10. Endocrine, nutritional and metabolic diseases (short label: Metabolic) ... 10
1.1.11. Diabetes mellitus (short label: Diabetes) ... 11
1.1.12. Diseases of the musculoskeletal system and connective tissue (short label: Musculoskelet. syst.).. 12
1.1.13. Neoplasms (short label: Neoplasm) ... 13
1.1.14. Diseases of the nervous system (short label: Nervous syst.) ... 14
1.1.15. Diseases of the ear and mastoid process (short label: Ear.mastoid)... 15
1.1.16. Diseases of the eye and adnexa (short label: Eye.adnexa)... 16
1.1.17. Pregnancy, childbirth and the puerperium (short label: Pregn.condition) ... 17
1.1.18. Mental and behavioral disorders (short label: Psych.condition) ... 18
1.1.19. Psychoactive drug prescriptions (short label: Psych.prescrip.)... 19
1.1.20. Diseases of the respiratory system (short label: Respiratory syst.) ... 20
1.1.21. Diseases of the genitourinary system (short label: Genitourinary syst.) ... 21

1.2. ADDITIONAL RESULTS OF THE COUNTERFACTUAL ANALYSIS ... 22

1.2.1 Figure S1 - Box plot: Disabilities (short label: Disability) ... 22
1.2.2. Figure S2 - Box plot: Injury, poisoning and certain other consequences of external causes (short label: Cons.ext.causes) ... 23
1.2.3. Figure S3 - Box plot: Diseases of the skin and subcutaneous tissue (short label: Skin) ... 24
1.2.4. Figure S4 - Box plot: Diseases of the digestive system (short label: Digestive syst.) ... 25
1.2.5. Figure S5 - Box plot: Diseases of the blood and blood-forming organs and certain disorders involving the immune mechanism (short label: Blood) ... 26
1.2.6. Figure S6 - Box plot: Certain infectious and parasitic diseases (short label: Inf.diseases)... 27
1.2.7. Figure S7 - Box plot: Notifiable infectious diseases (short label: Inf.notify) ... 28
1.2.8. Figure S8 - Box plot: Diseases of the circulatory system (short label: Circulatory syst.)... 29
1.2.9. Figure S9 - Box plot: Hypertension (short label: Hypertension)... 30
1.2.10. Figure S10 - Box plot: Endocrine, nutritional and metabolic diseases (short label: Metabolic) ... 31
1.2.11. Figure S11 - Box plot: Diabetes mellitus (short label: Diabetes)... 32
1.2.12. Figure S12 - Box plot: Diseases of the musculoskeletal system and connective tissue (short label: Musculoskelet. syst.) ... 33
1.2.13. Figure S13 - Box plot: Neoplasms (short label: Neoplasm)... 34
1.2.14. Figure S14 - Box plot: Diseases of the nervous system (short label: Nervous syst.) ... 35
1.2.15. Figure S15 - Box plot: Diseases of the ear and mastoid process (short label: Ear.mastoid)... 36
1.2.16. Figure S16 - Box plot: Diseases of the eye and adnexa (short label: Eye.adnexa)... 37
1.2.17. Figure S17 - Box plot: Pregnancy, childbirth and the puerperium (short label: Pregn.condition) ... 38
1.2.18. Figure S18 - Box plot: Mental and behavioral disorders (short label: Psych.condition) ... 39
1.2.19. Figure S19 - Box plot: Psychoactive drug prescriptions (short label: Psych.prescrip.)... 40
1.2.20. Figure S20 - Box plot: Diseases of the respiratory system (short label: Respiratory syst.) ... 41
1.2.21. Figure S21 - Box plot: Diseases of the genitourinary system (short label: Genitourinary syst.) ... 42

2. SENSITIVITY ANALYSES 1 – 3 ... 43

2.1 DETAILED RESULTS FOR THE SENSITIVITY ANALYSIS 1 ... 43

2.1.1. Disabilities (short label: Disability) ... 43
2.1.2. Injury, poisoning and certain other consequences of external causes (short label: Cons.ext.causes). 44

2.1.3. Diseases of the skin and subcutaneous tissue (short label: Skin) 45
2.1.4. Diseases of the digestive system (short label: Digestive syst.) 46
2.1.5. Certain infectious and parasitic diseases (short label: Inf.diseases) 47
2.1.6. Notifiable infectious diseases (short label: Inf.notify) 48
2.1.7. Diseases of the circulatory system (short label: Circulatory syst.) 49
2.1.8. Hypertension (short label: Hypertension) 50
2.1.9. Endocrine, nutritional and metabolic diseases (short label: Metabolic) 51
2.1.10. Diabetes mellitus (short label: Diabetes) 52
2.1.11. Diseases of the musculoskeletal system and connective tissue (short label: Musculoskelet. syst.) .. 53
2.1.12. Neoplasms (short label: Neoplasm) 54
2.1.13. Diseases of the nervous system (short label: Nervous syst.) 55
2.1.14. Diseases of the ear and mastoid process (short label: Ear.mastoid) 56
2.1.15. Diseases of the eye and adnexa (short label: Eye.adnexa) 57
2.1.16. Pregnancy, childbirth and the puerperium (short label: Pregn.condition) 58
2.1.17. Benzodiazepine prescriptions (short label: Benzo.prescrip) 59
2.1.18. Mental and behavioural disorders (short label: Psych.condition) 60
2.1.19. Psychoactive drug prescriptions (short label: Psych.prescrip.) 61
2.1.20. Diseases of the respiratory system (short label: Respiratory syst.) 62
2.1.21. Diseases of the genitourinary system (short label: Genitourinary syst.) 63
2.2 DETAILED RESULTS FOR THE SENSITIVITY ANALYSIS 2 64
2.2.1. Disabilities (short label: Disability) 64
2.2.2. Injury, poisoning and certain other consequences of external causes (short label: Cons.ext.causes). 65
2.2.3. Diseases of the skin and subcutaneous tissue (short label: Skin) 66
2.2.4. Diseases of the digestive system (short label: Digestive syst.) 67
2.2.5. Diseases of the blood and blood-forming organs and certain disorders involving the immune mechanism (short label: Blood) 68
2.2.6. Certain infectious and parasitic diseases (short label: Inf.diseases) 69
2.2.7. Notifiable infectious diseases (short label: Inf.notify) 70
2.2.8. Diseases of the circulatory system (short label: Circulatory syst.) 71
2.2.9. Endocrine, nutritional and metabolic diseases (short label: Metabolic) 72
2.2.10. Diabetes mellitus (short label: Diabetes) 73
2.2.11. Diseases of the musculoskeletal system and connective tissue (short label: Musculoskelet. syst.) .. 74
2.2.12. Neoplasms (short label: Neoplasm) 75
2.2.13. Diseases of the nervous system (short label: Nervous syst.) 76
2.2.14. Diseases of the ear and mastoid process (short label: Ear.mastoid) 77
2.2.15. Diseases of the eye and adnexa (short label: Eye.adnexa) 78
2.2.16. Pregnancy, childbirth and the puerperium (short label: Pregn.condition) 79
2.2.17. Mental and behavioral disorders (short label: Psych.condition) 80
2.2.18. Psychoactive drug prescriptions (short label: Psych.prescrip.) 81
2.2.19. Diseases of the respiratory system (short label: Respiratory syst.) 82
2.2.20. Diseases of the genitourinary system (short label: Genitourinary syst.) 83
2.2.21. Figure S22: Forestplot of sensitivity analysis 2 on subset 3 showing the effects of the variables Peri-pandemic time trend and Peri-pandemic Incidence (Ref.: Pre-pandemic). 84
2.3. DETAILED RESULTS FOR THE SENSITIVITY ANALYSIS 3 85
2.3.1. Disabilities (short label: Disability) 85
2.3.2. Injury, poisoning and certain other consequences of external causes (short label: Cons.ext.causes). 86
2.3.3. Diseases of the skin and subcutaneous tissue (short label: Skin) 87
2.3.4. Diseases of the digestive system (short label: Digestive syst.) 88
2.3.5. Diseases of the blood and blood-forming organs and certain disorders involving the immune mechanism (short label: Blood) 89
2.3.6. Certain infectious and parasitic diseases (short label: Inf.diseases) 90
2.3.7. Notifiable infectious diseases (short label: Inf.notify) 91
2.3.8. Diseases of the circulatory system (short label: Circulatory syst.) 92
2.3.9. Hypertension (short label: Hypertension) 93
2.3.10. Endocrine, nutritional and metabolic diseases (short label: Metabolic) 94
2.3.11. Diabetes mellitus (short label: Diabetes) 95
2.3.12. Diseases of the musculoskeletal system and connective tissue (short label: Musculoskelet. syst.) .. 96
2.3.13. Neoplasms (short label: Neoplasm) 97

2.3.14. Diseases of the nervous system (short label: Nervous syst.) 98
2.3.15. Diseases of the eye and adnexa (short label: Eye.adnexa).......... 99
2.3.16. Pregnancy, childbirth and the puerperium (short label: Pregn.condition) 100
2.3.17. Mental and behavioural disorders (short label: Psych.condition) 101
2.3.18. Psychoactive drug prescriptions (short label: Psych.prescrip.) 102
2.3.19. Diseases of the respiratory system (short label: Respiratory syst.) 103
2.3.20. Diseases of the genitourinary system (short label: Genitourinary syst.) 104
2.3.21. Figure S23: Forestplot of sensitivity analysis 3 on subset 4 showing the effects of the variables Peri-pandemic time trend and Peri-pandemic Incidence (Ref.: Pre-pandemic). 105

3. AVAILABILITY OF CONTRIBUTED SURVEILLANCE DATA PER CENTRE AND MONTHS 106

3.1 Figure S24: Data availability per centres and months. 106
3.2. Figure S25: Availability of contributed surveillance data per centre and months 107
3.3 Table: Mean age of patients before and after onset of the COVID-19 pandemic, 2018 – 2023 108

4. TOTAL NUMBER OF PERSONS RESIDING IN GERMANY FOR HUMANITARIAN REGIONS, BY STATE AND COUNTRY OF ORIGIN 109

5. SENSITIVITY ANALYSES 4 – 5 110

5.1. Figure S26: Forest Plot of Sensitivity analysis 4, nuanced analysis of peri-pandemic phase 110
5.2. Figure S27: Forest Plot of Sensitivity analysis 5, seasonality effects 111

6. SUPPLEMENTARY TABLES S1 – S3 112

6.1 Supplementary Table S1: Sociodemographic characteristics of patients in refugee centres per month, number of cases of 21 indicators per month as well as incidence proportions of 21 indicators per month, 2018 – 2023, N = 109,175 with 215,864 patient-months. 112
6.2 Supplementary Table S2: Sociodemographic characteristics of patients and occupancy in refugee centres per month, number of cases of 21 indicators per month as well as incidence proportions of 21 indicators per month, 2018 – 2023, N = 102,967 refugees. 113
6.3 Supplementary Table S3: Sociodemographic characteristics of patients and occupancy in refugee centres per month, number of cases of 21 indicators per month as well as incidence proportions of 21 indicators per month, 2018 – 2023, N = 155,546 refugees. 114

1. Main Analysis

1.1. Detailed results of the main analysis

The original R output containing all models, a forest plot and boxplots of observed, expected outcome, and expected counterfactual values of the incidence of all health indicators (Figure S2 to S21) are shown below.

1.1.1 Disabilities (short label: Disability)

```
Family: nbinom2  ( log )
Formula:
m1[, var] ~ `% adults (occup.)` + `% male (occup.)` + `Peri-pandemic time trend` +
    time + `Peri-pandemic Inc.` + (1 | centre) + offset(log(occupancy))
Zero inflation:             ~1
Data: m1

     AIC      BIC   logLik deviance df.resid
  1170.8   1204.6   -576.4   1152.8      305

Random effects:

Conditional model:
 Groups Name        Variance Std.Dev.
 centre (Intercept) 1.578    1.256
Number of obs: 314, groups:  centre, 21

Dispersion parameter for nbinom2 family (): 4.42

Conditional model:
                            Estimate Std. Error z value Pr(>|z|)
(Intercept)                -5.080452   0.907600  -5.598 2.17e-08 ***
`% adults (occup.)`        -0.510148   0.163196  -3.126  0.00177 **
`% male (occup.)`           0.562270   0.140769   3.994 6.49e-05 ***
`Peri-pandemic time trend` -0.017974   0.022900  -0.785  0.43252
time                       -0.006581   0.022383  -0.294  0.76873
`Peri-pandemic Inc.`        0.633184   0.272205   2.326  0.02001 *
---
Signif. codes:  0 '***' 0.001 '**' 0.01 '*' 0.05 '.' 0.1 ' ' 1

Zero-inflation model:
            Estimate Std. Error z value Pr(>|z|)
(Intercept)  -0.5115     0.1903  -2.688  0.00719 **
---
Signif. codes:  0 '***' 0.001 '**' 0.01 '*' 0.05 '.' 0.1 ' ' 1
                                incident rate ratio 2.5 % 97.5 %
cond.(Intercept)                               0.01  0.00   0.04
cond.`% adults (occup.)`                       0.60  0.44   0.83
cond.`% male (occup.)`                         1.75  1.33   2.31
cond.`Peri-pandemic time trend`                0.98  0.94   1.03
cond.time                                      0.99  0.95   1.04
cond.`Peri-pandemic Inc.`                      1.88  1.10   3.21
zi.(Intercept)                                 0.60  0.41   0.87
cond.Std.Dev.(Intercept)|centre                3.51  2.15   7.84
```

1.1.2. Injury, poisoning and certain other consequences of external causes (short label: Cons.ext.causes)

```
Family: nbinom2  ( log )
Formula:
m1[, var] ~ `% adults (occup.)` + `% male (occup.)` + `Peri-pandemic time trend` +
    time + `Peri-pandemic Inc.` + (1 | centre) + offset(log(occupancy))
Zero inflation:             ~1
Data: m1

     AIC      BIC   logLik deviance df.resid
  1785.7   1819.5   -883.9   1767.7      305

Random effects:

Conditional model:
 Groups Name        Variance Std.Dev.
 centre (Intercept) 0.1359   0.3686
Number of obs: 314, groups:  centre, 21

Dispersion parameter for nbinom2 family (): 10.2

Conditional model:
                            Estimate Std. Error z value Pr(>|z|)
(Intercept)                -3.679417   0.422544  -8.708  < 2e-16 ***
`% adults (occup.)`        -0.120798   0.073621  -1.641   0.1008
`% male (occup.)`           0.146760   0.061145   2.400   0.0164 *
`Peri-pandemic time trend`  0.006264   0.013928   0.450   0.6529
time                       -0.021108   0.013660  -1.545   0.1223
`Peri-pandemic Inc.`        0.632944   0.149785   4.226 2.38e-05 ***
---
Signif. codes:  0 '***' 0.001 '**' 0.01 '*' 0.05 '.' 0.1 ' ' 1

Zero-inflation model:
            Estimate Std. Error z value Pr(>|z|)
(Intercept)  -1.3560     0.1538  -8.818   <2e-16 ***
---
Signif. codes:  0 '***' 0.001 '**' 0.01 '*' 0.05 '.' 0.1 ' ' 1
                                incident rate ratio 2.5 % 97.5 %
cond.(Intercept)                               0.03  0.01   0.06
cond.`% adults (occup.)`                       0.89  0.77   1.02
cond.`% male (occup.)`                         1.16  1.03   1.31
cond.`Peri-pandemic time trend`                1.01  0.98   1.03
cond.time                                      0.98  0.95   1.01
cond.`Peri-pandemic Inc.`                      1.88  1.40   2.53
zi.(Intercept)                                 0.26  0.19   0.35
cond.Std.Dev.(Intercept)|centre                1.45  1.29   1.70
```

1.1.3. Diseases of the skin and subcutaneous tissue (short label: Skin)

```
Family: nbinom2  ( log )
Formula:
m1[, var] ~ `% adults (occup.)` + `% male (occup.)` + `Peri-pandemic time trend` +
    time + `Peri-pandemic Inc.` + (1 | centre) + offset(log(occupancy))
Zero inflation:             ~1
Data: m1

     AIC      BIC   logLik deviance df.resid
  1842.1   1875.8   -912.0   1824.1      305

Random effects:

Conditional model:
 Groups Name        Variance Std.Dev.
 centre (Intercept) 0.4238   0.651
Number of obs: 314, groups:  centre, 21

Dispersion parameter for nbinom2 family (): 12.3

Conditional model:
                            Estimate Std. Error z value Pr(>|z|)
(Intercept)                -4.445065   0.410909 -10.818   <2e-16 ***
`% adults (occup.)`        -0.045945   0.066025  -0.696   0.4865
`% male (occup.)`           0.158311   0.057723   2.743   0.0061 **
`Peri-pandemic time trend` -0.012494   0.012000  -1.041   0.2978
time                        0.004734   0.011686   0.405   0.6854
`Peri-pandemic Inc.`        0.164214   0.128224   1.281   0.2003
---
Signif. codes:  0 '***' 0.001 '**' 0.01 '*' 0.05 '.' 0.1 ' ' 1

Zero-inflation model:
            Estimate Std. Error z value Pr(>|z|)
(Intercept)   -1.944      0.203  -9.576   <2e-16 ***
---
Signif. codes:  0 '***' 0.001 '**' 0.01 '*' 0.05 '.' 0.1 ' ' 1
                                incident rate ratio 2.5 % 97.5 %
cond.(Intercept)                               0.01  0.01   0.03
cond.`% adults (occup.)`                       0.96  0.84   1.09
cond.`% male (occup.)`                         1.17  1.05   1.31
cond.`Peri-pandemic time trend`                0.99  0.96   1.01
cond.time                                      1.00  0.98   1.03
cond.`Peri-pandemic Inc.`                      1.18  0.92   1.52
zi.(Intercept)                                 0.14  0.10   0.21
cond.Std.Dev.(Intercept)|centre                1.92  1.55   2.64
```

1.1.4. Diseases of the digestive system (short label: Digestive syst.)

```
Family: nbinom2  ( log )
Formula:
m1[, var] ~ `% adults (occup.)` + `% male (occup.)` + `Peri-pandemic time trend` +
    time + `Peri-pandemic Inc.` + (1 | centre) + offset(log(occupancy))
Zero inflation:             ~1
Data: m1

     AIC      BIC   logLik deviance df.resid
  2114.1   2147.8  -1048.0   2096.1      305

Random effects:

Conditional model:
 Groups Name        Variance Std.Dev.
 centre (Intercept) 0.3329   0.5769
Number of obs: 314, groups:  centre, 21

Dispersion parameter for nbinom2 family (): 13.3

Conditional model:
                            Estimate Std. Error z value Pr(>|z|)
(Intercept)                -2.764646   0.323089  -8.557   <2e-16 ***
`% adults (occup.)`        -0.112432   0.048982  -2.295   0.0217 *
`% male (occup.)`           0.063951   0.045235   1.414   0.1574
`Peri-pandemic time trend`  0.004493   0.009985   0.450   0.6527
time                       -0.004754   0.009676  -0.491   0.6232
`Peri-pandemic Inc.`        0.110849   0.108840   1.018   0.3085
---
Signif. codes:  0 '***' 0.001 '**' 0.01 '*' 0.05 '.' 0.1 ' ' 1

Zero-inflation model:
            Estimate Std. Error z value Pr(>|z|)
(Intercept)  -3.4361     0.4064  -8.456   <2e-16 ***
---
Signif. codes:  0 '***' 0.001 '**' 0.01 '*' 0.05 '.' 0.1 ' ' 1
                                incident rate ratio 2.5 % 97.5 %
cond.(Intercept)                               0.06  0.03   0.12
cond.`% adults (occup.)`                       0.89  0.81   0.98
cond.`% male (occup.)`                         1.07  0.98   1.16
cond.`Peri-pandemic time trend`                1.00  0.99   1.02
cond.time                                      1.00  0.98   1.01
cond.`Peri-pandemic Inc.`                      1.12  0.90   1.38
zi.(Intercept)                                 0.03  0.01   0.07
cond.Std.Dev.(Intercept)|centre                1.78  1.50   2.29
```

1.1.5. Diseases of the blood and blood-forming organs and certain disorders involving the immune mechanism (short label: Blood)

```
Family: nbinom2  ( log )
Formula:
m1[, var] ~ `% adults (occup.)` + `% male (occup.)` + `Peri-pandemic time trend` +
    time + `Peri-pandemic Inc.` + (1 | centre) + offset(log(occupancy))
Zero inflation:             ~1
Data: m1

     AIC      BIC   logLik deviance df.resid
   831.9    865.7   -407.0    813.9      305

Random effects:

Conditional model:
 Groups Name        Variance Std.Dev.
 centre (Intercept) 4.389    2.095
Number of obs: 314, groups:  centre, 21

Dispersion parameter for nbinom2 family (): 12.2

Conditional model:
                           Estimate Std. Error z value Pr(>|z|)
(Intercept)                -4.69630    1.02970  -4.561 5.09e-06 ***
`% adults (occup.)`        -0.33090    0.16803  -1.969   0.0489 *
`% male (occup.)`           0.14129    0.15241   0.927   0.3539
`Peri-pandemic time trend` -0.01067    0.01876  -0.569   0.5696
time                       -0.02070    0.01849  -1.120   0.2627
`Peri-pandemic Inc.`        0.37604    0.20432   1.840   0.0657 .
---
Signif. codes:  0 '***' 0.001 '**' 0.01 '*' 0.05 '.' 0.1 ' ' 1

Zero-inflation model:
            Estimate Std. Error z value Pr(>|z|)
(Intercept)  -0.2919     0.2137  -1.366    0.172
                                incident rate ratio 2.5 % 97.5 %
cond.(Intercept)                               0.01  0.00   0.07
cond.`% adults (occup.)`                       0.72  0.52   1.00
cond.`% male (occup.)`                         1.15  0.85   1.55
cond.`Peri-pandemic time trend`                0.99  0.95   1.03
cond.time                                      0.98  0.94   1.02
cond.`Peri-pandemic Inc.`                      1.46  0.98   2.17
zi.(Intercept)                                 0.75  0.49   1.14
cond.Std.Dev.(Intercept)|centre                8.12  3.31  38.97
```

1.1.6. Certain infectious and parasitic diseases (short label: Inf.diseases)

```
Family: nbinom2  ( log )
Formula:
m1[, var] ~ `% adults (occup.)` + `% male (occup.)` + `Peri-pandemic time trend` +
    time + `Peri-pandemic Inc.` + (1 | centre) + offset(log(occupancy))
Zero inflation:             ~1
Data: m1

     AIC      BIC   logLik deviance df.resid
  2199.2   2232.9  -1090.6   2181.2      305

Random effects:

Conditional model:
 Groups Name        Variance Std.Dev.
 centre (Intercept) 0.4618   0.6795
Number of obs: 314, groups:  centre, 21

Dispersion parameter for nbinom2 family (): 11.1

Conditional model:
                             Estimate Std. Error z value Pr(>|z|)
(Intercept)                -3.5596034  0.3534869 -10.070  < 2e-16 ***
`% adults (occup.)`        -0.0578762  0.0487142  -1.188  0.23480
`% male (occup.)`           0.1240465  0.0466320   2.660  0.00781 **
`Peri-pandemic time trend`  0.0077290  0.0102655   0.753  0.45151
time                        0.0006766  0.0098950   0.068  0.94548
`Peri-pandemic Inc.`       -0.1380722  0.1137031  -1.214  0.22462
---
Signif. codes:  0 '***' 0.001 '**' 0.01 '*' 0.05 '.' 0.1 ' ' 1

Zero-inflation model:
            Estimate Std. Error z value Pr(>|z|)
(Intercept)  -2.7885     0.2817    -9.9   <2e-16 ***
---
Signif. codes:  0 '***' 0.001 '**' 0.01 '*' 0.05 '.' 0.1 ' ' 1
                                incident rate ratio 2.5 % 97.5 %
cond.(Intercept)                               0.03  0.01   0.06
cond.`% adults (occup.)`                       0.94  0.86   1.04
cond.`% male (occup.)`                         1.13  1.03   1.24
cond.`Peri-pandemic time trend`                1.01  0.99   1.03
cond.time                                      1.00  0.98   1.02
cond.`Peri-pandemic Inc.`                      0.87  0.70   1.09
zi.(Intercept)                                 0.06  0.04   0.11
cond.Std.Dev.(Intercept)|centre                1.97  1.60   2.67
```

1.1.7. Notifiable infectious diseases (short label: Inf.notify)

```
Family: nbinom2  ( log )
Formula:
m1[, var] ~ `% adults (occup.)` + `% male (occup.)` + `Peri-pandemic time trend` +
    time + `Peri-pandemic Inc.` + (1 | centre) + offset(log(occupancy))
Zero inflation:             ~1
Data: m1

     AIC      BIC   logLik deviance df.resid
   966.6   1000.4   -474.3    948.6      305

Random effects:

Conditional model:
 Groups Name        Variance Std.Dev.
 centre (Intercept) 1.273    1.128
Number of obs: 314, groups:  centre, 21

Dispersion parameter for nbinom2 family (): 16.2

Conditional model:
                           Estimate Std. Error z value Pr(>|z|)
(Intercept)                -3.23501    0.81761  -3.957  7.6e-05 ***
`% adults (occup.)`        -0.27835    0.15236  -1.827   0.0677 .
`% male (occup.)`           0.14563    0.13040   1.117   0.2641
`Peri-pandemic time trend`  0.02459    0.01665   1.477   0.1397
time                       -0.04002    0.01663  -2.407   0.0161 *
`Peri-pandemic Inc.`        0.22357    0.20718   1.079   0.2805
---
Signif. codes:  0 '***' 0.001 '**' 0.01 '*' 0.05 '.' 0.1 ' ' 1

Zero-inflation model:
            Estimate Std. Error z value Pr(>|z|)
(Intercept)   0.1383     0.1647    0.84    0.401
                                incident rate ratio 2.5 % 97.5 %
cond.(Intercept)                               0.04  0.01   0.20
cond.`% adults (occup.)`                       0.76  0.56   1.02
cond.`% male (occup.)`                         1.16  0.90   1.49
cond.`Peri-pandemic time trend`                1.02  0.99   1.06
cond.time                                      0.96  0.93   0.99
cond.`Peri-pandemic Inc.`                      1.25  0.83   1.88
zi.(Intercept)                                 1.15  0.83   1.59
cond.Std.Dev.(Intercept)|centre                3.09  1.95   6.69
```

1.1.8. Diseases of the circulatory system (short label: Circulatory syst.)

```
Family: nbinom2  ( log )
Formula:
m1[, var] ~ `% adults (occup.)` + `% male (occup.)` + `Peri-pandemic time trend` +
    time + `Peri-pandemic Inc.` + (1 | centre) + offset(log(occupancy))
Zero inflation:             ~1
Data: m1

     AIC      BIC   logLik deviance df.resid
  1688.1   1721.8   -835.0   1670.1      305

Random effects:

Conditional model:
 Groups Name        Variance Std.Dev.
 centre (Intercept) 0.1796   0.4237
Number of obs: 314, groups:  centre, 21

Dispersion parameter for nbinom2 family (): 11.8

Conditional model:
                           Estimate Std. Error z value Pr(>|z|)
(Intercept)                -3.83919    0.42061  -9.128  < 2e-16 ***
`% adults (occup.)`         0.15688    0.06992   2.244  0.02484 *
`% male (occup.)`          -0.20273    0.06166  -3.288  0.00101 **
`Peri-pandemic time trend`  0.01241    0.01369   0.907  0.36457
time                       -0.01104    0.01329  -0.831  0.40620
`Peri-pandemic Inc.`        0.23329    0.14513   1.607  0.10795
---
Signif. codes:  0 '***' 0.001 '**' 0.01 '*' 0.05 '.' 0.1 ' ' 1

Zero-inflation model:
            Estimate Std. Error z value Pr(>|z|)
(Intercept)  -1.4094     0.1789  -7.876 3.37e-15 ***
---
Signif. codes:  0 '***' 0.001 '**' 0.01 '*' 0.05 '.' 0.1 ' ' 1
                                incident rate ratio 2.5 % 97.5 %
cond.(Intercept)                               0.02  0.01   0.05
cond.`% adults (occup.)`                       1.17  1.02   1.34
cond.`% male (occup.)`                         0.82  0.72   0.92
cond.`Peri-pandemic time trend`                1.01  0.99   1.04
cond.time                                      0.99  0.96   1.02
cond.`Peri-pandemic Inc.`                      1.26  0.95   1.68
zi.(Intercept)                                 0.24  0.17   0.35
cond.Std.Dev.(Intercept)|centre                1.53  1.32   1.89
```

1.1.9. Hypertension (short label: Hypertension)

```
Family: nbinom2  ( log )
Formula:
m1[, var] ~ `% adults (occup.)` + `% male (occup.)` + `Peri-pandemic time trend` +
    time + `Peri-pandemic Inc.` + (1 | centre) + offset(log(occupancy))
Zero inflation:             ~1
Data: m1

     AIC      BIC   logLik deviance df.resid
  1317.4   1351.1   -649.7   1299.4      305

Random effects:

Conditional model:
 Groups Name        Variance Std.Dev.
 centre (Intercept) 0.6677   0.8171
Number of obs: 314, groups:  centre, 21

Dispersion parameter for nbinom2 family (): 18.6

Conditional model:
                           Estimate Std. Error z value Pr(>|z|)
(Intercept)                -4.53933    0.54051  -8.398   <2e-16 ***
`% adults (occup.)`         0.12319    0.09687   1.272   0.2035
`% male (occup.)`          -0.12535    0.08223  -1.524   0.1274
`Peri-pandemic time trend`  0.03014    0.01402   2.150   0.0316 *
time                       -0.01888    0.01370  -1.378   0.1683
`Peri-pandemic Inc.`        0.23900    0.16155   1.479   0.1390
---
Signif. codes:  0 '***' 0.001 '**' 0.01 '*' 0.05 '.' 0.1 ' ' 1

Zero-inflation model:
            Estimate Std. Error z value Pr(>|z|)
(Intercept)  -0.2888     0.1385  -2.086    0.037 *
---
Signif. codes:  0 '***' 0.001 '**' 0.01 '*' 0.05 '.' 0.1 ' ' 1
                                incident rate ratio 2.5 % 97.5 %
cond.(Intercept)                               0.01  0.00   0.03
cond.`% adults (occup.)`                       1.13  0.94   1.37
cond.`% male (occup.)`                         0.88  0.75   1.04
cond.`Peri-pandemic time trend`                1.03  1.00   1.06
cond.time                                      0.98  0.96   1.01
cond.`Peri-pandemic Inc.`                      1.27  0.93   1.74
zi.(Intercept)                                 0.75  0.57   0.98
cond.Std.Dev.(Intercept)|centre                2.26  1.64   3.87
```

1.1.10. Endocrine, nutritional and metabolic diseases (short label: Metabolic)

```
Family: nbinom2  ( log )
Formula:
m1[, var] ~ `% adults (occup.)` + `% male (occup.)` + `Peri-pandemic time trend` +
    time + `Peri-pandemic Inc.` + (1 | centre) + offset(log(occupancy))
Zero inflation:             ~1
Data: m1

     AIC      BIC   logLik deviance df.resid
  1593.1   1626.9   -787.6   1575.1      305

Random effects:

Conditional model:
 Groups Name        Variance Std.Dev.
 centre (Intercept) 0.395    0.6285
Number of obs: 314, groups:  centre, 21

Dispersion parameter for nbinom2 family (): 12.1

Conditional model:
                             Estimate Std. Error z value Pr(>|z|)
(Intercept)                -3.7422201  0.4743201  -7.890 3.03e-15 ***
`% adults (occup.)`         0.0797585  0.0807184   0.988  0.32310
`% male (occup.)`          -0.1892092  0.0737886  -2.564  0.01034 *
`Peri-pandemic time trend` -0.0076682  0.0137915  -0.556  0.57820
time                        0.0009939  0.0134646   0.074  0.94116
`Peri-pandemic Inc.`        0.4521714  0.1546416   2.924  0.00346 **
---
Signif. codes:  0 '***' 0.001 '**' 0.01 '*' 0.05 '.' 0.1 ' ' 1

Zero-inflation model:
            Estimate Std. Error z value Pr(>|z|)
(Intercept)  -1.1021     0.1561  -7.061 1.65e-12 ***
---
Signif. codes:  0 '***' 0.001 '**' 0.01 '*' 0.05 '.' 0.1 ' ' 1
                                incident rate ratio 2.5 % 97.5 %
cond.(Intercept)                               0.02  0.01   0.06
cond.`% adults (occup.)`                       1.08  0.92   1.27
cond.`% male (occup.)`                         0.83  0.72   0.96
cond.`Peri-pandemic time trend`                0.99  0.97   1.02
cond.time                                      1.00  0.97   1.03
cond.`Peri-pandemic Inc.`                      1.57  1.16   2.13
zi.(Intercept)                                 0.33  0.24   0.45
cond.Std.Dev.(Intercept)|centre                1.87  1.47   2.80
```

1.1.11. Diabetes mellitus (short label: Diabetes)

```
Family: nbinom2  ( log )
Formula:
m1[, var] ~ `% adults (occup.)` + `% male (occup.)` + `Peri-pandemic time trend` +
    time + `Peri-pandemic Inc.` + (1 | centre) + offset(log(occupancy))
Zero inflation:             ~1
Data: m1

     AIC      BIC   logLik deviance df.resid
  1098.4   1132.2   -540.2   1080.4      305

Random effects:

Conditional model:
 Groups Name        Variance Std.Dev.
 centre (Intercept) 1.743    1.32
Number of obs: 314, groups:  centre, 21

Dispersion parameter for nbinom2 family (): 11.4

Conditional model:
                           Estimate Std. Error z value Pr(>|z|)
(Intercept)                -5.37149    0.73940  -7.265 3.74e-13 ***
`% adults (occup.)`         0.26902    0.12618   2.132   0.0330 *
`% male (occup.)`          -0.33573    0.11106  -3.023   0.0025 **
`Peri-pandemic time trend`  0.03292    0.01795   1.834   0.0666 .
time                       -0.02344    0.01753  -1.337   0.1812
`Peri-pandemic Inc.`        0.36686    0.19898   1.844   0.0652 .
---
Signif. codes:  0 '***' 0.001 '**' 0.01 '*' 0.05 '.' 0.1 ' ' 1

Zero-inflation model:
            Estimate Std. Error z value Pr(>|z|)
(Intercept)  -0.7568     0.2138   -3.54    4e-04 ***
---
Signif. codes:  0 '***' 0.001 '**' 0.01 '*' 0.05 '.' 0.1 ' ' 1
                                incident rate ratio 2.5 % 97.5 %
cond.(Intercept)                               0.00  0.00   0.02
cond.`% adults (occup.)`                       1.31  1.02   1.68
cond.`% male (occup.)`                         0.71  0.57   0.89
cond.`Peri-pandemic time trend`                1.03  1.00   1.07
cond.time                                      0.98  0.94   1.01
cond.`Peri-pandemic Inc.`                      1.44  0.98   2.13
zi.(Intercept)                                 0.47  0.31   0.71
cond.Std.Dev.(Intercept)|centre                3.74  2.26   8.52
```

1.1.12. Diseases of the musculoskeletal system and connective tissue (short label: Musculoskelet. syst.)

```
Family: nbinom2  ( log )
Formula:
m1[, var] ~ `% adults (occup.)` + `% male (occup.)` + `Peri-pandemic time trend` +
    time + `Peri-pandemic Inc.` + (1 | centre) + offset(log(occupancy))
Zero inflation:             ~1
Data: m1

     AIC      BIC   logLik deviance df.resid
  2011.8   2045.6   -996.9   1993.8      305

Random effects:

Conditional model:
 Groups Name        Variance Std.Dev.
 centre (Intercept) 0.2745   0.5239
Number of obs: 314, groups:  centre, 21

Dispersion parameter for nbinom2 family (): 11.5

Conditional model:
                            Estimate Std. Error z value Pr(>|z|)
(Intercept)                -3.331808   0.358723  -9.288  < 2e-16 ***
`% adults (occup.)`        -0.074037   0.057225  -1.294  0.19575
`% male (occup.)`           0.093682   0.051807   1.808  0.07056 .
`Peri-pandemic time trend` -0.001399   0.010890  -0.128  0.89781
time                       -0.013360   0.010530  -1.269  0.20453
`Peri-pandemic Inc.`        0.307821   0.118424   2.599  0.00934 **
---
Signif. codes:  0 '***' 0.001 '**' 0.01 '*' 0.05 '.' 0.1 ' ' 1

Zero-inflation model:
            Estimate Std. Error z value Pr(>|z|)
(Intercept)  -2.5743     0.2889   -8.91   <2e-16 ***
---
Signif. codes:  0 '***' 0.001 '**' 0.01 '*' 0.05 '.' 0.1 ' ' 1
                                incident rate ratio 2.5 % 97.5 %
cond.(Intercept)                               0.04  0.02   0.07
cond.`% adults (occup.)`                       0.93  0.83   1.04
cond.`% male (occup.)`                         1.10  0.99   1.22
cond.`Peri-pandemic time trend`                1.00  0.98   1.02
cond.time                                      0.99  0.97   1.01
cond.`Peri-pandemic Inc.`                      1.36  1.08   1.72
zi.(Intercept)                                 0.08  0.04   0.13
cond.Std.Dev.(Intercept)|centre                1.69  1.41   2.21
```

1.1.13. Neoplasms (short label: Neoplasm)

```
Family: nbinom2  ( log )
Formula:
m1[, var] ~ `% adults (occup.)` + `% male (occup.)` + `Peri-pandemic time trend` +
    time + `Peri-pandemic Inc.` + (1 | centre) + offset(log(occupancy))
Zero inflation:             ~1
Data: m1

     AIC      BIC   logLik deviance df.resid
   756.4    790.1   -369.2    738.4      305

Random effects:

Conditional model:
 Groups Name        Variance Std.Dev.
 centre (Intercept) 11.17    3.342
Number of obs: 314, groups:  centre, 21

Dispersion parameter for nbinom2 family (): 22.9

Conditional model:
                             Estimate Std. Error z value Pr(>|z|)
(Intercept)                -8.7690185  1.6300346  -5.380 7.46e-08 ***
`% adults (occup.)`         0.3055067  0.1298687   2.352  0.01865 *
`% male (occup.)`          -0.3309042  0.1258543  -2.629  0.00856 **
`Peri-pandemic time trend` -0.0071942  0.0168281  -0.428  0.66901
time                        0.0001899  0.0164764   0.012  0.99080
`Peri-pandemic Inc.`        0.4331863  0.1921056   2.255  0.02414 *
---
Signif. codes:  0 '***' 0.001 '**' 0.01 '*' 0.05 '.' 0.1 ' ' 1

Zero-inflation model:
            Estimate Std. Error z value Pr(>|z|)
(Intercept)  -0.2814     0.1917  -1.468    0.142
                                incident rate ratio 2.5 %  97.5 %
cond.(Intercept)                              0.00  0.00    0.00
cond.`% adults (occup.)`                      1.36  1.05    1.75
cond.`% male (occup.)`                        0.72  0.56    0.92
cond.`Peri-pandemic time trend`               0.99  0.96    1.03
cond.time                                     1.00  0.97    1.03
cond.`Peri-pandemic Inc.`                     1.54  1.06    2.25
zi.(Intercept)                                0.75  0.52    1.10
cond.Std.Dev.(Intercept)|centre              28.27  4.90 1129.34
```

1.1.14. Diseases of the nervous system (short label: Nervous syst.)

```
Family: nbinom2  ( log )
Formula:
m1[, var] ~ `% adults (occup.)` + `% male (occup.)` + `Peri-pandemic time trend` +
    time + `Peri-pandemic Inc.` + (1 | centre) + offset(log(occupancy))
Zero inflation:             ~1
Data: m1

     AIC      BIC   logLik deviance df.resid
  1584.9   1618.6   -783.4   1566.9      305

Random effects:

Conditional model:
 Groups Name        Variance Std.Dev.
 centre (Intercept) 0.6525   0.8078
Number of obs: 314, groups:  centre, 21

Dispersion parameter for nbinom2 family (): 8.04

Conditional model:
                            Estimate Std. Error z value Pr(>|z|)
(Intercept)                -3.909887   0.564248  -6.929 4.23e-12 ***
`% adults (occup.)`         0.043241   0.090252   0.479    0.632
`% male (occup.)`          -0.100882   0.085168  -1.185    0.236
`Peri-pandemic time trend` -0.004481   0.015653  -0.286    0.775
time                       -0.005518   0.015171  -0.364    0.716
`Peri-pandemic Inc.`        0.278864   0.169735   1.643    0.100
---
Signif. codes:  0 '***' 0.001 '**' 0.01 '*' 0.05 '.' 0.1 ' ' 1

Zero-inflation model:
            Estimate Std. Error z value Pr(>|z|)
(Intercept)  -1.3743     0.1851  -7.423 1.14e-13 ***
---
Signif. codes:  0 '***' 0.001 '**' 0.01 '*' 0.05 '.' 0.1 ' ' 1
                                incident rate ratio 2.5 % 97.5 %
cond.(Intercept)                               0.02  0.01   0.06
cond.`% adults (occup.)`                       1.04  0.87   1.25
cond.`% male (occup.)`                         0.90  0.77   1.07
cond.`Peri-pandemic time trend`                1.00  0.97   1.03
cond.time                                      0.99  0.97   1.02
cond.`Peri-pandemic Inc.`                      1.32  0.95   1.84
zi.(Intercept)                                 0.25  0.18   0.36
cond.Std.Dev.(Intercept)|centre                2.24  1.67   3.56
```

1.1.15. Diseases of the ear and mastoid process (short label: Ear.mastoid)

```
Family: nbinom2  ( log )
Formula:
m1[, var] ~ `% adults (occup.)` + `% male (occup.)` + `Peri-pandemic time trend` +
    time + `Peri-pandemic Inc.` + (1 | centre) + offset(log(occupancy))
Zero inflation:             ~1
Data: m1

     AIC      BIC   logLik deviance df.resid
  1118.0   1151.7   -550.0   1100.0      305

Random effects:

Conditional model:
 Groups Name        Variance  Std.Dev.
 centre (Intercept) 5.235e-10 2.288e-05
Number of obs: 314, groups:  centre, 21

Dispersion parameter for nbinom2 family (): 7.98

Conditional model:
                            Estimate Std. Error z value Pr(>|z|)
(Intercept)                -3.370449   0.552753  -6.098 1.08e-09 ***
`% adults (occup.)`        -0.186731   0.121206  -1.541  0.12341
`% male (occup.)`           0.045126   0.104642   0.431  0.66629
`Peri-pandemic time trend` -0.007714   0.018076  -0.427  0.66956
time                       -0.008242   0.017418  -0.473  0.63610
`Peri-pandemic Inc.`        0.530520   0.201922   2.627  0.00861 **
---
Signif. codes:  0 '***' 0.001 '**' 0.01 '*' 0.05 '.' 0.1 ' ' 1

Zero-inflation model:
             Estimate Std. Error z value Pr(>|z|)
(Intercept) -0.005658   0.131832  -0.043    0.966
                                incident rate ratio 2.5 % 97.5 %
cond.(Intercept)                              0.03  0.01   0.10
cond.`% adults (occup.)`                      0.83  0.65   1.05
cond.`% male (occup.)`                        1.05  0.85   1.28
cond.`Peri-pandemic time trend`               0.99  0.96   1.03
cond.time                                     0.99  0.96   1.03
cond.`Peri-pandemic Inc.`                     1.70  1.14   2.53
zi.(Intercept)                                0.99  0.77   1.29
cond.Std.Dev.(Intercept)|centre               1.00  1.00    Inf
```

1.1.16. Diseases of the eye and adnexa (short label: Eye.adnexa)

```
Family: nbinom2  ( log )
Formula:
m1[, var] ~ `% adults (occup.)` + `% male (occup.)` + `Peri-pandemic time trend` +
    time + `Peri-pandemic Inc.` + (1 | centre) + offset(log(occupancy))
Zero inflation:             ~1
Data: m1

     AIC      BIC   logLik deviance df.resid
  1314.5   1348.2   -648.2   1296.5      305

Random effects:

Conditional model:
 Groups Name        Variance Std.Dev.
 centre (Intercept) 0.279    0.5282
Number of obs: 314, groups:  centre, 21

Dispersion parameter for nbinom2 family (): 9.32

Conditional model:
                            Estimate Std. Error z value Pr(>|z|)
(Intercept)                -3.938758   0.597472  -6.592 4.33e-11 ***
`% adults (occup.)`        -0.048666   0.116366  -0.418    0.676
`% male (occup.)`           0.001256   0.099254   0.013    0.990
`Peri-pandemic time trend` -0.004267   0.017416  -0.245    0.806
time                       -0.010804   0.017083  -0.632    0.527
`Peri-pandemic Inc.`        0.296389   0.186959   1.585    0.113
---
Signif. codes:  0 '***' 0.001 '**' 0.01 '*' 0.05 '.' 0.1 ' ' 1

Zero-inflation model:
            Estimate Std. Error z value Pr(>|z|)
(Intercept)  -0.3919     0.1642  -2.387    0.017 *
---
Signif. codes:  0 '***' 0.001 '**' 0.01 '*' 0.05 '.' 0.1 ' ' 1
                                incident rate ratio 2.5 % 97.5 %
cond.(Intercept)                               0.02  0.01   0.06
cond.`% adults (occup.)`                       0.95  0.76   1.20
cond.`% male (occup.)`                         1.00  0.82   1.22
cond.`Peri-pandemic time trend`                1.00  0.96   1.03
cond.time                                      0.99  0.96   1.02
cond.`Peri-pandemic Inc.`                      1.34  0.93   1.94
zi.(Intercept)                                 0.68  0.49   0.93
cond.Std.Dev.(Intercept)|centre                1.70  1.28   3.07
```

1.1.17. Pregnancy, childbirth and the puerperium (short label: Pregn.condition)

```
Family: nbinom2  ( log )
Formula:
m1[, var] ~ `% adults (occup.)` + `% male (occup.)` + `Peri-pandemic time trend` +
    time + `Peri-pandemic Inc.` + (1 | centre) + offset(log(occupancy))
Zero inflation:             ~1
Data: m1

     AIC      BIC   logLik deviance df.resid
  1481.7   1515.4   -731.8   1463.7      305

Random effects:

Conditional model:
 Groups Name        Variance Std.Dev.
 centre (Intercept) 5.14     2.267
Number of obs: 314, groups:  centre, 21

Dispersion parameter for nbinom2 family (): 11.5

Conditional model:
                            Estimate Std. Error z value Pr(>|z|)
(Intercept)                -4.360910   0.722090  -6.039 1.55e-09 ***
`% adults (occup.)`         0.060918   0.066143   0.921   0.3570
`% male (occup.)`          -0.160179   0.073316  -2.185   0.0289 *
`Peri-pandemic time trend`  0.024855   0.014929   1.665   0.0959 .
time                       -0.029815   0.014558  -2.048   0.0406 *
`Peri-pandemic Inc.`       -0.001921   0.151705  -0.013   0.9899
---
Signif. codes:  0 '***' 0.001 '**' 0.01 '*' 0.05 '.' 0.1 ' ' 1

Zero-inflation model:
            Estimate Std. Error z value Pr(>|z|)
(Intercept)  -2.2833     0.3173  -7.197 6.16e-13 ***
---
Signif. codes:  0 '***' 0.001 '**' 0.01 '*' 0.05 '.' 0.1 ' ' 1
                                incident rate ratio 2.5 % 97.5 %
cond.(Intercept)                               0.01  0.00   0.05
cond.`% adults (occup.)`                       1.06  0.93   1.21
cond.`% male (occup.)`                         0.85  0.74   0.98
cond.`Peri-pandemic time trend`                1.03  1.00   1.06
cond.time                                      0.97  0.94   1.00
cond.`Peri-pandemic Inc.`                      1.00  0.74   1.34
zi.(Intercept)                                 0.10  0.05   0.19
cond.Std.Dev.(Intercept)|centre                9.65  4.44  31.37
```

1.1.18. Mental and behavioral disorders (short label: Psych.condition)

```
Family: nbinom2  ( log )
Formula:
m1[, var] ~ `% adults (occup.)` + `% male (occup.)` + `Peri-pandemic time trend` +
    time + `Peri-pandemic Inc.` + (1 | centre) + offset(log(occupancy))
Zero inflation:             ~1
Data: m1

     AIC      BIC   logLik deviance df.resid
  2198.7   2232.4  -1090.3   2180.7      305

Random effects:

Conditional model:
 Groups Name        Variance Std.Dev.
 centre (Intercept) 0.2134   0.462
Number of obs: 314, groups:  centre, 21

Dispersion parameter for nbinom2 family (): 5.25

Conditional model:
                            Estimate Std. Error z value Pr(>|z|)
(Intercept)                -3.809383   0.455850  -8.357  < 2e-16 ***
`% adults (occup.)`         0.030713   0.070384   0.436 0.662573
`% male (occup.)`           0.038564   0.060646   0.636 0.524851
`Peri-pandemic time trend` -0.002087   0.014594  -0.143 0.886305
time                       -0.009526   0.013996  -0.681 0.496116
`Peri-pandemic Inc.`        0.545234   0.157961   3.452 0.000557 ***
---
Signif. codes:  0 '***' 0.001 '**' 0.01 '*' 0.05 '.' 0.1 ' ' 1

Zero-inflation model:
            Estimate Std. Error z value Pr(>|z|)
(Intercept)  -2.7049     0.2825  -9.576   <2e-16 ***
---
Signif. codes:  0 '***' 0.001 '**' 0.01 '*' 0.05 '.' 0.1 ' ' 1
                                incident rate ratio 2.5 % 97.5 %
cond.(Intercept)                               0.02  0.01   0.05
cond.`% adults (occup.)`                       1.03  0.90   1.18
cond.`% male (occup.)`                         1.04  0.92   1.17
cond.`Peri-pandemic time trend`                1.00  0.97   1.03
cond.time                                      0.99  0.96   1.02
cond.`Peri-pandemic Inc.`                      1.73  1.27   2.35
zi.(Intercept)                                 0.07  0.04   0.12
cond.Std.Dev.(Intercept)|centre                1.59  1.37   1.97
```

1.1.19. Psychoactive drug prescriptions (short label: Psych.prescrip.)

```
Family: nbinom2  ( log )
Formula:
m1[, var] ~ `% adults (occup.)` + `% male (occup.)` + `Peri-pandemic time trend` +
    time + `Peri-pandemic Inc.` + (1 | centre) + offset(log(occupancy))
Zero inflation:             ~1
Data: m1

     AIC      BIC   logLik deviance df.resid
  1235.6   1269.3   -608.8   1217.6      305

Random effects:

Conditional model:
 Groups Name        Variance Std.Dev.
 centre (Intercept) 0.9663   0.983
Number of obs: 314, groups:  centre, 21

Dispersion parameter for nbinom2 family (): 5.51

Conditional model:
                            Estimate Std. Error z value Pr(>|z|)
(Intercept)                -5.001227   0.808504  -6.186 6.18e-10 ***
`% adults (occup.)`         0.266237   0.104808   2.540   0.0111 *
`% male (occup.)`          -0.264153   0.103525  -2.552   0.0107 *
`Peri-pandemic time trend` -0.015950   0.024209  -0.659   0.5100
time                       -0.005799   0.023270  -0.249   0.8032
`Peri-pandemic Inc.`        0.666472   0.227569   2.929   0.0034 **
---
Signif. codes:  0 '***' 0.001 '**' 0.01 '*' 0.05 '.' 0.1 ' ' 1

Zero-inflation model:
            Estimate Std. Error z value Pr(>|z|)
(Intercept)  -0.5153     0.1690  -3.048   0.0023 **
---
Signif. codes:  0 '***' 0.001 '**' 0.01 '*' 0.05 '.' 0.1 ' ' 1
                                incident rate ratio 2.5 % 97.5 %
cond.(Intercept)                               0.01  0.00   0.03
cond.`% adults (occup.)`                       1.31  1.06   1.60
cond.`% male (occup.)`                         0.77  0.63   0.94
cond.`Peri-pandemic time trend`                0.98  0.94   1.03
cond.time                                      0.99  0.95   1.04
cond.`Peri-pandemic Inc.`                      1.95  1.25   3.04
zi.(Intercept)                                 0.60  0.43   0.83
cond.Std.Dev.(Intercept)|centre                2.67  1.84   4.90
```

1.1.20. Diseases of the respiratory system (short label: Respiratory syst.)

```
Family: nbinom2  ( log )
Formula:
m1[, var] ~ `% adults (occup.)` + `% male (occup.)` + `Peri-pandemic time trend` +
    time + `Peri-pandemic Inc.` + (1 | centre) + offset(log(occupancy))
Zero inflation:             ~1
Data: m1

     AIC      BIC   logLik deviance df.resid
  2405.1   2438.9  -1193.6   2387.1      305

Random effects:

Conditional model:
 Groups Name        Variance Std.Dev.
 centre (Intercept) 0.1379   0.3714
Number of obs: 314, groups:  centre, 21

Dispersion parameter for nbinom2 family (): 5.12

Conditional model:
                           Estimate Std. Error z value Pr(>|z|)
(Intercept)                -2.39658    0.41551  -5.768 8.03e-09 ***
`% adults (occup.)`         0.04299    0.06341   0.678   0.4978
`% male (occup.)`          -0.04109    0.05760  -0.713   0.4756
`Peri-pandemic time trend`  0.03851    0.01306   2.948   0.0032 **
time                       -0.02138    0.01238  -1.727   0.0842 .
`Peri-pandemic Inc.`       -0.68075    0.14184  -4.799 1.59e-06 ***
---
Signif. codes:  0 '***' 0.001 '**' 0.01 '*' 0.05 '.' 0.1 ' ' 1

Zero-inflation model:
            Estimate Std. Error z value Pr(>|z|)
(Intercept)  -2.3875     0.2269  -10.52   <2e-16 ***
---
Signif. codes:  0 '***' 0.001 '**' 0.01 '*' 0.05 '.' 0.1 ' ' 1
                                incident rate ratio 2.5 % 97.5 %
cond.(Intercept)                               0.09  0.04   0.21
cond.`% adults (occup.)`                       1.04  0.92   1.18
cond.`% male (occup.)`                         0.96  0.86   1.07
cond.`Peri-pandemic time trend`                1.04  1.01   1.07
cond.time                                      0.98  0.96   1.00
cond.`Peri-pandemic Inc.`                      0.51  0.38   0.67
zi.(Intercept)                                 0.09  0.06   0.14
cond.Std.Dev.(Intercept)|centre                1.45  1.26   1.81
```

1.1.21. Diseases of the genitourinary system (short label: Genitourinary syst.)

```
Family: nbinom2  ( log )
Formula:
m1[, var] ~ `% adults (occup.)` + `% male (occup.)` + `Peri-pandemic time trend` +
    time + `Peri-pandemic Inc.` + (1 | centre) + offset(log(occupancy))
Zero inflation:             ~1
Data: m1

     AIC      BIC   logLik deviance df.resid
  1619.7   1653.5   -800.9   1601.7      305

Random effects:

Conditional model:
 Groups Name        Variance Std.Dev.
 centre (Intercept) 0.5315   0.7291
Number of obs: 314, groups:  centre, 21

Dispersion parameter for nbinom2 family (): 14.9

Conditional model:
                           Estimate Std. Error z value Pr(>|z|)
(Intercept)                -3.26847    0.43173  -7.571 3.72e-14 ***
`% adults (occup.)`         0.05271    0.05973   0.882  0.37757
`% male (occup.)`          -0.18561    0.06078  -3.054  0.00226 **
`Peri-pandemic time trend`  0.01087    0.01302   0.835  0.40378
time                       -0.01901    0.01265  -1.503  0.13293
`Peri-pandemic Inc.`        0.41512    0.13558   3.062  0.00220 **
---
Signif. codes:  0 '***' 0.001 '**' 0.01 '*' 0.05 '.' 0.1 ' ' 1

Zero-inflation model:
            Estimate Std. Error z value Pr(>|z|)
(Intercept)  -1.4543     0.1883  -7.724 1.13e-14 ***
---
Signif. codes:  0 '***' 0.001 '**' 0.01 '*' 0.05 '.' 0.1 ' ' 1
                                incident rate ratio 2.5 % 97.5 %
cond.(Intercept)                               0.04  0.02   0.09
cond.`% adults (occup.)`                       1.05  0.94   1.19
cond.`% male (occup.)`                         0.83  0.74   0.94
cond.`Peri-pandemic time trend`                1.01  0.99   1.04
cond.time                                      0.98  0.96   1.01
cond.`Peri-pandemic Inc.`                      1.51  1.16   1.98
zi.(Intercept)                                 0.23  0.16   0.34
cond.Std.Dev.(Intercept)|centre                2.07  1.59   3.14
```

1.2. Additional results of the counterfactual analysis

Box plots of observed and expected outcome values as well as expected counterfactual values of the incidence of all health indicators.

1.2.1 Figure S1 - Box plot: Disabilities (short label: Disability)

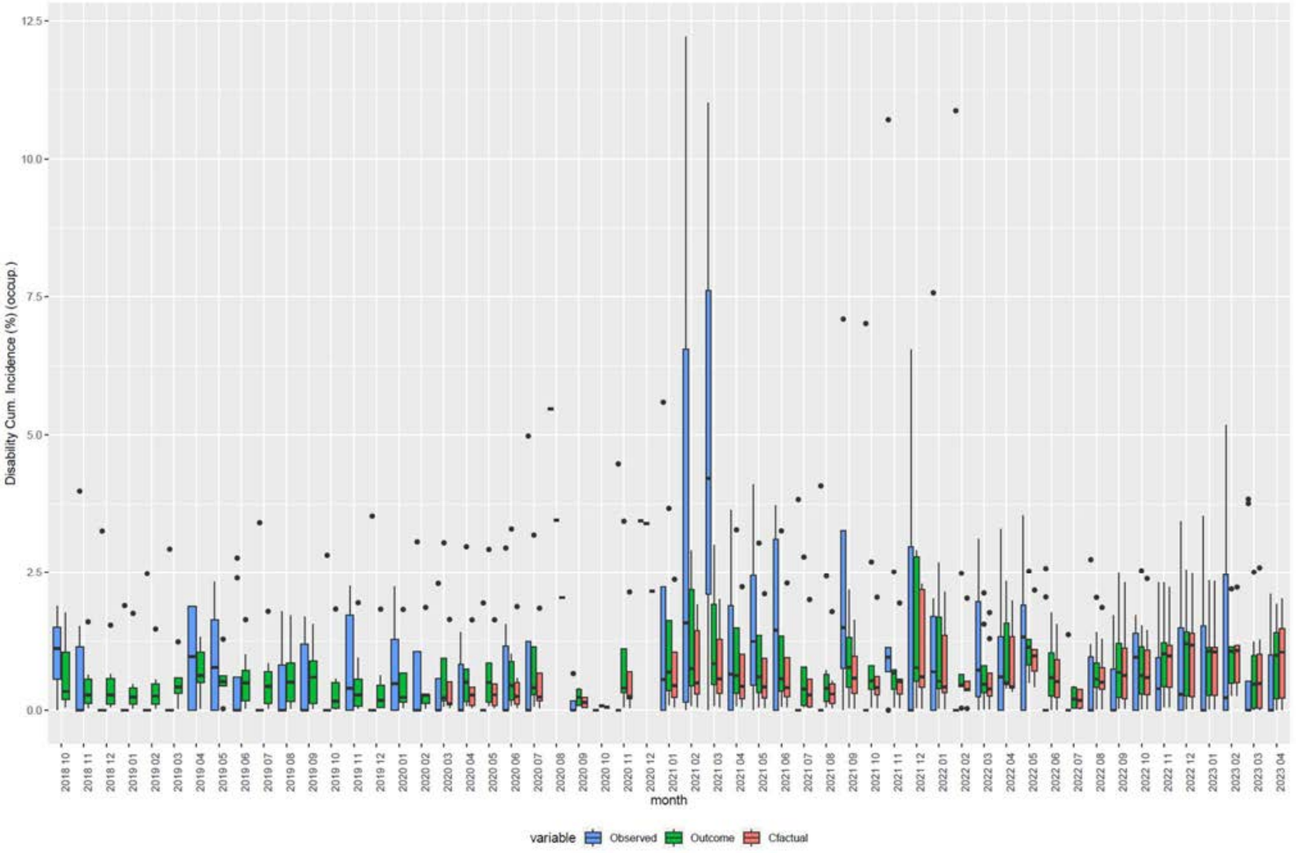

Legend: Observed: observed crude incidence proportions, i.e. cases of individuals with one or more new diagnoses defined by the indicator variable divided by the total number of refugee centre inhabitants (occupancy) and multiplied by 100. Outcome: estimated outcome values based on negative binomial regression models, adjusted for age, sex, centre, and secular trends. Cfactual: estimated counterfactual values based on the negative binomial regression models, adjusted for age, sex, centre and secular trends while setting covid = 0. Y-axis: cumulative incidence in %. Boxes: interquartile range (IQR; the 25th and 75th percentiles). Whiskers: The upper whisker extends from the hinge to the largest value no further than 1.5 * IQR from the hinge. The lower whisker extends from the hinge to the smallest value at most 1.5 * IQR of the hinge. Data beyond the end of the whiskers are plotted individually by black dots. Horizontal black bar in boxes: Median. N = 836 centre-months. Source data are provided in the 'source_data.xlsx' file.

1.2.2. Figure S2 - Box plot: Injury, poisoning and certain other consequences of external causes (short label: Cons.ext.causes)

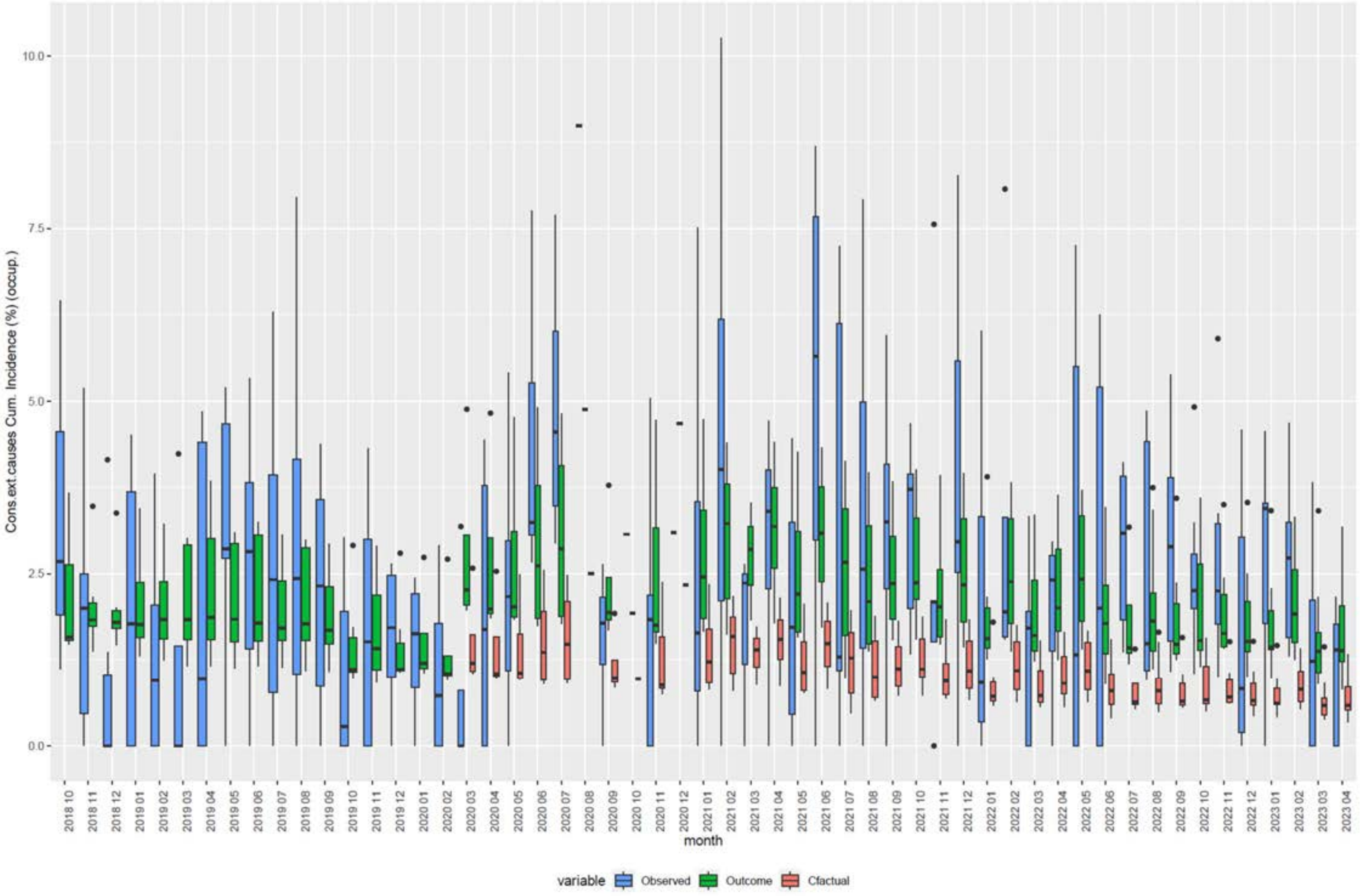

Legend: Observed: observed crude incidence proportions, i.e. cases of individuals with one or more new diagnoses defined by the indicator variable divided by the total number of refugee centre inhabitants (occupancy) and multiplied by 100. Outcome: estimated outcome values based on negative binomial regression models, adjusted for age, sex, centre, and secular trends. Cfactual: estimated counterfactual values based on the negative binomial regression models, adjusted for age, sex, centre and secular trends while setting covid = 0. Y-axis: cumulative incidence in %. Boxes: interquartile range (IQR; the 25th and 75th percentiles). Whiskers: The upper whisker extends from the hinge to the largest value no further than 1.5 * IQR from the hinge. The lower whisker extends from the hinge to the smallest value at most 1.5 * IQR of the hinge. Data beyond the end of the whiskers are plotted individually by black dots. Horizontal black bar in boxes: Median. N = 836 centre-months. Source data are provided in the 'source_data.xlsx' file.

1.2.3. Figure S3 - Box plot: Diseases of the skin and subcutaneous tissue (short label: Skin)

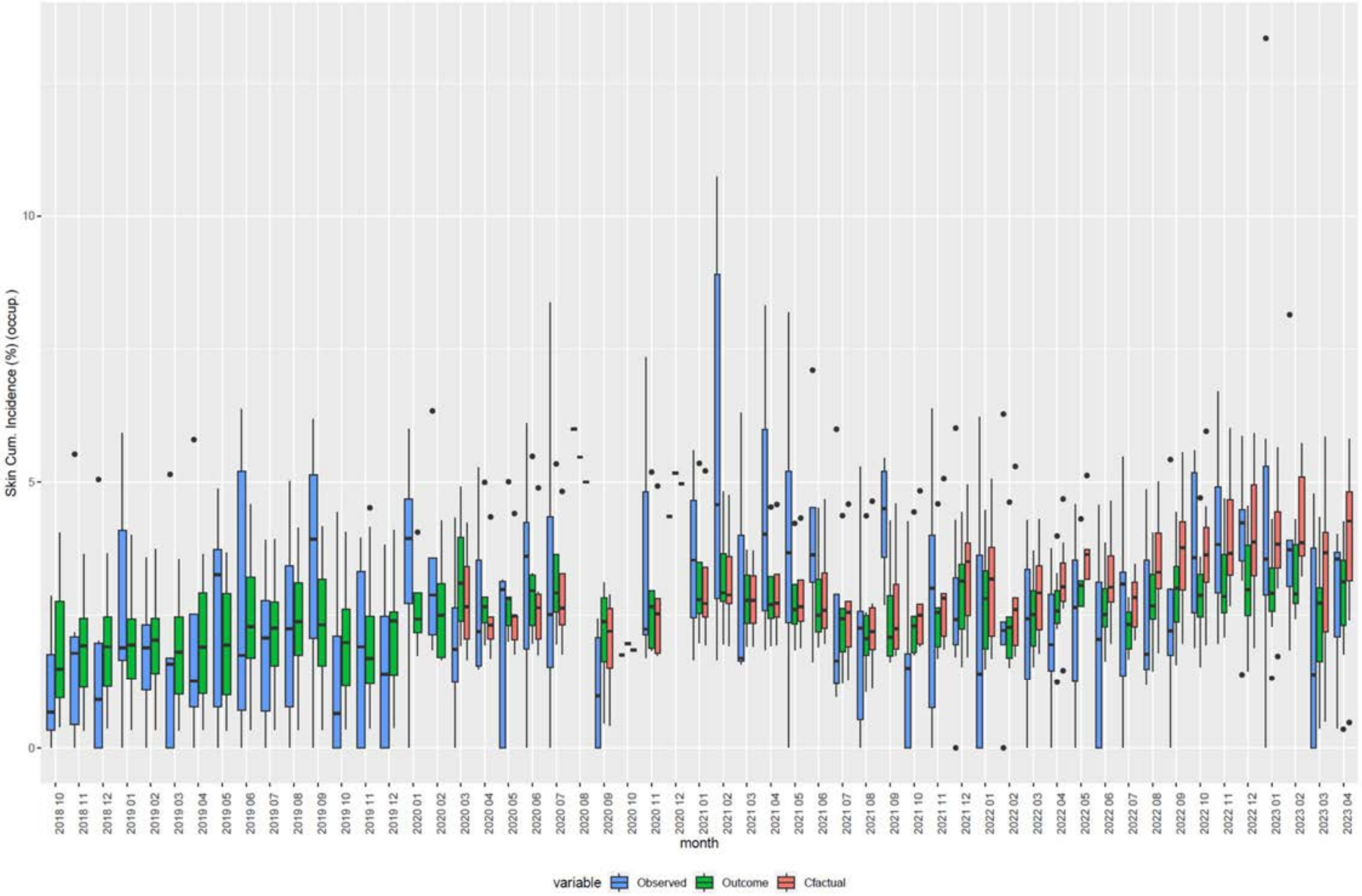

Legend: Observed: observed crude incidence proportions, i.e. cases of individuals with one or more new diagnoses defined by the indicator variable divided by the total number of refugee centre inhabitants (occupancy) and multiplied by 100. Outcome: estimated outcome values based on negative binomial regression models, adjusted for age, sex, centre, and secular trends. Cfactual: estimated counterfactual values based on the negative binomial regression models, adjusted for age, sex, centre and secular trends while setting covid = 0. Y-axis: cumulative incidence in %. Boxes: interquartile range (IQR; the 25th and 75th percentiles). Whiskers: The upper whisker extends from the hinge to the largest value no further than 1.5 * IQR from the hinge. The lower whisker extends from the hinge to the smallest value at most 1.5 * IQR of the hinge. Data beyond the end of the whiskers are plotted individually by black dots. Horizontal black bar in boxes: Median. N = 836 centre-months. Source data are provided in the 'source_data.xlsx' file.

1.2.4. Figure S4 - Box plot: Diseases of the digestive system (short label: Digestive syst.)

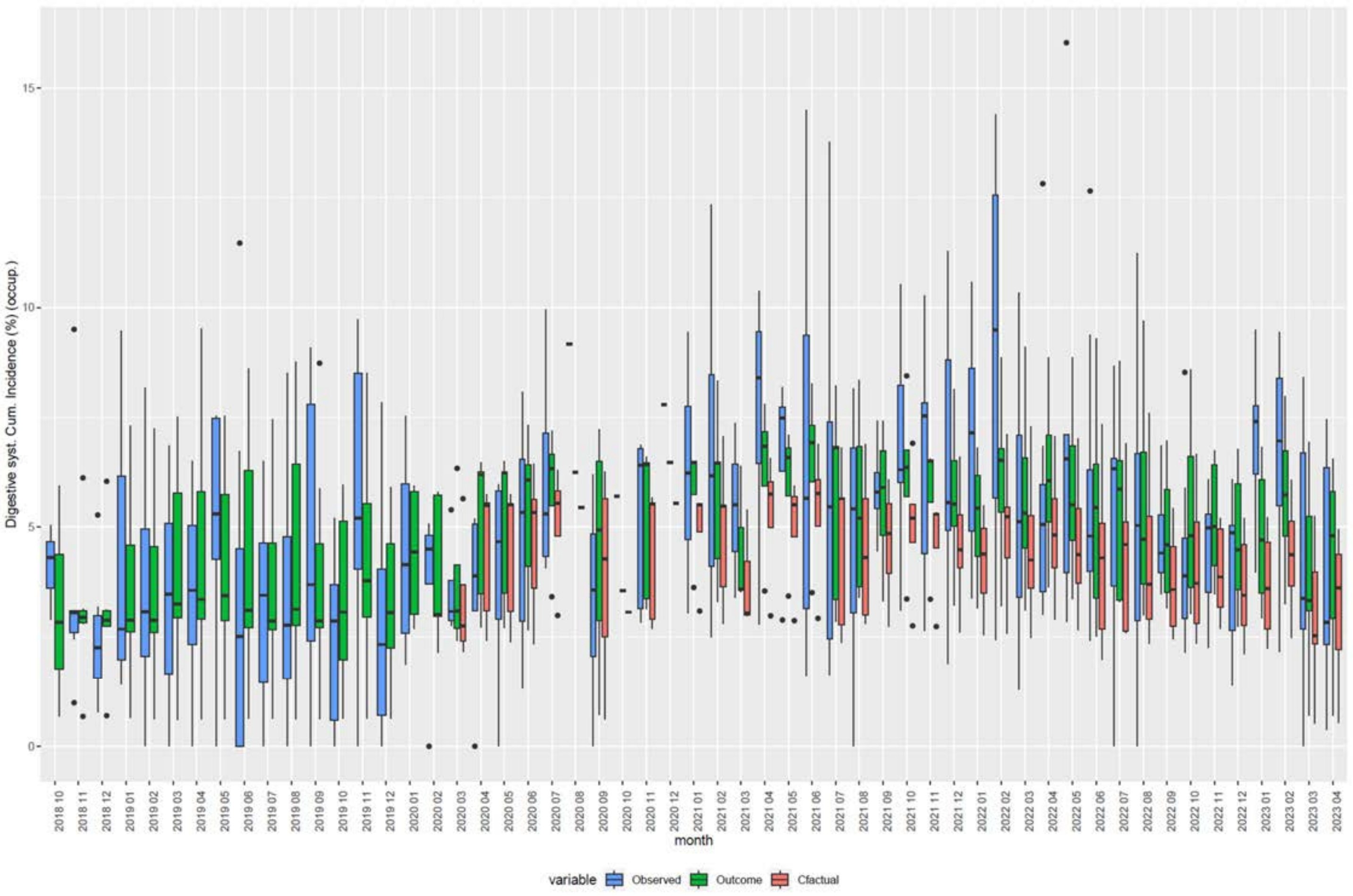

Legend: Observed: observed crude incidence proportions, i.e. cases of individuals with one or more new diagnoses defined by the indicator variable divided by the total number of refugee centre inhabitants (occupancy) and multiplied by 100. Outcome: estimated outcome values based on negative binomial regression models, adjusted for age, sex, centre, and secular trends. Cfactual: estimated counterfactual values based on the negative binomial regression models, adjusted for age, sex, centre and secular trends while setting covid = 0. Y-axis: cumulative incidence in %. Boxes: interquartile range (IQR; the 25th and 75th percentiles). Whiskers: The upper whisker extends from the hinge to the largest value no further than 1.5 * IQR from the hinge. The lower whisker extends from the hinge to the smallest value at most 1.5 * IQR of the hinge. Data beyond the end of the whiskers are plotted individually by black dots. Horizontal black bar in boxes: Median. N = 836 centre-months. Source data are provided in the 'source_data.xlsx' file.

1.2.5. Figure S5 - Box plot: Diseases of the blood and blood-forming organs and certain disorders involving the immune mechanism (short label: Blood)

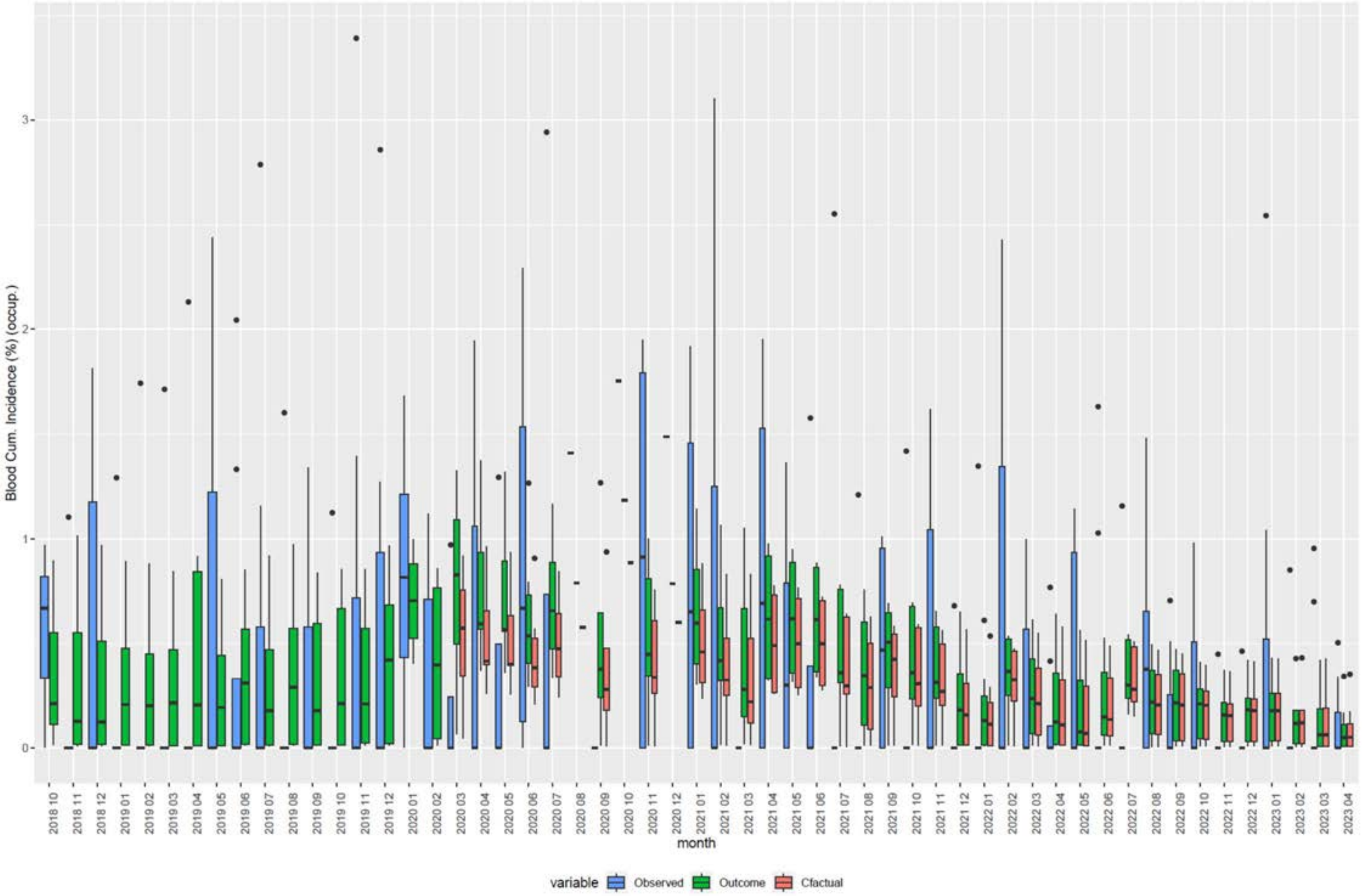

Legend: Observed: observed crude incidence proportions, i.e. cases of individuals with one or more new diagnoses defined by the indicator variable divided by the total number of refugee centre inhabitants (occupancy) and multiplied by 100. Outcome: estimated outcome values based on negative binomial regression models, adjusted for age, sex, centre, and secular trends. Cfactual: estimated counterfactual values based on the negative binomial regression models, adjusted for age, sex, centre and secular trends while setting covid = 0. Y-axis: cumulative incidence in %. Boxes: interquartile range (IQR; the 25th and 75th percentiles). Whiskers: The upper whisker extends from the hinge to the largest value no further than 1.5 * IQR from the hinge. The lower whisker extends from the hinge to the smallest value at most 1.5 * IQR of the hinge. Data beyond the end of the whiskers are plotted individually by black dots. Horizontal black bar in boxes: Median. N = 836 centre-months. Source data are provided in the 'source_data.xlsx' file.

1.2.6. Figure S6 - Box plot: Certain infectious and parasitic diseases (short label: Inf.diseases)

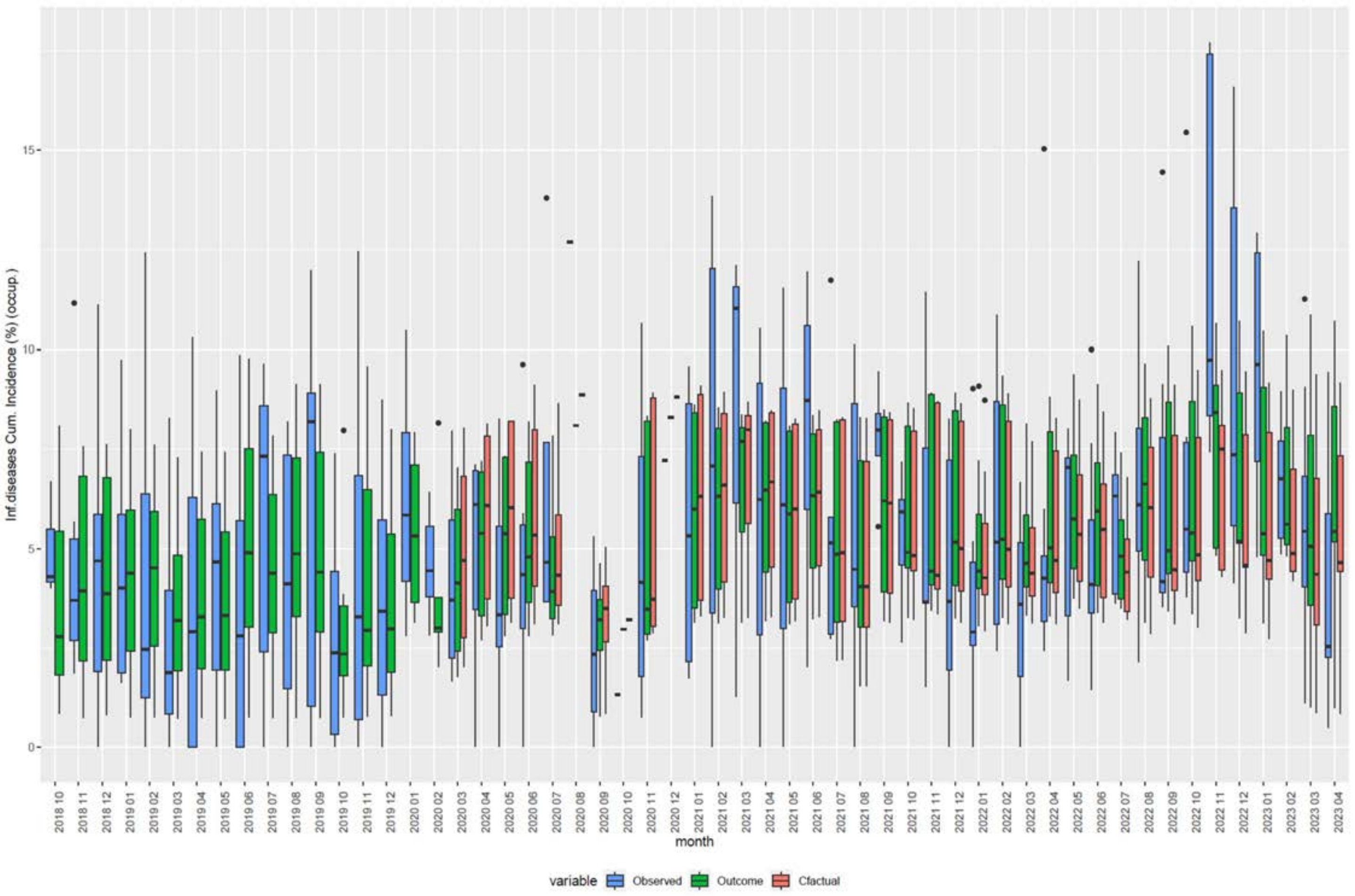

Legend: Observed: observed crude incidence proportions, i.e. cases of individuals with one or more new diagnoses defined by the indicator variable divided by the total number of refugee centre inhabitants (occupancy) and multiplied by 100. Outcome: estimated outcome values based on negative binomial regression models, adjusted for age, sex, centre, and secular trends. Cfactual: estimated counterfactual values based on the negative binomial regression models, adjusted for age, sex, centre and secular trends while setting covid = 0. Y-axis: cumulative incidence in %. Boxes: interquartile range (IQR; the 25th and 75th percentiles). Whiskers: The upper whisker extends from the hinge to the largest value no further than 1.5 * IQR from the hinge. The lower whisker extends from the hinge to the smallest value at most 1.5 * IQR of the hinge. Data beyond the end of the whiskers are plotted individually by black dots. Horizontal black bar in boxes: Median. N = 836 centre-months. Source data are provided in the 'source_data.xlsx' file.

1.2.7. Figure S7 - Box plot: Notifiable infectious diseases (short label: Inf.notify)

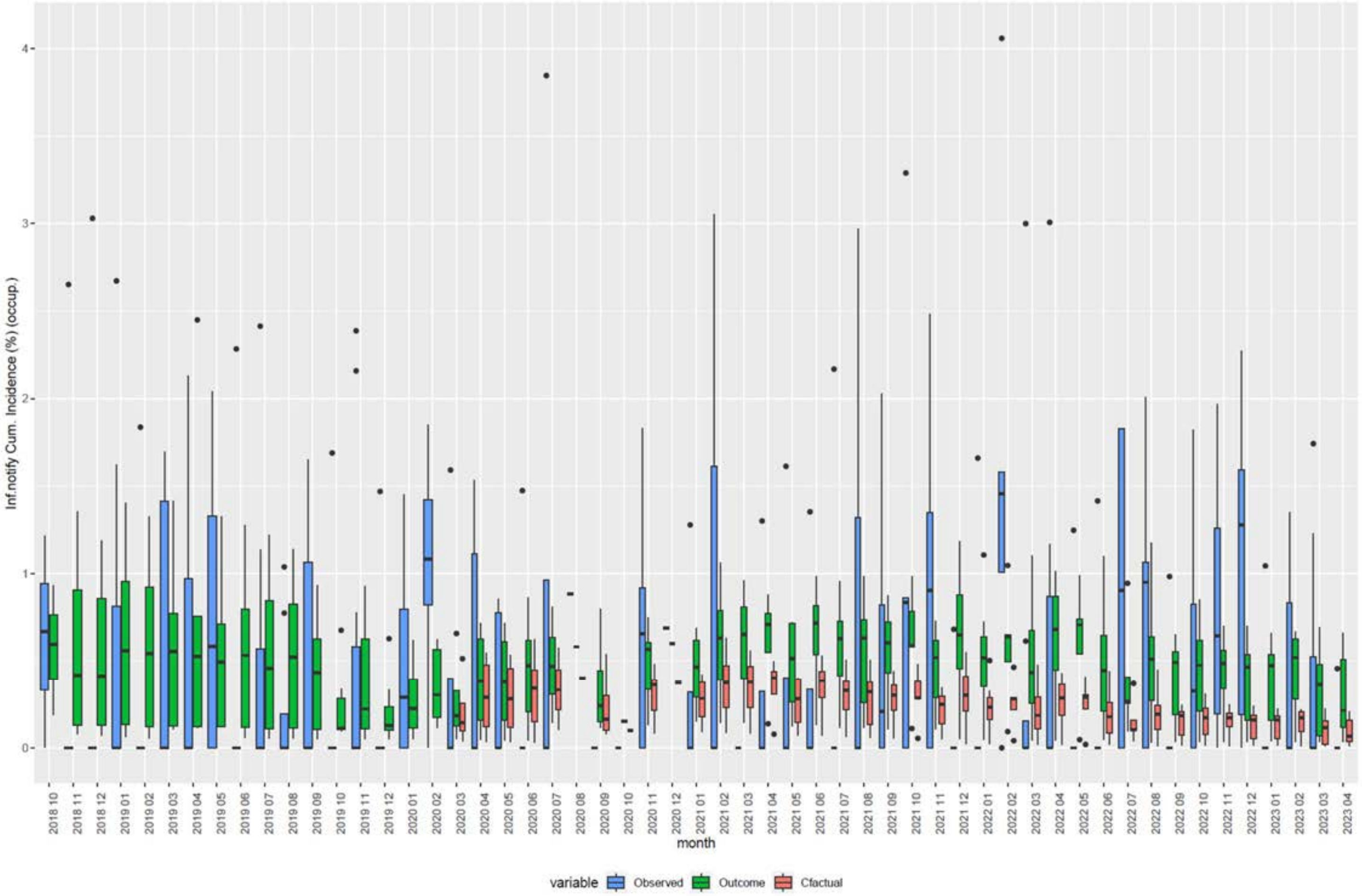

Legend: Observed: observed crude incidence proportions, i.e. cases of individuals with one or more new diagnoses defined by the indicator variable divided by the total number of refugee centre inhabitants (occupancy) and multiplied by 100. Outcome: estimated outcome values based on negative binomial regression models, adjusted for age, sex, centre, and secular trends. Cfactual: estimated counterfactual values based on the negative binomial regression models, adjusted for age, sex, centre and secular trends while setting covid = 0. Y-axis: cumulative incidence in %. Boxes: interquartile range (IQR; the 25th and 75th percentiles). Whiskers: The upper whisker extends from the hinge to the largest value no further than 1.5 * IQR from the hinge. The lower whisker extends from the hinge to the smallest value at most 1.5 * IQR of the hinge. Data beyond the end of the whiskers are plotted individually by black dots. Horizontal black bar in boxes: Median. N = 836 centre-months. Source data are provided in the 'source_data.xlsx' file.

1.2.8. Figure S8 - Box plot: Diseases of the circulatory system (short label: Circulatory syst.)

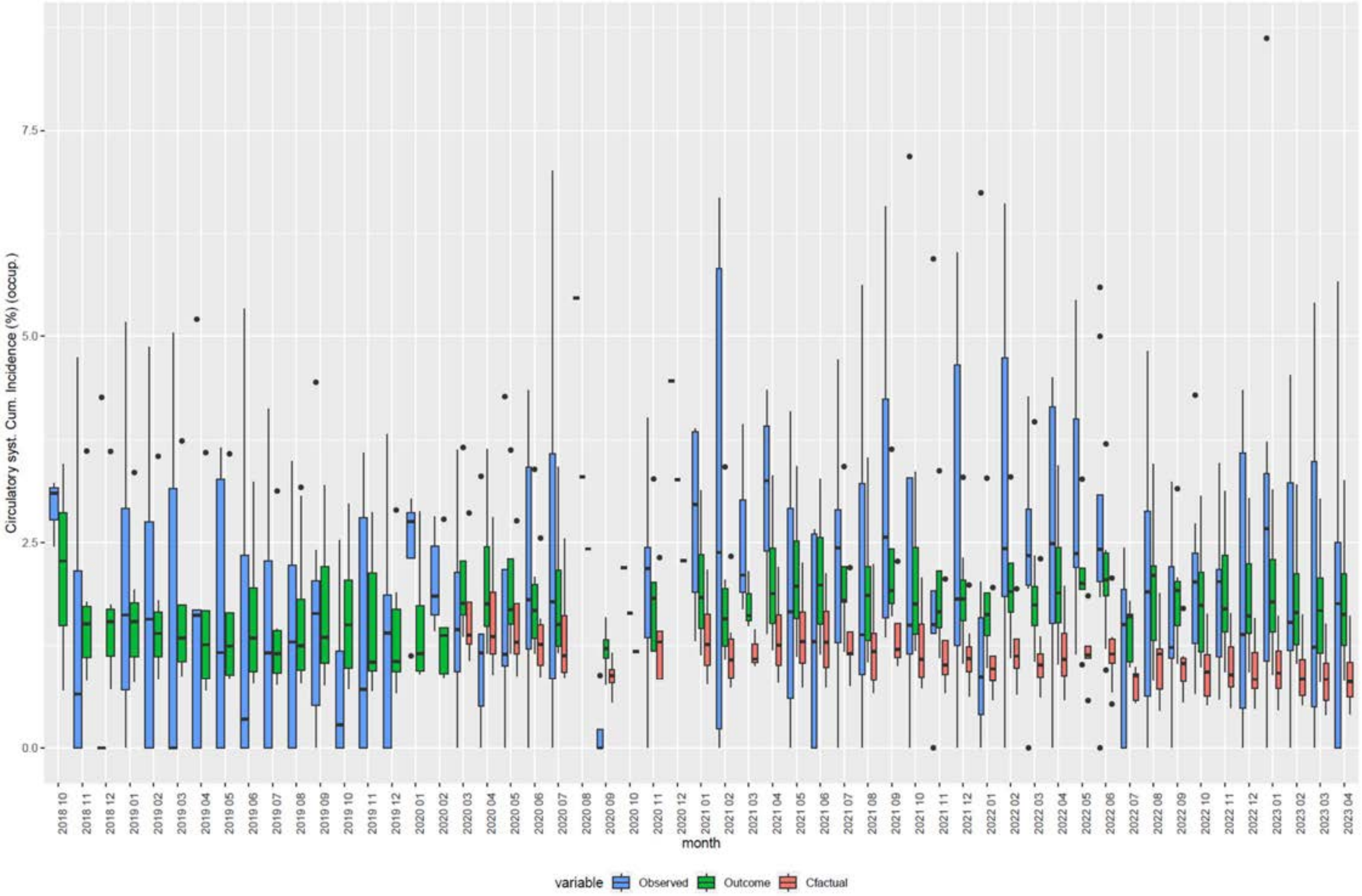

Legend: Observed: observed crude incidence proportions, i.e. cases of individuals with one or more new diagnoses defined by the indicator variable divided by the total number of refugee centre inhabitants (occupancy) and multiplied by 100. Outcome: estimated outcome values based on negative binomial regression models, adjusted for age, sex, centre, and secular trends. Cfactual: estimated counterfactual values based on the negative binomial regression models, adjusted for age, sex, centre and secular trends while setting covid = 0. Y-axis: cumulative incidence in %. Boxes: interquartile range (IQR; the 25th and 75th percentiles). Whiskers: The upper whisker extends from the hinge to the largest value no further than 1.5 * IQR from the hinge. The lower whisker extends from the hinge to the smallest value at most 1.5 * IQR of the hinge. Data beyond the end of the whiskers are plotted individually by black dots. Horizontal black bar in boxes: Median. N = 836 centre-months. Source data are provided in the 'source_data.xlsx' file.

1.2.9. Figure S9 - Box plot: Hypertension (short label: Hypertension)

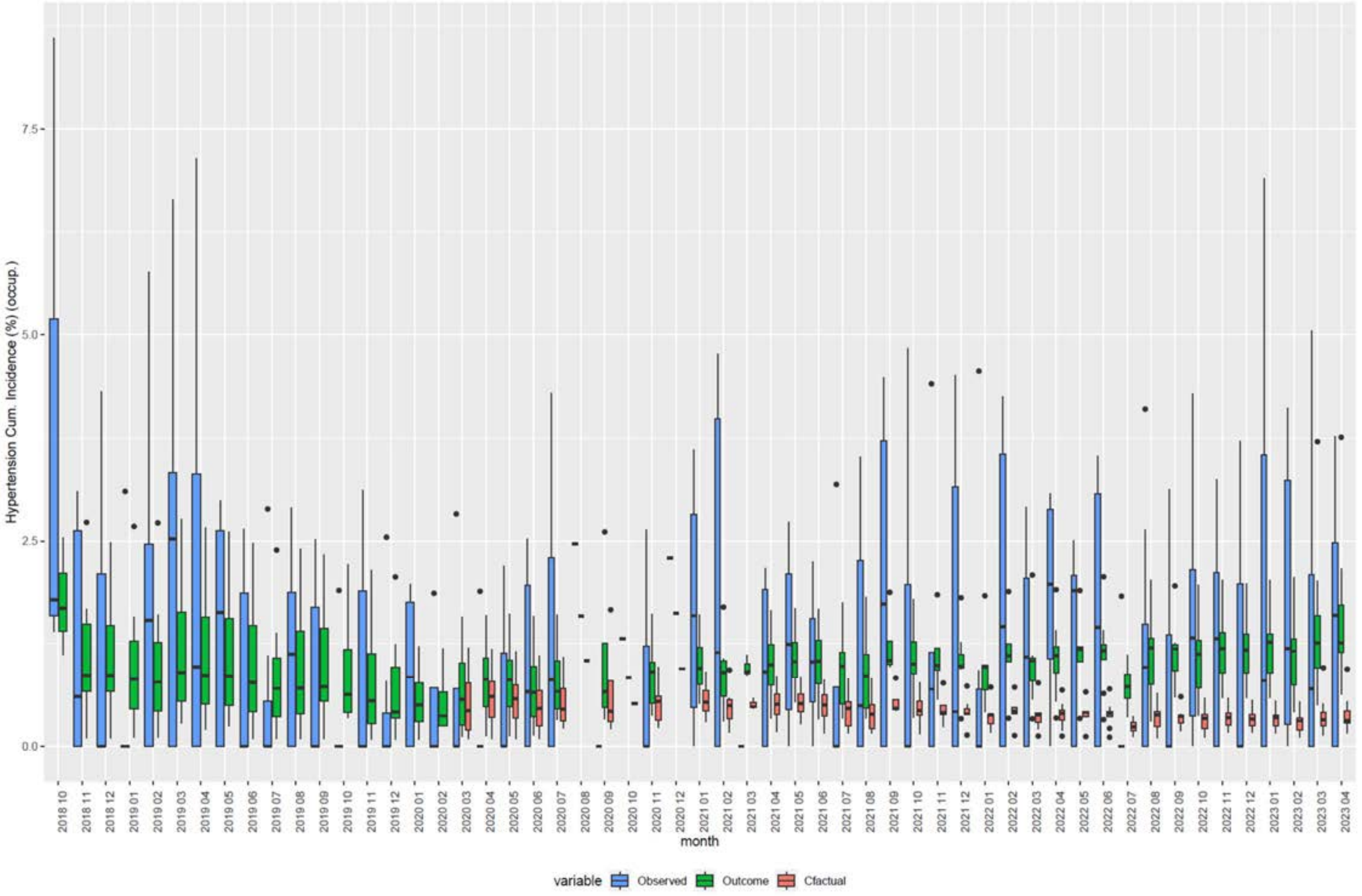

Legend: Observed: observed crude incidence proportions, i.e. cases of individuals with one or more new diagnoses defined by the indicator variable divided by the total number of refugee centre inhabitants (occupancy) and multiplied by 100. Outcome: estimated outcome values based on negative binomial regression models, adjusted for age, sex, centre, and secular trends. Cfactual: estimated counterfactual values based on the negative binomial regression models, adjusted for age, sex, centre and secular trends while setting covid = 0. Y-axis: cumulative incidence in %. Boxes: interquartile range (IQR; the 25th and 75th percentiles). Whiskers: The upper whisker extends from the hinge to the largest value no further than 1.5 * IQR from the hinge. The lower whisker extends from the hinge to the smallest value at most 1.5 * IQR of the hinge. Data beyond the end of the whiskers are plotted individually by black dots. Horizontal black bar in boxes: Median. N = 836 centre-months. Source data are provided in the 'source_data.xlsx' file.

1.2.10. Figure S10 - Box plot: Endocrine, nutritional and metabolic diseases (short label: Metabolic)

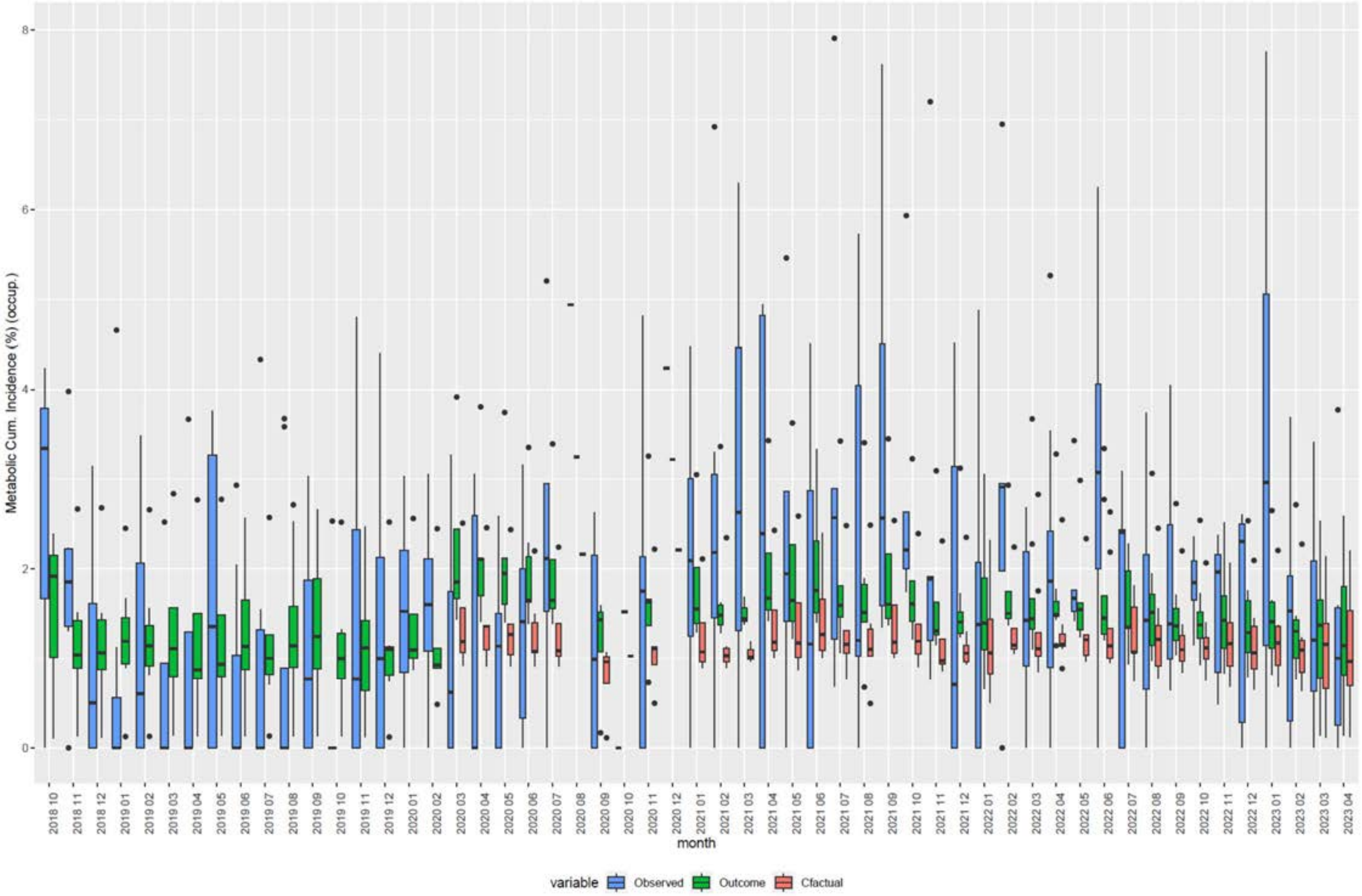

Legend: Observed: observed crude incidence proportions, i.e. cases of individuals with one or more new diagnoses defined by the indicator variable divided by the total number of refugee centre inhabitants (occupancy) and multiplied by 100. Outcome: estimated outcome values based on negative binomial regression models, adjusted for age, sex, centre, and secular trends. Cfactual: estimated counterfactual values based on the negative binomial regression models, adjusted for age, sex, centre and secular trends while setting covid = 0. Y-axis: cumulative incidence in %. Boxes: interquartile range (IQR; the 25th and 75th percentiles). Whiskers: The upper whisker extends from the hinge to the largest value no further than 1.5 * IQR from the hinge. The lower whisker extends from the hinge to the smallest value at most 1.5 * IQR of the hinge. Data beyond the end of the whiskers are plotted individually by black dots. Horizontal black bar in boxes: Median. N = 836 centre-months. Source data are provided in the 'source_data.xlsx' file.

1.2.11. Figure S11 - Box plot: Diabetes mellitus (short label: Diabetes)

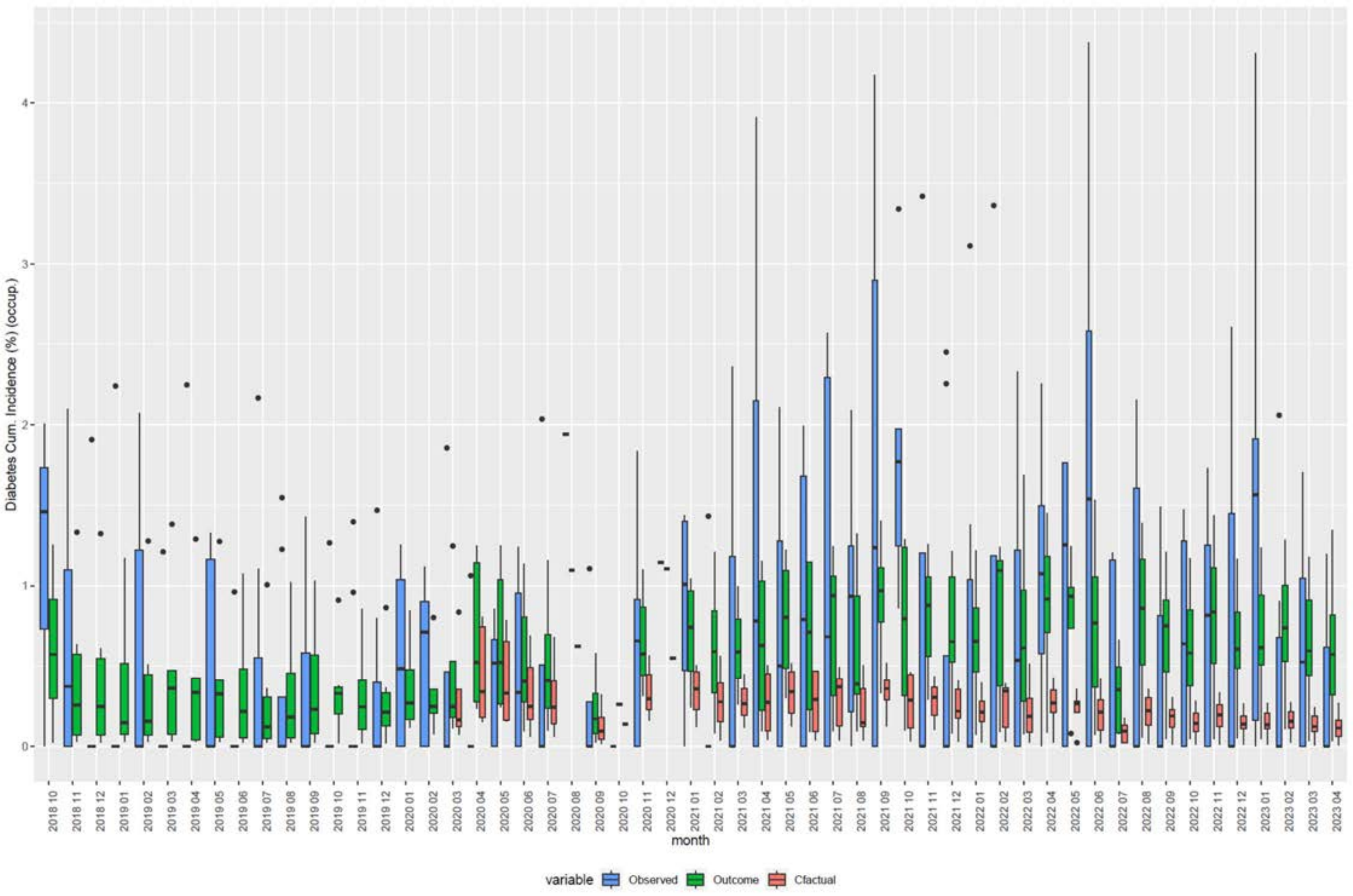

Legend: Observed: observed crude incidence proportions, i.e. cases of individuals with one or more new diagnoses defined by the indicator variable divided by the total number of refugee centre inhabitants (occupancy) and multiplied by 100. Outcome: estimated outcome values based on negative binomial regression models, adjusted for age, sex, centre, and secular trends. Cfactual: estimated counterfactual values based on the negative binomial regression models, adjusted for age, sex, centre and secular trends while setting covid = 0. Y-axis: cumulative incidence in %. Boxes: interquartile range (IQR; the 25th and 75th percentiles). Whiskers: The upper whisker extends from the hinge to the largest value no further than 1.5 * IQR from the hinge. The lower whisker extends from the hinge to the smallest value at most 1.5 * IQR of the hinge. Data beyond the end of the whiskers are plotted individually by black dots. Horizontal black bar in boxes: Median. N = 836 centre-months. Source data are provided in the 'source_data.xlsx' file.

1.2.12. Figure S12 - Box plot: Diseases of the musculoskeletal system and connective tissue (short label: Musculoskelet. syst.)

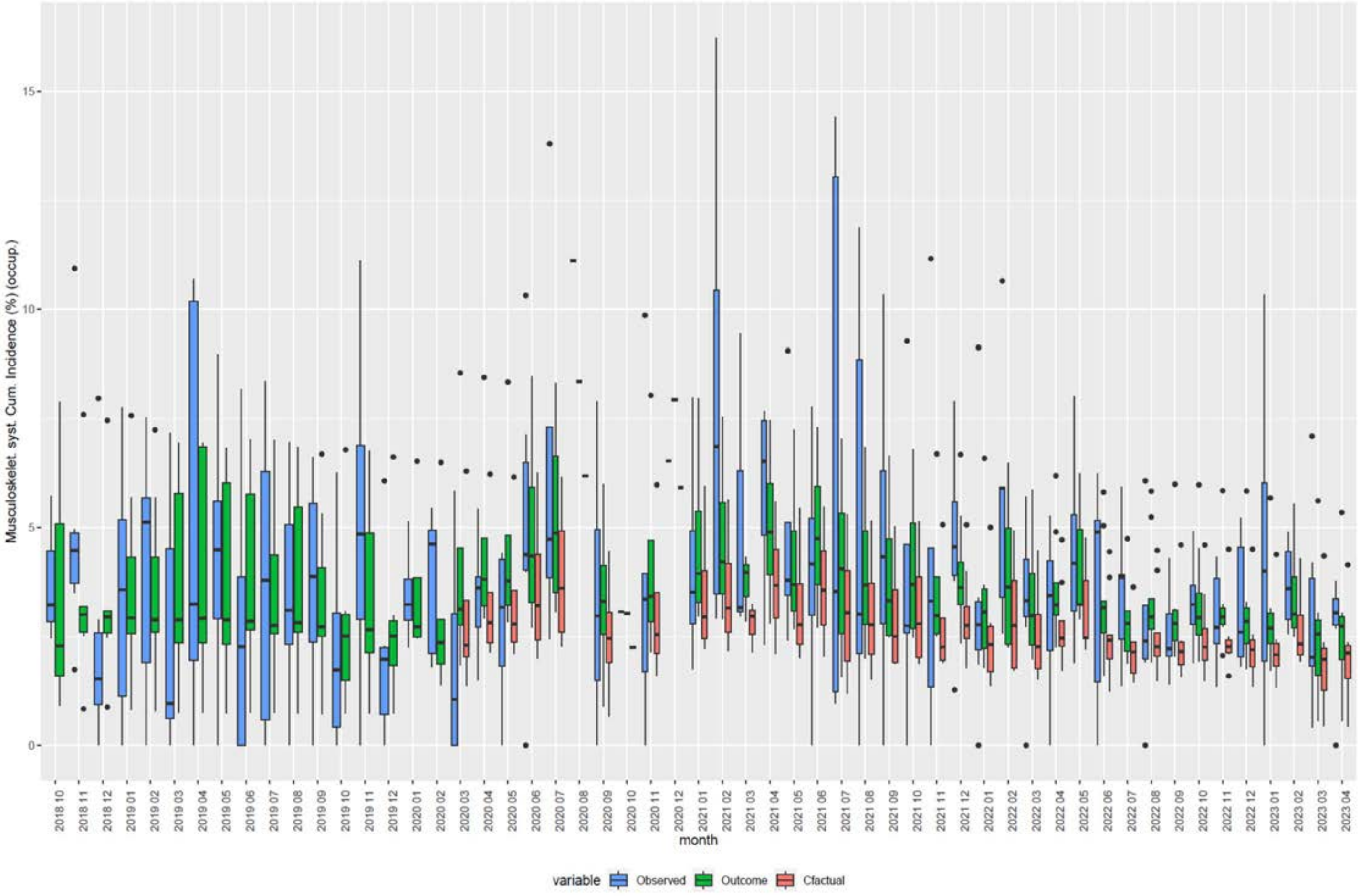

Legend: Observed: observed crude incidence proportions, i.e. cases of individuals with one or more new diagnoses defined by the indicator variable divided by the total number of refugee centre inhabitants (occupancy) and multiplied by 100. Outcome: estimated outcome values based on negative binomial regression models, adjusted for age, sex, centre, and secular trends. Cfactual: estimated counterfactual values based on the negative binomial regression models, adjusted for age, sex, centre and secular trends while setting covid = 0. Y-axis: cumulative incidence in %. Boxes: interquartile range (IQR; the 25th and 75th percentiles). Whiskers: The upper whisker extends from the hinge to the largest value no further than 1.5 * IQR from the hinge. The lower whisker extends from the hinge to the smallest value at most 1.5 * IQR of the hinge. Data beyond the end of the whiskers are plotted individually by black dots. Horizontal black bar in boxes: Median. N = 836 centre-months. Source data are provided in the 'source_data.xlsx' file.

1.2.13. Figure S13 - Box plot: Neoplasms (short label: Neoplasm)

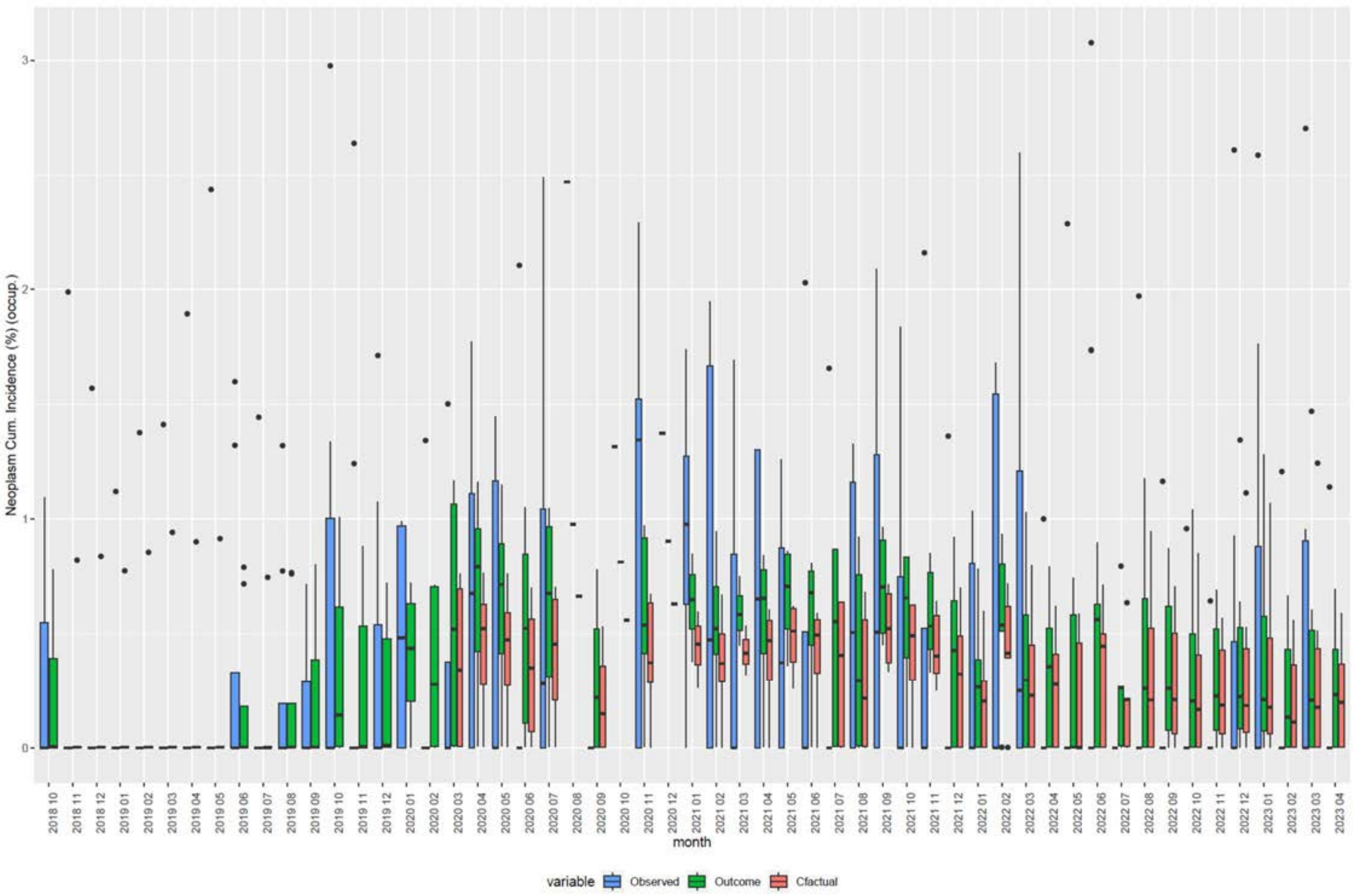

Legend: Observed: observed crude incidence proportions, i.e. cases of individuals with one or more new diagnoses defined by the indicator variable divided by the total number of refugee centre inhabitants (occupancy) and multiplied by 100. Outcome: estimated outcome values based on negative binomial regression models, adjusted for age, sex, centre, and secular trends. Cfactual: estimated counterfactual values based on the negative binomial regression models, adjusted for age, sex, centre and secular trends while setting covid = 0. Y-axis: cumulative incidence in %. Boxes: interquartile range (IQR; the 25th and 75th percentiles). Whiskers: The upper whisker extends from the hinge to the largest value no further than 1.5 * IQR from the hinge. The lower whisker extends from the hinge to the smallest value at most 1.5 * IQR of the hinge. Data beyond the end of the whiskers are plotted individually by black dots. Horizontal black bar in boxes: Median. N = 836 centre-months. Source data are provided in the 'source_data.xlsx' file.

1.2.14. Figure S14 - Box plot: Diseases of the nervous system (short label: Nervous syst.)

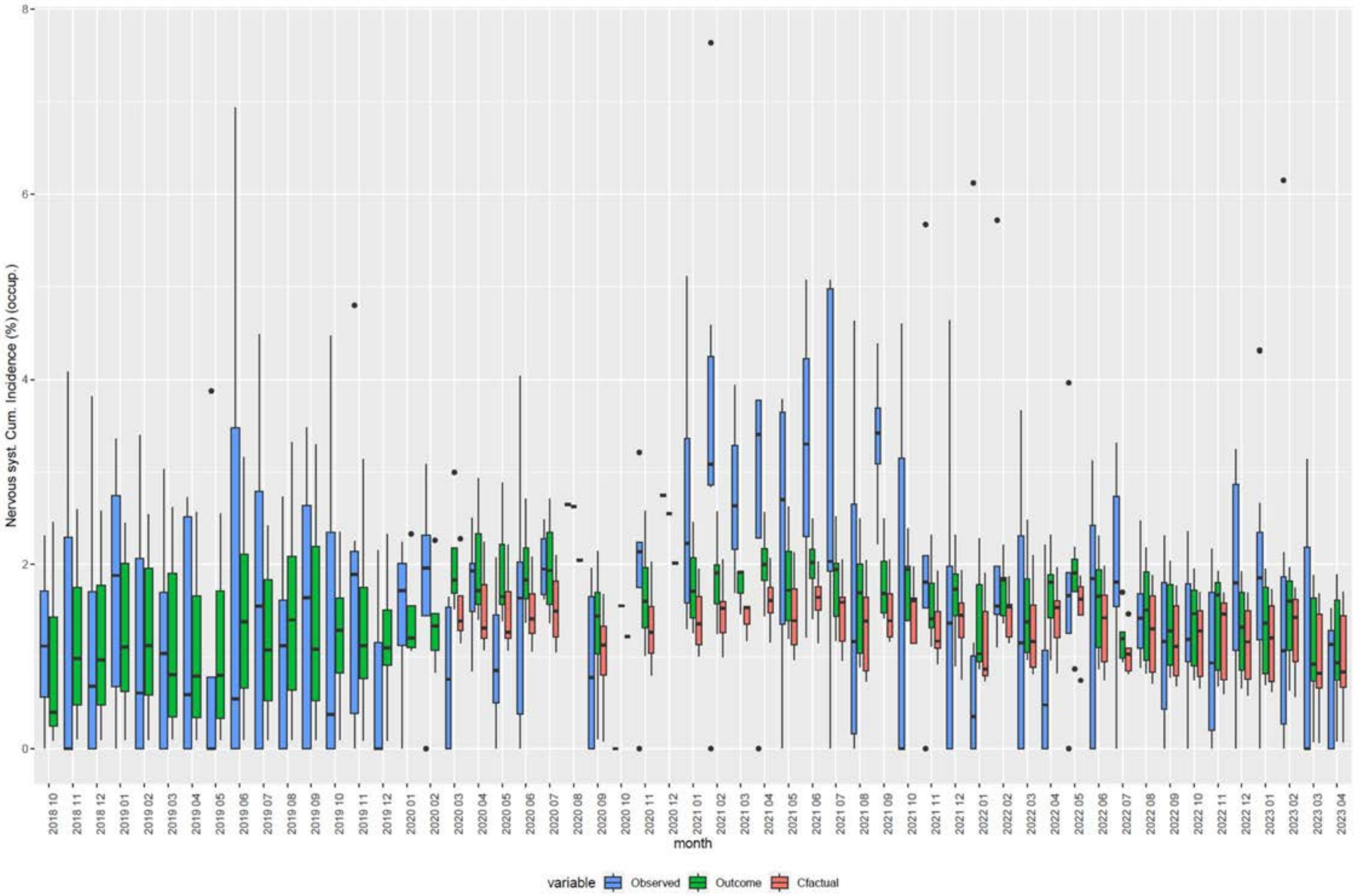

Legend: Observed: observed crude incidence proportions, i.e. cases of individuals with one or more new diagnoses defined by the indicator variable divided by the total number of refugee centre inhabitants (occupancy) and multiplied by 100. Outcome: estimated outcome values based on negative binomial regression models, adjusted for age, sex, centre, and secular trends. Cfactual: estimated counterfactual values based on the negative binomial regression models, adjusted for age, sex, centre and secular trends while setting covid = 0. Y-axis: cumulative incidence in %. Boxes: interquartile range (IQR; the 25th and 75th percentiles). Whiskers: The upper whisker extends from the hinge to the largest value no further than 1.5 * IQR from the hinge. The lower whisker extends from the hinge to the smallest value at most 1.5 * IQR of the hinge. Data beyond the end of the whiskers are plotted individually by black dots. Horizontal black bar in boxes: Median. N = 836 centre-months. Source data are provided in the 'source_data.xlsx' file.

1.2.15. Figure S15 - Box plot: Diseases of the ear and mastoid process (short label: Ear.mastoid)

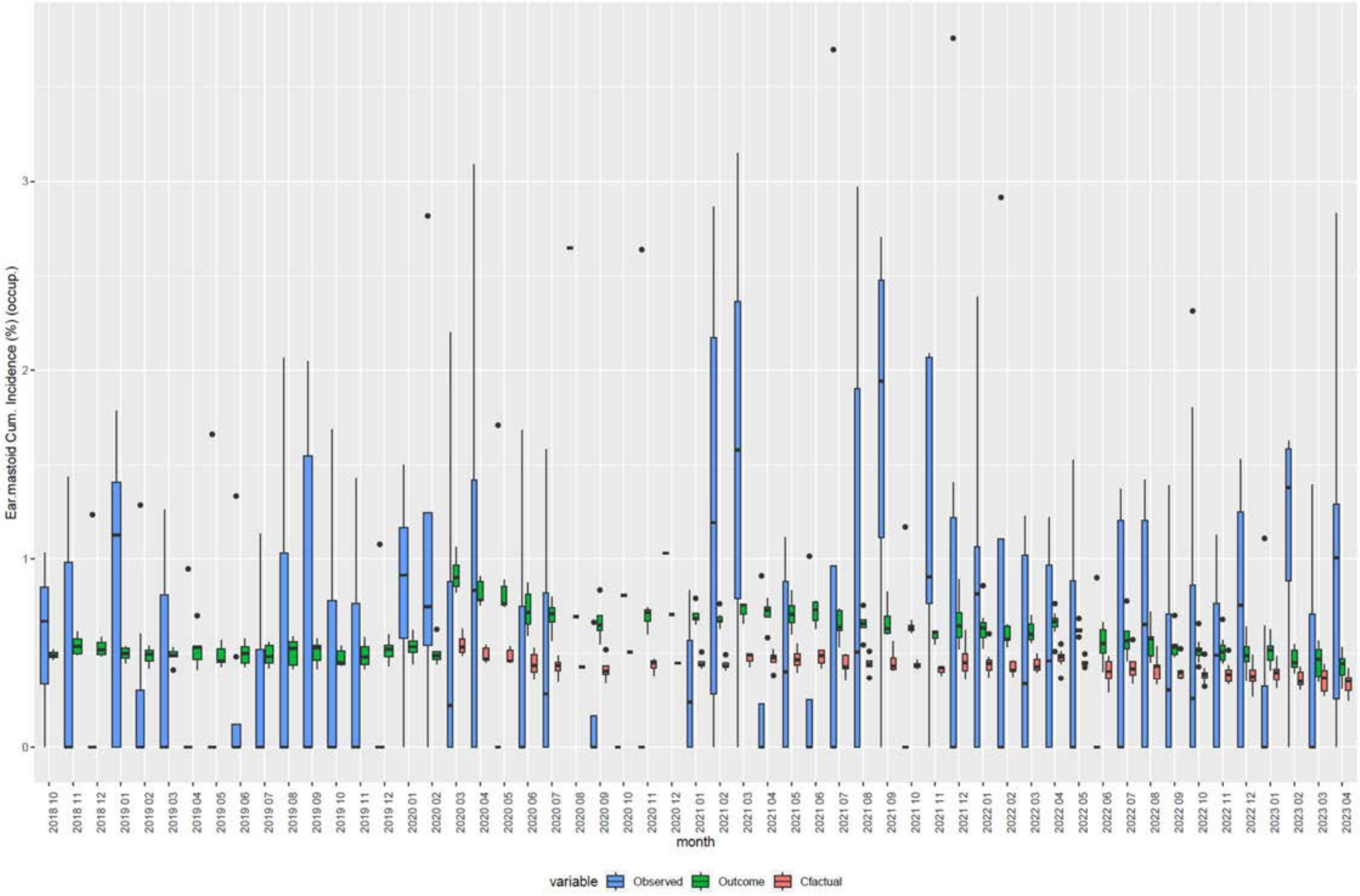

Legend: Observed: observed crude incidence proportions, i.e. cases of individuals with one or more new diagnoses defined by the indicator variable divided by the total number of refugee centre inhabitants (occupancy) and multiplied by 100. Outcome: estimated outcome values based on negative binomial regression models, adjusted for age, sex, centre, and secular trends. Cfactual: estimated counterfactual values based on the negative binomial regression models, adjusted for age, sex, centre and secular trends while setting covid = 0. Y-axis: cumulative incidence in %. Boxes: interquartile range (IQR; the 25th and 75th percentiles). Whiskers: The upper whisker extends from the hinge to the largest value no further than 1.5 * IQR from the hinge. The lower whisker extends from the hinge to the smallest value at most 1.5 * IQR of the hinge. Data beyond the end of the whiskers are plotted individually by black dots. Horizontal black bar in boxes: Median. N = 836 centre-months. Source data are provided in the 'source_data.xlsx' file.

1.2.16. Figure S16 - Box plot: Diseases of the eye and adnexa (short label: Eye.adnexa)

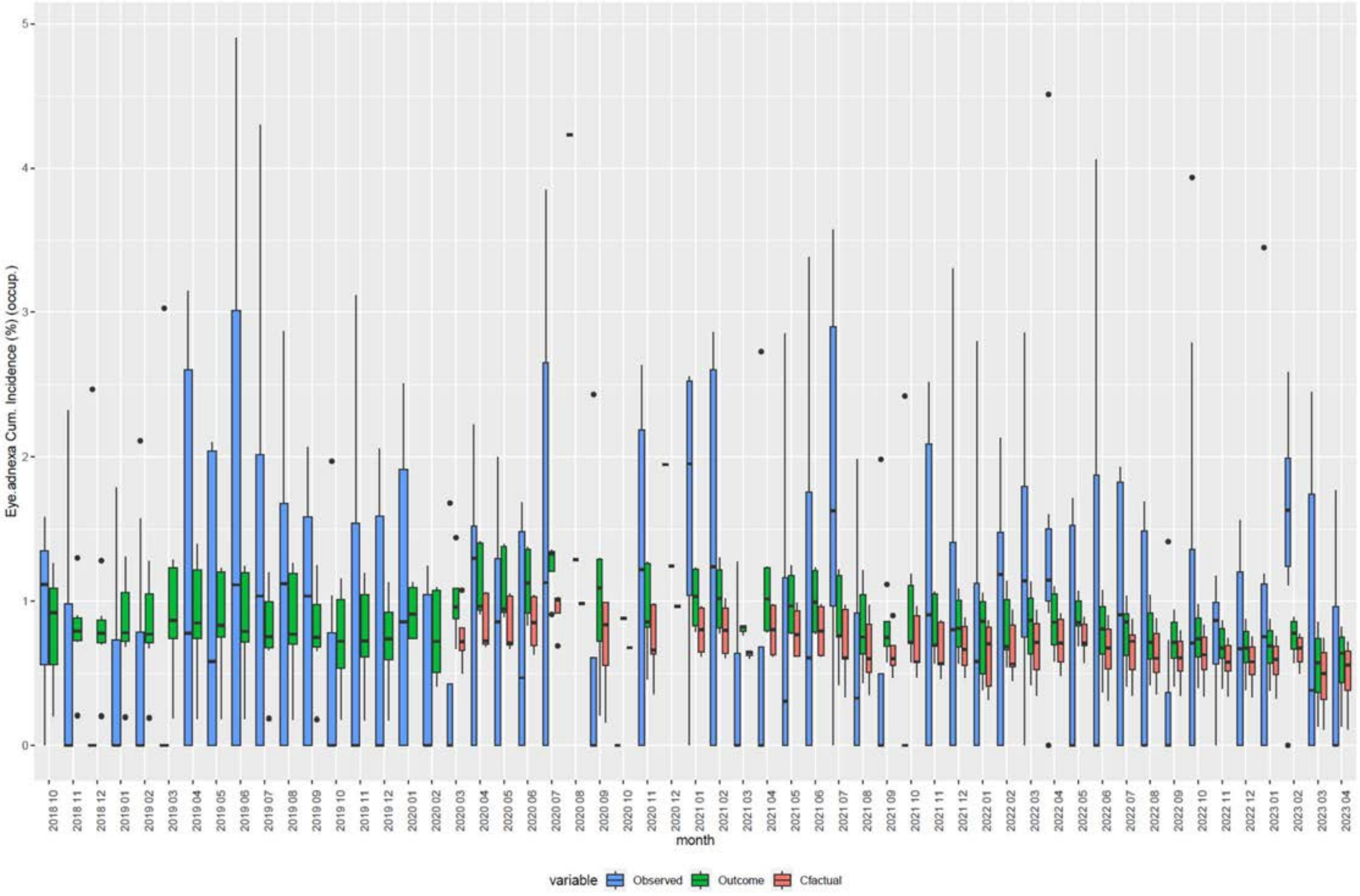

Legend: Observed: observed crude incidence proportions, i.e. cases of individuals with one or more new diagnoses defined by the indicator variable divided by the total number of refugee centre inhabitants (occupancy) and multiplied by 100. Outcome: estimated outcome values based on negative binomial regression models, adjusted for age, sex, centre, and secular trends. Cfactual: estimated counterfactual values based on the negative binomial regression models, adjusted for age, sex, centre and secular trends while setting covid = 0. Y-axis: cumulative incidence in %. Boxes: interquartile range (IQR; the 25th and 75th percentiles). Whiskers: The upper whisker extends from the hinge to the largest value no further than 1.5 * IQR from the hinge. The lower whisker extends from the hinge to the smallest value at most 1.5 * IQR of the hinge. Data beyond the end of the whiskers are plotted individually by black dots. Horizontal black bar in boxes: Median. N = 836 centre-months. Source data are provided in the 'source_data.xlsx' file.

1.2.17. Figure S17 - Box plot: Pregnancy, childbirth and the puerperium (short label: Pregn.condition)

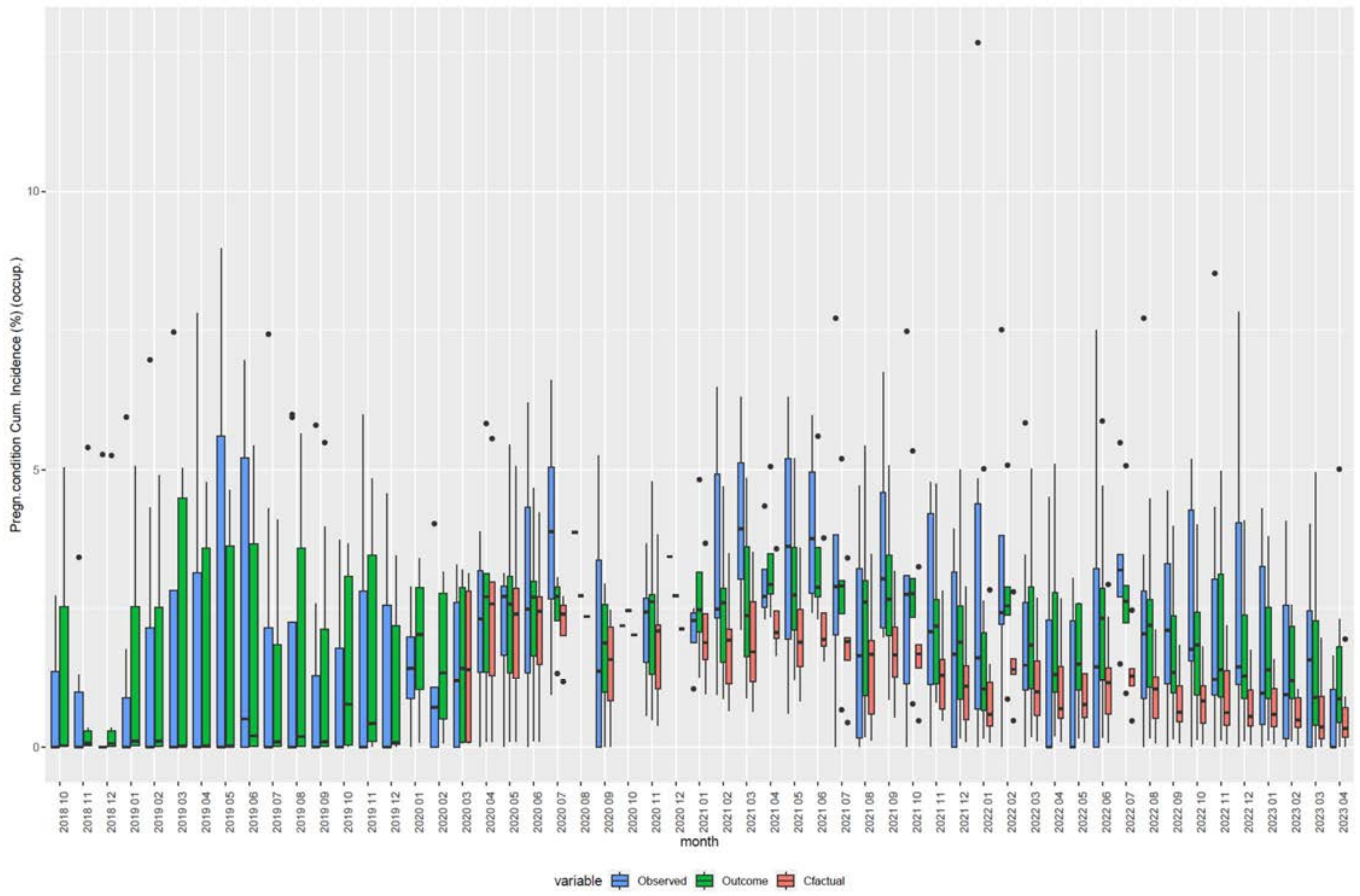

Legend: Observed: observed crude incidence proportions, i.e. cases of individuals with one or more new diagnoses defined by the indicator variable divided by the total number of refugee centre inhabitants (occupancy) and multiplied by 100. Outcome: estimated outcome values based on negative binomial regression models, adjusted for age, sex, centre, and secular trends. Cfactual: estimated counterfactual values based on the negative binomial regression models, adjusted for age, sex, centre and secular trends while setting covid = 0. Y-axis: cumulative incidence in %. Boxes: interquartile range (IQR; the 25th and 75th percentiles). Whiskers: The upper whisker extends from the hinge to the largest value no further than 1.5 * IQR from the hinge. The lower whisker extends from the hinge to the smallest value at most 1.5 * IQR of the hinge. Data beyond the end of the whiskers are plotted individually by black dots. Horizontal black bar in boxes: Median. N = 836 centre-months. Source data are provided in the 'source_data.xlsx' file.

1.2.18. Figure S18 - Box plot: Mental and behavioral disorders (short label: Psych.condition)

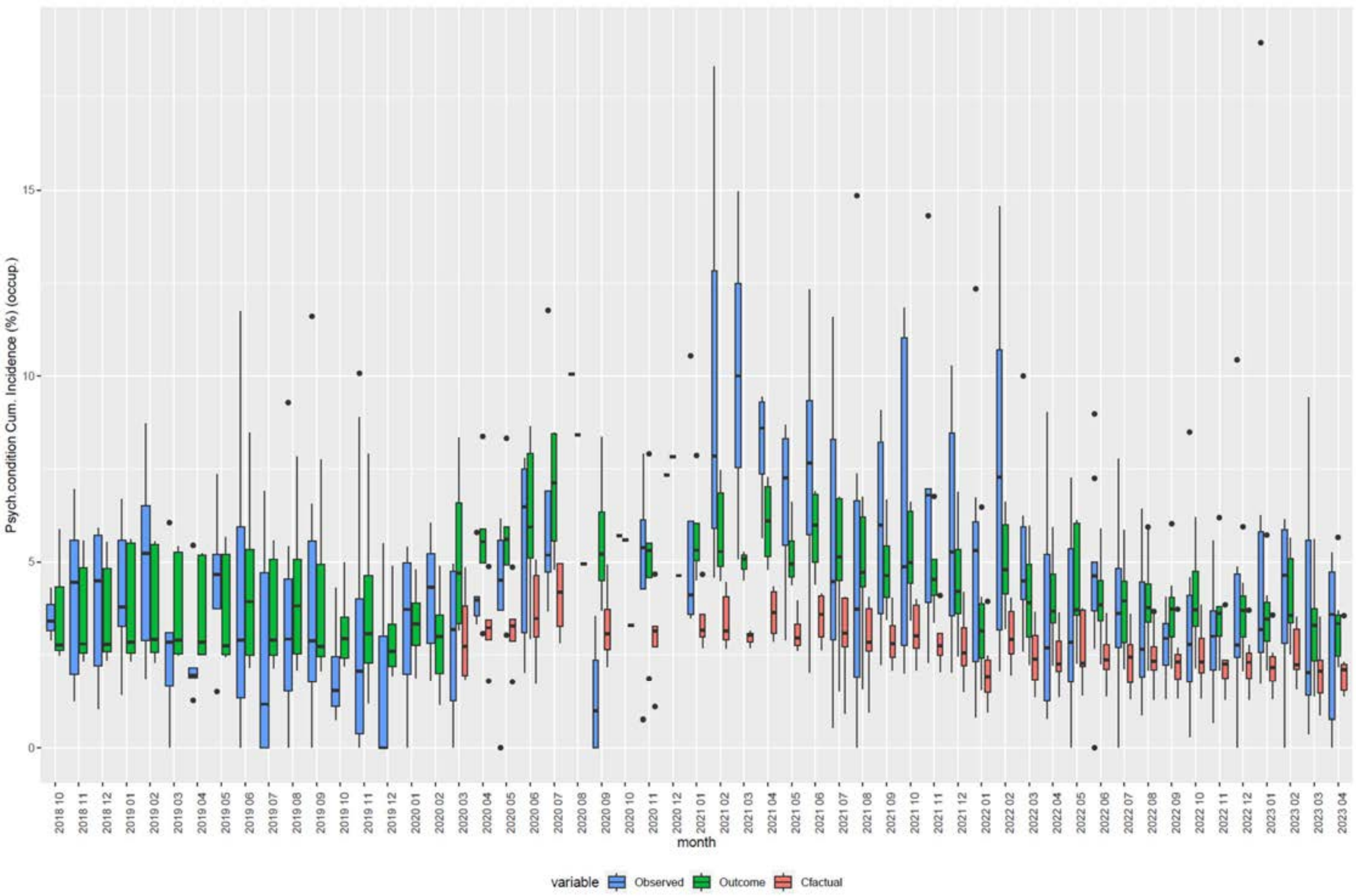

Legend: Observed: observed crude incidence proportions, i.e. cases of individuals with one or more new diagnoses defined by the indicator variable divided by the total number of refugee centre inhabitants (occupancy) and multiplied by 100. Outcome: estimated outcome values based on negative binomial regression models, adjusted for age, sex, centre, and secular trends. Cfactual: estimated counterfactual values based on the negative binomial regression models, adjusted for age, sex, centre and secular trends while setting covid = 0. Y-axis: cumulative incidence in %. Boxes: interquartile range (IQR; the 25th and 75th percentiles). Whiskers: The upper whisker extends from the hinge to the largest value no further than 1.5 * IQR from the hinge. The lower whisker extends from the hinge to the smallest value at most 1.5 * IQR of the hinge. Data beyond the end of the whiskers are plotted individually by black dots. Horizontal black bar in boxes: Median. N = 836 centre-months. Source data are provided in the 'source_data.xlsx' file.

1.2.19. Figure S19 - Box plot: Psychoactive drug prescriptions (short label: Psych.prescrip.)

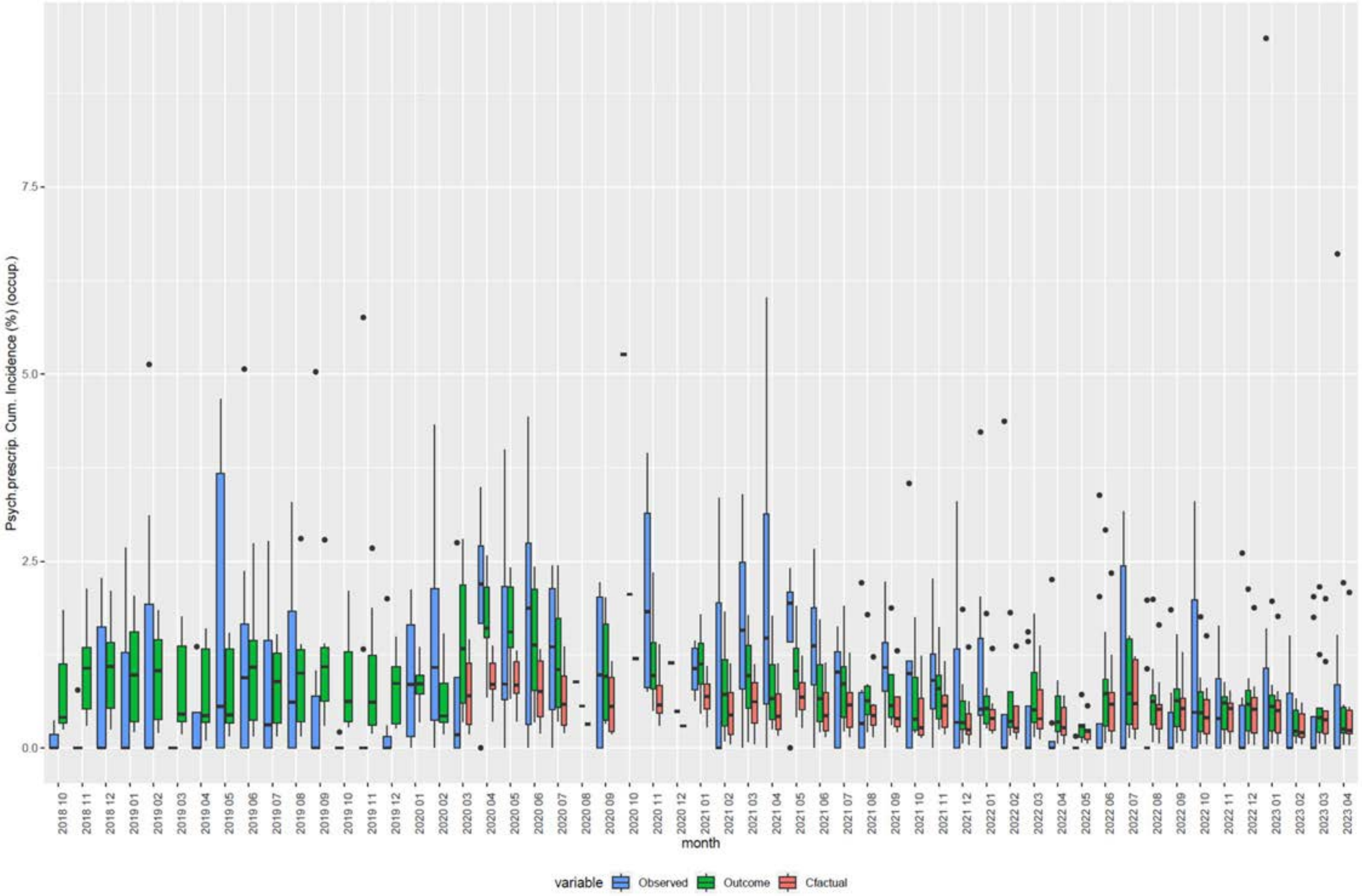

Legend: Observed: observed crude incidence proportions, i.e. cases of individuals with one or more new diagnoses defined by the indicator variable divided by the total number of refugee centre inhabitants (occupancy) and multiplied by 100. Outcome: estimated outcome values based on negative binomial regression models, adjusted for age, sex, centre, and secular trends. Cfactual: estimated counterfactual values based on the negative binomial regression models, adjusted for age, sex, centre and secular trends while setting covid = 0. Y-axis: cumulative incidence in %. Boxes: interquartile range (IQR; the 25th and 75th percentiles). Whiskers: The upper whisker extends from the hinge to the largest value no further than 1.5 * IQR from the hinge. The lower whisker extends from the hinge to the smallest value at most 1.5 * IQR of the hinge. Data beyond the end of the whiskers are plotted individually by black dots. Horizontal black bar in boxes: Median. N = 836 centre-months. Source data are provided in the 'source_data.xlsx' file.

1.2.20. Figure S20 - Box plot: Diseases of the respiratory system (short label: Respiratory syst.)

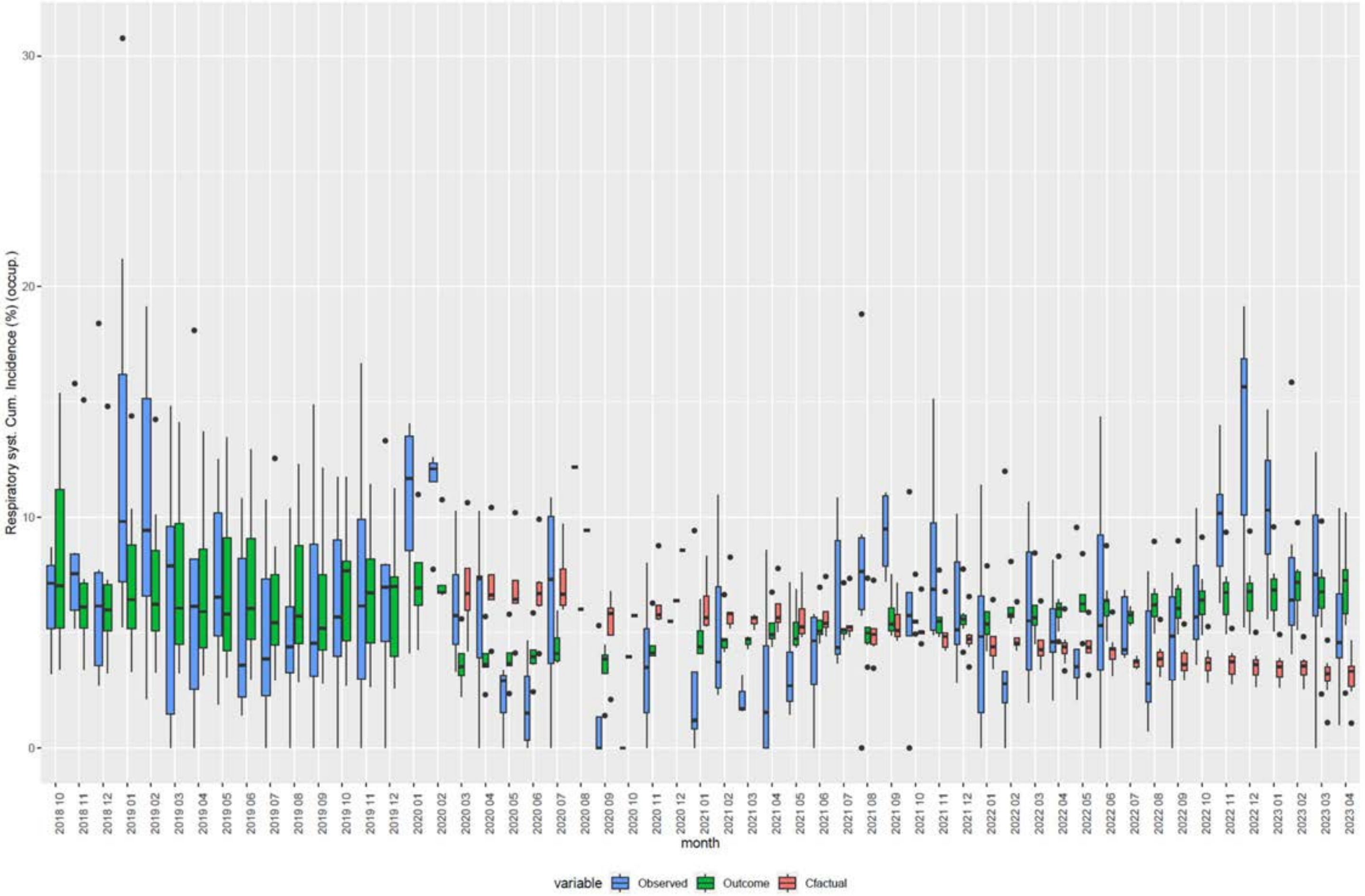

Legend: Observed: observed crude incidence proportions, i.e. cases of individuals with one or more new diagnoses defined by the indicator variable divided by the total number of refugee centre inhabitants (occupancy) and multiplied by 100. Outcome: estimated outcome values based on negative binomial regression models, adjusted for age, sex, centre, and secular trends. Cfactual: estimated counterfactual values based on the negative binomial regression models, adjusted for age, sex, centre and secular trends while setting covid = 0. Y-axis: cumulative incidence in %. Boxes: interquartile range (IQR; the 25th and 75th percentiles). Whiskers: The upper whisker extends from the hinge to the largest value no further than 1.5 * IQR from the hinge. The lower whisker extends from the hinge to the smallest value at most 1.5 * IQR of the hinge. Data beyond the end of the whiskers are plotted individually by black dots. Horizontal black bar in boxes: Median. N = 836 centre-months. Source data are provided in the 'source_data.xlsx' file.

1.2.21. Figure S21 - Box plot: Diseases of the genitourinary system (short label: Genitourinary syst.)

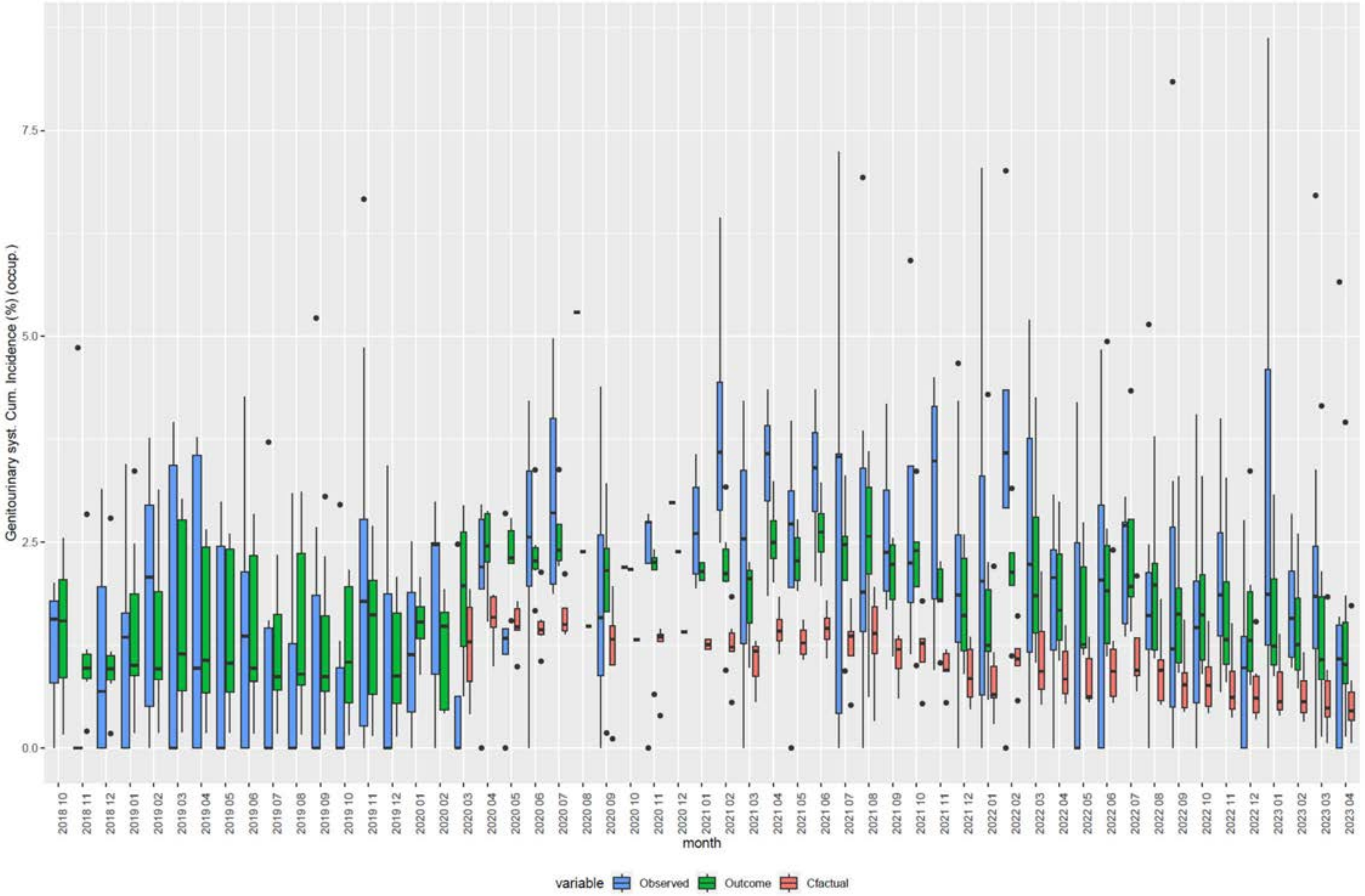

Legend: Observed: observed crude incidence proportions, i.e. cases of individuals with one or more new diagnoses defined by the indicator variable divided by the total number of refugee centre inhabitants (occupancy) and multiplied by 100. Outcome: estimated outcome values based on negative binomial regression models, adjusted for age, sex, centre, and secular trends. Cfactual: estimated counterfactual values based on the negative binomial regression models, adjusted for age, sex, centre and secular trends while setting covid = 0. Y-axis: cumulative incidence in %. Boxes: interquartile range (IQR; the 25th and 75th percentiles). Whiskers: The upper whisker extends from the hinge to the largest value no further than 1.5 * IQR from the hinge. The lower whisker extends from the hinge to the smallest value at most 1.5 * IQR of the hinge. Data beyond the end of the whiskers are plotted individually by black dots. Horizontal black bar in boxes: Median. N = 836 centre-months. Source data are provided in the 'source_data.xlsx' file.

2. Sensitivity analyses 1 – 3

2.1 Detailed results for the sensitivity analysis 1

Sensitivity analysis 1 was performed on subset 1 (see flow chart). Compared to the main analysis, we additionally adjusted the models for country of origin of the patients and used the number of patients as denominator. Therefore, we calculated the percentage of patients belonging to the 5 most frequent countries of origin in the data, respectively. Thus, the models of sensitivity analysis 1 contain the variables `% Nigerian pat., `% Afghani pat., `% Syrian pat., `% Iraqi pat. and `% Turkish pat. in addition to the percentage of adult and male patients as well as immediate pandemic effects (covid), peri-pandemic time trends (postslope) and secular trends (time) as well as a random effect on centre. The description of subset 1 (see additional excel file Table S1) and original R output for each model (see below), are reported. The model for the variable Diseases of the blood and blood-forming organs and certain disorders involving the immune mechanism (short label: Blood) did not converge, therefore, the model is not shown in sensitivity analysis 1.

2.1.1. Disabilities (short label: Disability)

```
Family: nbinom2  ( log )
Formula:
m1[, var] ~ `% adults (pat.)` + `% male (pat.)` + `% Nigerian pat.` +
    `% Afghani pat.` + `% Syrian pat.` + `% Iraqi pat.` + `% Turkish pat.` +
    `Peri-pandemic time trend` + time + `Peri-pandemic Inc.` +
    (1 | centre) + offset(log(patients))
Zero inflation:            ~1
Data: m1

     AIC      BIC   logLik deviance df.resid
  2697.4   2763.5  -1334.7   2669.4      819

Random effects:

Conditional model:
 Groups Name        Variance Std.Dev.
 centre (Intercept) 1.737    1.318
Number of obs: 833, groups:  centre, 25

Dispersion parameter for nbinom2 family (): 13.7

Conditional model:
                            Estimate Std. Error z value Pr(>|z|)
(Intercept)                -6.412487   0.566018 -11.329  < 2e-16 ***
`% adults (pat.)`           0.105993   0.072495   1.462 0.143718
`% male (pat.)`             0.078776   0.055264   1.425 0.154026
`% Nigerian pat.`          -0.922656   0.513490  -1.797 0.072362 .
`% Afghani pat.`           -1.083967   0.282532  -3.837 0.000125 ***
`% Syrian pat.`            -1.772981   0.398966  -4.444 8.83e-06 ***
`% Iraqi pat.`              3.578473   0.904603   3.956 7.63e-05 ***
`% Turkish pat.`           -1.956572   0.604877  -3.235 0.001218 **
`Peri-pandemic time trend` -0.023363   0.009100  -2.567 0.010246 *
time                        0.022687   0.008689   2.611 0.009026 **
`Peri-pandemic Inc.`        0.237468   0.129214   1.838 0.066093 .
---
Signif. codes:  0 '***' 0.001 '**' 0.01 '*' 0.05 '.' 0.1 ' ' 1

Zero-inflation model:
            Estimate Std. Error z value Pr(>|z|)
(Intercept)  -0.6103     0.1200  -5.086 3.65e-07 ***
---
Signif. codes:  0 '***' 0.001 '**' 0.01 '*' 0.05 '.' 0.1 ' ' 1
                                 incident rate ratio 2.5 % 97.5 %
cond.(Intercept)                                0.00  0.00   0.00
cond.`% adults (pat.)`                          1.11  0.96   1.28
cond.`% male (pat.)`                            1.08  0.97   1.21
cond.`% Nigerian pat.`                          0.40  0.15   1.09
cond.`% Afghani pat.`                           0.34  0.19   0.59
cond.`% Syrian pat.`                            0.17  0.08   0.37
cond.`% Iraqi pat.`                            35.82  6.08 210.92
cond.`% Turkish pat.`                           0.14  0.04   0.46
cond.`Peri-pandemic time trend`                 0.98  0.96   0.99
cond.time                                       1.02  1.01   1.04
cond.`Peri-pandemic Inc.`                       1.27  0.98   1.63
zi.(Intercept)                                  0.54  0.43   0.69
cond.Std.Dev.(Intercept)|centre                 3.74  2.47   6.80
```

2.1.2. Injury, poisoning and certain other consequences of external causes (short label: Cons.ext.causes)

```
Family: nbinom2  ( log )
Formula:
m1[, var] ~ `% adults (pat.)` + `% male (pat.)` + `% Nigerian pat.` +
    `% Afghani pat.` + `% Syrian pat.` + `% Iraqi pat.` + `% Turkish pat.` +
    `Peri-pandemic time trend` + time + `Peri-pandemic Inc.` +
    (1 | centre) + offset(log(patients))
Zero inflation:             ~1
Data: m1

     AIC      BIC   logLik deviance df.resid
  4459.6   4525.7  -2215.8   4431.6      819

Random effects:

Conditional model:
 Groups Name        Variance Std.Dev.
 centre (Intercept) 0.2878   0.5364
Number of obs: 833, groups:  centre, 25

Dispersion parameter for nbinom2 family (): 9.61

Conditional model:
                            Estimate Std. Error z value Pr(>|z|)
(Intercept)                -3.615890   0.344464 -10.497  < 2e-16 ***
`% adults (pat.)`           0.053797   0.044584   1.207 0.227574
`% male (pat.)`             0.066124   0.032885   2.011 0.044354 *
`% Nigerian pat.`           0.067896   0.257240   0.264 0.791826
`% Afghani pat.`           -0.523046   0.189710  -2.757 0.005832 **
`% Syrian pat.`            -0.941450   0.262154  -3.591 0.000329 ***
`% Iraqi pat.`             -0.324862   0.642417  -0.506 0.613076
`% Turkish pat.`           -0.314200   0.348141  -0.903 0.366789
`Peri-pandemic time trend`  0.000331   0.005896   0.056 0.955232
time                       -0.009304   0.005473  -1.700 0.089104 .
`Peri-pandemic Inc.`        0.286908   0.087463   3.280 0.001037 **
---
Signif. codes:  0 '***' 0.001 '**' 0.01 '*' 0.05 '.' 0.1 ' ' 1

Zero-inflation model:
            Estimate Std. Error z value Pr(>|z|)
(Intercept)  -1.4800     0.1285  -11.51   <2e-16 ***
---
Signif. codes:  0 '***' 0.001 '**' 0.01 '*' 0.05 '.' 0.1 ' ' 1
                                incident rate ratio 2.5 % 97.5 %
cond.(Intercept)                               0.03  0.01   0.05
cond.`% adults (pat.)`                         1.06  0.97   1.15
cond.`% male (pat.)`                           1.07  1.00   1.14
cond.`% Nigerian pat.`                         1.07  0.65   1.77
cond.`% Afghani pat.`                          0.59  0.41   0.86
cond.`% Syrian pat.`                           0.39  0.23   0.65
cond.`% Iraqi pat.`                            0.72  0.21   2.55
cond.`% Turkish pat.`                          0.73  0.37   1.45
cond.`Peri-pandemic time trend`                1.00  0.99   1.01
cond.time                                      0.99  0.98   1.00
cond.`Peri-pandemic Inc.`                      1.33  1.12   1.58
zi.(Intercept)                                 0.23  0.18   0.29
cond.Std.Dev.(Intercept)|centre                1.71  1.47   2.10
```

2.1.3. Diseases of the skin and subcutaneous tissue (short label: Skin)

```
Family: nbinom2  ( log )
Formula:
m1[, var] ~ `% adults (pat.)` + `% male (pat.)` + `% Nigerian pat.` +
    `% Afghani pat.` + `% Syrian pat.` + `% Iraqi pat.` + `% Turkish pat.` +
    `Peri-pandemic time trend` + time + `Peri-pandemic Inc.` +
    (1 | centre) + offset(log(patients))
Zero inflation:             ~1
Data: m1

     AIC      BIC   logLik deviance df.resid
  4504.9   4571.1  -2238.5   4476.9      819

Random effects:

Conditional model:
 Groups Name        Variance Std.Dev.
 centre (Intercept) 0.1662   0.4077
Number of obs: 833, groups:  centre, 25

Dispersion parameter for nbinom2 family (): 20.5

Conditional model:
                            Estimate Std. Error z value Pr(>|z|)
(Intercept)                -4.015472   0.273899 -14.660  < 2e-16 ***
`% adults (pat.)`           0.039547   0.036261   1.091 0.275436
`% male (pat.)`             0.066737   0.026184   2.549 0.010810 *
`% Nigerian pat.`           0.586952   0.226975   2.586 0.009710 **
`% Afghani pat.`            0.481466   0.145977   3.298 0.000973 ***
`% Syrian pat.`             0.210206   0.203923   1.031 0.302628
`% Iraqi pat.`             -0.353865   0.529557  -0.668 0.503987
`% Turkish pat.`            0.205358   0.249819   0.822 0.411060
`Peri-pandemic time trend` -0.014818   0.004900  -3.024 0.002495 **
time                        0.010667   0.004654   2.292 0.021920 *
`Peri-pandemic Inc.`       -0.094438   0.068051  -1.388 0.165217
---
Signif. codes:  0 '***' 0.001 '**' 0.01 '*' 0.05 '.' 0.1 ' ' 1

Zero-inflation model:
            Estimate Std. Error z value Pr(>|z|)
(Intercept)  -1.5413     0.1124  -13.71   <2e-16 ***
---
Signif. codes:  0 '***' 0.001 '**' 0.01 '*' 0.05 '.' 0.1 ' ' 1
                                incident rate ratio 2.5 % 97.5 %
cond.(Intercept)                               0.02  0.01   0.03
cond.`% adults (pat.)`                         1.04  0.97   1.12
cond.`% male (pat.)`                           1.07  1.02   1.13
cond.`% Nigerian pat.`                         1.80  1.15   2.81
cond.`% Afghani pat.`                          1.62  1.22   2.15
cond.`% Syrian pat.`                           1.23  0.83   1.84
cond.`% Iraqi pat.`                            0.70  0.25   1.98
cond.`% Turkish pat.`                          1.23  0.75   2.00
cond.`Peri-pandemic time trend`                0.99  0.98   0.99
cond.time                                      1.01  1.00   1.02
cond.`Peri-pandemic Inc.`                      0.91  0.80   1.04
zi.(Intercept)                                 0.21  0.17   0.27
cond.Std.Dev.(Intercept)|centre                1.50  1.35   1.75
```

2.1.4. Diseases of the digestive system (short label: Digestive syst.)

```
Family: nbinom2  ( log )
Formula:
m1[, var] ~ `% adults (pat.)` + `% male (pat.)` + `% Nigerian pat.` +
    `% Afghani pat.` + `% Syrian pat.` + `% Iraqi pat.` + `% Turkish pat.` +
    `Peri-pandemic time trend` + time + `Peri-pandemic Inc.` +
    (1 | centre) + offset(log(patients))
Zero inflation:             ~1
Data: m1

     AIC      BIC   logLik deviance df.resid
  5454.1   5520.3  -2713.1   5426.1      819

Random effects:

Conditional model:
 Groups Name        Variance Std.Dev.
 centre (Intercept) 0.3353   0.579
Number of obs: 833, groups:  centre, 25

Dispersion parameter for nbinom2 family (): 14.2

Conditional model:
                            Estimate Std. Error z value Pr(>|z|)
(Intercept)                -2.818665   0.255107 -11.049  < 2e-16 ***
`% adults (pat.)`           0.057062   0.029961   1.905 0.056839 .
`% male (pat.)`             0.027639   0.021341   1.295 0.195282
`% Nigerian pat.`          -0.410869   0.190870  -2.153 0.031349 *
`% Afghani pat.`           -0.315658   0.132208  -2.388 0.016959 *
`% Syrian pat.`            -0.361260   0.186012  -1.942 0.052120 .
`% Iraqi pat.`              0.911605   0.463162   1.968 0.049043 *
`% Turkish pat.`           -0.858419   0.230888  -3.718 0.000201 ***
`Peri-pandemic time trend`  0.001786   0.004513   0.396 0.692279
time                       -0.002184   0.004277  -0.511 0.609527
`Peri-pandemic Inc.`       -0.025370   0.062159  -0.408 0.683169
---
Signif. codes:  0 '***' 0.001 '**' 0.01 '*' 0.05 '.' 0.1 ' ' 1

Zero-inflation model:
            Estimate Std. Error z value Pr(>|z|)
(Intercept)  -3.1396     0.2309   -13.6   <2e-16 ***
---
Signif. codes:  0 '***' 0.001 '**' 0.01 '*' 0.05 '.' 0.1 ' ' 1
                                incident rate ratio 2.5 % 97.5 %
cond.(Intercept)                               0.06  0.04   0.10
cond.`% adults (pat.)`                         1.06  1.00   1.12
cond.`% male (pat.)`                           1.03  0.99   1.07
cond.`% Nigerian pat.`                         0.66  0.46   0.96
cond.`% Afghani pat.`                          0.73  0.56   0.95
cond.`% Syrian pat.`                           0.70  0.48   1.00
cond.`% Iraqi pat.`                            2.49  1.00   6.17
cond.`% Turkish pat.`                          0.42  0.27   0.67
cond.`Peri-pandemic time trend`                1.00  0.99   1.01
cond.time                                      1.00  0.99   1.01
cond.`Peri-pandemic Inc.`                      0.97  0.86   1.10
zi.(Intercept)                                 0.04  0.03   0.07
cond.Std.Dev.(Intercept)|centre                1.78  1.54   2.16
```

2.1.5. Certain infectious and parasitic diseases (short label: Inf.diseases)

```
Family: nbinom2  ( log )
Formula:
m1[, var] ~ `% adults (pat.)` + `% male (pat.)` + `% Nigerian pat.` +
    `% Afghani pat.` + `% Syrian pat.` + `% Iraqi pat.` + `% Turkish pat.` +
    `Peri-pandemic time trend` + time + `Peri-pandemic Inc.` +
    (1 | centre) + offset(log(patients))
Zero inflation:             ~1
Data: m1

     AIC      BIC   logLik deviance df.resid
  5515.9   5582.0  -2743.9   5487.9      819

Random effects:

Conditional model:
 Groups Name        Variance Std.Dev.
 centre (Intercept) 0.2477   0.4977
Number of obs: 833, groups:  centre, 25

Dispersion parameter for nbinom2 family (): 9.78

Conditional model:
                            Estimate Std. Error z value Pr(>|z|)
(Intercept)                -2.550152   0.276179  -9.234  < 2e-16 ***
`% adults (pat.)`          -0.044701   0.034794  -1.285 0.198887
`% male (pat.)`             0.092458   0.026083   3.545 0.000393 ***
`% Nigerian pat.`           0.099690   0.229338   0.435 0.663791
`% Afghani pat.`           -0.012910   0.155899  -0.083 0.934001
`% Syrian pat.`             0.809492   0.226545   3.573 0.000353 ***
`% Iraqi pat.`             -0.801375   0.548479  -1.461 0.143992
`% Turkish pat.`            0.020525   0.270403   0.076 0.939495
`Peri-pandemic time trend`  0.016827   0.005117   3.288 0.001009 **
time                       -0.005378   0.004822  -1.115 0.264699
`Peri-pandemic Inc.`       -0.338650   0.073687  -4.596 4.31e-06 ***
---
Signif. codes:  0 '***' 0.001 '**' 0.01 '*' 0.05 '.' 0.1 ' ' 1

Zero-inflation model:
            Estimate Std. Error z value Pr(>|z|)
(Intercept)  -2.2197     0.1507  -14.73   <2e-16 ***
---
Signif. codes:  0 '***' 0.001 '**' 0.01 '*' 0.05 '.' 0.1 ' ' 1
                                incident rate ratio 2.5 % 97.5 %
cond.(Intercept)                               0.08  0.05   0.13
cond.`% adults (pat.)`                         0.96  0.89   1.02
cond.`% male (pat.)`                           1.10  1.04   1.15
cond.`% Nigerian pat.`                         1.10  0.70   1.73
cond.`% Afghani pat.`                          0.99  0.73   1.34
cond.`% Syrian pat.`                           2.25  1.44   3.50
cond.`% Iraqi pat.`                            0.45  0.15   1.31
cond.`% Turkish pat.`                          1.02  0.60   1.73
cond.`Peri-pandemic time trend`                1.02  1.01   1.03
cond.time                                      0.99  0.99   1.00
cond.`Peri-pandemic Inc.`                      0.71  0.62   0.82
zi.(Intercept)                                 0.11  0.08   0.15
cond.Std.Dev.(Intercept)|centre                1.64  1.44   1.97
```

2.1.6. Notifiable infectious diseases (short label: Inf.notify)

```
Family: nbinom2  ( log )
Formula:
m1[, var] ~ `% adults (pat.)` + `% male (pat.)` + `% Nigerian pat.` +
    `% Afghani pat.` + `% Syrian pat.` + `% Iraqi pat.` + `% Turkish pat.` +
    `Peri-pandemic time trend` + time + `Peri-pandemic Inc.` +
    (1 | centre) + offset(log(patients))
Zero inflation:             ~1
Data: m1

     AIC      BIC   logLik deviance df.resid
  2355.6   2421.8  -1163.8   2327.6      819

Random effects:

Conditional model:
 Groups Name        Variance Std.Dev.
 centre (Intercept) 0.9691   0.9844
Number of obs: 833, groups:  centre, 25

Dispersion parameter for nbinom2 family (): 3.52

Conditional model:
                            Estimate Std. Error z value Pr(>|z|)
(Intercept)                -2.559594   0.784803  -3.261  0.00111 **
`% adults (pat.)`          -0.190225   0.114619  -1.660  0.09699 .
`% male (pat.)`            -0.065861   0.083196  -0.792  0.42858
`% Nigerian pat.`          -0.271223   0.684103  -0.396  0.69176
`% Afghani pat.`           -0.210921   0.483357  -0.436  0.66257
`% Syrian pat.`            -1.629448   0.658605  -2.474  0.01336 *
`% Iraqi pat.`              4.214885   1.518073   2.776  0.00550 **
`% Turkish pat.`            0.055686   0.878380   0.063  0.94945
`Peri-pandemic time trend`  0.014634   0.011965   1.223  0.22132
time                       -0.006167   0.011250  -0.548  0.58357
`Peri-pandemic Inc.`       -0.467751   0.214648  -2.179  0.02932 *
---
Signif. codes:  0 '***' 0.001 '**' 0.01 '*' 0.05 '.' 0.1 ' ' 1

Zero-inflation model:
            Estimate Std. Error z value Pr(>|z|)
(Intercept)  -0.2508     0.1594  -1.573    0.116
                                incident rate ratio 2.5 %  97.5 %
cond.(Intercept)                               0.08  0.02    0.36
cond.`% adults (pat.)`                         0.83  0.66    1.04
cond.`% male (pat.)`                           0.94  0.80    1.10
cond.`% Nigerian pat.`                         0.76  0.20    2.91
cond.`% Afghani pat.`                          0.81  0.31    2.09
cond.`% Syrian pat.`                           0.20  0.05    0.71
cond.`% Iraqi pat.`                           67.69  3.45 1326.44
cond.`% Turkish pat.`                          1.06  0.19    5.91
cond.`Peri-pandemic time trend`                1.01  0.99    1.04
cond.time                                      0.99  0.97    1.02
cond.`Peri-pandemic Inc.`                      0.63  0.41    0.95
zi.(Intercept)                                 0.78  0.57    1.06
cond.Std.Dev.(Intercept)|centre                2.68  1.96    4.24
```

2.1.7. Diseases of the circulatory system (short label: Circulatory syst.)

```
Family: nbinom2  ( log )
Formula:
m1[, var] ~ `% adults (pat.)` + `% male (pat.)` + `% Nigerian pat.` +
    `% Afghani pat.` + `% Syrian pat.` + `% Iraqi pat.` + `% Turkish pat.` +
    `Peri-pandemic time trend` + time + `Peri-pandemic Inc.` +
    (1 | centre) + offset(log(patients))
Zero inflation:             ~1
Data: m1

     AIC      BIC   logLik deviance df.resid
  3928.8   3994.9  -1950.4   3900.8      819

Random effects:

Conditional model:
 Groups Name        Variance Std.Dev.
 centre (Intercept) 0.3293   0.5739
Number of obs: 833, groups:  centre, 25

Dispersion parameter for nbinom2 family (): 22.4

Conditional model:
                            Estimate Std. Error z value Pr(>|z|)
(Intercept)                -2.961464   0.329387  -8.991  < 2e-16 ***
`% adults (pat.)`           0.040254   0.042516   0.947  0.34374
`% male (pat.)`            -0.077415   0.030121  -2.570  0.01017 *
`% Nigerian pat.`          -0.677167   0.307027  -2.206  0.02741 *
`% Afghani pat.`           -1.254071   0.176060  -7.123 1.06e-12 ***
`% Syrian pat.`            -1.621604   0.259446  -6.250 4.10e-10 ***
`% Iraqi pat.`             -0.323469   0.620006  -0.522  0.60187
`% Turkish pat.`           -1.171569   0.408216  -2.870  0.00411 **
`Peri-pandemic time trend` -0.006053   0.005436  -1.114  0.26544
time                        0.004882   0.005227   0.934  0.35029
`Peri-pandemic Inc.`        0.053721   0.077283   0.695  0.48698
---
Signif. codes:  0 '***' 0.001 '**' 0.01 '*' 0.05 '.' 0.1 ' ' 1

Zero-inflation model:
            Estimate Std. Error z value Pr(>|z|)
(Intercept)   -1.364      0.124     -11   <2e-16 ***
---
Signif. codes:  0 '***' 0.001 '**' 0.01 '*' 0.05 '.' 0.1 ' ' 1
                                incident rate ratio 2.5 % 97.5 %
cond.(Intercept)                               0.05  0.03   0.10
cond.`% adults (pat.)`                         1.04  0.96   1.13
cond.`% male (pat.)`                           0.93  0.87   0.98
cond.`% Nigerian pat.`                         0.51  0.28   0.93
cond.`% Afghani pat.`                          0.29  0.20   0.40
cond.`% Syrian pat.`                           0.20  0.12   0.33
cond.`% Iraqi pat.`                            0.72  0.21   2.44
cond.`% Turkish pat.`                          0.31  0.14   0.69
cond.`Peri-pandemic time trend`                0.99  0.98   1.00
cond.time                                      1.00  0.99   1.02
cond.`Peri-pandemic Inc.`                      1.06  0.91   1.23
zi.(Intercept)                                 0.26  0.20   0.33
cond.Std.Dev.(Intercept)|centre                1.78  1.49   2.28
```

2.1.8. Hypertension (short label: Hypertension)

```
Family: nbinom2  ( log )
Formula:
m1[, var] ~ `% adults (pat.)` + `% male (pat.)` + `% Nigerian pat.` +
    `% Afghani pat.` + `% Syrian pat.` + `% Iraqi pat.` + `% Turkish pat.` +
    `Peri-pandemic time trend` + time + `Peri-pandemic Inc.` +
    (1 | centre) + offset(log(patients))
Zero inflation:             ~1
Data: m1

     AIC      BIC   logLik deviance df.resid
  3137.6   3203.8  -1554.8   3109.6      819

Random effects:

Conditional model:
 Groups Name        Variance Std.Dev.
 centre (Intercept) 0.9299   0.9643
Number of obs: 833, groups:  centre, 25

Dispersion parameter for nbinom2 family (): 10.5

Conditional model:
                            Estimate Std. Error z value Pr(>|z|)
(Intercept)                -4.068551   0.504728  -8.061 7.57e-16 ***
`% adults (pat.)`           0.089876   0.065343   1.375  0.16899
`% male (pat.)`            -0.029547   0.042734  -0.691  0.48931
`% Nigerian pat.`          -0.983700   0.446375  -2.204  0.02754 *
`% Afghani pat.`           -1.528921   0.273341  -5.593 2.23e-08 ***
`% Syrian pat.`            -2.509935   0.393602  -6.377 1.81e-10 ***
`% Iraqi pat.`             -2.703359   0.881951  -3.065  0.00218 **
`% Turkish pat.`           -2.748211   0.695610  -3.951 7.79e-05 ***
`Peri-pandemic time trend`  0.001312   0.008385   0.156  0.87567
time                        0.006443   0.007903   0.815  0.41493
`Peri-pandemic Inc.`       -0.087981   0.117355  -0.750  0.45343
---
Signif. codes:  0 '***' 0.001 '**' 0.01 '*' 0.05 '.' 0.1 ' ' 1

Zero-inflation model:
            Estimate Std. Error z value Pr(>|z|)
(Intercept)  -0.6491     0.1091  -5.952 2.65e-09 ***
---
Signif. codes:  0 '***' 0.001 '**' 0.01 '*' 0.05 '.' 0.1 ' ' 1
                                incident rate ratio 2.5 % 97.5 %
cond.(Intercept)                               0.02  0.01   0.05
cond.`% adults (pat.)`                         1.09  0.96   1.24
cond.`% male (pat.)`                           0.97  0.89   1.06
cond.`% Nigerian pat.`                         0.37  0.16   0.90
cond.`% Afghani pat.`                          0.22  0.13   0.37
cond.`% Syrian pat.`                           0.08  0.04   0.18
cond.`% Iraqi pat.`                            0.07  0.01   0.38
cond.`% Turkish pat.`                          0.06  0.02   0.25
cond.`Peri-pandemic time trend`                1.00  0.98   1.02
cond.time                                      1.01  0.99   1.02
cond.`Peri-pandemic Inc.`                      0.92  0.73   1.15
zi.(Intercept)                                 0.52  0.42   0.65
cond.Std.Dev.(Intercept)|centre                2.62  1.96   3.97
```

2.1.9. Endocrine, nutritional and metabolic diseases (short label: Metabolic)

```
Family: nbinom2  ( log )
Formula:
m1[, var] ~ `% adults (pat.)` + `% male (pat.)` + `% Nigerian pat.` +
    `% Afghani pat.` + `% Syrian pat.` + `% Iraqi pat.` + `% Turkish pat.` +
    `Peri-pandemic time trend` + time + `Peri-pandemic Inc.` +
    (1 | centre) + offset(log(patients))
Zero inflation:            ~1
Data: m1

     AIC      BIC   logLik deviance df.resid
  3604.5   3670.6  -1788.2   3576.5      819

Random effects:

Conditional model:
 Groups Name        Variance Std.Dev.
 centre (Intercept) 0.8274   0.9096
Number of obs: 833, groups:  centre, 25

Dispersion parameter for nbinom2 family (): 23.3

Conditional model:
                            Estimate Std. Error z value Pr(>|z|)
(Intercept)                -3.093600   0.392975  -7.872 3.48e-15 ***
`% adults (pat.)`           0.053937   0.045996   1.173 0.240937
`% male (pat.)`            -0.164372   0.034220  -4.803 1.56e-06 ***
`% Nigerian pat.`          -1.372154   0.325264  -4.219 2.46e-05 ***
`% Afghani pat.`           -0.695231   0.185913  -3.740 0.000184 ***
`% Syrian pat.`            -0.119284   0.294989  -0.404 0.685941
`% Iraqi pat.`             -0.595675   0.676921  -0.880 0.378872
`% Turkish pat.`           -1.249784   0.379511  -3.293 0.000991 ***
`Peri-pandemic time trend` -0.011970   0.005948  -2.013 0.044166 *
time                        0.010021   0.005659   1.771 0.076575 .
`Peri-pandemic Inc.`       -0.051856   0.083837  -0.619 0.536222
---
Signif. codes:  0 '***' 0.001 '**' 0.01 '*' 0.05 '.' 0.1 ' ' 1

Zero-inflation model:
            Estimate Std. Error z value Pr(>|z|)
(Intercept)  -1.2150     0.1139  -10.66   <2e-16 ***
---
Signif. codes:  0 '***' 0.001 '**' 0.01 '*' 0.05 '.' 0.1 ' ' 1
                                incident rate ratio 2.5 % 97.5 %
cond.(Intercept)                               0.05  0.02   0.10
cond.`% adults (pat.)`                         1.06  0.96   1.15
cond.`% male (pat.)`                           0.85  0.79   0.91
cond.`% Nigerian pat.`                         0.25  0.13   0.48
cond.`% Afghani pat.`                          0.50  0.35   0.72
cond.`% Syrian pat.`                           0.89  0.50   1.58
cond.`% Iraqi pat.`                            0.55  0.15   2.08
cond.`% Turkish pat.`                          0.29  0.14   0.60
cond.`Peri-pandemic time trend`                0.99  0.98   1.00
cond.time                                      1.01  1.00   1.02
cond.`Peri-pandemic Inc.`                      0.95  0.81   1.12
zi.(Intercept)                                 0.30  0.24   0.37
cond.Std.Dev.(Intercept)|centre                2.48  1.87   3.73
```

2.1.10. Diabetes mellitus (short label: Diabetes)

```
Family: nbinom2  ( log )
Formula:
m1[, var] ~ `% adults (pat.)` + `% male (pat.)` + `% Nigerian pat.` +
    `% Afghani pat.` + `% Syrian pat.` + `% Iraqi pat.` + `% Turkish pat.` +
    `Peri-pandemic time trend` + time + `Peri-pandemic Inc.` +
    (1 | centre) + offset(log(patients))
Zero inflation:             ~1
Data: m1

     AIC      BIC   logLik deviance df.resid
  2494.4   2560.5  -1233.2   2466.4      819

Random effects:

Conditional model:
 Groups Name        Variance Std.Dev.
 centre (Intercept) 1.659    1.288
Number of obs: 833, groups:  centre, 25

Dispersion parameter for nbinom2 family ():  141

Conditional model:
                            Estimate Std. Error z value Pr(>|z|)
(Intercept)                -5.248444   0.547586  -9.585  < 2e-16 ***
`% adults (pat.)`           0.217509   0.064102   3.393 0.000691 ***
`% male (pat.)`            -0.172059   0.044663  -3.852 0.000117 ***
`% Nigerian pat.`          -1.349833   0.465169  -2.902 0.003710 **
`% Afghani pat.`           -1.282341   0.249877  -5.132 2.87e-07 ***
`% Syrian pat.`            -0.191228   0.391739  -0.488 0.625443
`% Iraqi pat.`             -0.650963   0.871928  -0.747 0.455318
`% Turkish pat.`           -1.019271   0.573273  -1.778 0.075406 .
`Peri-pandemic time trend`  0.001841   0.007849   0.235 0.814537
time                        0.005976   0.007711   0.775 0.438358
`Peri-pandemic Inc.`       -0.071151   0.102025  -0.697 0.485559
---
Signif. codes:  0 '***' 0.001 '**' 0.01 '*' 0.05 '.' 0.1 ' ' 1

Zero-inflation model:
            Estimate Std. Error z value Pr(>|z|)
(Intercept)  -0.6165     0.1174  -5.252 1.51e-07 ***
---
Signif. codes:  0 '***' 0.001 '**' 0.01 '*' 0.05 '.' 0.1 ' ' 1
                                incident rate ratio 2.5 % 97.5 %
cond.(Intercept)                              0.01  0.00   0.02
cond.`% adults (pat.)`                        1.24  1.10   1.41
cond.`% male (pat.)`                          0.84  0.77   0.92
cond.`% Nigerian pat.`                        0.26  0.10   0.65
cond.`% Afghani pat.`                         0.28  0.17   0.45
cond.`% Syrian pat.`                          0.83  0.38   1.78
cond.`% Iraqi pat.`                           0.52  0.09   2.88
cond.`% Turkish pat.`                         0.36  0.12   1.11
cond.`Peri-pandemic time trend`               1.00  0.99   1.02
cond.time                                     1.01  0.99   1.02
cond.`Peri-pandemic Inc.`                     0.93  0.76   1.14
zi.(Intercept)                                0.54  0.43   0.68
cond.Std.Dev.(Intercept)|centre               3.63  2.38   6.78
```

2.1.11. Diseases of the musculoskeletal system and connective tissue (short label: Musculoskelet. syst.)

```
Family: nbinom2  ( log )
Formula:
m1[, var] ~ `% adults (pat.)` + `% male (pat.)` + `% Nigerian pat.` +
    `% Afghani pat.` + `% Syrian pat.` + `% Iraqi pat.` + `% Turkish pat.` +
    `Peri-pandemic time trend` + time + `Peri-pandemic Inc.` +
    (1 | centre) + offset(log(patients))
Zero inflation:            ~1
Data: m1

     AIC      BIC   logLik deviance df.resid
  4871.1   4937.2  -2421.5   4843.1      819

Random effects:

Conditional model:
 Groups Name        Variance Std.Dev.
 centre (Intercept) 0.1776   0.4214
Number of obs: 833, groups:  centre, 25

Dispersion parameter for nbinom2 family (): 21.2

Conditional model:
                            Estimate Std. Error z value Pr(>|z|)
(Intercept)                -3.864077   0.243388 -15.876  < 2e-16 ***
`% adults (pat.)`           0.147491   0.030207   4.883 1.05e-06 ***
`% male (pat.)`            -0.011759   0.022374  -0.526  0.59919
`% Nigerian pat.`           0.351551   0.186657   1.883  0.05965 .
`% Afghani pat.`           -0.163994   0.134959  -1.215  0.22431
`% Syrian pat.`            -0.635539   0.185675  -3.423  0.00062 ***
`% Iraqi pat.`              0.244921   0.473522   0.517  0.60499
`% Turkish pat.`           -0.658439   0.244085  -2.698  0.00698 **
`Peri-pandemic time trend` -0.020429   0.004358  -4.688 2.76e-06 ***
time                        0.009249   0.004084   2.265  0.02354 *
`Peri-pandemic Inc.`        0.065582   0.060267   1.088  0.27651
---
Signif. codes:  0 '***' 0.001 '**' 0.01 '*' 0.05 '.' 0.1 ' ' 1

Zero-inflation model:
            Estimate Std. Error z value Pr(>|z|)
(Intercept)  -2.0566     0.1476  -13.93   <2e-16 ***
---
Signif. codes:  0 '***' 0.001 '**' 0.01 '*' 0.05 '.' 0.1 ' ' 1
                                incident rate ratio 2.5 % 97.5 %
cond.(Intercept)                               0.02  0.01   0.03
cond.`% adults (pat.)`                         1.16  1.09   1.23
cond.`% male (pat.)`                           0.99  0.95   1.03
cond.`% Nigerian pat.`                         1.42  0.99   2.05
cond.`% Afghani pat.`                          0.85  0.65   1.11
cond.`% Syrian pat.`                           0.53  0.37   0.76
cond.`% Iraqi pat.`                            1.28  0.51   3.23
cond.`% Turkish pat.`                          0.52  0.32   0.84
cond.`Peri-pandemic time trend`                0.98  0.97   0.99
cond.time                                      1.01  1.00   1.02
cond.`Peri-pandemic Inc.`                      1.07  0.95   1.20
zi.(Intercept)                                 0.13  0.10   0.17
cond.Std.Dev.(Intercept)|centre                1.52  1.36   1.79
```

2.1.12. Neoplasms (short label: Neoplasm)

```
Family: nbinom2  ( log )
Formula:
m1[, var] ~ `% adults (pat.)` + `% male (pat.)` + `% Nigerian pat.` +
    `% Afghani pat.` + `% Syrian pat.` + `% Iraqi pat.` + `% Turkish pat.` +
    `Peri-pandemic time trend` + time + `Peri-pandemic Inc.` +
    (1 | centre) + offset(log(patients))
Zero inflation:             ~1
Data: m1

     AIC      BIC   logLik deviance df.resid
  1903.8   1970.0   -937.9   1875.8      819

Random effects:

Conditional model:
 Groups Name        Variance Std.Dev.
 centre (Intercept) 3.054    1.748
Number of obs: 833, groups:  centre, 25

Dispersion parameter for nbinom2 family (): 18.2

Conditional model:
                            Estimate Std. Error z value Pr(>|z|)
(Intercept)                -6.188907   0.756885  -8.177 2.91e-16 ***
`% adults (pat.)`           0.143355   0.090637   1.582  0.11373
`% male (pat.)`            -0.160212   0.069444  -2.307  0.02105 *
`% Nigerian pat.`          -0.805618   0.541449  -1.488  0.13678
`% Afghani pat.`           -1.335414   0.331247  -4.031 5.54e-05 ***
`% Syrian pat.`            -1.749677   0.583663  -2.998  0.00272 **
`% Iraqi pat.`             -1.179324   1.310520  -0.900  0.36818
`% Turkish pat.`           -3.671272   1.162872  -3.157  0.00159 **
`Peri-pandemic time trend` -0.026288   0.009150  -2.873  0.00407 **
time                        0.023456   0.008388   2.796  0.00517 **
`Peri-pandemic Inc.`       -0.037677   0.134096  -0.281  0.77873
---
Signif. codes:  0 '***' 0.001 '**' 0.01 '*' 0.05 '.' 0.1 ' ' 1

Zero-inflation model:
            Estimate Std. Error z value Pr(>|z|)
(Intercept)  -0.7157     0.1517  -4.718 2.39e-06 ***
---
Signif. codes:  0 '***' 0.001 '**' 0.01 '*' 0.05 '.' 0.1 ' ' 1
                                incident rate ratio 2.5 % 97.5 %
cond.(Intercept)                               0.00  0.00   0.01
cond.`% adults (pat.)`                         1.15  0.97   1.38
cond.`% male (pat.)`                           0.85  0.74   0.98
cond.`% Nigerian pat.`                         0.45  0.15   1.29
cond.`% Afghani pat.`                          0.26  0.14   0.50
cond.`% Syrian pat.`                           0.17  0.06   0.55
cond.`% Iraqi pat.`                            0.31  0.02   4.01
cond.`% Turkish pat.`                          0.03  0.00   0.25
cond.`Peri-pandemic time trend`                0.97  0.96   0.99
cond.time                                      1.02  1.01   1.04
cond.`Peri-pandemic Inc.`                      0.96  0.74   1.25
zi.(Intercept)                                 0.49  0.36   0.66
cond.Std.Dev.(Intercept)|centre                5.74  3.19  13.96
```

2.1.13. Diseases of the nervous system (short label: Nervous syst.)

```
Family: nbinom2  ( log )
Formula:
m1[, var] ~ `% adults (pat.)` + `% male (pat.)` + `% Nigerian pat.` +
    `% Afghani pat.` + `% Syrian pat.` + `% Iraqi pat.` + `% Turkish pat.` +
    `Peri-pandemic time trend` + time + `Peri-pandemic Inc.` +
    (1 | centre) + offset(log(patients))
Zero inflation:             ~1
Data: m1

     AIC      BIC   logLik deviance df.resid
  3878.0   3944.2  -1925.0   3850.0      819

Random effects:

Conditional model:
 Groups Name        Variance Std.Dev.
 centre (Intercept) 0.4019   0.6339
Number of obs: 833, groups:  centre, 25

Dispersion parameter for nbinom2 family (): 10.7

Conditional model:
                            Estimate Std. Error z value Pr(>|z|)
(Intercept)                -3.076537   0.392796  -7.832 4.79e-15 ***
`% adults (pat.)`           0.102921   0.051401   2.002  0.04525 *
`% male (pat.)`            -0.092772   0.039508  -2.348  0.01886 *
`% Nigerian pat.`          -0.687900   0.295840  -2.325  0.02006 *
`% Afghani pat.`           -0.303570   0.207763  -1.461  0.14398
`% Syrian pat.`            -0.901624   0.311444  -2.895  0.00379 **
`% Iraqi pat.`             -0.064429   0.733157  -0.088  0.92997
`% Turkish pat.`           -0.942839   0.400307  -2.355  0.01851 *
`Peri-pandemic time trend`  0.009527   0.006273   1.519  0.12879
time                       -0.018504   0.005787  -3.198  0.00139 **
`Peri-pandemic Inc.`        0.064904   0.091177   0.712  0.47656
---
Signif. codes:  0 '***' 0.001 '**' 0.01 '*' 0.05 '.' 0.1 ' ' 1

Zero-inflation model:
            Estimate Std. Error z value Pr(>|z|)
(Intercept)  -1.1847     0.1148  -10.32   <2e-16 ***
---
Signif. codes:  0 '***' 0.001 '**' 0.01 '*' 0.05 '.' 0.1 ' ' 1
                                incident rate ratio 2.5 % 97.5 %
cond.(Intercept)                               0.05  0.02   0.10
cond.`% adults (pat.)`                         1.11  1.00   1.23
cond.`% male (pat.)`                           0.91  0.84   0.98
cond.`% Nigerian pat.`                         0.50  0.28   0.90
cond.`% Afghani pat.`                          0.74  0.49   1.11
cond.`% Syrian pat.`                           0.41  0.22   0.75
cond.`% Iraqi pat.`                            0.94  0.22   3.95
cond.`% Turkish pat.`                          0.39  0.18   0.85
cond.`Peri-pandemic time trend`                1.01  1.00   1.02
cond.time                                      0.98  0.97   0.99
cond.`Peri-pandemic Inc.`                      1.07  0.89   1.28
zi.(Intercept)                                 0.31  0.24   0.38
cond.Std.Dev.(Intercept)|centre                1.89  1.55   2.49
```

2.1.14. Diseases of the ear and mastoid process (short label: Ear.mastoid)

```
Family: nbinom2  ( log )
Formula:
m1[, var] ~ `% adults (pat.)` + `% male (pat.)` + `% Nigerian pat.` +
    `% Afghani pat.` + `% Syrian pat.` + `% Iraqi pat.` + `% Turkish pat.` +
    `Peri-pandemic time trend` + time + `Peri-pandemic Inc.` +
    (1 | centre) + offset(log(patients))
Zero inflation:             ~1
Data: m1

     AIC      BIC   logLik deviance df.resid
  2652.4   2718.6  -1312.2   2624.4      819

Random effects:

Conditional model:
 Groups Name        Variance Std.Dev.
 centre (Intercept) 0.249    0.499
Number of obs: 833, groups:  centre, 25

Dispersion parameter for nbinom2 family (): 15.5

Conditional model:
                             Estimate Std. Error z value Pr(>|z|)
(Intercept)                -3.0908416  0.5066898  -6.100 1.06e-09 ***
`% adults (pat.)`          -0.1994617  0.0770308  -2.589  0.00961 **
`% male (pat.)`             0.0942341  0.0594699   1.585  0.11306
`% Nigerian pat.`          -0.3112341  0.4937532  -0.630  0.52847
`% Afghani pat.`            0.1152962  0.2732895   0.422  0.67311
`% Syrian pat.`            -0.7659059  0.3860241  -1.984  0.04725 *
`% Iraqi pat.`              0.7741997  0.9619189   0.805  0.42091
`% Turkish pat.`            0.4407597  0.4784947   0.921  0.35698
`Peri-pandemic time trend` -0.0090287  0.0088433  -1.021  0.30728
time                        0.0007974  0.0084003   0.095  0.92437
`Peri-pandemic Inc.`        0.0683099  0.1336840   0.511  0.60937
---
Signif. codes:  0 '***' 0.001 '**' 0.01 '*' 0.05 '.' 0.1 ' ' 1

Zero-inflation model:
            Estimate Std. Error z value Pr(>|z|)
(Intercept)  -0.2061     0.1099  -1.875   0.0607 .
---
Signif. codes:  0 '***' 0.001 '**' 0.01 '*' 0.05 '.' 0.1 ' ' 1
                                incident rate ratio 2.5 % 97.5 %
cond.(Intercept)                               0.05  0.02   0.12
cond.`% adults (pat.)`                         0.82  0.70   0.95
cond.`% male (pat.)`                           1.10  0.98   1.23
cond.`% Nigerian pat.`                         0.73  0.28   1.93
cond.`% Afghani pat.`                          1.12  0.66   1.92
cond.`% Syrian pat.`                           0.46  0.22   0.99
cond.`% Iraqi pat.`                            2.17  0.33  14.29
cond.`% Turkish pat.`                          1.55  0.61   3.97
cond.`Peri-pandemic time trend`                0.99  0.97   1.01
cond.time                                      1.00  0.98   1.02
cond.`Peri-pandemic Inc.`                      1.07  0.82   1.39
zi.(Intercept)                                 0.81  0.66   1.01
cond.Std.Dev.(Intercept)|centre                1.65  1.38   2.15
```

2.1.15. Diseases of the eye and adnexa (short label: Eye.adnexa)

```
Family: nbinom2  ( log )
Formula:          
m1[, var] ~ `% adults (pat.)` + `% male (pat.)` + `% Nigerian pat.` +  
    `% Afghani pat.` + `% Syrian pat.` + `% Iraqi pat.` + `% Turkish pat.` +  
    `Peri-pandemic time trend` + time + `Peri-pandemic Inc.` +  
    (1 | centre) + offset(log(patients))
Zero inflation:             ~1
Data: m1

     AIC      BIC   logLik deviance df.resid 
  3190.9   3257.0  -1581.4   3162.9      819 

Random effects:

Conditional model:
 Groups Name        Variance Std.Dev.
 centre (Intercept) 0.8383   0.9156  
Number of obs: 833, groups:  centre, 25

Dispersion parameter for nbinom2 family (): 19.6 

Conditional model:
                            Estimate Std. Error z value Pr(>|z|)    
(Intercept)                -4.405537   0.442300  -9.961  < 2e-16 ***
`% adults (pat.)`           0.020197   0.060228   0.335 0.737360    
`% male (pat.)`            -0.012845   0.045119  -0.285 0.775875    
`% Nigerian pat.`           1.136225   0.326541   3.480 0.000502 ***
`% Afghani pat.`           -0.465491   0.208518  -2.232 0.025590 *  
`% Syrian pat.`            -0.460260   0.325584  -1.414 0.157466    
`% Iraqi pat.`              1.439972   0.852072   1.690 0.091035 .  
`% Turkish pat.`           -0.447603   0.359834  -1.244 0.213530    
`Peri-pandemic time trend` -0.011148   0.006533  -1.706 0.087944 .  
time                        0.007396   0.006098   1.213 0.225160    
`Peri-pandemic Inc.`        0.127019   0.093655   1.356 0.175021    
---
Signif. codes:  0 '***' 0.001 '**' 0.01 '*' 0.05 '.' 0.1 ' ' 1

Zero-inflation model:
            Estimate Std. Error z value Pr(>|z|)    
(Intercept)  -0.8247     0.1185   -6.96  3.4e-12 ***
---
Signif. codes:  0 '***' 0.001 '**' 0.01 '*' 0.05 '.' 0.1 ' ' 1
                                incident rate ratio 2.5 % 97.5 %
cond.(Intercept)                               0.01  0.01   0.03
cond.`% adults (pat.)`                         1.02  0.91   1.15
cond.`% male (pat.)`                           0.99  0.90   1.08
cond.`% Nigerian pat.`                         3.11  1.64   5.91
cond.`% Afghani pat.`                          0.63  0.42   0.94
cond.`% Syrian pat.`                           0.63  0.33   1.19
cond.`% Iraqi pat.`                            4.22  0.79  22.42
cond.`% Turkish pat.`                          0.64  0.32   1.29
cond.`Peri-pandemic time trend`                0.99  0.98   1.00
cond.time                                      1.01  1.00   1.02
cond.`Peri-pandemic Inc.`                      1.14  0.95   1.36
zi.(Intercept)                                 0.44  0.35   0.55
cond.Std.Dev.(Intercept)|centre                2.50  1.89   3.71
```

2.1.16. Pregnancy, childbirth and the puerperium (short label: Pregn.condition)

```
Family: nbinom2  ( log )
Formula:
m1[, var] ~ `% adults (pat.)` + `% male (pat.)` + `% Nigerian pat.` +
    `% Afghani pat.` + `% Syrian pat.` + `% Iraqi pat.` + `% Turkish pat.` +
    `Peri-pandemic time trend` + time + `Peri-pandemic Inc.` +
    (1 | centre) + offset(log(patients))
Zero inflation:             ~1
Data: m1

     AIC      BIC   logLik deviance df.resid
  3512.3   3578.4  -1742.1   3484.3      819

Random effects:

Conditional model:
 Groups Name        Variance Std.Dev.
 centre (Intercept) 2.195    1.482
Number of obs: 833, groups:  centre, 25

Dispersion parameter for nbinom2 family (): 17.1

Conditional model:
                            Estimate Std. Error z value Pr(>|z|)
(Intercept)                -3.111039   0.465880  -6.678 2.43e-11 ***
`% adults (pat.)`           0.097115   0.044942   2.161 0.030705 *
`% male (pat.)`            -0.290336   0.037350  -7.773 7.64e-15 ***
`% Nigerian pat.`           0.875595   0.244497   3.581 0.000342 ***
`% Afghani pat.`           -0.523973   0.193567  -2.707 0.006791 **
`% Syrian pat.`            -0.672549   0.297657  -2.259 0.023854 *
`% Iraqi pat.`              0.734098   0.656844   1.118 0.263731
`% Turkish pat.`            0.770472   0.318342   2.420 0.015509 *
`Peri-pandemic time trend`  0.004857   0.005876   0.827 0.408460
time                       -0.007784   0.005441  -1.431 0.152546
`Peri-pandemic Inc.`        0.079896   0.081664   0.978 0.327902
---
Signif. codes:  0 '***' 0.001 '**' 0.01 '*' 0.05 '.' 0.1 ' ' 1

Zero-inflation model:
            Estimate Std. Error z value Pr(>|z|)
(Intercept)  -1.5194     0.1526  -9.957   <2e-16 ***
---
Signif. codes:  0 '***' 0.001 '**' 0.01 '*' 0.05 '.' 0.1 ' ' 1
                                incident rate ratio 2.5 % 97.5 %
cond.(Intercept)                               0.04  0.02   0.11
cond.`% adults (pat.)`                         1.10  1.01   1.20
cond.`% male (pat.)`                           0.75  0.70   0.80
cond.`% Nigerian pat.`                         2.40  1.49   3.88
cond.`% Afghani pat.`                          0.59  0.41   0.87
cond.`% Syrian pat.`                           0.51  0.28   0.91
cond.`% Iraqi pat.`                            2.08  0.58   7.55
cond.`% Turkish pat.`                          2.16  1.16   4.03
cond.`Peri-pandemic time trend`                1.00  0.99   1.02
cond.time                                      0.99  0.98   1.00
cond.`Peri-pandemic Inc.`                      1.08  0.92   1.27
zi.(Intercept)                                 0.22  0.16   0.30
cond.Std.Dev.(Intercept)|centre                4.40  2.85   8.13
```

2.1.17. Benzodiazepine prescriptions (short label: Benzo.prescrip)

```
Family: nbinom2  ( log )
Formula:
m1[, var] ~ `% adults (pat.)` + `% male (pat.)` + `% Nigerian pat.` +
    `% Afghani pat.` + `% Syrian pat.` + `% Iraqi pat.` + `% Turkish pat.` +
    `Peri-pandemic time trend` + time + `Peri-pandemic Inc.` +
    (1 | centre) + offset(log(patients))
Zero inflation:             ~1
Data: m1

     AIC      BIC   logLik deviance df.resid
  1394.0   1460.2   -683.0   1366.0      819

Random effects:

Conditional model:
 Groups Name        Variance Std.Dev.
 centre (Intercept) 2.981    1.727
Number of obs: 833, groups:  centre, 25

Dispersion parameter for nbinom2 family (): 23.5

Conditional model:
                           Estimate Std. Error z value Pr(>|z|)
(Intercept)                -5.17674    0.96924  -5.341 9.24e-08 ***
`% adults (pat.)`          -0.17223    0.13646  -1.262 0.206889
`% male (pat.)`             0.17414    0.13023   1.337 0.181172
`% Nigerian pat.`          -1.36406    1.10155  -1.238 0.215601
`% Afghani pat.`           -0.83254    0.57748  -1.442 0.149396
`% Syrian pat.`            -3.15498    0.85303  -3.699 0.000217 ***
`% Iraqi pat.`              2.78800    2.09270   1.332 0.182778
`% Turkish pat.`            0.38303    1.06629   0.359 0.719430
`Peri-pandemic time trend` -0.03403    0.01749  -1.945 0.051731 .
time                        0.01691    0.01605   1.054 0.292050
`Peri-pandemic Inc.`        0.04284    0.21307   0.201 0.840640
---
Signif. codes:  0 '***' 0.001 '**' 0.01 '*' 0.05 '.' 0.1 ' ' 1

Zero-inflation model:
            Estimate Std. Error z value Pr(>|z|)
(Intercept)   0.5979     0.1324   4.514 6.35e-06 ***
---
Signif. codes:  0 '***' 0.001 '**' 0.01 '*' 0.05 '.' 0.1 ' ' 1
                                incident rate ratio 2.5 % 97.5 %
cond.(Intercept)                               0.01  0.00   0.04
cond.`% adults (pat.)`                         0.84  0.64   1.10
cond.`% male (pat.)`                           1.19  0.92   1.54
cond.`% Nigerian pat.`                         0.26  0.03   2.21
cond.`% Afghani pat.`                          0.43  0.14   1.35
cond.`% Syrian pat.`                           0.04  0.01   0.23
cond.`% Iraqi pat.`                           16.25  0.27 982.03
cond.`% Turkish pat.`                          1.47  0.18  11.86
cond.`Peri-pandemic time trend`                0.97  0.93   1.00
cond.time                                      1.02  0.99   1.05
cond.`Peri-pandemic Inc.`                      1.04  0.69   1.58
zi.(Intercept)                                 1.82  1.40   2.36
cond.Std.Dev.(Intercept)|centre                5.62  2.99  15.21
```

2.1.18. Mental and behavioural disorders (short label: Psych.condition)

```
Family: nbinom2  ( log )
Formula:
m1[, var] ~ `% adults (pat.)` + `% male (pat.)` + `% Nigerian pat.` +
    `% Afghani pat.` + `% Syrian pat.` + `% Iraqi pat.` + `% Turkish pat.` +
    `Peri-pandemic time trend` + time + `Peri-pandemic Inc.` +
    (1 | centre) + offset(log(patients))
Zero inflation:             ~1
Data: m1

     AIC      BIC   logLik deviance df.resid
  4969.2   5035.4  -2470.6   4941.2      819

Random effects:

Conditional model:
 Groups Name        Variance Std.Dev.
 centre (Intercept) 0.2634   0.5132
Number of obs: 833, groups:  centre, 25

Dispersion parameter for nbinom2 family (): 11.4

Conditional model:
                            Estimate Std. Error z value Pr(>|z|)
(Intercept)               -3.6793425  0.2887046 -12.744  < 2e-16 ***
`% adults (pat.)`          0.0885852  0.0356365   2.486 0.012926 *
`% male (pat.)`            0.0695034  0.0263166   2.641 0.008265 **
`% Nigerian pat.`         -1.0768145  0.2475035  -4.351 1.36e-05 ***
`% Afghani pat.`          -0.1055598  0.1514849  -0.697 0.485907
`% Syrian pat.`           -2.0554698  0.2403574  -8.552  < 2e-16 ***
`% Iraqi pat.`             1.6344078  0.4945719   3.305 0.000951 ***
`% Turkish pat.`          -1.3526825  0.2641851  -5.120 3.05e-07 ***
`Peri-pandemic time trend` -0.0077628  0.0053589  -1.449 0.147453
time                      -0.0003604  0.0050689  -0.071 0.943324
`Peri-pandemic Inc.`       0.2051514  0.0744593   2.755 0.005865 **
---
Signif. codes:  0 '***' 0.001 '**' 0.01 '*' 0.05 '.' 0.1 ' ' 1

Zero-inflation model:
            Estimate Std. Error z value Pr(>|z|)
(Intercept)   -2.432      0.154   -15.8   <2e-16 ***
---
Signif. codes:  0 '***' 0.001 '**' 0.01 '*' 0.05 '.' 0.1 ' ' 1
                                incident rate ratio 2.5 % 97.5 %
cond.(Intercept)                               0.03  0.01   0.04
cond.`% adults (pat.)`                         1.09  1.02   1.17
cond.`% male (pat.)`                           1.07  1.02   1.13
cond.`% Nigerian pat.`                         0.34  0.21   0.55
cond.`% Afghani pat.`                          0.90  0.67   1.21
cond.`% Syrian pat.`                           0.13  0.08   0.21
cond.`% Iraqi pat.`                            5.13  1.94  13.51
cond.`% Turkish pat.`                          0.26  0.15   0.43
cond.`Peri-pandemic time trend`                0.99  0.98   1.00
cond.time                                      1.00  0.99   1.01
cond.`Peri-pandemic Inc.`                      1.23  1.06   1.42
zi.(Intercept)                                 0.09  0.06   0.12
cond.Std.Dev.(Intercept)|centre                1.67  1.44   2.07
```

2.1.19. Psychoactive drug prescriptions (short label: Psych.prescrip.)

```
Family: nbinom2  ( log )
Formula:
m1[, var] ~ `% adults (pat.)` + `% male (pat.)` + `% Nigerian pat.` +
    `% Afghani pat.` + `% Syrian pat.` + `% Iraqi pat.` + `% Turkish pat.` +
    `Peri-pandemic time trend` + time + `Peri-pandemic Inc.` +
    (1 | centre) + offset(log(patients))
Zero inflation:             ~1
Data: m1

     AIC      BIC   logLik deviance df.resid
  2975.5   3041.6  -1473.7   2947.5      819

Random effects:

Conditional model:
 Groups Name        Variance Std.Dev.
 centre (Intercept) 1.865    1.366
Number of obs: 833, groups:  centre, 25

Dispersion parameter for nbinom2 family (): 16.2

Conditional model:
                            Estimate Std. Error z value Pr(>|z|)
(Intercept)                -5.023014   0.516833  -9.719  < 2e-16 ***
`% adults (pat.)`           0.118360   0.061911   1.912  0.05590 .
`% male (pat.)`            -0.117117   0.048256  -2.427  0.01522 *
`% Nigerian pat.`          -0.446378   0.449108  -0.994  0.32026
`% Afghani pat.`           -0.114083   0.232808  -0.490  0.62411
`% Syrian pat.`            -0.346219   0.364970  -0.949  0.34281
`% Iraqi pat.`              1.007335   0.907410   1.110  0.26695
`% Turkish pat.`           -0.010285   0.511443  -0.020  0.98396
`Peri-pandemic time trend` -0.037938   0.007629  -4.973  6.6e-07 ***
time                        0.022761   0.007226   3.150  0.00163 **
`Peri-pandemic Inc.`        0.194214   0.098992   1.962  0.04977 *
---
Signif. codes:  0 '***' 0.001 '**' 0.01 '*' 0.05 '.' 0.1 ' ' 1

Zero-inflation model:
            Estimate Std. Error z value Pr(>|z|)
(Intercept)  -0.7364     0.1027  -7.169 7.57e-13 ***
---
Signif. codes:  0 '***' 0.001 '**' 0.01 '*' 0.05 '.' 0.1 ' ' 1
                                incident rate ratio 2.5 % 97.5 %
cond.(Intercept)                               0.01  0.00   0.02
cond.`% adults (pat.)`                         1.13  1.00   1.27
cond.`% male (pat.)`                           0.89  0.81   0.98
cond.`% Nigerian pat.`                         0.64  0.27   1.54
cond.`% Afghani pat.`                          0.89  0.57   1.41
cond.`% Syrian pat.`                           0.71  0.35   1.45
cond.`% Iraqi pat.`                            2.74  0.46  16.21
cond.`% Turkish pat.`                          0.99  0.36   2.70
cond.`Peri-pandemic time trend`                0.96  0.95   0.98
cond.time                                      1.02  1.01   1.04
cond.`Peri-pandemic Inc.`                      1.21  1.00   1.47
zi.(Intercept)                                 0.48  0.39   0.59
cond.Std.Dev.(Intercept)|centre                3.92  2.65   6.78
```

2.1.20. Diseases of the respiratory system (short label: Respiratory syst.)

```
Family: nbinom2  ( log )
Formula:
m1[, var] ~ `% adults (pat.)` + `% male (pat.)` + `% Nigerian pat.` +
    `% Afghani pat.` + `% Syrian pat.` + `% Iraqi pat.` + `% Turkish pat.` +
    `Peri-pandemic time trend` + time + `Peri-pandemic Inc.` +
    (1 | centre) + offset(log(patients))
Zero inflation:             ~1
Data: m1

     AIC      BIC   logLik deviance df.resid
  5890.4   5956.5  -2931.2   5862.4      819

Random effects:

Conditional model:
 Groups Name        Variance Std.Dev.
 centre (Intercept) 0.1733   0.4163
Number of obs: 833, groups:  centre, 25

Dispersion parameter for nbinom2 family (): 5.52

Conditional model:
                            Estimate Std. Error z value Pr(>|z|)
(Intercept)                 0.233297   0.305374   0.764  0.44488
`% adults (pat.)`          -0.232171   0.039752  -5.840 5.20e-09 ***
`% male (pat.)`             0.021954   0.028021   0.783  0.43335
`% Nigerian pat.`           0.062074   0.235054   0.264  0.79171
`% Afghani pat.`           -0.602000   0.183331  -3.284  0.00102 **
`% Syrian pat.`             0.013895   0.231066   0.060  0.95205
`% Iraqi pat.`             -2.521455   0.630855  -3.997 6.42e-05 ***
`% Turkish pat.`            0.278357   0.320639   0.868  0.38532
`Peri-pandemic time trend`  0.017304   0.006049   2.861  0.00423 **
time                       -0.007825   0.005746  -1.362  0.17326
`Peri-pandemic Inc.`       -0.854503   0.082814 -10.318  < 2e-16 ***
---
Signif. codes:  0 '***' 0.001 '**' 0.01 '*' 0.05 '.' 0.1 ' ' 1

Zero-inflation model:
            Estimate Std. Error z value Pr(>|z|)
(Intercept)  -2.3459     0.1524  -15.39   <2e-16 ***
---
Signif. codes:  0 '***' 0.001 '**' 0.01 '*' 0.05 '.' 0.1 ' ' 1
                                incident rate ratio 2.5 % 97.5 %
cond.(Intercept)                               1.26  0.69   2.30
cond.`% adults (pat.)`                         0.79  0.73   0.86
cond.`% male (pat.)`                           1.02  0.97   1.08
cond.`% Nigerian pat.`                         1.06  0.67   1.69
cond.`% Afghani pat.`                          0.55  0.38   0.78
cond.`% Syrian pat.`                           1.01  0.64   1.59
cond.`% Iraqi pat.`                            0.08  0.02   0.28
cond.`% Turkish pat.`                          1.32  0.70   2.48
cond.`Peri-pandemic time trend`                1.02  1.01   1.03
cond.time                                      0.99  0.98   1.00
cond.`Peri-pandemic Inc.`                      0.43  0.36   0.50
zi.(Intercept)                                 0.10  0.07   0.13
cond.Std.Dev.(Intercept)|centre                1.52  1.36   1.75
```

2.1.21. Diseases of the genitourinary system (short label: Genitourinary syst.)

```
Family: nbinom2  ( log )
Formula:
m1[, var] ~ `% adults (pat.)` + `% male (pat.)` + `% Nigerian pat.` +
    `% Afghani pat.` + `% Syrian pat.` + `% Iraqi pat.` + `% Turkish pat.` +
    `Peri-pandemic time trend` + time + `Peri-pandemic Inc.` +
    (1 | centre) + offset(log(patients))
Zero inflation:            ~1
Data: m1

     AIC      BIC   logLik deviance df.resid
  3897.2   3963.3  -1934.6   3869.2      819

Random effects:

Conditional model:
 Groups Name        Variance Std.Dev.
 centre (Intercept) 0.5943   0.7709
Number of obs: 833, groups:  centre, 25

Dispersion parameter for nbinom2 family (): 27.5

Conditional model:
                            Estimate Std. Error z value Pr(>|z|)
(Intercept)                -3.431118   0.338215 -10.145  < 2e-16 ***
`% adults (pat.)`           0.082182   0.040015   2.054   0.0400 *
`% male (pat.)`            -0.120329   0.030781  -3.909 9.26e-05 ***
`% Nigerian pat.`           0.353303   0.235175   1.502   0.1330
`% Afghani pat.`           -0.130645   0.159883  -0.817   0.4139
`% Syrian pat.`            -0.492830   0.237050  -2.079   0.0376 *
`% Iraqi pat.`              0.488616   0.557512   0.876   0.3808
`% Turkish pat.`           -0.486583   0.283500  -1.716   0.0861 .
`Peri-pandemic time trend` -0.010481   0.005359  -1.956   0.0505 .
time                        0.001738   0.005085   0.342   0.7324
`Peri-pandemic Inc.`        0.163252   0.073031   2.235   0.0254 *
---
Signif. codes:  0 '***' 0.001 '**' 0.01 '*' 0.05 '.' 0.1 ' ' 1

Zero-inflation model:
            Estimate Std. Error z value Pr(>|z|)
(Intercept)  -1.3938     0.1141  -12.21   <2e-16 ***
---
Signif. codes:  0 '***' 0.001 '**' 0.01 '*' 0.05 '.' 0.1 ' ' 1
                                incident rate ratio 2.5 % 97.5 %
cond.(Intercept)                               0.03  0.02   0.06
cond.`% adults (pat.)`                         1.09  1.00   1.17
cond.`% male (pat.)`                           0.89  0.83   0.94
cond.`% Nigerian pat.`                         1.42  0.90   2.26
cond.`% Afghani pat.`                          0.88  0.64   1.20
cond.`% Syrian pat.`                           0.61  0.38   0.97
cond.`% Iraqi pat.`                            1.63  0.55   4.86
cond.`% Turkish pat.`                          0.61  0.35   1.07
cond.`Peri-pandemic time trend`                0.99  0.98   1.00
cond.time                                      1.00  0.99   1.01
cond.`Peri-pandemic Inc.`                      1.18  1.02   1.36
zi.(Intercept)                                 0.25  0.20   0.31
cond.Std.Dev.(Intercept)|centre                2.16  1.74   2.91
```

2.2 Detailed results for the sensitivity analysis 2

In sensitivity analysis 2, we repeated the main analysis on subset 3, where the occupancy totals equal the totals in occupancy age-and sex-strata AND n_occ≥n_pat. The description of subset 3 (see additional excel file Table S2), original R output for each model Code 2), as well as a forest plot showing the effects of the variables Peri-pandemic time trend and Peri-pandemic Incidence (Ref.: Pre-pandemic) (Figure S2) are shown below. The model for the variable Hypertension (short label: Hypertension) did not converge, therefore, the model is not shown in sensitivity analysis 2.

2.2.1. Disabilities (short label: Disability)

```
Family: nbinom2  ( log )
Formula:
m1[, var] ~ `% adults (occup.)` + `% male (occup.)` + `Peri-pandemic time trend` +
    time + `Peri-pandemic Inc.` + (1 | centre) + offset(log(occupancy))
Zero inflation:            ~1
Data: m1

     AIC      BIC   logLik deviance df.resid
   869.7    901.0   -425.8    851.7      230

Random effects:

Conditional model:
 Groups Name        Variance Std.Dev.
 centre (Intercept) 1.918    1.385
Number of obs: 239, groups:  centre, 19

Dispersion parameter for nbinom2 family (): 5.46

Conditional model:
                           Estimate Std. Error z value Pr(>|z|)
(Intercept)                -5.40045    0.98521  -5.482 4.22e-08 ***
`% adults (occup.)`        -0.52062    0.17725  -2.937  0.00331 **
`% male (occup.)`           0.55229    0.16796   3.288  0.00101 **
`Peri-pandemic time trend` -0.02639    0.02689  -0.982  0.32631
time                        0.01826    0.02620   0.697  0.48588
`Peri-pandemic Inc.`        0.19353    0.29752   0.650  0.51539
---
Signif. codes:  0 '***' 0.001 '**' 0.01 '*' 0.05 '.' 0.1 ' ' 1

Zero-inflation model:
            Estimate Std. Error z value Pr(>|z|)
(Intercept)  -0.3848     0.2122  -1.814   0.0697 .
---
Signif. codes:  0 '***' 0.001 '**' 0.01 '*' 0.05 '.' 0.1 ' ' 1
                                 incident rate ratio 2.5 % 97.5 %
cond.(Intercept)                                0.00  0.00   0.03
cond.`% adults (occup.)`                        0.59  0.42   0.84
cond.`% male (occup.)`                          1.74  1.25   2.41
cond.`Peri-pandemic time trend`                 0.97  0.92   1.03
cond.time                                       1.02  0.97   1.07
cond.`Peri-pandemic Inc.`                       1.21  0.68   2.17
zi.(Intercept)                                  0.68  0.45   1.03
cond.Std.Dev.(Intercept)|centre                 3.99  2.17  11.94
```

2.2.2. Injury, poisoning and certain other consequences of external causes (short label: Cons.ext.causes)

```
Family: nbinom2  ( log )
Formula:
m1[, var] ~ `% adults (occup.)` + `% male (occup.)` + `Peri-pandemic time trend` +
    time + `Peri-pandemic Inc.` + (1 | centre) + offset(log(occupancy))
Zero inflation:             ~1
Data: m1

     AIC      BIC   logLik deviance df.resid
  1376.7   1408.0   -679.4   1358.7      230

Random effects:

Conditional model:
 Groups Name        Variance Std.Dev.
 centre (Intercept) 0.1475   0.3841
Number of obs: 239, groups:  centre, 19

Dispersion parameter for nbinom2 family ():   11

Conditional model:
                           Estimate Std. Error z value Pr(>|z|)
(Intercept)                -4.05240    0.47394  -8.550  < 2e-16 ***
`% adults (occup.)`        -0.03409    0.08977  -0.380 0.704154
`% male (occup.)`           0.10976    0.07844   1.399 0.161706
`Peri-pandemic time trend`  0.01334    0.01567   0.852 0.394441
time                       -0.02363    0.01530  -1.544 0.122547
`Peri-pandemic Inc.`        0.59212    0.16119   3.673 0.000239 ***
---
Signif. codes:  0 '***' 0.001 '**' 0.01 '*' 0.05 '.' 0.1 ' ' 1

Zero-inflation model:
            Estimate Std. Error z value Pr(>|z|)
(Intercept)  -1.3346     0.1609  -8.297   <2e-16 ***
---
Signif. codes:  0 '***' 0.001 '**' 0.01 '*' 0.05 '.' 0.1 ' ' 1
                                incident rate ratio 2.5 % 97.5 %
cond.(Intercept)                               0.02  0.01   0.04
cond.`% adults (occup.)`                       0.97  0.81   1.15
cond.`% male (occup.)`                         1.12  0.96   1.30
cond.`Peri-pandemic time trend`                1.01  0.98   1.05
cond.time                                      0.98  0.95   1.01
cond.`Peri-pandemic Inc.`                      1.81  1.32   2.48
zi.(Intercept)                                 0.26  0.19   0.36
cond.Std.Dev.(Intercept)|centre                1.47  1.29   1.79
```

2.2.3. Diseases of the skin and subcutaneous tissue (short label: Skin)

```
Family: nbinom2  ( log )
Formula:
m1[, var] ~ `% adults (occup.)` + `% male (occup.)` + `Peri-pandemic time trend` +
    time + `Peri-pandemic Inc.` + (1 | centre) + offset(log(occupancy))
Zero inflation:             ~1
Data: m1

     AIC      BIC   logLik deviance df.resid
  1398.8   1430.1   -690.4   1380.8      230

Random effects:

Conditional model:
 Groups Name        Variance Std.Dev.
 centre (Intercept) 0.4601   0.6783
Number of obs: 239, groups:  centre, 19

Dispersion parameter for nbinom2 family (): 14.6

Conditional model:
                            Estimate Std. Error z value Pr(>|z|)
(Intercept)                -4.192429   0.460519  -9.104   <2e-16 ***
`% adults (occup.)`        -0.061129   0.084425  -0.724   0.4690
`% male (occup.)`           0.134904   0.075197   1.794   0.0728 .
`Peri-pandemic time trend`  0.000237   0.013425   0.018   0.9859
time                       -0.003477   0.013094  -0.266   0.7906
`Peri-pandemic Inc.`        0.179096   0.141353   1.267   0.2052
---
Signif. codes:  0 '***' 0.001 '**' 0.01 '*' 0.05 '.' 0.1 ' ' 1

Zero-inflation model:
            Estimate Std. Error z value Pr(>|z|)
(Intercept)  -2.0483     0.2407  -8.509   <2e-16 ***
---
Signif. codes:  0 '***' 0.001 '**' 0.01 '*' 0.05 '.' 0.1 ' ' 1
                                incident rate ratio 2.5 % 97.5 %
cond.(Intercept)                               0.02  0.01   0.04
cond.`% adults (occup.)`                       0.94  0.80   1.11
cond.`% male (occup.)`                         1.14  0.99   1.33
cond.`Peri-pandemic time trend`                1.00  0.97   1.03
cond.time                                      1.00  0.97   1.02
cond.`Peri-pandemic Inc.`                      1.20  0.91   1.58
zi.(Intercept)                                 0.13  0.08   0.21
cond.Std.Dev.(Intercept)|centre                1.97  1.57   2.76
```

2.2.4. Diseases of the digestive system (short label: Digestive syst.)

```
Family: nbinom2  ( log )
Formula:
m1[, var] ~ `% adults (occup.)` + `% male (occup.)` + `Peri-pandemic time trend` +
    time + `Peri-pandemic Inc.` + (1 | centre) + offset(log(occupancy))
Zero inflation:             ~1
Data: m1

     AIC      BIC   logLik deviance df.resid
  1594.6   1625.8   -788.3   1576.6      230

Random effects:

Conditional model:
 Groups Name        Variance Std.Dev.
 centre (Intercept) 0.3809   0.6172
Number of obs: 239, groups:  centre, 19

Dispersion parameter for nbinom2 family (): 16.7

Conditional model:
                            Estimate Std. Error z value Pr(>|z|)
(Intercept)                -2.783569   0.361063  -7.709 1.26e-14 ***
`% adults (occup.)`        -0.207295   0.065309  -3.174  0.00150 **
`% male (occup.)`           0.177420   0.061228   2.898  0.00376 **
`Peri-pandemic time trend`  0.006152   0.011034   0.558  0.57713
time                       -0.008582   0.010743  -0.799  0.42441
`Peri-pandemic Inc.`        0.148243   0.118961   1.246  0.21271
---
Signif. codes:  0 '***' 0.001 '**' 0.01 '*' 0.05 '.' 0.1 ' ' 1

Zero-inflation model:
            Estimate Std. Error z value Pr(>|z|)
(Intercept)   -3.504      0.476  -7.361 1.83e-13 ***
---
Signif. codes:  0 '***' 0.001 '**' 0.01 '*' 0.05 '.' 0.1 ' ' 1
                                incident rate ratio 2.5 % 97.5 %
cond.(Intercept)                               0.06  0.03   0.13
cond.`% adults (occup.)`                       0.81  0.72   0.92
cond.`% male (occup.)`                         1.19  1.06   1.35
cond.`Peri-pandemic time trend`                1.01  0.98   1.03
cond.time                                      0.99  0.97   1.01
cond.`Peri-pandemic Inc.`                      1.16  0.92   1.46
zi.(Intercept)                                 0.03  0.01   0.08
cond.Std.Dev.(Intercept)|centre                1.85  1.53   2.45
```

2.2.5. Diseases of the blood and blood-forming organs and certain disorders involving the immune mechanism (short label: Blood)

```
Family: nbinom2  ( log )
Formula:
m1[, var] ~ `% adults (occup.)` + `% male (occup.)` + `Peri-pandemic time trend` +
    time + `Peri-pandemic Inc.` + (1 | centre) + offset(log(occupancy))
Zero inflation:            ~1
Data: m1

     AIC      BIC   logLik deviance df.resid
   590.4    621.7   -286.2    572.4      230

Random effects:

Conditional model:
 Groups Name        Variance Std.Dev.
 centre (Intercept) 5.197    2.28
Number of obs: 239, groups:  centre, 19

Dispersion parameter for nbinom2 family (): 22.7

Conditional model:
                           Estimate Std. Error z value Pr(>|z|)
(Intercept)                -4.56302    1.18371  -3.855 0.000116 ***
`% adults (occup.)`        -0.40290    0.21245  -1.896 0.057901 .
`% male (occup.)`           0.18690    0.20744   0.901 0.367599
`Peri-pandemic time trend`  0.02058    0.01969   1.045 0.295895
time                       -0.04614    0.02049  -2.252 0.024328 *
`Peri-pandemic Inc.`        0.45374    0.20892   2.172 0.029868 *
---
Signif. codes:  0 '***' 0.001 '**' 0.01 '*' 0.05 '.' 0.1 ' ' 1

Zero-inflation model:
            Estimate Std. Error z value Pr(>|z|)
(Intercept)  -0.2650     0.2583  -1.026    0.305
                                incident rate ratio 2.5 % 97.5 %
cond.(Intercept)                               0.01  0.00   0.11
cond.`% adults (occup.)`                       0.67  0.44   1.01
cond.`% male (occup.)`                         1.21  0.80   1.81
cond.`Peri-pandemic time trend`                1.02  0.98   1.06
cond.time                                      0.95  0.92   0.99
cond.`Peri-pandemic Inc.`                      1.57  1.05   2.37
zi.(Intercept)                                 0.77  0.46   1.27
cond.Std.Dev.(Intercept)|centre                9.77  3.44  67.36
```

2.2.6. Certain infectious and parasitic diseases (short label: Inf.diseases)

```
Family: nbinom2  ( log )
Formula:
m1[, var] ~ `% adults (occup.)` + `% male (occup.)` + `Peri-pandemic time trend` +
    time + `Peri-pandemic Inc.` + (1 | centre) + offset(log(occupancy))
Zero inflation:             ~1
Data: m1

     AIC      BIC   logLik deviance df.resid
  1682.4   1713.7   -832.2   1664.4      230

Random effects:

Conditional model:
 Groups Name        Variance Std.Dev.
 centre (Intercept) 0.5103   0.7144
Number of obs: 239, groups:  centre, 19

Dispersion parameter for nbinom2 family (): 11.1

Conditional model:
                             Estimate Std. Error z value Pr(>|z|)
(Intercept)                -3.5313614  0.4328828  -8.158 3.41e-16 ***
`% adults (occup.)`        -0.1132578  0.0745628  -1.519  0.12877
`% male (occup.)`           0.1938355  0.0715659   2.708  0.00676 **
`Peri-pandemic time trend`  0.0062331  0.0123018   0.507  0.61238
time                        0.0008531  0.0118481   0.072  0.94260
`Peri-pandemic Inc.`       -0.1197251  0.1361063  -0.880  0.37905
---
Signif. codes:  0 '***' 0.001 '**' 0.01 '*' 0.05 '.' 0.1 ' ' 1

Zero-inflation model:
            Estimate Std. Error z value Pr(>|z|)
(Intercept)  -2.5261     0.2944   -8.58   <2e-16 ***
---
Signif. codes:  0 '***' 0.001 '**' 0.01 '*' 0.05 '.' 0.1 ' ' 1
                                incident rate ratio 2.5 % 97.5 %
cond.(Intercept)                               0.03  0.01   0.07
cond.`% adults (occup.)`                       0.89  0.77   1.03
cond.`% male (occup.)`                         1.21  1.06   1.40
cond.`Peri-pandemic time trend`                1.01  0.98   1.03
cond.time                                      1.00  0.98   1.02
cond.`Peri-pandemic Inc.`                      0.89  0.68   1.16
zi.(Intercept)                                 0.08  0.04   0.14
cond.Std.Dev.(Intercept)|centre                2.04  1.62   2.88
```

2.2.7. Notifiable infectious diseases (short label: Inf.notify)

```
Family: nbinom2  ( log )
Formula:
m1[, var] ~ `% adults (occup.)` + `% male (occup.)` + `Peri-pandemic time trend`
+
    time + `Peri-pandemic Inc.` + (1 | centre) + offset(log(occupancy))
Zero inflation:             ~1
Data: m1

     AIC      BIC   logLik deviance df.resid
   703.5    734.8   -342.8    685.5      230

Random effects:

Conditional model:
 Groups Name        Variance Std.Dev.
 centre (Intercept) 1.696    1.302
Number of obs: 239, groups:  centre, 19

Dispersion parameter for nbinom2 family (): 25.4

Conditional model:
                           Estimate Std. Error z value Pr(>|z|)
(Intercept)                -3.61065    0.92868  -3.888 0.000101 ***
`% adults (occup.)`        -0.18198    0.18975  -0.959 0.337549
`% male (occup.)`           0.09919    0.17747   0.559 0.576217
`Peri-pandemic time trend`  0.04381    0.01852   2.366 0.017997 *
time                       -0.05762    0.01926  -2.992 0.002775 **
`Peri-pandemic Inc.`        0.36967    0.21270   1.738 0.082217 .
---
Signif. codes:  0 '***' 0.001 '**' 0.01 '*' 0.05 '.' 0.1 ' ' 1

Zero-inflation model:
            Estimate Std. Error z value Pr(>|z|)
(Intercept)   0.1874     0.1878   0.998    0.318
                                incident rate ratio 2.5 % 97.5 %
cond.(Intercept)                               0.03  0.00   0.17
cond.`% adults (occup.)`                       0.83  0.57   1.21
cond.`% male (occup.)`                         1.10  0.78   1.56
cond.`Peri-pandemic time trend`                1.04  1.01   1.08
cond.time                                      0.94  0.91   0.98
cond.`Peri-pandemic Inc.`                      1.45  0.95   2.20
zi.(Intercept)                                 1.21  0.83   1.74
cond.Std.Dev.(Intercept)|centre                3.68  2.14   9.29
```

2.2.8. Diseases of the circulatory system (short label: Circulatory syst.)

```
Family: nbinom2  ( log )
Formula:
m1[, var] ~ `% adults (occup.)` + `% male (occup.)` + `Peri-pandemic time trend` +
    time + `Peri-pandemic Inc.` + (1 | centre) + offset(log(occupancy))
Zero inflation:             ~1
Data: m1

     AIC      BIC   logLik deviance df.resid
  1291.6   1322.8   -636.8   1273.6      230

Random effects:

Conditional model:
 Groups Name        Variance Std.Dev.
 centre (Intercept) 0.204    0.4516
Number of obs: 239, groups:  centre, 19

Dispersion parameter for nbinom2 family (): 11.7

Conditional model:
                           Estimate Std. Error z value Pr(>|z|)
(Intercept)                -3.66912    0.51667  -7.101 1.23e-12 ***
`% adults (occup.)`         0.07027    0.10537   0.667   0.5048
`% male (occup.)`          -0.10968    0.09009  -1.217   0.2234
`Peri-pandemic time trend`  0.02576    0.01668   1.544   0.1226
time                       -0.02458    0.01635  -1.504   0.1326
`Peri-pandemic Inc.`        0.32736    0.16730   1.957   0.0504 .
---
Signif. codes:  0 '***' 0.001 '**' 0.01 '*' 0.05 '.' 0.1 ' ' 1

Zero-inflation model:
            Estimate Std. Error z value Pr(>|z|)
(Intercept)  -1.5596     0.2305  -6.767 1.32e-11 ***
---
Signif. codes:  0 '***' 0.001 '**' 0.01 '*' 0.05 '.' 0.1 ' ' 1
                                incident rate ratio 2.5 % 97.5 %
cond.(Intercept)                               0.03  0.01   0.07
cond.`% adults (occup.)`                       1.07  0.87   1.32
cond.`% male (occup.)`                         0.90  0.75   1.07
cond.`Peri-pandemic time trend`                1.03  0.99   1.06
cond.time                                      0.98  0.94   1.01
cond.`Peri-pandemic Inc.`                      1.39  1.00   1.93
zi.(Intercept)                                 0.21  0.13   0.33
cond.Std.Dev.(Intercept)|centre                1.57  1.34   2.01
```

2.2.9. Endocrine, nutritional and metabolic diseases (short label: Metabolic)

```
Family: nbinom2  ( log )
Formula:
m1[, var] ~ `% adults (occup.)` + `% male (occup.)` + `Peri-pandemic time trend` +
    time + `Peri-pandemic Inc.` + (1 | centre) + offset(log(occupancy))
Zero inflation:            ~1
Data: m1

     AIC      BIC   logLik deviance df.resid
  1178.9   1210.2   -580.4   1160.9      230

Random effects:

Conditional model:
 Groups Name        Variance Std.Dev.
 centre (Intercept) 0.468    0.6841
Number of obs: 239, groups:  centre, 19

Dispersion parameter for nbinom2 family (): 12.2

Conditional model:
                           Estimate Std. Error z value Pr(>|z|)
(Intercept)                -4.09451    0.57391  -7.134 9.72e-13 ***
`% adults (occup.)`         0.12423    0.11630   1.068   0.2855
`% male (occup.)`          -0.22798    0.11017  -2.069   0.0385 *
`Peri-pandemic time trend` -0.01142    0.01680  -0.680   0.4965
time                        0.01118    0.01658   0.674   0.5001
`Peri-pandemic Inc.`        0.36593    0.17446   2.097   0.0360 *
---
Signif. codes:  0 '***' 0.001 '**' 0.01 '*' 0.05 '.' 0.1 ' ' 1

Zero-inflation model:
            Estimate Std. Error z value Pr(>|z|)
(Intercept)  -1.1609     0.1986  -5.844 5.08e-09 ***
---
Signif. codes:  0 '***' 0.001 '**' 0.01 '*' 0.05 '.' 0.1 ' ' 1
                                incident rate ratio 2.5 % 97.5 %
cond.(Intercept)                               0.02  0.01   0.05
cond.`% adults (occup.)`                       1.13  0.90   1.42
cond.`% male (occup.)`                         0.80  0.64   0.99
cond.`Peri-pandemic time trend`                0.99  0.96   1.02
cond.time                                      1.01  0.98   1.04
cond.`Peri-pandemic Inc.`                      1.44  1.02   2.03
zi.(Intercept)                                 0.31  0.21   0.46
cond.Std.Dev.(Intercept)|centre                1.98  1.52   3.07
```

2.2.10. Diabetes mellitus (short label: Diabetes)

```
Family: nbinom2  ( log )
Formula:
m1[, var] ~ `% adults (occup.)` + `% male (occup.)` + `Peri-pandemic time trend` +
    time + `Peri-pandemic Inc.` + (1 | centre) + offset(log(occupancy))
Zero inflation:             ~1
Data: m1

     AIC      BIC   logLik deviance df.resid
   819.0    850.3   -400.5    801.0      230

Random effects:

Conditional model:
 Groups Name        Variance Std.Dev.
 centre (Intercept) 2.39     1.546
Number of obs: 239, groups:  centre, 19

Dispersion parameter for nbinom2 family (): 11.9

Conditional model:
                           Estimate Std. Error z value Pr(>|z|)
(Intercept)                -5.72639    0.86530  -6.618 3.65e-11 ***
`% adults (occup.)`         0.27747    0.16233   1.709   0.0874 .
`% male (occup.)`          -0.35118    0.15356  -2.287   0.0222 *
`Peri-pandemic time trend`  0.02691    0.02157   1.248   0.2122
time                       -0.01410    0.02149  -0.656   0.5118
`Peri-pandemic Inc.`        0.30862    0.22133   1.394   0.1632
---
Signif. codes:  0 '***' 0.001 '**' 0.01 '*' 0.05 '.' 0.1 ' ' 1

Zero-inflation model:
            Estimate Std. Error z value Pr(>|z|)
(Intercept)  -0.9806     0.2410   -4.07 4.71e-05 ***
---
Signif. codes:  0 '***' 0.001 '**' 0.01 '*' 0.05 '.' 0.1 ' ' 1
                                incident rate ratio 2.5 % 97.5 %
cond.(Intercept)                               0.00  0.00   0.02
cond.`% adults (occup.)`                       1.32  0.96   1.81
cond.`% male (occup.)`                         0.70  0.52   0.95
cond.`Peri-pandemic time trend`                1.03  0.98   1.07
cond.time                                      0.99  0.95   1.03
cond.`Peri-pandemic Inc.`                      1.36  0.88   2.10
zi.(Intercept)                                 0.38  0.23   0.60
cond.Std.Dev.(Intercept)|centre                4.69  2.56  12.66
```

2.2.11. Diseases of the musculoskeletal system and connective tissue (short label: Musculoskelet. syst.)

```
Family: nbinom2  ( log )
Formula:
m1[, var] ~ `% adults (occup.)` + `% male (occup.)` + `Peri-pandemic time trend` +
    time + `Peri-pandemic Inc.` + (1 | centre) + offset(log(occupancy))
Zero inflation:             ~1
Data: m1

     AIC      BIC   logLik deviance df.resid
  1514.7   1546.0   -748.4   1496.7      230

Random effects:

Conditional model:
 Groups Name        Variance Std.Dev.
 centre (Intercept) 0.2855   0.5344
Number of obs: 239, groups:  centre, 19

Dispersion parameter for nbinom2 family (): 14.3

Conditional model:
                            Estimate Std. Error z value Pr(>|z|)
(Intercept)                -3.292168   0.392584  -8.386   <2e-16 ***
`% adults (occup.)`        -0.092210   0.071380  -1.292   0.1964
`% male (occup.)`           0.103486   0.067135   1.541   0.1232
`Peri-pandemic time trend`  0.007846   0.012111   0.648   0.5171
time                       -0.018451   0.011740  -1.572   0.1160
`Peri-pandemic Inc.`        0.320197   0.129530   2.472   0.0134 *
---
Signif. codes:  0 '***' 0.001 '**' 0.01 '*' 0.05 '.' 0.1 ' ' 1

Zero-inflation model:
            Estimate Std. Error z value Pr(>|z|)
(Intercept)  -2.6152     0.3304  -7.915 2.46e-15 ***
---
Signif. codes:  0 '***' 0.001 '**' 0.01 '*' 0.05 '.' 0.1 ' ' 1
                                incident rate ratio 2.5 % 97.5 %
cond.(Intercept)                               0.04  0.02   0.08
cond.`% adults (occup.)`                       0.91  0.79   1.05
cond.`% male (occup.)`                         1.11  0.97   1.26
cond.`Peri-pandemic time trend`                1.01  0.98   1.03
cond.time                                      0.98  0.96   1.00
cond.`Peri-pandemic Inc.`                      1.38  1.07   1.78
zi.(Intercept)                                 0.07  0.04   0.14
cond.Std.Dev.(Intercept)|centre                1.71  1.43   2.24
```

2.2.12. Neoplasms (short label: Neoplasm)

```
Family: nbinom2  ( log )
Formula:
m1[, var] ~ `% adults (occup.)` + `% male (occup.)` + `Peri-pandemic time trend` +
    time + `Peri-pandemic Inc.` + (1 | centre) + offset(log(occupancy))
Zero inflation:             ~1
Data: m1

     AIC      BIC   logLik deviance df.resid
   550.4    581.6   -266.2    532.4      230

Random effects:

Conditional model:
 Groups Name        Variance Std.Dev.
 centre (Intercept) 13.16    3.627
Number of obs: 239, groups:  centre, 19

Dispersion parameter for nbinom2 family (): 22.2

Conditional model:
                            Estimate Std. Error z value Pr(>|z|)
(Intercept)                -8.839482   2.213216  -3.994  6.5e-05 ***
`% adults (occup.)`         0.255468   0.236687   1.079   0.2804
`% male (occup.)`          -0.323105   0.235418  -1.372   0.1699
`Peri-pandemic time trend` -0.001380   0.021423  -0.064   0.9487
time                       -0.001592   0.022714  -0.070   0.9441
`Peri-pandemic Inc.`        0.466146   0.224357   2.078   0.0377 *
---
Signif. codes:  0 '***' 0.001 '**' 0.01 '*' 0.05 '.' 0.1 ' ' 1

Zero-inflation model:
            Estimate Std. Error z value Pr(>|z|)
(Intercept)  -0.3434     0.2316  -1.482    0.138
                                incident rate ratio 2.5 %  97.5 %
cond.(Intercept)                              0.00  0.00    0.01
cond.`% adults (occup.)`                      1.29  0.81    2.05
cond.`% male (occup.)`                        0.72  0.46    1.15
cond.`Peri-pandemic time trend`               1.00  0.96    1.04
cond.time                                     1.00  0.95    1.04
cond.`Peri-pandemic Inc.`                     1.59  1.03    2.47
zi.(Intercept)                                0.71  0.45    1.12
cond.Std.Dev.(Intercept)|centre              37.60  4.31 8166.14
```

2.2.13. Diseases of the nervous system (short label: Nervous syst.)

```
Family: nbinom2  ( log )
Formula:
m1[, var] ~ `% adults (occup.)` + `% male (occup.)` + `Peri-pandemic time trend` +
    time + `Peri-pandemic Inc.` + (1 | centre) + offset(log(occupancy))
Zero inflation:             ~1
Data: m1

     AIC      BIC   logLik deviance df.resid
  1191.7   1222.9   -586.8   1173.7      230

Random effects:

Conditional model:
 Groups Name        Variance Std.Dev.
 centre (Intercept) 0.9611   0.9804
Number of obs: 239, groups:  centre, 19

Dispersion parameter for nbinom2 family (): 8.92

Conditional model:
                            Estimate Std. Error z value Pr(>|z|)
(Intercept)               -4.4477063  0.6686563  -6.652  2.9e-11 ***
`% adults (occup.)`        0.0471092  0.1178180   0.400    0.689
`% male (occup.)`         -0.0375216  0.1163595  -0.322    0.747
`Peri-pandemic time trend`  0.0008918  0.0181117   0.049    0.961
time                      -0.0076241  0.0177619  -0.429    0.668
`Peri-pandemic Inc.`       0.2227041  0.1933077   1.152    0.249
---
Signif. codes:  0 '***' 0.001 '**' 0.01 '*' 0.05 '.' 0.1 ' ' 1

Zero-inflation model:
            Estimate Std. Error z value Pr(>|z|)
(Intercept)  -1.5704     0.2334   -6.73  1.7e-11 ***
---
Signif. codes:  0 '***' 0.001 '**' 0.01 '*' 0.05 '.' 0.1 ' ' 1
                                incident rate ratio 2.5 % 97.5 %
cond.(Intercept)                               0.01  0.00   0.04
cond.`% adults (occup.)`                       1.05  0.83   1.32
cond.`% male (occup.)`                         0.96  0.77   1.21
cond.`Peri-pandemic time trend`                1.00  0.97   1.04
cond.time                                      0.99  0.96   1.03
cond.`Peri-pandemic Inc.`                      1.25  0.86   1.83
zi.(Intercept)                                 0.21  0.13   0.33
cond.Std.Dev.(Intercept)|centre                2.67  1.85   4.79
```

2.2.14. Diseases of the ear and mastoid process (short label: Ear.mastoid)

```
Family: nbinom2  ( log )
Formula:
m1[, var] ~ `% adults (occup.)` + `% male (occup.)` + `Peri-pandemic time trend` +
    time + `Peri-pandemic Inc.` + (1 | centre) + offset(log(occupancy))
Zero inflation:             ~1
Data: m1

     AIC      BIC   logLik deviance df.resid
   833.6    864.9   -407.8    815.6      230

Random effects:

Conditional model:
 Groups Name        Variance Std.Dev.
 centre (Intercept) 0.002941 0.05423
Number of obs: 239, groups:  centre, 19

Dispersion parameter for nbinom2 family (): 8.89

Conditional model:
                             Estimate Std. Error z value Pr(>|z|)
(Intercept)                -3.3309259  0.7722958  -4.313 1.61e-05 ***
`% adults (occup.)`        -0.1809829  0.1849905  -0.978   0.3279
`% male (occup.)`           0.0354757  0.1533557   0.231   0.8171
`Peri-pandemic time trend` -0.0009758  0.0212938  -0.046   0.9634
time                       -0.0113137  0.0211378  -0.535   0.5925
`Peri-pandemic Inc.`        0.4948346  0.2238581   2.210   0.0271 *
---
Signif. codes:  0 '***' 0.001 '**' 0.01 '*' 0.05 '.' 0.1 ' ' 1

Zero-inflation model:
            Estimate Std. Error z value Pr(>|z|)
(Intercept)  0.07438    0.15365   0.484    0.628
                                incident rate ratio 2.5 %    97.5 %
cond.(Intercept)                               0.04  0.01      0.16
cond.`% adults (occup.)`                       0.83  0.58      1.20
cond.`% male (occup.)`                         1.04  0.77      1.40
cond.`Peri-pandemic time trend`                1.00  0.96      1.04
cond.time                                      0.99  0.95      1.03
cond.`Peri-pandemic Inc.`                      1.64  1.06      2.54
zi.(Intercept)                                 1.08  0.80      1.46
cond.Std.Dev.(Intercept)|centre                1.06  1.00 104766.10
```

2.2.15. Diseases of the eye and adnexa (short label: Eye.adnexa)

```
Family: nbinom2  ( log )
Formula:
m1[, var] ~ `% adults (occup.)` + `% male (occup.)` + `Peri-pandemic time trend` +
    time + `Peri-pandemic Inc.` + (1 | centre) + offset(log(occupancy))
Zero inflation:             ~1
Data: m1

     AIC      BIC   logLik deviance df.resid
  1019.0   1050.3   -500.5   1001.0      230

Random effects:

Conditional model:
 Groups Name        Variance Std.Dev.
 centre (Intercept) 0.07023  0.265
Number of obs: 239, groups:  centre, 19

Dispersion parameter for nbinom2 family (): 10.9

Conditional model:
                            Estimate Std. Error z value Pr(>|z|)
(Intercept)                -4.201494   0.635224  -6.614 3.74e-11 ***
`% adults (occup.)`         0.064243   0.128306   0.501    0.617
`% male (occup.)`          -0.065335   0.104671  -0.624    0.532
`Peri-pandemic time trend` -0.002007   0.019921  -0.101    0.920
time                       -0.006458   0.019627  -0.329    0.742
`Peri-pandemic Inc.`        0.212734   0.197349   1.078    0.281
---
Signif. codes:  0 '***' 0.001 '**' 0.01 '*' 0.05 '.' 0.1 ' ' 1

Zero-inflation model:
            Estimate Std. Error z value Pr(>|z|)
(Intercept)  -0.2664     0.1570  -1.697   0.0897 .
---
Signif. codes:  0 '***' 0.001 '**' 0.01 '*' 0.05 '.' 0.1 ' ' 1
                                incident rate ratio 2.5 % 97.5 %
cond.(Intercept)                               0.01  0.00   0.05
cond.`% adults (occup.)`                       1.07  0.83   1.37
cond.`% male (occup.)`                         0.94  0.76   1.15
cond.`Peri-pandemic time trend`                1.00  0.96   1.04
cond.time                                      0.99  0.96   1.03
cond.`Peri-pandemic Inc.`                      1.24  0.84   1.82
zi.(Intercept)                                 0.77  0.56   1.04
cond.Std.Dev.(Intercept)|centre                1.30  1.09   2.18
```

2.2.16. Pregnancy, childbirth and the puerperium (short label: Pregn.condition)

```
Family: nbinom2  ( log )
Formula:
m1[, var] ~ `% adults (occup.)` + `% male (occup.)` + `Peri-pandemic time trend` +
    time + `Peri-pandemic Inc.` + (1 | centre) + offset(log(occupancy))
Zero inflation:            ~1
Data: m1

     AIC      BIC   logLik deviance df.resid
  1070.5   1101.7   -526.2   1052.5      230

Random effects:

Conditional model:
 Groups Name        Variance Std.Dev.
 centre (Intercept) 5.105    2.259
Number of obs: 239, groups:  centre, 19

Dispersion parameter for nbinom2 family ():   13

Conditional model:
                           Estimate Std. Error z value Pr(>|z|)
(Intercept)                -4.01048    0.78143  -5.132 2.86e-07 ***
`% adults (occup.)`        -0.02323    0.09583  -0.242 0.808478
`% male (occup.)`          -0.07488    0.10354  -0.723 0.469565
`Peri-pandemic time trend`  0.05241    0.01671   3.136 0.001711 **
time                       -0.05680    0.01644  -3.456 0.000549 ***
`Peri-pandemic Inc.`        0.14204    0.16686   0.851 0.394659
---
Signif. codes:  0 '***' 0.001 '**' 0.01 '*' 0.05 '.' 0.1 ' ' 1

Zero-inflation model:
            Estimate Std. Error z value Pr(>|z|)
(Intercept)  -2.9646     0.6929  -4.278 1.88e-05 ***
---
Signif. codes:  0 '***' 0.001 '**' 0.01 '*' 0.05 '.' 0.1 ' ' 1
                                incident rate ratio 2.5 % 97.5 %
cond.(Intercept)                               0.02  0.00   0.08
cond.`% adults (occup.)`                       0.98  0.81   1.18
cond.`% male (occup.)`                         0.93  0.76   1.14
cond.`Peri-pandemic time trend`                1.05  1.02   1.09
cond.time                                      0.94  0.91   0.98
cond.`Peri-pandemic Inc.`                      1.15  0.83   1.60
zi.(Intercept)                                 0.05  0.01   0.20
cond.Std.Dev.(Intercept)|centre                9.58  4.35  32.13
```

2.2.17. Mental and behavioral disorders (short label: Psych.condition)

```
Family: nbinom2  ( log )
Formula:
m1[, var] ~ `% adults (occup.)` + `% male (occup.)` + `Peri-pandemic time trend` +
    time + `Peri-pandemic Inc.` + (1 | centre) + offset(log(occupancy))
Zero inflation:             ~1
Data: m1

     AIC      BIC   logLik deviance df.resid
  1633.7   1665.0   -807.8   1615.7      230

Random effects:

Conditional model:
 Groups Name        Variance Std.Dev.
 centre (Intercept) 0.2138   0.4623
Number of obs: 239, groups:  centre, 19

Dispersion parameter for nbinom2 family (): 6.91

Conditional model:
                            Estimate Std. Error z value Pr(>|z|)
(Intercept)                -3.639119   0.494276  -7.363 1.80e-13 ***
`% adults (occup.)`        -0.023652   0.090409  -0.262    0.794
`% male (occup.)`           0.081296   0.079313   1.025    0.305
`Peri-pandemic time trend`  0.004695   0.015514   0.303    0.762
time                       -0.018415   0.014829  -1.242    0.214
`Peri-pandemic Inc.`        0.666479   0.170602   3.907 9.36e-05 ***
---
Signif. codes:  0 '***' 0.001 '**' 0.01 '*' 0.05 '.' 0.1 ' ' 1

Zero-inflation model:
            Estimate Std. Error z value Pr(>|z|)
(Intercept)  -2.7158     0.3181  -8.538   <2e-16 ***
---
Signif. codes:  0 '***' 0.001 '**' 0.01 '*' 0.05 '.' 0.1 ' ' 1
                                incident rate ratio 2.5 % 97.5 %
cond.(Intercept)                               0.03  0.01   0.07
cond.`% adults (occup.)`                       0.98  0.82   1.17
cond.`% male (occup.)`                         1.08  0.93   1.27
cond.`Peri-pandemic time trend`                1.00  0.97   1.04
cond.time                                      0.98  0.95   1.01
cond.`Peri-pandemic Inc.`                      1.95  1.39   2.72
zi.(Intercept)                                 0.07  0.04   0.12
cond.Std.Dev.(Intercept)|centre                1.59  1.36   2.01
```

2.2.18. Psychoactive drug prescriptions (short label: Psych.prescrip.)

```
Family: nbinom2  ( log )
Formula:
m1[, var] ~ `% adults (occup.)` + `% male (occup.)` + `Peri-pandemic time trend` +
    time + `Peri-pandemic Inc.` + (1 | centre) + offset(log(occupancy))
Zero inflation:             ~1
Data: m1

     AIC      BIC   logLik deviance df.resid
   937.6    968.9   -459.8    919.6      230

Random effects:

Conditional model:
 Groups Name        Variance Std.Dev.
 centre (Intercept) 0.6859   0.8282
Number of obs: 239, groups:  centre, 19

Dispersion parameter for nbinom2 family (): 6.02

Conditional model:
                           Estimate Std. Error z value Pr(>|z|)
(Intercept)                -3.82127    0.95358  -4.007 6.14e-05 ***
`% adults (occup.)`         0.01230    0.18333   0.067 0.946502
`% male (occup.)`          -0.07368    0.16855  -0.437 0.661985
`Peri-pandemic time trend`  0.01316    0.02878   0.457 0.647470
time                       -0.03752    0.02772  -1.354 0.175834
`Peri-pandemic Inc.`        0.95924    0.26170   3.665 0.000247 ***
---
Signif. codes:  0 '***' 0.001 '**' 0.01 '*' 0.05 '.' 0.1 ' ' 1

Zero-inflation model:
            Estimate Std. Error z value Pr(>|z|)
(Intercept)  -0.3696     0.1894  -1.952    0.051 .
---
Signif. codes:  0 '***' 0.001 '**' 0.01 '*' 0.05 '.' 0.1 ' ' 1
                                incident rate ratio 2.5 % 97.5 %
cond.(Intercept)                               0.02  0.00   0.14
cond.`% adults (occup.)`                       1.01  0.71   1.45
cond.`% male (occup.)`                         0.93  0.67   1.29
cond.`Peri-pandemic time trend`                1.01  0.96   1.07
cond.time                                      0.96  0.91   1.02
cond.`Peri-pandemic Inc.`                      2.61  1.56   4.36
zi.(Intercept)                                 0.69  0.48   1.00
cond.Std.Dev.(Intercept)|centre                2.29  1.62   4.12
```

2.2.19. Diseases of the respiratory system (short label: Respiratory syst.)

```
Family: nbinom2  ( log )
Formula:
m1[, var] ~ `% adults (occup.)` + `% male (occup.)` + `Peri-pandemic time trend` +
    time + `Peri-pandemic Inc.` + (1 | centre) + offset(log(occupancy))
Zero inflation:             ~1
Data: m1

     AIC      BIC   logLik deviance df.resid
  1829.7   1861.0   -905.9   1811.7      230

Random effects:

Conditional model:
 Groups Name        Variance Std.Dev.
 centre (Intercept) 0.2022   0.4496
Number of obs: 239, groups:  centre, 19

Dispersion parameter for nbinom2 family (): 5.31

Conditional model:
                           Estimate Std. Error z value Pr(>|z|)
(Intercept)                -2.38987    0.50947  -4.691 2.72e-06 ***
`% adults (occup.)`         0.05190    0.09786   0.530   0.5958
`% male (occup.)`          -0.02997    0.08984  -0.334   0.7387
`Peri-pandemic time trend`  0.04513    0.01493   3.023   0.0025 **
time                       -0.02808    0.01432  -1.960   0.0500 *
`Peri-pandemic Inc.`       -0.71346    0.16645  -4.286 1.82e-05 ***
---
Signif. codes:  0 '***' 0.001 '**' 0.01 '*' 0.05 '.' 0.1 ' ' 1

Zero-inflation model:
            Estimate Std. Error z value Pr(>|z|)
(Intercept)  -2.3500     0.2567  -9.156   <2e-16 ***
---
Signif. codes:  0 '***' 0.001 '**' 0.01 '*' 0.05 '.' 0.1 ' ' 1
                                incident rate ratio 2.5 % 97.5 %
cond.(Intercept)                               0.09  0.03   0.25
cond.`% adults (occup.)`                       1.05  0.87   1.28
cond.`% male (occup.)`                         0.97  0.81   1.16
cond.`Peri-pandemic time trend`                1.05  1.02   1.08
cond.time                                      0.97  0.95   1.00
cond.`Peri-pandemic Inc.`                      0.49  0.35   0.68
zi.(Intercept)                                 0.10  0.06   0.16
cond.Std.Dev.(Intercept)|centre                1.57  1.32   2.06
```

2.2.20. Diseases of the genitourinary system (short label: Genitourinary syst.)

```
Family: nbinom2  ( log )
Formula:
m1[, var] ~ `% adults (occup.)` + `% male (occup.)` + `Peri-pandemic time trend` +
    time + `Peri-pandemic Inc.` + (1 | centre) + offset(log(occupancy))
Zero inflation:             ~1
Data: m1

     AIC      BIC   logLik deviance df.resid
  1174.9   1206.2   -578.4   1156.9      230

Random effects:

Conditional model:
 Groups Name        Variance Std.Dev.
 centre (Intercept) 0.3374   0.5809
Number of obs: 239, groups:  centre, 19

Dispersion parameter for nbinom2 family ():   27

Conditional model:
                           Estimate Std. Error z value Pr(>|z|)
(Intercept)                -3.39191    0.46381  -7.313 2.61e-13 ***
`% adults (occup.)`         0.06931    0.09096   0.762 0.446072
`% male (occup.)`          -0.17829    0.08837  -2.018 0.043633 *
`Peri-pandemic time trend`  0.01480    0.01366   1.084 0.278519
time                       -0.02218    0.01353  -1.640 0.101023
`Peri-pandemic Inc.`        0.49160    0.13724   3.582 0.000341 ***
---
Signif. codes:  0 '***' 0.001 '**' 0.01 '*' 0.05 '.' 0.1 ' ' 1

Zero-inflation model:
            Estimate Std. Error z value Pr(>|z|)
(Intercept)  -1.2011     0.1939  -6.195 5.85e-10 ***
---
Signif. codes:  0 '***' 0.001 '**' 0.01 '*' 0.05 '.' 0.1 ' ' 1
                                incident rate ratio 2.5 % 97.5 %
cond.(Intercept)                               0.03  0.01   0.08
cond.`% adults (occup.)`                       1.07  0.90   1.28
cond.`% male (occup.)`                         0.84  0.70   0.99
cond.`Peri-pandemic time trend`                1.01  0.99   1.04
cond.time                                      0.98  0.95   1.00
cond.`Peri-pandemic Inc.`                      1.63  1.25   2.14
zi.(Intercept)                                 0.30  0.21   0.44
cond.Std.Dev.(Intercept)|centre                1.79  1.44   2.52
```

2.2.21. Figure S22: Forestplot of sensitivity analysis 2 on subset 3 showing the effects of the variables Peri-pandemic time trend and Peri-pandemic Incidence (Ref.: Pre-pandemic).

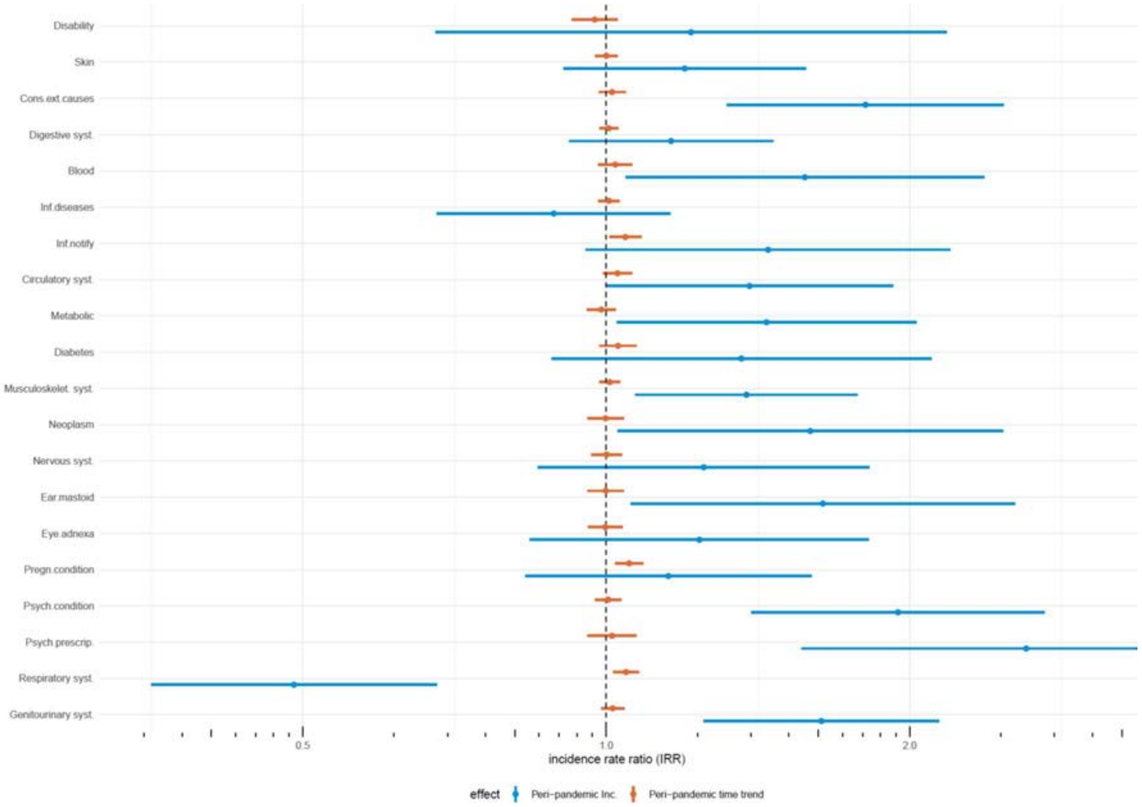

Legend: The incidence rate ratios (IRR) are shown with associated 95% confidence intervals (CI). Estimates are derived for each indicator separately and adjusted for the proportion of males, the proportion of adults, secular trends, and potential influences of the characteristics of refugee centres (random intercept). Y-axis: log-scale. N = 314 centre-months. Source data are provided in the 'source_data.xlsx' file.

2.3. Detailed results for the sensitivity analysis 3

In sensitivity analysis 3, we repeated the main analysis on subset 4, where all available occupancy data was used. The description of subset 4 (see additional excel file Table S3), original R output for each model, as well as a forest plot showing the effects of the variables Peri-pandemic time trend and Peri-pandemic Incidence (Ref.: Pre-pandemic) (Figure S26) are shown below. The model for the variable Diseases of the ear and mastoid process (short label: Ear.mastoid) did not converge, therefore, the model is not shown in sensitivity analysis 3.

2.3.1. Disabilities (short label: Disability)

```
Family: nbinom2  ( log )
Formula:
m1[, var] ~ `% adults (occup.)` + `% male (occup.)` + `Peri-pandemic time trend` +
    time + `Peri-pandemic Inc.` + (1 | centre) + offset(log(occupancy))
Zero inflation:            ~1
Data: m1

     AIC      BIC   logLik deviance df.resid
  1538.8   1574.3   -760.4   1520.8      369

Random effects:

Conditional model:
 Groups Name        Variance Std.Dev.
 centre (Intercept) 1.718    1.311
Number of obs: 378, groups:  centre, 21

Dispersion parameter for nbinom2 family (): 3.13

Conditional model:
                           Estimate Std. Error z value Pr(>|z|)
(Intercept)                -5.70333    0.79171  -7.204 5.85e-13 ***
`% adults (occup.)`        -0.36569    0.12929  -2.828  0.00468 **
`% male (occup.)`           0.52036    0.12626   4.121 3.77e-05 ***
`Peri-pandemic time trend` -0.02650    0.02200  -1.204  0.22851
time                       -0.00391    0.02095  -0.187  0.85195
`Peri-pandemic Inc.`        0.78047    0.25805   3.024  0.00249 **
---
Signif. codes:  0 '***' 0.001 '**' 0.01 '*' 0.05 '.' 0.1 ' ' 1

Zero-inflation model:
            Estimate Std. Error z value Pr(>|z|)
(Intercept)  -0.6574     0.1820  -3.613 0.000303 ***
---
Signif. codes:  0 '***' 0.001 '**' 0.01 '*' 0.05 '.' 0.1 ' ' 1
                                incident rate ratio 2.5 % 97.5 %
cond.(Intercept)                               0.00  0.00   0.02
cond.`% adults (occup.)`                       0.69  0.54   0.89
cond.`% male (occup.)`                         1.68  1.31   2.16
cond.`Peri-pandemic time trend`                0.97  0.93   1.02
cond.time                                      1.00  0.96   1.04
cond.`Peri-pandemic Inc.`                      2.18  1.32   3.62
zi.(Intercept)                                 0.52  0.36   0.74
cond.Std.Dev.(Intercept)|centre                3.71  2.35   7.46
```

2.3.2. Injury, poisoning and certain other consequences of external causes (short label: Cons.ext.causes)

```
Family: nbinom2  ( log )
Formula:
m1[, var] ~ `% adults (occup.)` + `% male (occup.)` + `Peri-pandemic time trend` +
    time + `Peri-pandemic Inc.` + (1 | centre) + offset(log(occupancy))
Zero inflation:             ~1
Data: m1

     AIC      BIC   logLik deviance df.resid
  2177.7   2213.1  -1079.8   2159.7      369

Random effects:

Conditional model:
 Groups Name        Variance Std.Dev.
 centre (Intercept) 0.3899   0.6244
Number of obs: 378, groups:  centre, 21

Dispersion parameter for nbinom2 family (): 6.65

Conditional model:
                            Estimate Std. Error z value Pr(>|z|)
(Intercept)                -3.676021   0.439331  -8.367  < 2e-16 ***
`% adults (occup.)`        -0.140289   0.069403  -2.021  0.04324 *
`% male (occup.)`           0.173124   0.062467   2.771  0.00558 **
`Peri-pandemic time trend` -0.001789   0.013872  -0.129  0.89739
time                       -0.020623   0.013491  -1.529  0.12637
`Peri-pandemic Inc.`        0.649998   0.145814   4.458 8.28e-06 ***
---
Signif. codes:  0 '***' 0.001 '**' 0.01 '*' 0.05 '.' 0.1 ' ' 1

Zero-inflation model:
            Estimate Std. Error z value Pr(>|z|)
(Intercept)  -1.6778     0.1902  -8.821   <2e-16 ***
---
Signif. codes:  0 '***' 0.001 '**' 0.01 '*' 0.05 '.' 0.1 ' ' 1
                                incident rate ratio 2.5 % 97.5 %
cond.(Intercept)                               0.03  0.01   0.06
cond.`% adults (occup.)`                       0.87  0.76   1.00
cond.`% male (occup.)`                         1.19  1.05   1.34
cond.`Peri-pandemic time trend`                1.00  0.97   1.03
cond.time                                      0.98  0.95   1.01
cond.`Peri-pandemic Inc.`                      1.92  1.44   2.55
zi.(Intercept)                                 0.19  0.13   0.27
cond.Std.Dev.(Intercept)|centre                1.87  1.52   2.55
```

2.3.3. Diseases of the skin and subcutaneous tissue (short label: Skin)

```
Family: nbinom2  ( log )
Formula:
m1[, var] ~ `% adults (occup.)` + `% male (occup.)` + `Peri-pandemic time trend` +
    time + `Peri-pandemic Inc.` + (1 | centre) + offset(log(occupancy))
Zero inflation:             ~1
Data: m1

     AIC      BIC   logLik deviance df.resid
  2236.9   2272.4  -1109.5   2218.9      369

Random effects:

Conditional model:
 Groups Name        Variance Std.Dev.
 centre (Intercept) 0.404    0.6356
Number of obs: 378, groups:  centre, 21

Dispersion parameter for nbinom2 family (): 9.08

Conditional model:
                            Estimate Std. Error z value Pr(>|z|)
(Intercept)                -4.196910   0.387137 -10.841  < 2e-16 ***
`% adults (occup.)`        -0.064414   0.059742  -1.078  0.28094
`% male (occup.)`           0.158142   0.055818   2.833  0.00461 **
`Peri-pandemic time trend` -0.016152   0.011525  -1.402  0.16106
time                        0.003722   0.011163   0.333  0.73885
`Peri-pandemic Inc.`        0.225897   0.121819   1.854  0.06369 .
---
Signif. codes:  0 '***' 0.001 '**' 0.01 '*' 0.05 '.' 0.1 ' ' 1

Zero-inflation model:
            Estimate Std. Error z value Pr(>|z|)
(Intercept)  -1.9732     0.1938  -10.18   <2e-16 ***
---
Signif. codes:  0 '***' 0.001 '**' 0.01 '*' 0.05 '.' 0.1 ' ' 1
                                incident rate ratio 2.5 % 97.5 %
cond.(Intercept)                               0.02  0.01   0.03
cond.`% adults (occup.)`                       0.94  0.83   1.05
cond.`% male (occup.)`                         1.17  1.05   1.31
cond.`Peri-pandemic time trend`                0.98  0.96   1.01
cond.time                                      1.00  0.98   1.03
cond.`Peri-pandemic Inc.`                      1.25  0.99   1.59
zi.(Intercept)                                 0.14  0.10   0.20
cond.Std.Dev.(Intercept)|centre                1.89  1.55   2.52
```

2.3.4. Diseases of the digestive system (short label: Digestive syst.)

```
Family: nbinom2  ( log )
Formula:
m1[, var] ~ `% adults (occup.)` + `% male (occup.)` + `Peri-pandemic time trend` +
    time + `Peri-pandemic Inc.` + (1 | centre) + offset(log(occupancy))
Zero inflation:             ~1
Data: m1

     AIC      BIC   logLik deviance df.resid
  2677.0   2712.4  -1329.5   2659.0      369

Random effects:

Conditional model:
 Groups Name        Variance Std.Dev.
 centre (Intercept) 0.3875   0.6225
Number of obs: 378, groups:  centre, 21

Dispersion parameter for nbinom2 family (): 6.52

Conditional model:
                            Estimate Std. Error z value Pr(>|z|)
(Intercept)                -2.980369   0.356069  -8.370  < 2e-16 ***
`% adults (occup.)`        -0.143691   0.049418  -2.908  0.00364 **
`% male (occup.)`           0.155081   0.049484   3.134  0.00172 **
`Peri-pandemic time trend` -0.010046   0.011078  -0.907  0.36449
time                       -0.003621   0.010676  -0.339  0.73450
`Peri-pandemic Inc.`        0.368743   0.119961   3.074  0.00211 **
---
Signif. codes:  0 '***' 0.001 '**' 0.01 '*' 0.05 '.' 0.1 ' ' 1

Zero-inflation model:
            Estimate Std. Error z value Pr(>|z|)
(Intercept)  -3.7903     0.4933  -7.684 1.54e-14 ***
---
Signif. codes:  0 '***' 0.001 '**' 0.01 '*' 0.05 '.' 0.1 ' ' 1
                                incident rate ratio 2.5 % 97.5 %
cond.(Intercept)                               0.05  0.03   0.10
cond.`% adults (occup.)`                       0.87  0.79   0.95
cond.`% male (occup.)`                         1.17  1.06   1.29
cond.`Peri-pandemic time trend`                0.99  0.97   1.01
cond.time                                      1.00  0.98   1.02
cond.`Peri-pandemic Inc.`                      1.45  1.14   1.83
zi.(Intercept)                                 0.02  0.01   0.06
cond.Std.Dev.(Intercept)|centre                1.86  1.55   2.41
```

2.3.5. Diseases of the blood and blood-forming organs and certain disorders involving the immune mechanism (short label: Blood)

```
Family: nbinom2  ( log )
Formula:
m1[, var] ~ `% adults (occup.)` + `% male (occup.)` + `Peri-pandemic time trend` +
    time + `Peri-pandemic Inc.` + (1 | centre) + offset(log(occupancy))
Zero inflation:             ~1
Data: m1

     AIC      BIC   logLik deviance df.resid
   967.2   1002.6   -474.6    949.2      369

Random effects:

Conditional model:
 Groups Name        Variance Std.Dev.
 centre (Intercept) 5.318    2.306
Number of obs: 378, groups:  centre, 21

Dispersion parameter for nbinom2 family (): 11.4

Conditional model:
                           Estimate Std. Error z value Pr(>|z|)
(Intercept)                -5.68354    1.00585  -5.650  1.6e-08 ***
`% adults (occup.)`        -0.08862    0.13969  -0.634    0.526
`% male (occup.)`          -0.04234    0.13205  -0.321    0.748
`Peri-pandemic time trend` -0.01842    0.01666  -1.106    0.269
time                       -0.01116    0.01597  -0.698    0.485
`Peri-pandemic Inc.`        0.46793    0.18168   2.576    0.010 *
---
Signif. codes:  0 '***' 0.001 '**' 0.01 '*' 0.05 '.' 0.1 ' ' 1

Zero-inflation model:
            Estimate Std. Error z value Pr(>|z|)
(Intercept)  -0.3919     0.1904  -2.058   0.0396 *
---
Signif. codes:  0 '***' 0.001 '**' 0.01 '*' 0.05 '.' 0.1 ' ' 1
                                incident rate ratio 2.5 % 97.5 %
cond.(Intercept)                              0.00  0.00   0.02
cond.`% adults (occup.)`                      0.92  0.70   1.20
cond.`% male (occup.)`                        0.96  0.74   1.24
cond.`Peri-pandemic time trend`               0.98  0.95   1.01
cond.time                                     0.99  0.96   1.02
cond.`Peri-pandemic Inc.`                     1.60  1.12   2.28
zi.(Intercept)                                0.68  0.47   0.98
cond.Std.Dev.(Intercept)|centre              10.03  3.84  52.17
```

2.3.6. Certain infectious and parasitic diseases (short label: Inf.diseases)

```
Family: nbinom2  ( log )
Formula:
m1[, var] ~ `% adults (occup.)` + `% male (occup.)` + `Peri-pandemic time trend` +
    time + `Peri-pandemic Inc.` + (1 | centre) + offset(log(occupancy))
Zero inflation:             ~1
Data: m1

     AIC      BIC   logLik deviance df.resid
  2781.0   2816.4  -1381.5   2763.0      369

Random effects:

Conditional model:
 Groups Name        Variance Std.Dev.
 centre (Intercept) 0.5502   0.7418
Number of obs: 378, groups:  centre, 21

Dispersion parameter for nbinom2 family (): 4.76

Conditional model:
                            Estimate Std. Error z value Pr(>|z|)
(Intercept)                -3.106706   0.411942  -7.542 4.64e-14 ***
`% adults (occup.)`        -0.106603   0.054196  -1.967  0.04918 *
`% male (occup.)`           0.146743   0.056696   2.588  0.00965 **
`Peri-pandemic time trend`  0.007699   0.012193   0.631  0.52778
time                       -0.007757   0.011701  -0.663  0.50740
`Peri-pandemic Inc.`        0.016434   0.133681   0.123  0.90216
---
Signif. codes:  0 '***' 0.001 '**' 0.01 '*' 0.05 '.' 0.1 ' ' 1

Zero-inflation model:
            Estimate Std. Error z value Pr(>|z|)
(Intercept)   -2.975      0.298  -9.986   <2e-16 ***
---
Signif. codes:  0 '***' 0.001 '**' 0.01 '*' 0.05 '.' 0.1 ' ' 1
                                incident rate ratio 2.5 % 97.5 %
cond.(Intercept)                               0.04  0.02   0.10
cond.`% adults (occup.)`                       0.90  0.81   1.00
cond.`% male (occup.)`                         1.16  1.04   1.29
cond.`Peri-pandemic time trend`                1.01  0.98   1.03
cond.time                                      0.99  0.97   1.02
cond.`Peri-pandemic Inc.`                      1.02  0.78   1.32
zi.(Intercept)                                 0.05  0.03   0.09
cond.Std.Dev.(Intercept)|centre                2.10  1.68   2.90
```

2.3.7. Notifiable infectious diseases (short label: Inf.notify)

```
Family: nbinom2  ( log )
Formula:
m1[, var] ~ `% adults (occup.)` + `% male (occup.)` + `Peri-pandemic time trend` +
    time + `Peri-pandemic Inc.` + (1 | centre) + offset(log(occupancy))
Zero inflation:             ~1
Data: m1

     AIC      BIC   logLik deviance df.resid
  1166.6   1202.0   -574.3   1148.6      369

Random effects:

Conditional model:
 Groups Name        Variance Std.Dev.
 centre (Intercept) 1.278    1.13
Number of obs: 378, groups:  centre, 21

Dispersion parameter for nbinom2 family (): 10.7

Conditional model:
                           Estimate Std. Error z value Pr(>|z|)
(Intercept)                -4.16816    0.79472  -5.245 1.56e-07 ***
`% adults (occup.)`        -0.20708    0.14465  -1.432   0.1523
`% male (occup.)`           0.21340    0.12854   1.660   0.0969 .
`Peri-pandemic time trend`  0.01231    0.01631   0.755   0.4504
time                       -0.03086    0.01588  -1.943   0.0520 .
`Peri-pandemic Inc.`        0.14620    0.20179   0.725   0.4687
---
Signif. codes:  0 '***' 0.001 '**' 0.01 '*' 0.05 '.' 0.1 ' ' 1

Zero-inflation model:
            Estimate Std. Error z value Pr(>|z|)
(Intercept)   0.1068     0.1478   0.722     0.47
                                incident rate ratio 2.5 % 97.5 %
cond.(Intercept)                               0.02  0.00   0.07
cond.`% adults (occup.)`                       0.81  0.61   1.08
cond.`% male (occup.)`                         1.24  0.96   1.59
cond.`Peri-pandemic time trend`                1.01  0.98   1.05
cond.time                                      0.97  0.94   1.00
cond.`Peri-pandemic Inc.`                      1.16  0.78   1.72
zi.(Intercept)                                 1.11  0.83   1.49
cond.Std.Dev.(Intercept)|centre                3.10  2.03   6.07
```

2.3.8. Diseases of the circulatory system (short label: Circulatory syst.)

```
Family: nbinom2  ( log )
Formula:
m1[, var] ~ `% adults (occup.)` + `% male (occup.)` + `Peri-pandemic time trend` +
    time + `Peri-pandemic Inc.` + (1 | centre) + offset(log(occupancy))
Zero inflation:             ~1
Data: m1

     AIC      BIC   logLik deviance df.resid
  2134.5   2169.9  -1058.2   2116.5      369

Random effects:

Conditional model:
 Groups Name        Variance Std.Dev.
 centre (Intercept) 0.2723   0.5219
Number of obs: 378, groups:  centre, 21

Dispersion parameter for nbinom2 family (): 5.09

Conditional model:
                            Estimate Std. Error z value Pr(>|z|)
(Intercept)                -3.934152   0.458827  -8.574   <2e-16 ***
`% adults (occup.)`         0.117963   0.070063   1.684   0.0922 .
`% male (occup.)`          -0.123153   0.067015  -1.838   0.0661 .
`Peri-pandemic time trend` -0.003293   0.015252  -0.216   0.8291
time                       -0.006491   0.014750  -0.440   0.6599
`Peri-pandemic Inc.`        0.355435   0.160476   2.215   0.0268 *
---
Signif. codes:  0 '***' 0.001 '**' 0.01 '*' 0.05 '.' 0.1 ' ' 1

Zero-inflation model:
            Estimate Std. Error z value Pr(>|z|)
(Intercept)  -1.5177     0.1851  -8.202 2.37e-16 ***
---
Signif. codes:  0 '***' 0.001 '**' 0.01 '*' 0.05 '.' 0.1 ' ' 1
                                incident rate ratio 2.5 % 97.5 %
cond.(Intercept)                               0.02  0.01   0.05
cond.`% adults (occup.)`                       1.13  0.98   1.29
cond.`% male (occup.)`                         0.88  0.78   1.01
cond.`Peri-pandemic time trend`                1.00  0.97   1.03
cond.time                                      0.99  0.97   1.02
cond.`Peri-pandemic Inc.`                      1.43  1.04   1.95
zi.(Intercept)                                 0.22  0.15   0.32
cond.Std.Dev.(Intercept)|centre                1.69  1.43   2.13
```

2.3.9. Hypertension (short label: Hypertension)

```
Family: nbinom2  ( log )
Formula:
m1[, var] ~ `% adults (occup.)` + `% male (occup.)` + `Peri-pandemic time trend` +
    time + `Peri-pandemic Inc.` + (1 | centre) + offset(log(occupancy))
Zero inflation:             ~1
Data: m1

     AIC      BIC   logLik deviance df.resid
  1671.6   1707.0   -826.8   1653.6      369

Random effects:

Conditional model:
 Groups Name        Variance Std.Dev.
 centre (Intercept) 0.795    0.8916
Number of obs: 378, groups:  centre, 21

Dispersion parameter for nbinom2 family (): 5.03

Conditional model:
                           Estimate Std. Error z value Pr(>|z|)
(Intercept)                -5.10800    0.62270  -8.203 2.35e-16 ***
`% adults (occup.)`         0.17475    0.10140   1.723   0.0848 .
`% male (occup.)`          -0.06586    0.09105  -0.723   0.4694
`Peri-pandemic time trend`  0.03181    0.01803   1.764   0.0777 .
time                       -0.03194    0.01735  -1.841   0.0656 .
`Peri-pandemic Inc.`        0.44797    0.20140   2.224   0.0261 *
---
Signif. codes:  0 '***' 0.001 '**' 0.01 '*' 0.05 '.' 0.1 ' ' 1

Zero-inflation model:
            Estimate Std. Error z value Pr(>|z|)
(Intercept)  -0.4580     0.1406  -3.257  0.00112 **
---
Signif. codes:  0 '***' 0.001 '**' 0.01 '*' 0.05 '.' 0.1 ' ' 1
                                incident rate ratio 2.5 % 97.5 %
cond.(Intercept)                               0.01  0.00   0.02
cond.`% adults (occup.)`                       1.19  0.98   1.45
cond.`% male (occup.)`                         0.94  0.78   1.12
cond.`Peri-pandemic time trend`                1.03  1.00   1.07
cond.time                                      0.97  0.94   1.00
cond.`Peri-pandemic Inc.`                      1.57  1.05   2.32
zi.(Intercept)                                 0.63  0.48   0.83
cond.Std.Dev.(Intercept)|centre                2.44  1.75   4.14
```

2.3.10. Endocrine, nutritional and metabolic diseases (short label: Metabolic)

```
Family: nbinom2  ( log )
Formula:
m1[, var] ~ `% adults (occup.)` + `% male (occup.)` + `Peri-pandemic time trend` +
    time + `Peri-pandemic Inc.` + (1 | centre) + offset(log(occupancy))
Zero inflation:             ~1
Data: m1

     AIC      BIC   logLik deviance df.resid
  1994.8   2030.2   -988.4   1976.8      369

Random effects:

Conditional model:
 Groups Name        Variance Std.Dev.
 centre (Intercept) 0.3215   0.567
Number of obs: 378, groups:  centre, 21

Dispersion parameter for nbinom2 family (): 6.73

Conditional model:
                            Estimate Std. Error z value Pr(>|z|)
(Intercept)                -3.846920   0.468351  -8.214  < 2e-16 ***
`% adults (occup.)`         0.074880   0.073472   1.019 0.308127
`% male (occup.)`          -0.123285   0.069759  -1.767 0.077179 .
`Peri-pandemic time trend` -0.011442   0.014083  -0.812 0.416514
time                       -0.002593   0.013632  -0.190 0.849125
`Peri-pandemic Inc.`        0.529316   0.155892   3.395 0.000685 ***
---
Signif. codes:  0 '***' 0.001 '**' 0.01 '*' 0.05 '.' 0.1 ' ' 1

Zero-inflation model:
            Estimate Std. Error z value Pr(>|z|)
(Intercept)  -1.1627     0.1508  -7.712 1.24e-14 ***
---
Signif. codes:  0 '***' 0.001 '**' 0.01 '*' 0.05 '.' 0.1 ' ' 1
                                incident rate ratio 2.5 % 97.5 %
cond.(Intercept)                               0.02  0.01   0.05
cond.`% adults (occup.)`                       1.08  0.93   1.24
cond.`% male (occup.)`                         0.88  0.77   1.01
cond.`Peri-pandemic time trend`                0.99  0.96   1.02
cond.time                                      1.00  0.97   1.02
cond.`Peri-pandemic Inc.`                      1.70  1.25   2.30
zi.(Intercept)                                 0.31  0.23   0.42
cond.Std.Dev.(Intercept)|centre                1.76  1.45   2.37
```

2.3.11. Diabetes mellitus (short label: Diabetes)

```
Family: nbinom2  ( log )
Formula:
m1[, var] ~ `% adults (occup.)` + `% male (occup.)` + `Peri-pandemic time trend` +
    time + `Peri-pandemic Inc.` + (1 | centre) + offset(log(occupancy))
Zero inflation:            ~1
Data: m1

     AIC      BIC   logLik deviance df.resid
  1403.7   1439.1   -692.8   1385.7      369

Random effects:

Conditional model:
 Groups Name        Variance Std.Dev.
 centre (Intercept) 1.538    1.24
Number of obs: 378, groups:  centre, 21

Dispersion parameter for nbinom2 family (): 6.56

Conditional model:
                           Estimate Std. Error z value Pr(>|z|)
(Intercept)                -5.72453    0.69482  -8.239   <2e-16 ***
`% adults (occup.)`         0.21487    0.11040   1.946   0.0516 .
`% male (occup.)`          -0.15092    0.09843  -1.533   0.1252
`Peri-pandemic time trend`  0.02601    0.01804   1.442   0.1494
time                       -0.02523    0.01742  -1.448   0.1476
`Peri-pandemic Inc.`        0.37904    0.19609   1.933   0.0532 .
---
Signif. codes:  0 '***' 0.001 '**' 0.01 '*' 0.05 '.' 0.1 ' ' 1

Zero-inflation model:
            Estimate Std. Error z value Pr(>|z|)
(Intercept)  -0.8277     0.1929   -4.29 1.78e-05 ***
---
Signif. codes:  0 '***' 0.001 '**' 0.01 '*' 0.05 '.' 0.1 ' ' 1
                                incident rate ratio 2.5 % 97.5 %
cond.(Intercept)                               0.00  0.00   0.01
cond.`% adults (occup.)`                       1.24  1.00   1.54
cond.`% male (occup.)`                         0.86  0.71   1.04
cond.`Peri-pandemic time trend`                1.03  0.99   1.06
cond.time                                      0.98  0.94   1.01
cond.`Peri-pandemic Inc.`                      1.46  0.99   2.15
zi.(Intercept)                                 0.44  0.30   0.64
cond.Std.Dev.(Intercept)|centre                3.46  2.22   6.86
```

2.3.12. Diseases of the musculoskeletal system and connective tissue (short label: Musculoskelet. syst.)

```
Family: nbinom2  ( log )
Formula:
m1[, var] ~ `% adults (occup.)` + `% male (occup.)` + `Peri-pandemic time trend` +
    time + `Peri-pandemic Inc.` + (1 | centre) + offset(log(occupancy))
Zero inflation:             ~1
Data: m1

     AIC      BIC   logLik deviance df.resid
  2427.1   2462.5  -1204.6   2409.1      369

Random effects:

Conditional model:
 Groups Name        Variance Std.Dev.
 centre (Intercept) 0.3461   0.5883
Number of obs: 378, groups:  centre, 21

Dispersion parameter for nbinom2 family (): 9.02

Conditional model:
                           Estimate Std. Error z value Pr(>|z|)
(Intercept)                -3.28573    0.35215  -9.330  < 2e-16 ***
`% adults (occup.)`        -0.10811    0.05312  -2.035 0.041811 *
`% male (occup.)`           0.13804    0.05027   2.746 0.006031 **
`Peri-pandemic time trend` -0.01053    0.01047  -1.006 0.314402
time                       -0.01164    0.01009  -1.154 0.248569
`Peri-pandemic Inc.`        0.37700    0.11190   3.369 0.000754 ***
---
Signif. codes:  0 '***' 0.001 '**' 0.01 '*' 0.05 '.' 0.1 ' ' 1

Zero-inflation model:
            Estimate Std. Error z value Pr(>|z|)
(Intercept)  -2.7304     0.2916  -9.363   <2e-16 ***
---
Signif. codes:  0 '***' 0.001 '**' 0.01 '*' 0.05 '.' 0.1 ' ' 1
                                incident rate ratio 2.5 % 97.5 %
cond.(Intercept)                               0.04  0.02   0.07
cond.`% adults (occup.)`                       0.90  0.81   1.00
cond.`% male (occup.)`                         1.15  1.04   1.27
cond.`Peri-pandemic time trend`                0.99  0.97   1.01
cond.time                                      0.99  0.97   1.01
cond.`Peri-pandemic Inc.`                      1.46  1.17   1.82
zi.(Intercept)                                 0.07  0.04   0.12
cond.Std.Dev.(Intercept)|centre                1.80  1.50   2.35
```

2.3.13. Neoplasms (short label: Neoplasm)

```
Family: nbinom2  ( log )
Formula:
m1[, var] ~ `% adults (occup.)` + `% male (occup.)` + `Peri-pandemic time trend` +
    time + `Peri-pandemic Inc.` + (1 | centre) + offset(log(occupancy))
Zero inflation:             ~1
Data: m1

     AIC      BIC   logLik deviance df.resid
   950.9    986.3   -466.4    932.9      369

Random effects:

Conditional model:
 Groups Name        Variance Std.Dev.
 centre (Intercept) 6.148    2.479
Number of obs: 378, groups:  centre, 21

Dispersion parameter for nbinom2 family (): 17.6

Conditional model:
                            Estimate Std. Error z value Pr(>|z|)
(Intercept)                -7.882397   1.060026  -7.436 1.04e-13 ***
`% adults (occup.)`         0.299245   0.114994   2.602  0.00926 **
`% male (occup.)`          -0.239454   0.116474  -2.056  0.03980 *
`Peri-pandemic time trend` -0.015269   0.015837  -0.964  0.33496
time                        0.007379   0.015242   0.484  0.62829
`Peri-pandemic Inc.`        0.257797   0.176317   1.462  0.14371
---
Signif. codes:  0 '***' 0.001 '**' 0.01 '*' 0.05 '.' 0.1 ' ' 1

Zero-inflation model:
            Estimate Std. Error z value Pr(>|z|)
(Intercept)  -0.1116     0.1799   -0.62    0.535
                                incident rate ratio 2.5 % 97.5 %
cond.(Intercept)                              0.00  0.00   0.00
cond.`% adults (occup.)`                      1.35  1.08   1.69
cond.`% male (occup.)`                        0.79  0.63   0.99
cond.`Peri-pandemic time trend`               0.98  0.95   1.02
cond.time                                     1.01  0.98   1.04
cond.`Peri-pandemic Inc.`                     1.29  0.92   1.83
zi.(Intercept)                                0.89  0.63   1.27
cond.Std.Dev.(Intercept)|centre              11.94  4.04  81.50
```

2.3.14. Diseases of the nervous system (short label: Nervous syst.)

```
Family: nbinom2  ( log )
Formula:
m1[, var] ~ `% adults (occup.)` + `% male (occup.)` + `Peri-pandemic time trend` +
    time + `Peri-pandemic Inc.` + (1 | centre) + offset(log(occupancy))
Zero inflation:            ~1
Data: m1

     AIC      BIC   logLik deviance df.resid
  1929.1   1964.5   -955.5   1911.1      369

Random effects:

Conditional model:
 Groups Name        Variance Std.Dev.
 centre (Intercept) 1.048    1.024
Number of obs: 378, groups:  centre, 21

Dispersion parameter for nbinom2 family (): 6.53

Conditional model:
                            Estimate Std. Error z value Pr(>|z|)
(Intercept)                -4.476232   0.543787  -8.232   <2e-16 ***
`% adults (occup.)`         0.039958   0.078550   0.509    0.611
`% male (occup.)`           0.010071   0.078717   0.128    0.898
`Peri-pandemic time trend` -0.013939   0.014442  -0.965    0.334
time                       -0.002738   0.013871  -0.197    0.844
`Peri-pandemic Inc.`        0.241305   0.153978   1.567    0.117
---
Signif. codes:  0 '***' 0.001 '**' 0.01 '*' 0.05 '.' 0.1 ' ' 1

Zero-inflation model:
            Estimate Std. Error z value Pr(>|z|)
(Intercept)   -1.428      0.169  -8.451   <2e-16 ***
---
Signif. codes:  0 '***' 0.001 '**' 0.01 '*' 0.05 '.' 0.1 ' ' 1
                                incident rate ratio 2.5 % 97.5 %
cond.(Intercept)                               0.01  0.00   0.03
cond.`% adults (occup.)`                       1.04  0.89   1.21
cond.`% male (occup.)`                         1.01  0.87   1.18
cond.`Peri-pandemic time trend`                0.99  0.96   1.01
cond.time                                      1.00  0.97   1.02
cond.`Peri-pandemic Inc.`                      1.27  0.94   1.72
zi.(Intercept)                                 0.24  0.17   0.33
cond.Std.Dev.(Intercept)|centre                2.78  1.97   4.71
```

2.3.15. Diseases of the eye and adnexa (short label: Eye.adnexa)

```
Family: nbinom2  ( log )
Formula:
m1[, var] ~ `% adults (occup.)` + `% male (occup.)` + `Peri-pandemic time trend` +
    time + `Peri-pandemic Inc.` + (1 | centre) + offset(log(occupancy))
Zero inflation:             ~1
Data: m1

     AIC      BIC   logLik deviance df.resid
  1540.3   1575.7   -761.2   1522.3      369

Random effects:

Conditional model:
 Groups Name        Variance Std.Dev.
 centre (Intercept) 0.2066   0.4546
Number of obs: 378, groups:  centre, 21

Dispersion parameter for nbinom2 family (): 9.59

Conditional model:
                            Estimate Std. Error z value Pr(>|z|)
(Intercept)                -3.845342   0.525679  -7.315 2.57e-13 ***
`% adults (occup.)`        -0.044302   0.098946  -0.448  0.65434
`% male (occup.)`           0.017774   0.086638   0.205  0.83745
`Peri-pandemic time trend`  0.006241   0.014943   0.418  0.67619
time                       -0.023924   0.014498  -1.650  0.09890 .
`Peri-pandemic Inc.`        0.459765   0.161817   2.841  0.00449 **
---
Signif. codes:  0 '***' 0.001 '**' 0.01 '*' 0.05 '.' 0.1 ' ' 1

Zero-inflation model:
            Estimate Std. Error z value Pr(>|z|)
(Intercept)  -0.3438     0.1453  -2.365    0.018 *
---
Signif. codes:  0 '***' 0.001 '**' 0.01 '*' 0.05 '.' 0.1 ' ' 1
                                incident rate ratio 2.5 % 97.5 %
cond.(Intercept)                               0.02  0.01   0.06
cond.`% adults (occup.)`                       0.96  0.79   1.16
cond.`% male (occup.)`                         1.02  0.86   1.21
cond.`Peri-pandemic time trend`                1.01  0.98   1.04
cond.time                                      0.98  0.95   1.00
cond.`Peri-pandemic Inc.`                      1.58  1.15   2.17
zi.(Intercept)                                 0.71  0.53   0.94
cond.Std.Dev.(Intercept)|centre                1.58  1.26   2.47
```

2.3.16. Pregnancy, childbirth and the puerperium (short label: Pregn.condition)

```
Family: nbinom2  ( log )
Formula:
m1[, var] ~ `% adults (occup.)` + `% male (occup.)` + `Peri-pandemic time trend` +
    time + `Peri-pandemic Inc.` + (1 | centre) + offset(log(occupancy))
Zero inflation:            ~1
Data: m1

     AIC      BIC   logLik deviance df.resid
  1894.4   1929.8   -938.2   1876.4      369

Random effects:

Conditional model:
 Groups Name        Variance Std.Dev.
 centre (Intercept) 6.002    2.45
Number of obs: 378, groups:  centre, 21

Dispersion parameter for nbinom2 family (): 9.63

Conditional model:
                           Estimate Std. Error z value Pr(>|z|)
(Intercept)                -4.32405    0.72945  -5.928 3.07e-09 ***
`% adults (occup.)`         0.04555    0.05660   0.805   0.4210
`% male (occup.)`          -0.11342    0.06670  -1.701   0.0890 .
`Peri-pandemic time trend`  0.01463    0.01330   1.100   0.2713
time                       -0.02373    0.01287  -1.843   0.0653 .
`Peri-pandemic Inc.`        0.08860    0.13414   0.661   0.5089
---
Signif. codes:  0 '***' 0.001 '**' 0.01 '*' 0.05 '.' 0.1 ' ' 1

Zero-inflation model:
            Estimate Std. Error z value Pr(>|z|)
(Intercept)  -1.7845     0.1802  -9.905   <2e-16 ***
---
Signif. codes:  0 '***' 0.001 '**' 0.01 '*' 0.05 '.' 0.1 ' ' 1
                                incident rate ratio 2.5 % 97.5 %
cond.(Intercept)                               0.01  0.00   0.06
cond.`% adults (occup.)`                       1.05  0.94   1.17
cond.`% male (occup.)`                         0.89  0.78   1.02
cond.`Peri-pandemic time trend`                1.01  0.99   1.04
cond.time                                      0.98  0.95   1.00
cond.`Peri-pandemic Inc.`                      1.09  0.84   1.42
zi.(Intercept)                                 0.17  0.12   0.24
cond.Std.Dev.(Intercept)|centre               11.59  5.03  41.07
```

2.3.17. Mental and behavioural disorders (short label: Psych.condition)

```
Family: nbinom2  ( log )
Formula:
m1[, var] ~ `% adults (occup.)` + `% male (occup.)` + `Peri-pandemic time trend` +
    time + `Peri-pandemic Inc.` + (1 | centre) + offset(log(occupancy))
Zero inflation:             ~1
Data: m1

     AIC      BIC   logLik deviance df.resid
  2745.9   2781.3  -1363.9   2727.9      369

Random effects:

Conditional model:
 Groups Name        Variance Std.Dev.
 centre (Intercept) 0.258    0.5079
Number of obs: 378, groups:  centre, 21

Dispersion parameter for nbinom2 family (): 3.58

Conditional model:
                           Estimate Std. Error z value Pr(>|z|)
(Intercept)                -4.00123    0.44899  -8.912  < 2e-16 ***
`% adults (occup.)`         0.05084    0.06523   0.779    0.436
`% male (occup.)`           0.06014    0.06210   0.968    0.333
`Peri-pandemic time trend` -0.01766    0.01446  -1.222    0.222
time                       -0.00343    0.01386  -0.247    0.805
`Peri-pandemic Inc.`        0.69664    0.15938   4.371 1.24e-05 ***
---
Signif. codes:  0 '***' 0.001 '**' 0.01 '*' 0.05 '.' 0.1 ' ' 1

Zero-inflation model:
            Estimate Std. Error z value Pr(>|z|)
(Intercept)  -3.0016     0.3086  -9.727   <2e-16 ***
---
Signif. codes:  0 '***' 0.001 '**' 0.01 '*' 0.05 '.' 0.1 ' ' 1
                                incident rate ratio 2.5 % 97.5 %
cond.(Intercept)                               0.02  0.01   0.04
cond.`% adults (occup.)`                       1.05  0.93   1.20
cond.`% male (occup.)`                         1.06  0.94   1.20
cond.`Peri-pandemic time trend`                0.98  0.96   1.01
cond.time                                      1.00  0.97   1.02
cond.`Peri-pandemic Inc.`                      2.01  1.47   2.74
zi.(Intercept)                                 0.05  0.03   0.09
cond.Std.Dev.(Intercept)|centre                1.66  1.42   2.08
```

2.3.18. Psychoactive drug prescriptions (short label: Psych.prescrip.)

```
Family: nbinom2  ( log )
Formula:
m1[, var] ~ `% adults (occup.)` + `% male (occup.)` + `Peri-pandemic time trend` +
    time + `Peri-pandemic Inc.` + (1 | centre) + offset(log(occupancy))
Zero inflation:             ~1
Data: m1

     AIC      BIC   logLik deviance df.resid
  1439.6   1475.0   -710.8   1421.6      369

Random effects:

Conditional model:
 Groups Name        Variance Std.Dev.
 centre (Intercept) 0.7462   0.8639
Number of obs: 378, groups:  centre, 21

Dispersion parameter for nbinom2 family (): 5.84

Conditional model:
                            Estimate Std. Error z value Pr(>|z|)
(Intercept)                -4.672674   0.669509  -6.979 2.97e-12 ***
`% adults (occup.)`         0.243560   0.092501   2.633 0.008462 **
`% male (occup.)`          -0.253955   0.095036  -2.672 0.007535 **
`Peri-pandemic time trend` -0.009668   0.018509  -0.522 0.601445
time                       -0.012505   0.017848  -0.701 0.483521
`Peri-pandemic Inc.`        0.626045   0.182993   3.421 0.000624 ***
---
Signif. codes:  0 '***' 0.001 '**' 0.01 '*' 0.05 '.' 0.1 ' ' 1

Zero-inflation model:
            Estimate Std. Error z value Pr(>|z|)
(Intercept)  -0.5191     0.1571  -3.304 0.000954 ***
---
Signif. codes:  0 '***' 0.001 '**' 0.01 '*' 0.05 '.' 0.1 ' ' 1
                                incident rate ratio 2.5 % 97.5 %
cond.(Intercept)                               0.01  0.00   0.03
cond.`% adults (occup.)`                       1.28  1.06   1.53
cond.`% male (occup.)`                         0.78  0.64   0.93
cond.`Peri-pandemic time trend`                0.99  0.96   1.03
cond.time                                      0.99  0.95   1.02
cond.`Peri-pandemic Inc.`                      1.87  1.31   2.68
zi.(Intercept)                                 0.60  0.44   0.81
cond.Std.Dev.(Intercept)|centre                2.37  1.74   3.86
```

2.3.19. Diseases of the respiratory system (short label: Respiratory syst.)

```
Family: nbinom2  ( log )
Formula:
m1[, var] ~ `% adults (occup.)` + `% male (occup.)` + `Peri-pandemic time trend` +
    time + `Peri-pandemic Inc.` + (1 | centre) + offset(log(occupancy))
Zero inflation:             ~1
Data: m1

     AIC      BIC   logLik deviance df.resid
  2951.3   2986.8  -1466.7   2933.3      369

Random effects:

Conditional model:
 Groups Name        Variance Std.Dev.
 centre (Intercept) 0.2836   0.5326
Number of obs: 378, groups:  centre, 21

Dispersion parameter for nbinom2 family (): 3.59

Conditional model:
                           Estimate Std. Error z value Pr(>|z|)
(Intercept)                -2.74161    0.43221  -6.343 2.25e-10 ***
`% adults (occup.)`         0.04600    0.06015   0.765 0.444488
`% male (occup.)`           0.03137    0.06063   0.517 0.604857
`Peri-pandemic time trend`  0.02535    0.01321   1.918 0.055095 .
time                       -0.01963    0.01255  -1.564 0.117814
`Peri-pandemic Inc.`       -0.53419    0.14319  -3.731 0.000191 ***
---
Signif. codes:  0 '***' 0.001 '**' 0.01 '*' 0.05 '.' 0.1 ' ' 1

Zero-inflation model:
            Estimate Std. Error z value Pr(>|z|)
(Intercept)  -2.5598     0.2439   -10.5   <2e-16 ***
---
Signif. codes:  0 '***' 0.001 '**' 0.01 '*' 0.05 '.' 0.1 ' ' 1
                                incident rate ratio 2.5 % 97.5 %
cond.(Intercept)                               0.06  0.03   0.15
cond.`% adults (occup.)`                       1.05  0.93   1.18
cond.`% male (occup.)`                         1.03  0.92   1.16
cond.`Peri-pandemic time trend`                1.03  1.00   1.05
cond.time                                      0.98  0.96   1.00
cond.`Peri-pandemic Inc.`                      0.59  0.44   0.78
zi.(Intercept)                                 0.08  0.05   0.12
cond.Std.Dev.(Intercept)|centre                1.70  1.43   2.22
```

2.3.20. Diseases of the genitourinary system (short label: Genitourinary syst.)

```
Family: nbinom2  ( log )
Formula:
m1[, var] ~ `% adults (occup.)` + `% male (occup.)` + `Peri-pandemic time trend` +
    time + `Peri-pandemic Inc.` + (1 | centre) + offset(log(occupancy))
Zero inflation:             ~1
Data: m1

     AIC      BIC   logLik deviance df.resid
  1966.9   2002.3   -974.4   1948.9      369

Random effects:

Conditional model:
 Groups Name        Variance Std.Dev.
 centre (Intercept) 0.7593   0.8714
Number of obs: 378, groups:  centre, 21

Dispersion parameter for nbinom2 family (): 11.6

Conditional model:
                           Estimate Std. Error z value Pr(>|z|)
(Intercept)                -3.30309    0.42879  -7.703 1.33e-14 ***
`% adults (occup.)`         0.04888    0.05490   0.890  0.37320
`% male (occup.)`          -0.15244    0.05767  -2.643  0.00821 **
`Peri-pandemic time trend`  0.01005    0.01230   0.817  0.41411
time                       -0.02491    0.01192  -2.090  0.03660 *
`Peri-pandemic Inc.`        0.54133    0.12672   4.272 1.94e-05 ***
---
Signif. codes:  0 '***' 0.001 '**' 0.01 '*' 0.05 '.' 0.1 ' ' 1

Zero-inflation model:
            Estimate Std. Error z value Pr(>|z|)
(Intercept)  -1.5689     0.1755  -8.941   <2e-16 ***
---
Signif. codes:  0 '***' 0.001 '**' 0.01 '*' 0.05 '.' 0.1 ' ' 1
                                incident rate ratio 2.5 % 97.5 %
cond.(Intercept)                               0.04  0.02   0.09
cond.`% adults (occup.)`                       1.05  0.94   1.17
cond.`% male (occup.)`                         0.86  0.77   0.96
cond.`Peri-pandemic time trend`                1.01  0.99   1.03
cond.time                                      0.98  0.95   1.00
cond.`Peri-pandemic Inc.`                      1.72  1.34   2.20
zi.(Intercept)                                 0.21  0.15   0.29
cond.Std.Dev.(Intercept)|centre                2.39  1.78   3.72
```

2.3.21. Figure S23: Forestplot of sensitivity analysis 3 on subset 4 showing the effects of the variables Peri-pandemic time trend and Peri-pandemic Incidence (Ref.: Pre-pandemic).

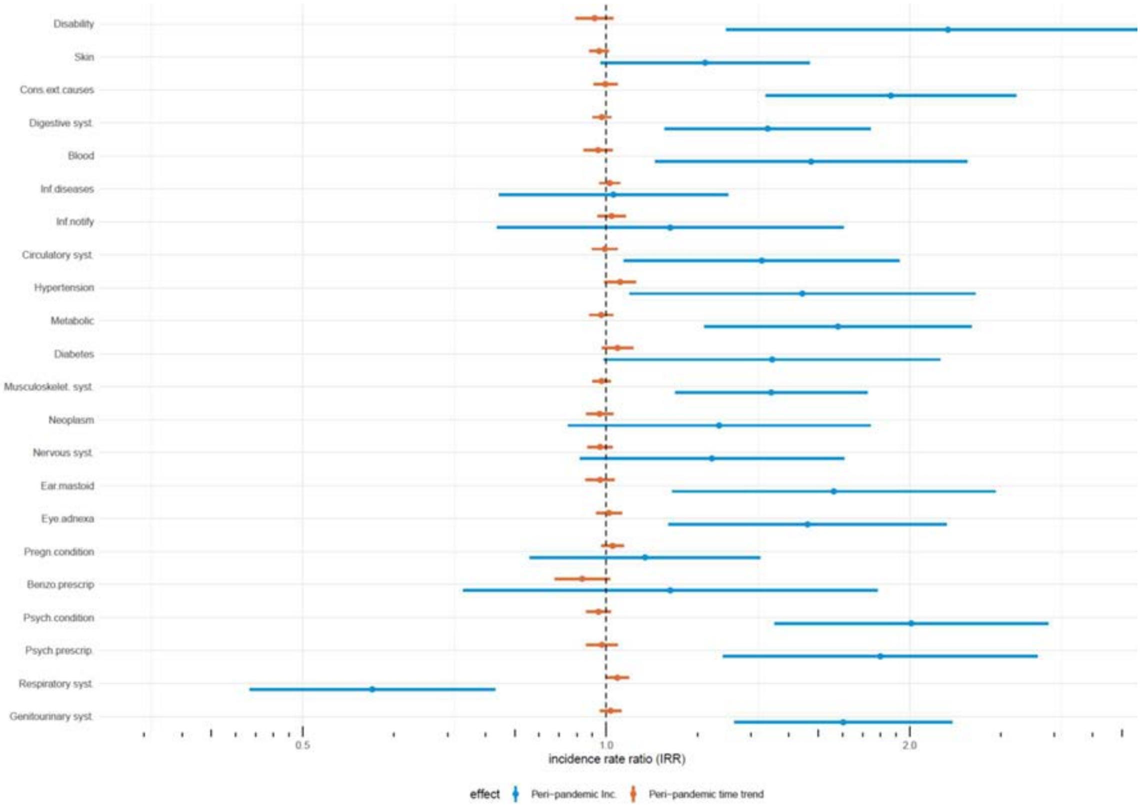

Legend: The incidence rate ratios (IRR) are shown with associated 95% confidence intervals (CI). Estimates are derived for each indicator separately and adjusted for the proportion of males, the proportion of adults, secular trends, and potential influences of the characteristics of refugee centres (random intercept). Y-axis: log-scale. N =378 centre-months. Source data are provided in the 'source_data.xlsx' file.

3. Availability of contributed surveillance data per centre and months

3.1 Figure S24: Data availability per centres and months.

	2018			2019												2020												2021												2022												2023				Months contributed to each dataset
ID	10	11	12	1	2	3	4	5	6	7	8	9	10	11	12	1	2	3	4	5	6	7	8	9	10	11	12	1	2	3	4	5	6	7	8	9	10	11	12	1	2	3	4	5	6	7	8	9	10	11	12	1	2	3	4	
C5	1	1	1	1	1	1	1	1	1	1	1	1	1	1	1	1	1	1	1	1	1	1	1	1	1	1	1	1	1	1	1	1	1	1	1	1	1	1	1	1	1	1	1	1	1	1	1	1	1	1	1	1	1	1	1	55
	4	4	4	4	4	4	4	4	4	4	4	4	4	4	4	0	0	0	0	0	0	4	0	4	4	0	0	4	4	0	0	0	0	0	4	0	4	0	0	0	4	0	0	0	0	0	0	0	0	0	0	0	0	4	4	25
C8	1	1	1	1	1	1	1	1	1	1	1	1	1	1	1	1	1	1	1	1	1	1	1	1	1	1	1	1	1	1	1	1	1	1	1	1	1	1	1	1	1	1	1	1	1	1	1	1	1	1	1	1	1	1	1	55
	4	4	4	4	4	4	4	4	4	4	4	4	4	4	4	4	4	4	4	4	4	4	4	4	4	4	4	4	4	4	4	4	4	4	4	4	4	4	4	4	4	4	4	4	4	0	4	4	4	4	4	4	4	4	4	54
C24	1	1	1	1	1	1	1	1	1	1	1	1	1	1	1	1	1	1	1	1	1	0	0	0	0	0	0	0	0	0	0	0	0	0	0	0	0	0	0	0	0	0	0	0	0	0	0	0	0	0	0	0	0	0	0	21
	0	4	4	4	4	0	0	0	4	4	4	4	0	4	4	4	0	4	4	4	4	0	0	0	0	0	0	0	0	0	0	0	0	0	0	0	0	0	0	0	0	0	0	0	0	0	0	0	0	0	0	0	0	0	0	15
C4	1	1	1	1	1	1	1	1	1	1	1	1	1	0	0	0	0	0	0	0	0	0	0	0	0	0	0	0	0	0	0	0	0	0	0	0	0	0	0	0	0	0	0	0	0	0	0	0	0	0	0	0	0	0	0	13
	0	4	4	4	4	0	0	0	4	4	4	4	0	4	4	4	0	4	4	4	4	0	0	0	0	0	0	0	0	0	0	0	0	0	0	0	0	0	0	0	0	0	0	0	0	0	0	0	0	0	0	0	0	0	0	15
C16	1	1	1	1	1	1	1	1	1	1	1	1	1	1	1	1	1	1	1	1	1	0	0	0	0	0	0	0	0	0	0	0	0	0	0	0	0	0	0	0	0	0	0	0	0	0	0	0	0	0	0	0	0	0	0	21
	0	4	4	4	4	0	0	0	4	4	4	4	0	4	4	4	0	4	4	4	4	0	0	0	0	0	0	0	0	0	0	0	0	0	0	0	0	0	0	0	0	0	0	0	0	0	0	0	0	0	0	0	0	0	0	15
C13	1	1	1	1	1	1	1	1	1	1	1	1	0	0	0	0	0	0	0	0	0	0	0	0	0	0	0	0	0	0	0	0	0	0	0	0	0	0	0	0	0	0	0	0	0	0	0	0	0	0	0	0	0	0	0	12
	4	4	4	4	4	4	4	4	4	4	4	4	4	4	4	4	4	4	4	4	0	0	0	0	0	0	0	0	0	0	0	0	0	0	0	0	0	0	0	0	0	0	0	0	0	0	0	0	0	0	0	0	0	0	0	20
C9	1	1	1	1	1	1	1	1	1	1	1	1	1	1	1	0	0	0	0	0	0	0	0	0	0	0	0	0	0	0	0	0	0	0	0	0	0	0	0	0	0	0	0	0	0	0	0	0	0	0	0	0	0	0	0	15
	0	4	4	4	4	4	4	4	4	4	4	4	4	4	4	4	0	0	0	0	0	0	0	0	0	0	0	0	0	0	0	0	0	0	0	0	0	0	0	0	0	0	0	0	0	0	0	0	0	0	0	0	0	0	0	15
C20	1	1	1	1	1	1	1	1	1	1	1	1	1	1	1	1	1	1	1	1	1	1	1	1	1	1	1	1	1	1	1	1	1	1	1	1	1	1	1	1	1	1	1	1	1	1	1	1	1	1	1	1	1	1	1	55
	0	0	0	0	0	0	0	0	0	0	0	0	0	0	0	0	0	0	0	0	0	0	0	0	0	0	0	0	0	0	0	0	0	0	0	0	0	0	0	0	0	0	0	0	0	0	0	0	0	0	0	0	0	0	0	0
C25	0	0	1	1	1	1	1	1	1	1	1	1	1	1	1	1	1	1	1	1	1	1	1	1	1	1	1	1	1	1	1	1	1	1	1	1	1	1	1	1	1	1	1	1	1	1	1	1	1	1	1	1	1	1	1	53
	0	0	0	4	4	4	4	4	4	4	4	0	4	4	0	0	0	0	0	0	4	4	0	4	0	0	0	0	4	0	4	4	4	4	4	4	4	0	4	4	4	4	4	4	4	4	4	4	4	4	4	4	4	4	4	38
C3	0	0	0	0	0	0	1	1	1	1	1	1	1	1	1	1	1	1	1	1	1	1	1	1	1	1	1	1	1	1	1	1	1	1	1	1	1	1	1	1	1	1	1	1	1	1	1	1	1	1	1	1	1	1	1	49
	0	0	0	0	0	0	0	0	0	0	0	0	4	0	0	0	4	0	4	4	4	0	0	0	0	4	0	4	4	4	4	4	4	4	4	4	4	4	4	0	4	4	4	0	4	0	4	0	0	4	0	0	0	0	0	24
C2	0	0	0	0	0	0	0	0	1	1	1	1	1	1	1	1	1	1	1	1	1	1	1	1	1	1	1	1	1	1	1	1	1	1	1	1	1	1	1	1	1	1	1	1	1	1	1	1	1	1	1	1	1	1	1	47
	0	0	0	0	0	0	0	0	0	0	0	0	4	4	4	4	4	4	4	4	4	4	0	4	4	4	0	4	4	4	4	4	4	4	4	4	4	4	4	0	4	4	0	0	4	4	4	4	4	0	4	4	0	4	0	35
C1	0	0	0	0	0	0	0	0	0	1	0	0	1	1	1	1	1	1	1	1	1	1	1	1	1	1	1	1	1	1	1	1	1	1	1	1	1	1	1	1	1	1	1	1	1	1	1	1	1	1	1	1	1	1	1	44
	0	0	0	0	0	0	0	0	0	0	0	0	4	0	4	4	4	0	4	4	4	4	0	4	0	4	0	4	4	0	0	4	0	0	0	0	0	4	0	4	0	4	4	0	4	4	4	4	4	4	4	4	4	4	4	28
C11	0	0	0	0	0	0	0	0	0	0	0	1	1	1	1	1	1	1	1	1	1	1	1	1	1	1	1	1	1	1	1	1	1	1	1	1	1	1	1	1	1	1	1	1	1	1	1	1	1	1	1	1	1	1	1	44
	0	0	0	0	0	0	0	0	0	0	0	0	0	4	0	0	0	0	0	0	0	0	0	0	0	0	0	0	0	0	0	0	0	0	4	0	0	0	0	0	0	0	0	0	0	0	0	0	0	0	0	0	0	0	0	2
C7	0	0	0	0	0	0	0	0	0	0	0	1	1	1	1	1	1	1	1	1	1	1	1	1	1	1	1	1	1	1	1	1	1	1	1	1	1	1	1	1	1	1	1	1	1	1	1	1	1	1	1	1	1	1	1	44
	0	0	0	0	0	0	0	0	0	0	0	0	0	4	0	0	4	0	0	0	0	0	0	0	0	4	0	0	0	0	0	0	0	4	4	0	0	0	0	4	0	0	0	0	0	0	0	0	0	0	0	0	0	4	0	7
C15	0	0	0	0	0	0	0	0	0	0	0	1	1	1	1	1	1	1	0	0	0	0	0	0	0	0	0	0	0	0	0	0	0	0	0	0	0	0	1	0	1	1	0	0	0	0	0	1	1	1	1	1	1	1	1	18
	0	0	0	0	0	0	0	0	0	0	0	0	0	4	0	0	0	0	0	0	0	0	0	0	0	0	0	0	0	0	0	0	0	0	0	0	0	0	0	0	0	0	0	0	0	0	0	0	0	0	0	0	0	0	0	1
C18	0	0	0	0	0	0	0	0	0	0	0	0	1	1	1	1	1	1	0	1	1	1	0	0	0	0	1	1	1	1	1	1	1	1	1	1	1	1	1	1	1	1	1	1	1	1	1	1	1	1	1	1	1	1	1	38
	0	0	0	0	0	0	0	0	0	0	0	0	4	0	4	0	4	4	0	0	0	0	0	0	0	0	0	0	0	0	0	0	0	0	0	0	0	0	0	0	0	0	0	0	0	0	0	0	0	0	0	0	0	0	0	4
C23*	0	0	0	0	0	0	0	0	0	0	0	0	0	0	0	1	1	1	1	1	1	1	1	1	1	1	1	1	1	1	1	1	1	1	1	1	1	1	1	1	1	1	1	0	0	0	0	0	0	0	0	0	0	0	0	28
	0	0	0	0	0	0	0	0	0	0	0	0	0	0	0	0	0	0	0	0	0	0	0	0	0	0	0	0	0	0	0	0	0	0	0	0	0	0	0	0	0	0	0	0	0	0	0	0	0	0	0	0	0	0	0	0
C22	0	0	0	0	0	0	0	0	0	0	0	0	0	0	0	0	0	1	1	1	1	1	1	1	1	1	1	1	1	1	1	1	1	1	1	1	1	1	1	1	1	1	1	1	1	1	1	1	1	1	1	1	1	1	1	38
	0	0	0	0	0	0	0	0	0	0	0	0	0	0	0	0	0	0	0	0	0	0	0	0	0	0	0	0	0	0	0	0	0	0	0	0	0	0	0	0	0	0	0	0	0	0	0	0	0	0	0	0	0	0	0	0
C10	0	0	0	0	0	0	0	0	0	0	0	0	0	0	0	0	0	0	0	0	0	0	0	0	0	0	0	1	1	1	1	1	1	1	1	1	1	1	1	1	1	1	1	1	1	1	1	1	1	1	1	1	1	1	1	28
	0	0	0	0	0	0	0	0	0	0	0	0	0	0	0	0	0	0	0	0	0	0	0	0	0	0	0	0	4	4	4	4	4	4	4	4	4	4	4	4	4	4	4	4	4	0	4	4	4	4	4	4	4	4	4	26
C19	0	0	0	0	0	0	0	0	0	0	0	0	0	0	0	0	0	0	0	0	0	0	0	0	0	0	0	1	1	1	1	1	1	1	1	1	1	1	1	1	1	1	1	1	1	1	1	1	1	1	1	1	1	1	1	28
	0	0	0	0	0	0	0	0	0	0	0	0	0	0	0	0	0	0	0	0	0	0	0	0	0	0	0	0	4	4	4	4	4	4	4	4	4	4	4	4	4	4	4	4	4	0	4	4	4	4	4	4	4	4	4	26
C14	0	0	0	0	0	0	0	0	0	0	0	0	0	0	0	0	0	0	0	0	0	0	0	0	0	0	0	0	1	1	1	1	1	1	1	1	1	1	1	1	1	1	1	0	0	1	1	1	1	1	1	1	1	1	1	25
	0	0	0	0	0	0	0	0	0	0	0	0	0	0	0	0	0	0	0	0	0	0	0	0	0	0	0	0	0	0	0	0	0	0	0	0	0	0	0	0	0	0	0	0	0	0	0	0	0	0	0	0	0	0	0	0
C17	0	0	0	0	0	0	0	0	0	0	0	0	0	0	0	0	0	0	0	0	0	0	0	0	0	0	0	0	0	0	0	0	0	1	1	1	1	1	1	1	1	1	1	1	1	1	1	1	1	1	1	1	1	1	1	22
	0	0	0	0	0	0	0	0	0	0	0	0	0	0	0	0	0	0	0	0	0	0	0	0	0	0	0	0	0	0	0	0	0	0	0	4	4	4	4	4	4	4	0	0	4	4	4	4	4	4	4	4	0	4	4	17
C12	0	0	0	0	0	0	0	0	0	0	0	0	0	0	0	0	0	0	0	0	0	0	0	0	0	0	0	0	0	0	0	0	0	0	1	1	1	1	1	1	1	1	1	1	1	1	1	1	1	1	1	1	1	1	1	21
	0	0	0	0	0	0	0	0	0	0	0	0	0	0	0	0	0	0	0	0	0	0	0	0	0	0	0	0	0	0	0	0	0	0	0	0	4	4	4	4	4	4	4	4	4	1	4	4	4	4	4	4	4	4	4	18.25
C6*	0	0	0	0	0	0	0	0	0	0	0	0	0	0	0	0	0	0	0	0	0	0	0	0	0	0	0	0	0	0	0	0	0	0	1	1	1	1	1	1	1	1	1	0	0	0	0	0	0	0	0	0	0	0	0	9
	0	0	0	0	0	0	0	0	0	0	0	0	0	0	0	0	0	0	0	0	0	0	0	0	0	0	0	0	0	0	0	0	0	0	0	4	4	4	4	4	4	4	4	0	4	4	4	0	0	4	4	4	4	4	4	17
C21	0	0	0	0	0	0	0	0	0	0	0	0	0	0	0	0	0	0	0	0	0	0	0	0	0	0	0	0	0	0	0	0	0	0	0	0	0	0	0	0	0	0	1	1	1	1	1	1	1	1	1	1	1	1	1	13
	0	0	0	0	0	0	0	0	0	0	0	0	0	0	0	0	0	0	0	0	0	0	0	0	0	0	0	0	0	0	0	0	0	0	0	0	0	0	0	0	0	0	0	0	0	4	0	0	0	0	0	0	0	0	0	1

*: centres were part of the network in 04/2023 and health facilities were running, but most current data was provided in 04/2022.

Legend:	
1	Data in patient data (subset 1)
4	Data in occupancy data (subset 4)
0	No data in dataset
0	Centre not part of research network
0	Centre closed

3.2. Figure S25: Availability of contributed surveillance data per centre and months

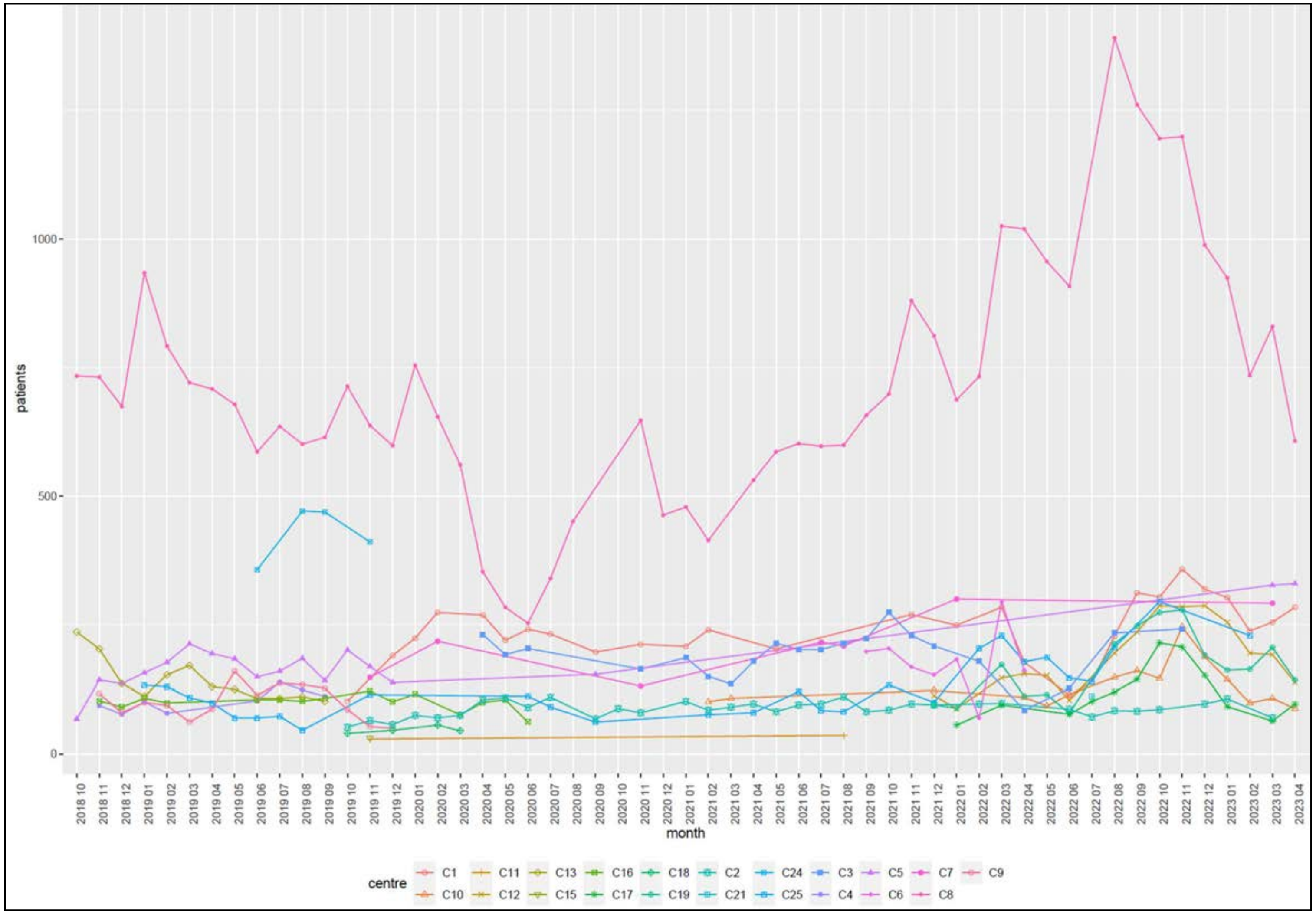

Legend: Available surveillance data as number of patients (Y-axis) per centre and months (X-axis) used in the analysis.

Each individually coloured line and corresponding shape represents a centre. N = 314 centre-months. Source data are provided in the 'source_data.xlsx' file.

3.3 Table: Mean age of patients before and after onset of the COVID-19 pandemic, 2018 – 2023

Centre	Before March 2020	After March 2020	Difference
1	27,8	N.a.	-
2	29,9	N.a.	-
3	31,9	27,0	4,9
4	24,7	24,1	0,6
5	26,8	27,2	-0,5
6	28,1	26,3	1,8
7	N.a.	28,6	-
8	N.a.	28,8	-
9	30,4	28,7	1,7
10	29,7	29,0	0,7
11	N.a.	27,8	-
12	29,3	29,3	0,0
13	28,8	27,7	1,1
14	31,8	30,3	1,5
15	27,0	30,1	-3,1
16	27,0	27,0	0,0
17	26,5	N.a.	-
18	24,3	23,3	1,0
19	N.a.	28,6	-
20	31,0	28,6	2,4
21	27,9	26,0	1,9
22	N.a.	24,3	-
23	N.a.	21,3	-
24	N.a.	27,1	-
25	N.a.	27,3	-
26	29,1	27,8	1,3
Column Mean	28,4	27,2	1,0
Column SD	2,1	2,2	1,7

Legend: N = 215,864 refugee patients

4. Total number of persons residing in Germany for humanitarian regions, by state and country of origin

	31.12.2018						31.12.2019						31.12.2020						31.12.2021						31.12.2022						31.12.2023					
	Baden-Wuerttemberg		Bavaria		Hamburg		Baden-Wuerttemberg		Bavaria		Hamburg		Baden-Wuerttemberg		Bavaria		Hamburg		Baden-Wuerttemberg		Bavaria		Hamburg		Baden-Wuerttemberg		Bavaria		Hamburg		Baden-Wuerttemberg		Bavaria		Hamburg	
	Total	%	Total	%	Total	%	Total	%	Total	%	Total	%	Total	%	Total	%	Total	%	Total	%	Total	%	Total	%	Total	%	Total	%	Total	%	Total	%	Total	%	Total	%
**Total**	202725		212015		52730		210005		213120		55160		210185		213085		51825		216675		219175		55970		362200		379405		91060		369800		384690		94880	
Ukraine	3340	1.65	8700	4.10	765	1.45	3345	1.59	8510	3.99	770	1.40	3270	1.56	7955	3.73	765	1.48	3255	1.50	7910	3.61	765	1.37	131075	36.19	149485	39.39	28645	31.46	124720	33.73	135265	35.16	29640	31.24
Syria	57000	28.12	52420	24.72	10995	20.85	61500	29.29	54155	25.41	11850	21.48	63720	30.32	56650	26.59	11230	21.67	66745	30.80	58625	26.75	11515	20.57	67665	18.68	62490	16.47	12295	13.50	70340	19.02	65535	17.04	12040	12.69
Afghanistan	20470	10.10	29115	13.73	15210	28.85	21010	10.00	28060	13.17	15850	28.73	20765	9.88	27660	12.98	14480	27.94	21880	10.10	30500	13.92	17245	30.81	27875	7.70	36445	9.61	20505	22.52	31600	8.55	43155	11.22	22340	23.55
Iraq	23555	11.62	28310	13.35	3870	7.34	23890	11.38	27990	13.13	3855	6.99	23605	11.23	27865	13.08	3465	6.69	24365	11.24	29015	13.24	3715	6.64	24595	6.79	28665	7.56	3765	4.13	22620	6.12	26530	6.90	3660	3.86
Turkey	9820	4.84	2855	1.35	1360	2.58	11075	5.27	3785	1.78	1450	2.63	11505	5.47	3915	1.84	1560	3.01	12695	5.86	4530	2.07	1625	2.90	18010	4.97	7790	2.05	2000	2.20	28265	7.64	16270	4.23	2700	2.85
Eritrea	6420	3.17	9275	4.37	2200	4.17	6830	3.25	9380	4.40	2435	4.41	6880	3.27	9595	4.50	2180	4.21	6990	3.23	9475	4.32	2325	4.15	7040	1.94	9510	2.51	2605	2.86	7110	1.92	9990	2.60	2740	2.89
Nigeria	8260	4.07	13135	6.20	200	0.38	9680	4.61	13680	6.42	235	0.43	9920	4.72	13475	6.32	245	0.47	9635	4.45	12540	5.72	265	0.47	9230	2.55	11815	3.11	320	0.35	8340	2.26	9820	2.55	295	0.31
Iran	5915	2.92	5565	2.62	4640	8.80	6165	2.94	5560	2.61	4705	8.53	5865	2.79	5505	2.58	4330	8.36	5930	2.74	5355	2.44	4505	8.05	6100	1.68	5670	1.49	4965	5.45	6035	1.63	5820	1.51	5175	5.45
Russian Federation	5075	2.50	7775	3.67	2500	4.74	5085	2.42	7595	3.56	2575	4.67	4925	2.34	7430	3.49	2440	4.71	4835	2.23	7260	3.31	2430	4.34	5375	1.48	8185	2.16	2705	2.97	5815	1.57	9065	2.36	2915	3.07
Cosovo	10250	5.06	4025	1.90	590	1.12	9830	4.68	3930	1.84	565	1.02	9605	4.57	3885	1.82	550	1.06	9385	4.33	3915	1.79	555	0.99	9375	2.59	3925	1.03	530	0.58	9250	2.50	4060	1.06	505	0.53
Somalia	2860	1.41	6250	2.95	835	1.58	3190	1.52	6530	3.06	955	1.73	3300	1.57	6370	2.99	910	1.76	3500	1.62	6585	3.00	1100	1.97	3570	0.99	6600	1.74	1175	1.29	3645	0.99	7400	1.92	1295	1.36
Unknown	4075	2.01	2550	1.20	940	1.78	4515	2.15	2690	1.26	1225	2.22	4845	2.31	2735	1.28	1215	2.34	5070	2.34	2730	1.25	1155	2.06	5145	1.42	2655	0.70	1385	1.52	5210	1.41	2675	0.70	1265	1.33
Gambia	8720	4.30	710	0.33	85	0.16	7890	3.76	770	0.36	85	0.15	7420	3.53	840	0.39	85	0.16	6895	3.18	815	0.37	95	0.17	6465	1.78	815	0.21	100	0.11	4705	1.27	705	0.18	105	0.11
Serbia	4495	2.22	1175	0.55	1015	1.92	4165	1.98	1150	0.54	1030	1.87	3930	1.87	1065	0.50	980	1.89	3795	1.75	1135	0.52	925	1.65	3825	1.06	1120	0.30	915	1.00	3835	1.04	1170	0.30	910	0.96
Ethiopia	530	0.26	6065	2.86	55	0.10	540	0.26	6080	2.85	65	0.12	540	0.26	5925	2.78	60	0.12	520	0.24	5900	2.69	65	0.12	500	0.14	5905	1.56	60	0.07	470	0.13	5160	1.34	60	0.06
Georgia	1430	0.71	715	0.34	100	0.19	1405	0.67	675	0.32	145	0.26	1270	0.60	580	0.27	120	0.23	1395	0.64	810	0.37	100	0.18	2815	0.78	1935	0.51	250	0.27	3355	0.91	2205	0.57	205	0.22
Pakistan	5040	2.49	3740	1.76	210	0.40	4575	2.18	3080	1.45	205	0.37	4095	1.95	2550	1.20	190	0.37	3670	1.69	2030	0.93	185	0.33	3345	0.92	1755	0.46	180	0.20	2775	0.75	1440	0.37	170	0.18
Bosnia and Herzegovina	2005	0.99	1925	0.91	680	1.29	1880	0.90	1890	0.89	675	1.22	1815	0.86	1835	0.86	665	1.28	1940	0.90	1865	0.85	695	1.24	2015	0.56	1850	0.49	640	0.70	1990	0.54	1810	0.47	630	0.66
Stateless	1460	0.72	2180	1.03	145	0.27	1520	0.72	2155	1.01	165	0.30	1495	0.71	2210	1.04	160	0.31	1560	0.72	2400	1.10	185	0.33	1605	0.44	2335	0.62	225	0.25	1600	0.43	2250	0.58	205	0.22
North Macedonia	1335	0.66	290	0.14	555	1.05	1215	0.58	295	0.14	535	0.97	1095	0.52	275	0.13	460	0.89	2090	0.96	515	0.23	590	1.05	2735	0.76	615	0.16	615	0.68	2740	0.74	595	0.15	530	0.56
Aserbaidschan	320	0.16	3485	1.64	165	0.31	305	0.15	3240	1.52	185	0.34	300	0.14	3145	1.48	185	0.36	305	0.14	2835	1.29	195	0.35	500	0.14	3050	0.80	315	0.35	555	0.15	3020	0.79	330	0.35
Vietnam	1045	0.52	2035	0.96	260	0.49	1020	0.49	1970	0.92	265	0.48	980	0.47	1910	0.90	255	0.49	950	0.44	1910	0.87	260	0.46	1115	0.31	2325	0.61	385	0.42	1165	0.32	2355	0.61	360	0.38
Cameroon	2160	1.07	60	0.03	20	0.04	2290	1.09	60	0.03	15	0.03	2265	1.08	55	0.03	20	0.04	2215	1.02	60	0.03	25	0.04	2430	0.67	80	0.02	40	0.04	2730	0.74	65	0.02	35	0.04
Armenia	225	0.11	1825	0.86	405	0.77	245	0.12	1520	0.71	405	0.73	220	0.10	1410	0.66	370	0.71	215	0.10	1305	0.60	375	0.67	345	0.10	1650	0.43	470	0.52	355	0.10	1710	0.44	490	0.52
China	1505	0.74	805	0.38	90	0.17	1580	0.75	755	0.35	95	0.17	1525	0.73	750	0.35	85	0.16	1535	0.71	750	0.34	85	0.15	1570	0.43	780	0.21	80	0.09	1485	0.40	715	0.19	80	0.08
Sri Lanka	2070	1.02	300	0.14	40	0.08	2125	1.01	275	0.13	40	0.07	2115	1.01	275	0.13	40	0.08	2070	0.96	270	0.12	35	0.06	2065	0.57	265	0.07	35	0.04	2065	0.56	255	0.07	35	0.04
Sierra Leone	75	0.04	2095	0.99	50	0.09	85	0.04	2075	0.97	45	0.08	80	0.04	2080	0.98	40	0.08	75	0.03	1950	0.89	40	0.07	75	0.02	1980	0.52	45	0.05	105	0.03	2195	0.57	55	0.06
Yemen	30	0.01	215	0.10	10	0.02	30	0.01	450	0.21	15	0.03	40	0.02	540	0.25	20	0.04	60	0.03	1125	0.51	30	0.05	60	0.02	1605	0.42	45	0.05	80	0.02	2580	0.67	50	0.05
Togo	1360	0.67	475	0.22	325	0.62	1510	0.72	470	0.22	305	0.55	1485	0.71	470	0.22	275	0.53	1455	0.67	450	0.21	260	0.46	1470	0.41	450	0.12	265	0.29	1390	0.38	430	0.11	260	0.27
India	1870	0.92	155	0.07	80	0.15	1700	0.81	150	0.07	80	0.15	1545	0.74	140	0.07	90	0.17	1455	0.67	125	0.06	80	0.14	1800	0.50	185	0.05	90	0.10	1950	0.53	170	0.04	75	0.08
Moldova	470	0.23	860	0.41	100	0.19	455	0.22	1020	0.48	95	0.17	445	0.21	810	0.38	90	0.17	425	0.20	1375	0.63	85	0.15	565	0.16	1560	0.41	110	0.12	605	0.16	1225	0.32	120	0.13
Algeria	1080	0.53	205	0.10	115	0.22	915	0.44	195	0.09	125	0.23	995	0.47	365	0.17	150	0.29	1120	0.52	325	0.15	165	0.29	1245	0.34	460	0.12	195	0.21	1355	0.37	605	0.16	215	0.23
Guinea	1095	0.54	225	0.11	200	0.38	1280	0.61	275	0.13	220	0.40	1205	0.57	275	0.13	240	0.46	1120	0.52	260	0.12	260	0.46	1100	0.30	250	0.07	325	0.36	1425	0.39	280	0.07	410	0.43
Tunisia	380	0.19	120	0.06	35	0.07	395	0.19	110	0.05	40	0.07	395	0.19	115	0.05	45	0.09	540	0.25	120	0.05	45	0.08	1025	0.28	355	0.09	105	0.12	1340	0.36	705	0.18	145	0.15
Congo	290	0.14	975	0.46	20	0.04	280	0.13	1000	0.47	25	0.05	270	0.13	1035	0.49	30	0.06	260	0.12	1045	0.48	35	0.06	295	0.08	1315	0.35	35	0.04	315	0.09	1655	0.43	30	0.03
Palestinian Territories	200	0.10	370	0.17	60	0.11	170	0.08	425	0.20	60	0.11	265	0.13	415	0.19	140	0.27	550	0.25	410	0.19	200	0.36	885	0.24	440	0.12	250	0.27	1210	0.33	435	0.11	280	0.30
Albania	785	0.39	530	0.25	355	0.67	695	0.33	510	0.24	360	0.65	620	0.29	465	0.22	345	0.67	670	0.31	500	0.23	345	0.62	815	0.23	585	0.15	360	0.40	795	0.21	535	0.14	305	0.32
Belarus	225	0.11	620	0.29	65	0.12	230	0.11	645	0.30	70	0.13	220	0.10	640	0.30	70	0.14	225	0.10	705	0.32	75	0.13	325	0.09	980	0.26	95	0.10	300	0.08	1160	0.30	100	0.11
Morocco	285	0.14	150	0.07	75	0.14	280	0.13	145	0.07	65	0.12	350	0.17	195	0.09	65	0.13	410	0.19	260	0.12	95	0.17	590	0.16	545	0.14	215	0.24	780	0.21	635	0.17	170	0.18
Libanon	925	0.46	305	0.14	160	0.30	885	0.42	295	0.14	155	0.28	845	0.40	290	0.14	155	0.30	850	0.39	300	0.14	160	0.29	885	0.24	300	0.08	215	0.24	870	0.24	280	0.07	240	0.25
Ghana	195	0.10	195	0.09	575	1.09	245	0.12	215	0.10	525	0.95	245	0.12	215	0.10	550	1.06	245	0.11	210	0.10	540	0.96	255	0.07	225	0.06	585	0.64	255	0.07	230	0.06	625	0.66
Serbia and Montenegro (05.02.2	595	0.29	960	0.45	5	0.01	555	0.26	945	0.44	5	0.01	505	0.24	895	0.42	5	0.01	455	0.21	815	0.37	5	0.01	420	0.12	745	0.20	5	0.01	395	0.11	710	0.18	5	0.01
Egypt	65	0.03	150	0.07	595	1.13	75	0.04	135	0.06	600	1.09	75	0.04	135	0.06	590	1.14	80	0.04	140	0.06	595	1.06	155	0.04	210	0.06	640	0.70	130	0.04	190	0.05	605	0.64
Serbia (incl. Cosovo) (03.06.200	375	0.18	1065	0.50	5	0.01	330	0.16	1035	0.49	5	0.01	310	0.15	1000	0.47	-	-	275	0.13	905	0.41	5	0.01	250	0.07	835	0.22	-	-	225	0.06	795	0.21	-	-
Cote d'Ivoire	85	0.04	365	0.17	85	0.16	80	0.04	440	0.21	90	0.16	75	0.04	445	0.21	95	0.18	85	0.04	425	0.19	105	0.19	100	0.03	520	0.14	110	0.12	125	0.03	1055	0.27	115	0.12
Jordan	65	0.03	385	0.18	30	0.06	80	0.04	455	0.21	35	0.06	75	0.04	505	0.24	25	0.05	80	0.04	530	0.24	25	0.04	115	0.03	705	0.19	40	0.04	120	0.03	955	0.25	40	0.04
Montenegro (ab 03.06.2006)	290	0.14	125	0.06	410	0.78	285	0.14	125	0.06	430	0.78	265	0.13	120	0.06	390	0.75	280	0.13	120	0.05	380	0.68	290	0.08	130	0.03	395	0.43	295	0.08	140	0.04	380	0.40
Uganda	10	0.00	510	0.24	-	-	10	0.00	555	0.26	-	-	15	0.01	590	0.28	-	-	15	0.01	625	0.29	-	-	20	0.01	725	0.19	-	-	15	0.00	960	0.25	-	-
Kazakhstan	180	0.09	450	0.21	70	0.13	180	0.09	450	0.21	70	0.13	180	0.09	475	0.22	70	0.14	180	0.08	470	0.21	60	0.11	210	0.06	520	0.14	65	0.07	215	0.06	535	0.14	65	0.07
Senegal	100	0.05	1155	0.54	15	0.03	110	0.05	1010	0.47	20	0.04	125	0.06	870	0.41	15	0.03	125	0.06	770	0.35	15	0.03	140	0.04	730	0.19	15	0.02	145	0.04	575	0.15	25	0.03
Usbekistan	295	0.15	285	0.13	70	0.13	285	0.14	285	0.13	65	0.12	265	0.13	270	0.13	65	0.13	255	0.12	265	0.12	65	0.12	310	0.09	325	0.09	105	0.12	305	0.08	320	0.08	105	0.11
Yugoslavia (27.04.1992-04.02.2	530	0.26	385	0.18	15	0.03	500	0.24	350	0.16	15	0.03	460	0.22	280	0.13	10	0.02	445	0.21	235	0.11	15	0.03	420	0.12	230	0.06	15	0.02	430	0.12	230	0.06	10	0.01
Tajikistan	10	0.00	110	0.05	10	0.02	10	0.00	130	0.06	15	0.03	5	0.00	190	0.09	15	0.03	10	0.00	165	0.08	15	0.03	40	0.01	590	0.16	25	0.03	50	0.01	585	0.15	30	0.03
Cuba	20	0.01	210	0.10	10	0.02	20	0.01	210	0.10	10	0.02	15	0.01	195	0.09	10	0.02	20	0.01	210	0.10	10	0.02	30	0.01	380	0.10	10	0.01	75	0.02	655	0.17	20	0.02
Myanmar	15	0.01	215	0.10	15	0.03	10	0.00	350	0.16	10	0.02	10	0.00	410	0.19	10	0.02	15	0.01	445	0.20	20	0.04	15	0.00	485	0.13	20	0.02	15	0.00	545	0.14	20	0.02
Sudan (without South Sudan) (s	75	0.04	155	0.07	30	0.06	95	0.05	195	0.09	35	0.06	160	0.08	210	0.10	40	0.08	185	0.08	225	0.10	50	0.09	180	0.05	230	0.06	70	0.08	205	0.06	315	0.08	75	0.08
Nicaragua	-	-	-	-	35	0.07	-	-	-	-	135	0.24	-	-	-	-	180	0.35	-	-	-	-	215	0.38	5	0.00	-	-	405	0.44	-	-	-	-	500	0.53
Tanzania	-	-	240	0.11	-	-	-	-	330	0.15	-	-	5	0.00	365	0.17	-	-	5	0.00	390	0.18	-	-	5	0.00	460	0.12	-	-	5	0.00	525	0.14	5	0.01
Mali	70	0.03	735	0.35	20	0.04	70	0.03	635	0.30	20	0.04	65	0.03	585	0.27	20	0.04	65	0.03	530	0.24	20	0.04	55	0.02	480	0.13	20	0.02	55	0.01	355	0.09	25	0.03
Benin	20	0.01	70	0.03	80	0.15	30	0.01	80	0.04	85	0.15	30	0.01	80	0.04	75	0.14	30	0.01	115	0.05	80	0.14	30	0.01	145	0.04	90	0.10	40	0.01	455	0.12	95	0.10
Libya	70	0.03	75	0.04	85	0.16	60	0.03	70	0.03	90	0.16	75	0.04	80	0.04	105	0.20	60	0.03	85	0.04	140	0.25	90	0.02	95	0.03	175	0.19	85	0.02	85	0.02	205	0.22
Venezuela	5	0.00	10	0.00	10	0.02	15	0.01	20	0.01	10	0.02	20	0.01	20	0.01	30	0.06	35	0.02	30	0.01	30	0.05	45	0.01	45	0.01	45	0.05	55	0.01	505	0.13	35	0.04
Kyrgyzstan	120	0.06	155	0.07	25	0.05	115	0.05	145	0.07	25	0.05	115	0.05	140	0.07	30	0.06	110	0.05	135	0.06	30	0.05	115	0.03	150	0.04	55	0.06	125	0.03	145	0.04	55	0.06
Angola	185	0.09	160	0.08	15	0.03	185	0.09	160	0.08	15	0.03	175	0.08	155	0.07	10	0.02	175	0.08	145	0.07	15	0.03	170	0.05	140	0.04	15	0.02	165	0.04	135	0.04	15	0.02
Turkmenistan	30	0.01	35	0.02	-	-	30	0.01	35	0.02	-	-	25	0.01	25	0.01	-	-	25	0.01	25	0.01	-	-	115	0.03	175	0.05	60	0.07	70	0.02	65	0.02	35	0.04
Peru	5	0.00	15	0.01	10	0.02	5	0.00	20	0.01	10	0.02	5	0.00	25	0.01	15	0.03	5	0.00	40	0.02	10	0.02	5	0.00	95	0.03	15	0.02	10	0.00	275	0.07	15	0.02
Bangladesh	40	0.02	125	0.06	15	0.03	40	0.02	125	0.06	15	0.03	35	0.02	115	0.05	20	0.04	40	0.02	115	0.05	15	0.03	40	0.01	115	0.03	15	0.02	40	0.01	130	0.03	15	0.02
Burundi	-	-	15	0.01	-	-	-	-	15	0.01	-	-	-	-	15	0.01	-	-	5	0.00	15	0.01	-	-	255	0.07	45	0.01	5	0.01	20	0.01	25	0.01	5	0.01
Congo, Republic	45	0.02	80	0.04	-	-	45	0.02	85	0.04	-	-	45	0.02	75	0.04	-	-	40	0.02	80	0.04	-	-	45	0.01	110	0.03	5	0.01	45	0.01	125	0.03	5	0.01
Burkina Faso	25	0.01	35	0.02	55	0.10	25	0.01	35	0.02	55	0.10	25	0.01	30	0.01	60	0.12	20	0.01	35	0.02	70	0.13	25	0.01	35	0.01	80	0.09	30	0.01	35	0.01	90	0.09
Israel	5	0.00	70	0.03	5	0.01	5	0.00	60	0.03	5	0.01	10	0.00	50	0.02	5	0.01	10	0.00	45	0.02	5	0.01	25	0.01	70	0.02	15	0.02	25	0.01	125	0.03	15	0.02
No information	110	0.05	190	0.09	520	0.99	115	0.05	165	0.08	390	0.71	115	0.05	130	0.06	265	0.51	85	0.04	105	0.05	210	0.38	20	0.01	65	0.02	5	0.01	15	0.00	60	0.02	5	0.01
Kenya	30	0.01	60	0.03	10	0.02	30	0.01	65	0.03	5	0.01	30	0.01	65	0.03	5	0.01	35	0.02	70	0.03	10	0.02	40	0.01	75	0.02	10	0.01	40	0.01	75	0.02	20	0.02
South Sudan (since 09.07.2011)	5	0.00	20	0.01	-	-	5	0.00	15	0.01	-	-	20	0.01	25	0.01	-	-	20	0.01	30	0.01	-	-	40	0.01	50	0.01	-	-	70	0.02	75	0.02	15	0.02
Nepal	5	0.00	50	0.02	30	0.06	5	0.00	60	0.03	30	0.05	5	0.00	55	0.03	25	0.05	5	0.00	55	0.03	25	0.04	5	0.00	65	0.02	30	0.03	5	0.00	55	0.01	35	0.04
Colombia	5	0.00	10	0.00	20	0.04	5	0.00	25	0.01	30	0.05	5	0.00	15	0.01	30	0.06	5	0.00	15	0.01	35	0.06	5	0.00	25	0.01	55	0.06	25	0.01	25	0.01	50	0.05
Guinea-Bissau	35	0.02	25	0.01	30	0.06	30	0.01	25	0.01	35	0.06	30	0.01	20	0.01	30	0.06	30	0.01	25	0.01	30	0.05	35	0.01	25	0.01	40	0.04	25	0.01	20	0.01	45	0.05
Brazil	-	-	35	0.02	5	0.01	5	0.00	45	0.02	10	0.02	5	0.00	45	0.02	10	0.02	5	0.00	55	0.03	10	0.02	5	0.00	70	0.02	10	0.01	5	0.00	85	0.02	10	0.01
Niger	15	0.01	25	0.01	45	0.09	15	0.01	20	0.01	50	0.09	10	0.00	15	0.01	40	0.08	10	0.00	10	0.00	50	0.09	10	0.00	10	0.00	55	0.06	10	0.00	15	0.00	60	0.06
Liberia	40	0.02	35	0.02	25	0.05	35	0.02	35	0.02	25	0.05	30	0.01	30	0.01	20	0.04	30	0.01	30	0.01	25	0.04	30	0.01	25	0.01	25	0.03	25	0.01	25	0.01	25	0.03
United States	20	0.01	20	0.01	5	0.01	25	0.01	15	0.01	5	0.01	25	0.01	15	0.01	5	0.01	25	0.01	15	0.01	5	0.01	35	0.01	35	0.01	15	0.02	40	0.01	35	0.01	10	0.01
Cambodia	30	0.01	60	0.03	-	-	30	0.01	65	0.03	-	-	35	0.02	50	0.02	-	-	30	0.01	55	0.03	-	-	35	0.01	50	0.01	-	-	30	0.01	50	0.01	-	-
Mongolia	20	0.01	30	0.01	-	-	25	0.01	35	0.02	-	-	20	0.01	40	0.02	-	-	25	0.01	35	0.02	-	-	30	0.01	30	0.01	-	-	30	0.01	45	0.01	-	-
Soviet Union (until 25.12.1991)	30	0.01	50	0.02	-	-	30	0.01	50	0.02	-	-	30	0.01	45	0.02	-	-	25	0.01	40	0.02	-	-	20	0.01	40	0.01	-	-	25	0.01	40	0.01	-	-
Democratic People's Republic o	100	0.05	10	0.00	-	-	95	0.05	5	0.00	-	-	75	0.04	5	0.00	-	-	65	0.03	5	0.00	-	-	65	0.02	5	0.00	-	-	50	0.01	5	0.00	-	-
Djibouti	-	-	20	0.01	-	-	-	-	25	0.01	-	-	-	-	25	0.01	-	-	-	-	30	0.01	-	-	-	-	45	0.01	-	-	5	0.00	75	0.02	-	-
Ecuador	-	-	-	-	30	0.06	-	-	-	-	25	0.05	-	-	-	-	25	0.05	-	-	-	-	25	0.04	-	-	5	0.00	35	0.04	5	0.00	-	-	60	0.06
Chile	20	0.01	5	0.00	25	0.05	20	0.01	5	0.00	20	0.04	20	0.01	5	0.00	20	0.04	20	0.01	5	0.00	20	0.04	20	0.01	5	0.00	20	0.02	20	0.01	10	0.00	20	0.02
Laos	40	0.02	15	0.01	5	0.01	35	0.02	15	0.01	-	-	35	0.02	15	0.01	5	0.01	30	0.01	15	0.01	5	0.01	35	0.01	10	0.00	5	0.01	35	0.01	10	0.00	5	0.01
Rwanda	15	0.01	25	0.01	5	0.01	15	0.01	20	0.01	5	0.01	15	0.01	20	0.01	5	0.01	10	0.00	25	0.01	5	0.01	10	0.00	25	0.01	5	0.01	10	0.00	30	0.01	5	0.01
Honduras	10	0.00	-	-	5	0.01	10	0.00	-	-	5	0.01	10	0.00	-	-	10	0.02	5	0.00	-	-	15	0.03	10	0.00	5	0.00	10	0.01	10	0.00	5	0.00	20	0.02
Thailand	15	0.01	10	0.00	10	0.02	20	0.01	5	0.00	5	0.01	15	0.01	5	0.00	5	0.01	15	0.01	5	0.00	10	0.02	20	0.01	5	0.00	10	0.01	20	0.01	5	0.00	10	0.01
Sudan (including South Sudan) (	35	0.02	35	0.02	-	-	30	0.01	20	0.01	-	-	25	0.01	20	0.01	-	-	20	0.01	15	0.01	-	-	25	0.01	15	0.00	-	-	20	0.01	15	0.00	-	-
Philippinee	10	0.00	10	0.00	10	0.02	10	0.00	10	0.00	10	0.02	10	0.00	5	0.00	10	0.02	10	0.00	10	0.00	5	0.01	10	0.00	10	0.00	5	0.01	10	0.00	15	0.00	10	0.01
Saudi Arabia	5	0.00	5	0.00	-	-	5	0.00	5	0.00	-	-	5	0.00	5	0.00	-	-	5	0.00	10	0.00	-	-	10	0.00	20	0.01	-	-	5	0.00	20	0.01	10	0.01
United Kingdom	-	-	-	-	-	-	-	-	-	-	-	-	-	-	-	-	-	-	15	0.01	10	0.00	5	0.01	15	0.00	15	0.00	5	0.01	15	0.00	15	0.00	5	0.01
Zimbabwe	-	-	5	0.00	5	0.01	-	-	5	0.00	5	0.01	-	-	5	0.00	5	0.01	5	0.00	5	0.00	5	0.01	5	0.00	10	0.00	15	0.02	10	0.00	10	0.00	10	0.01
Kuwait	5	0.00	-	-	10	0.02	5	0.00	-	-	15	0.03	5	0.00	5	0.00	15	0.03	5	0.00	5	0.00	15	0.03	5	0.00	10	0.00	10	0.01	5	0.00	10	0.00	10	0.01
South Korea	25	0.01	-	-	-	-	30	0.01	-	-	-	-	30	0.01	-	-	-	-	25	0.01	5	0.00	-	-	25	0.01	5	0.00	-	-	20	0.01	5	0.00	-	-
South Africa	5	0.00	10	0.00	5	0.01	5	0.00	15	0.01	5	0.01	5	0.00	15	0.01	5	0.01	5	0.00	15	0.01	5	0.01	5	0.00	20	0.01	5	0.01	5	0.00	15	0.00	5	0.01
Bhutan	-	-	30	0.01	10	0.02	-	-	20	0.01	5	0.01	-	-	20	0.01	5	0.01	-	-	15	0.01	5	0.01	-	-	15	0.00	5	0.01	-	-	20	0.01	5	0.01
Mozambique	5	0.00	10	0.00	5	0.01	5	0.00	5	0.00	-	-	5	0.00	10	0.00	-	-	5	0.00	10	0.00	5	0.01	5	0.00	10	0.00	5	0.01	5	0.00	10	0.00	5	0.01
Zambia	-	-	15	0.01	-	-	-	-	15	0.01	-	-	-	-	15	0.01	-	-	-	-	15	0.01	-	-	-	-	15	0.00	-	-	-	-	20	0.01	-	-
Taiwan	20	0.01	5	0.00	-	-	20	0.01	5	0.00	-	-	20	0.01	5	0.00	-	-	15	0.01	5	0.00	-	-	15	0.00	5	0.00	-	-	10	0.00	5	0.00	-	-
Mauretania	5	0.00	5	0.00	10	0.02	5	0.00	5	0.00	5	0.01	5	0.00	10	0.00	5	0.01	5	0.00	5	0.00	5	0.01	5	0.00	5	0.00	10	0.01	5	0.00	-	-	5	0.01
Chad	5	0.00	10	0.00	5	0.01	5	0.00	10	0.00	-	-	5	0.00	10	0.00	-	-	5	0.00	10	0.00	-	-	5	0.00	10	0.00	5	0.01	5	0.00	5	0.00	5	0.01
Indonesia	5	0.00	5	0.00	5	0.01	5	0.00	5	0.00	5	0.01	5	0.00	5	0.00	-	-	5	0.00	5	0.00	-	-	5	0.00	10	0.00	-	-	5	0.00	10	0.00	-	-
Comoros	15	0.01	-	-	-	-	15	0.01	-	-	-	-	15	0.01	-	-	-	-	15	0.01	-	-	-	-	15	0.00	-	-	-	-	15	0.00	-	-	-	-
Jamaica	-	-	5	0.00	-	-	-	-	5	0.00	-	-	-	-	5	0.00	-	-	-	-	5	0.00	5	0.01	-	-	5	0.00	5	0.01	5	0.00	5	0.00	5	0.01
Bolivia	-	-	-	-	-	-	-	-	-	-	-	-	-	-	-	-	-	-	-	-	10	0.00	-	-	-	-	10	0.00	-	-	-	-	10	0.00	-	-
Timor Leste	-	-	-	-	-	-	-	-	-	-	-	-	-	-	-	-	-	-	-	-	-	-	-	-	-	-	15	0.00	-	-	-	-	10	0.00	-	-
Italy	-	-	5	0.00	-	-	-	-	-	-	-	-	-	-	-	-	-	-	-	-	-	-	-	-	-	-	-	-	-	-	15	0.00	5	0.00	-	-
Argentina	5	0.00	-	-	-	-	5	0.00	-	-	-	-	5	0.00	-	-	-	-	5	0.00	-	-	-	-	5	0.00	5	0.00	-	-	5	0.00	5	0.00	-	-
Central African Republic	5	0.00	5	0.00	-	-	5	0.00	5	0.00	-	-	-	-	5	0.00	-	-	-	-	5	0.00	-	-	5	0.00	5	0.00	-	-	5	0.00	5	0.00	-	-
Bahrain	5	0.00	-	-	-	-	5	0.00	-	-	-	-	5	0.00	-	-	-	-	5	0.00	-	-	-	-	5	0.00	-	-	-	-	5	0.00	-	-	-	-
Hongkong	-	-	-	-	-	-	-	-	-	-	-	-	-	-	-	-	-	-	5	0.00	-	-	-	-	5	0.00	-	-	-	-	5	0.00	-	-	-	-
Canada	-	-	-	-	-	-	-	-	-	-	-	-	-	-	-	-	-	-	-	-	-	-	-	-	5	0.00	5	0.00	-	-	5	0.00	-	-	-	-
Mexico	-	-	5	0.00	-	-	-	-	5	0.00	-	-	-	-	5	0.00	-	-	5	0.00	5	0.00	-	-	-	-	5	0.00	5	0.01	-	-	5	0.00	-	-
Botswana	-	-	10	0.00	-	-	-	-	10	0.00	-	-	-	-	5	0.00	-	-	-	-	5	0.00	-	-	-	-	5	0.00	-	-	-	-	5	0.00	-	-
Gabun	5	0.00	-	-	-	-	5	0.00	-	-	-	-	5	0.00	-	-	-	-	5	0.00	-	-	-	-	5	0.00	-	-	-	-	5	0.00	-	-	-	-
Malaysia	5	0.00	-	-	-	-	5	0.00	-	-	-	-	5	0.00	-	-	-	-	5	0.00	-	-	-	-	5	0.00	-	-	-	-	5	0.00	-	-	-	-
Oman	-	-	-	-	-	-	5	0.00	-	-	-	-	5	0.00	-	-	-	-	5	0.00	5	0.00	-	-	5	0.00	-	-	-	-	5	0.00	-	-	-	-
Cabo Verde	-	-	-	-	-	-	5	0.00	-	-	-	-	5	0.00	-	-	-	-	5	0.00	-	-	-	-	5	0.00	-	-	-	-	5	0.00	-	-	-	-
El Salvador	5	0.00	-	-	5	0.01	5	0.00	-	-	5	0.01	5	0.00	-	-	5	0.01	5	0.00	-	-	-	-	5	0.00	-	-	-	-	5	0.00	-	-	-	-
Eswatini	-	-	-	-	-	-	-	-	-	-	-	-	-	-	-	-	-	-	-	-	-	-	-	-	-	-	-	-	-	-	-	-	5	0.00	-	-
Haiti	5	0.00	-	-	-	-	-	-	-	-	-	-	-	-	-	-	-	-	5	0.00	-	-	-	-	5	0.00	-	-	-	-	-	-	-	-	-	-
Madagascar	-	-	-	-	-	-	-	-	-	-	-	-	-	-	-	-	-	-	-	-	-	-	-	-	5	0.00	-	-	-	-	5	0.00	-	-	-	-
Namibia	-	-	-	-	-	-	-	-	-	-	-	-	-	-	-	-	-	-	-	-	-	-	-	-	5	0.00	-	-	-	-	5	0.00	-	-	-	-
Romania	5	0.00	5	0.00	-	-	5	0.00	5	0.00	-	-	-	-	-	-	-	-	-	-	-	-	-	-	-	-	-	-	-	-	5	0.00	-	-	-	-
Saint Vincent and the Grenadine	5	0.00	-	-	-	-	5	0.00	-	-	-	-	5	0.00	-	-	-	-	5	0.00	-	-	-	-	5	0.00	-	-	-	-	-	-	-	-	-	-

Legend: © Statistisches Bundesamt (Destatis), 2024, accessed on 18.06.2024; *including (1) persons with ongoing asylum applications, (2) persons who have received a protection status, and (3) persons whose application has been denied and who are obliged to leave the country

5. SENSITIVITY ANALYSES 4 – 5

5.1. Figure S26: Forest Plot of Sensitivity analysis 4, nuanced analysis of peri-pandemic phase

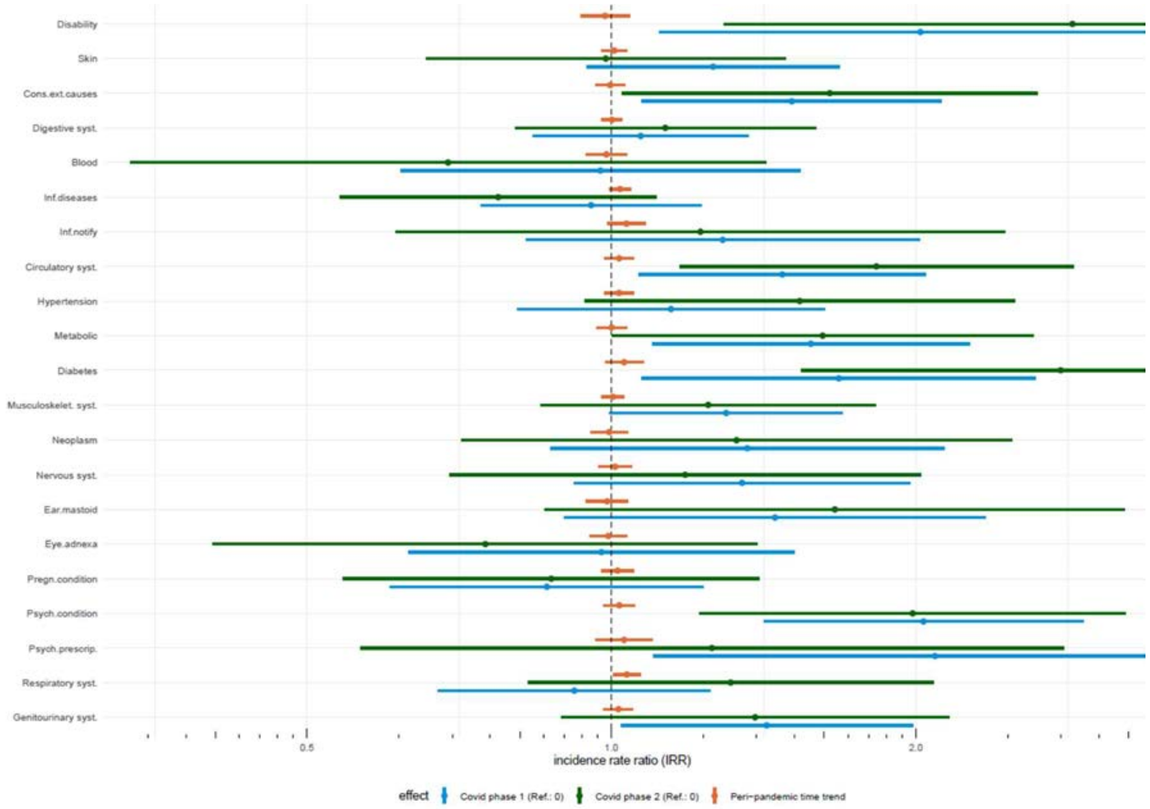

Legend: Reference: Pre-pandemic phase (before 02/2020). Covid phase 1: representing the early phase until the onset of vaccine availability, i.e. 02/2020 – 07/2021. Covid phase 2 of the pandemic: Emerging variants phase (e.g. delta, omicron etc.) and increasing vaccine role out, i.e. 08/2021 to 06/2023. N = 314 centre-months. Source data are provided in the 'source_data.xlsx' file.

5.2. Figure S27: Forest Plot of Sensitivity analysis 5, seasonality effects

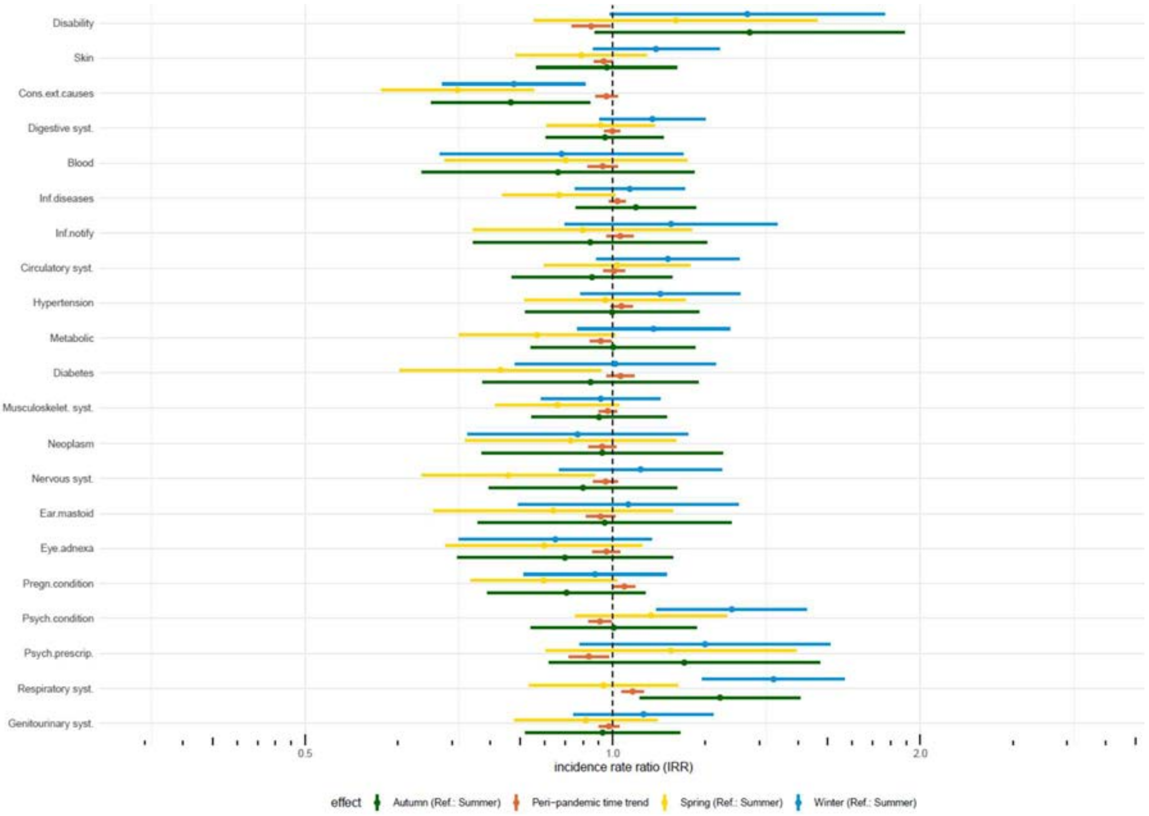

Legend: Reference: Pre-pandemic phase (before 02/2020). Autumn: months October and November. Spring: months March to May. Winter: months December to February. Summer (reference): June to September. N = 314 centre-months. Source data are provided in the 'source_data.xlsx' file.

6. SUPPLEMENTARY TABLES S1 – S3

6.1 Supplementary Table S1: Sociodemographic characteristics of patients in refugee centres per month, number of cases of 21 indicators per month as well as incidence proportions of 21 indicators per month, 2018 – 2023, N = 109,175 with 215,864 patient-months.

Table S1 Sociodemographic characteristics of patients in refugee centres per month, number of cases of 21 indicators per month as well as incidence proportions of 21 indicators per month, 2018 – 2023, N = 109,175 with 215,864 patient-months.

Variable	2018	2019	2020	2021	2022	2023	Total
Centre-months cluster	62	138	152	196	213	72	833
REC	49 (79%)	105 (76%)	122 (80%)	143 (73%)	161 (76%)	56 (78%)	636 (76%)
REG	13 (21%)	33 (24%)	30 (20%)	53 (27%)	52 (24%)	16 (22%)	197 (24%)
Sociodemographic characteristics							
% male (pat.)							
mean ± sd	6 ± 0.94	6 ± 1.4	5.7 ± 1.5	6.1 ± 1.3	6 ± 1.7	6.8 ± 2	6.1 ± 1.5
median (Q1, Q3)	5.6 (5.5, 6.2)	5.8 (5.3, 7)	5.8 (5, 6.5)	6.2 (5.4, 6.9)	6.1 (5, 7.1)	7.1 (5.9, 8)	6.1 (5.3, 6.9)
min - max	4.5 -- 9.1	1.7 -- 10	0 -- 9.5	2 -- 10	0 -- 10	2.7 -- 10	0 -- 10
% adults (pat.)							
mean ± sd	8 ± 1.1	8.1 ± 0.9	8 ± 1.1	7.9 ± 1	7.8 ± 1.1	8.2 ± 1.1	8 ± 1
median (Q1, Q3)	8.1 (7.2, 8.9)	8.2 (7.5, 8.7)	8.3 (7.6, 8.7)	7.9 (7.4, 8.5)	7.8 (7.1, 8.4)	8.2 (7.7, 8.7)	8 (7.4, 8.6)
min - max	5.5 -- 10	5.8 -- 10	0 -- 10	5.5 -- 10	4.6 -- 10	4.8 -- 10	0 -- 10
% Nigerian pat.							
mean ± sd	0.17 ± 0.17	0.24 ± 0.21	0.087 ± 0.072	0.045 ± 0.063	0.02 ± 0.031	0.028 ± 0.037	0.086 ± 0.13
median (Q1, Q3)	0.15 (0.032, 0.29)	0.21 (0.06, 0.36)	0.076 (0.035, 0.12)	0.024 (0, 0.052)	0.002 (0, 0.03)	0.011 (0, 0.049)	0.036 (0, 0.1)
min - max	0 -- 0.8	0 -- 0.92	0 -- 0.38	0 -- 0.35	0 -- 0.18	0 -- 0.17	0 -- 0.92
% Afghani pat.							
mean ± sd	0.1 ± 0.12	0.1 ± 0.11	0.16 ± 0.14	0.25 ± 0.23	0.2 ± 0.16	0.2 ± 0.17	0.18 ± 0.18
median (Q1, Q3)	0.043 (0.011, 0.15)	0.054 (0, 0.2)	0.17 (0.012, 0.27)	0.17 (0.049, 0.45)	0.18 (0.059, 0.32)	0.16 (0.06, 0.28)	0.14 (0.018, 0.29)
min - max	0 -- 0.39	0 -- 0.44	0 -- 0.51	0 -- 0.84	0 -- 0.59	0 -- 0.58	0 -- 0.84
% Syrian pat.							
mean ± sd	0.066 ± 0.051	0.037 ± 0.053	0.091 ± 0.13	0.16 ± 0.19	0.14 ± 0.23	0.2 ± 0.28	0.12 ± 0.19
median (Q1, Q3)	0.07 (0.0089, 0.11)	0.0029 (0, 0.061)	0.029 (0, 0.15)	0.07 (0, 0.31)	0.05 (0, 0.15)	0.096 (0.033, 0.18)	0.047 (0, 0.14)
min - max	0 -- 0.15	0 -- 0.27	0 -- 0.51	0 -- 0.87	0 -- 1	0 -- 1	0 -- 1
% Iraqi pat.							
mean ± sd	0.082 ± 0.07	0.038 ± 0.046	0.027 ± 0.041	0.03 ± 0.044	0.028 ± 0.045	0.0055 ± 0.013	0.032 ± 0.048
median (Q1, Q3)	0.077 (0, 0.13)	0.0058 (0, 0.073)	0 (0, 0.043)	0 (0, 0.048)	0.0084 (0, 0.041)	0 (0, 0)	0 (0, 0.054)
min - max	0 -- 0.29	0 -- 0.18	0 -- 0.15	0 -- 0.25	0 -- 0.32	0 -- 0.062	0 -- 0.32
% Turkish pat.							
mean ± sd	0.023 ± 0.032	0.025 ± 0.037	0.033 ± 0.044	0.026 ± 0.037	0.064 ± 0.11	0.087 ± 0.12	0.042 ± 0.076
median (Q1, Q3)	0 (0, 0.039)	0 (0, 0.043)	0.011 (0, 0.062)	0 (0, 0.044)	0 (0, 0.09)	0.056 (0, 0.12)	0 (0, 0.059)
min - max	0 -- 0.14	0 -- 0.14	0 -- 0.19	0 -- 0.19	0 -- 0.66	0 -- 0.61	0 -- 0.66
Patients							
mean ± sd	281 ± 230	233 ± 218	213 ± 183	241 ± 228	306 ± 380	299 ± 409	259 ± 287
median (Q1, Q3)	166 (97, 399)	132 (95, 392)	152 (82, 264)	180 (96, 272)	173 (90, 314)	163 (73, 299)	156 (91, 303)
min - max	68 -- 769	10 -- 934	5 -- 838	13 -- 1319	3 -- 2227	30 -- 1856	3 -- 2227
Cumulative Incidence (Incidence proportions) in %							
Disability Cum. Incidence (%) (pat.)							
mean ± sd	1.1 ± 1.5	0.88 ± 1.4	0.94 ± 1.8	2.2 ± 3.5	1.1 ± 1.9	0.99 ± 2	1.3 ± 2.4
median (Q1, Q3)	0 (0, 2.1)	0 (0, 1.5)	0 (0, 1.2)	0 (0, 3.1)	0 (0, 1.6)	0 (0, 0.9)	0 (0, 1.8)
min - max	0 -- 6.3	0 -- 6	0 -- 7.3	0 -- 16	0 -- 13	0 -- 8.2	0 -- 16
Skin Cum. Incidence (%) (pat.)							
mean ± sd	3.9 ± 2.8	4.5 ± 3.6	5.2 ± 3.9	4 ± 3.4	4.3 ± 3.5	4.6 ± 4.1	4.4 ± 3.6
median (Q1, Q3)	4.2 (1.3, 5.7)	4.7 (1.5, 6.5)	5.2 (1.8, 8)	3.9 (0, 6.2)	3.9 (1.7, 6.5)	4.5 (0.45, 7.1)	4.3 (1.3, 6.6)
min - max	0 -- 11	0 -- 25	0 -- 17	0 -- 16	0 -- 23	0 -- 22	0 -- 25

Cons.ext.causes Cum. Incidence (%) (pat.)							
mean ± sd	4.7 ± 3	4.3 ± 4.1	4.3 ± 3.7	3.6 ± 3.3	3.5 ± 3.1	3 ± 3	3.8 ± 3.4
median (Q1, Q3)	5.2 (2.5, 6.9)	4.4 (0, 6.4)	4.5 (0, 6.8)	3.4 (0, 5.5)	3.3 (0, 5.3)	2.6 (0, 4.8)	3.7 (0, 6)
min - max	0 -- 11	0 -- 30	0 -- 13	0 -- 17	0 -- 14	0 -- 9.6	0 -- 30
Digestive syst. Cum. Incidence (%) (pat.)							
mean ± sd	8 ± 3.6	7.9 ± 4.7	10 ± 6.1	10 ± 7.1	9.6 ± 6.5	11 ± 6.4	9.6 ± 6.2
median (Q1, Q3)	8 (5.6, 10)	8.7 (4.8, 11)	10 (6.7, 13)	9.7 (5.8, 15)	9 (4.9, 14)	9.8 (6.3, 15)	9.2 (5.6, 13)
min - max	0 -- 19	0 -- 22	0 -- 28	0 -- 30	0 -- 37	0 -- 37	0 -- 37
Blood Cum. Incidence (%) (pat.)							
mean ± sd	0.66 ± 1	0.75 ± 1.5	0.63 ± 1.2	0.32 ± 0.8	0.31 ± 0.74	0.28 ± 0.79	0.47 ± 1
median (Q1, Q3)	0 (0, 1.4)	0 (0, 1.3)	0 (0, 0.97)	0 (0, 0)	0 (0, 0)	0 (0, 0)	0 (0, 0.26)
min - max	0 -- 4.4	0 -- 9.2	0 -- 5	0 -- 4.7	0 -- 5.2	0 -- 3.9	0 -- 9.2
Inf.diseases Cum. Incidence (%) (pat.)							
mean ± sd	10 ± 5.6	8.5 ± 6.4	7.2 ± 5.7	7 ± 5.3	9.9 ± 7.2	11 ± 6.1	8.6 ± 6.3
median (Q1, Q3)	9.6 (5.9, 14)	8.8 (3.5, 12)	6.7 (3.4, 10)	6.8 (2.6, 11)	8.9 (4.5, 14)	10 (5.9, 15)	8.2 (3.9, 13)
min - max	0 -- 33	0 -- 25	0 -- 35	0 -- 22	0 -- 34	0 -- 22	0 -- 35
Inf.notify Cum. Incidence (%) (pat.)							
mean ± sd	1 ± 1.3	0.79 ± 1.2	0.66 ± 1.2	0.42 ± 1	0.81 ± 1.6	0.41 ± 0.83	0.67 ± 1.3
median (Q1, Q3)	0 (0, 2)	0 (0, 1.6)	0 (0, 1.2)	0 (0, 0)	0 (0, 1.3)	0 (0, 0.37)	0 (0, 1.1)
min - max	0 -- 4	0 -- 6.1	0 -- 5.5	0 -- 7.7	0 -- 12	0 -- 3.8	0 -- 12
Circulatory syst. Cum. Incidence (%) (pat.)							
mean ± sd	3.5 ± 2.7	2.9 ± 2.6	3 ± 3	2.7 ± 2.7	2.9 ± 2.9	2.5 ± 2.8	2.9 ± 2.8
median (Q1, Q3)	3.5 (1, 5.2)	3 (0, 4.9)	2.8 (0, 5.3)	2 (0, 4.6)	2.5 (0, 4.8)	1.5 (0, 4)	2.6 (0, 4.9)
min - max	0 -- 13	0 -- 9.2	0 -- 12	0 -- 12	0 -- 11	0 -- 11	0 -- 13
Hypertension Cum. Incidence (%) (pat.)							
mean ± sd	2.2 ± 2.8	1.7 ± 2.1	1.4 ± 1.9	1.3 ± 2	1.7 ± 2.4	1.6 ± 2.5	1.6 ± 2.2
median (Q1, Q3)	1.1 (0, 3.5)	0 (0, 3.1)	0 (0, 2.5)	0 (0, 2)	0 (0, 2.9)	0 (0, 2.7)	0 (0, 2.8)
min - max	0 -- 12	0 -- 8.8	0 -- 8.2	0 -- 9.2	0 -- 12	0 -- 8.9	0 -- 12
Metabolic Cum. Incidence (%) (pat.)							
mean ± sd	2.8 ± 2.4	2.2 ± 2.4	2.2 ± 2.4	2.3 ± 2.5	2.3 ± 2.4	2 ± 2.2	2.3 ± 2.4
median (Q1, Q3)	2.9 (0, 4.4)	2.1 (0, 4.1)	1.8 (0, 4.1)	1.8 (0, 3.9)	1.9 (0, 3.8)	1.4 (0, 3.2)	1.9 (0, 4)
min - max	0 -- 8.1	0 -- 8.6	0 -- 8	0 -- 11	0 -- 9.6	0 -- 8.4	0 -- 11
Diabetes Cum. Incidence (%) (pat.)							
mean ± sd	0.99 ± 1.3	0.81 ± 1.3	0.8 ± 1.1	0.91 ± 1.4	0.96 ± 1.4	0.94 ± 1.3	0.89 ± 1.3
median (Q1, Q3)	0 (0, 1.8)	0 (0, 1.5)	0 (0, 1.6)	0 (0, 1.8)	0 (0, 2)	0 (0, 1.7)	0 (0, 1.8)
min - max	0 -- 3.9	0 -- 7.4	0 -- 5.4	0 -- 6.1	0 -- 6.1	0 -- 4.7	0 -- 7.4
Musculoskelet. syst. Cum. Incidence (%) (pat.)							
mean ± sd	7 ± 4	7.3 ± 4.9	7.3 ± 4.7	6.3 ± 4.6	5.1 ± 3.4	5 ± 3.8	6.3 ± 4.4
median (Q1, Q3)	5.8 (4.1, 10)	7.4 (4.6, 10)	7.2 (4.6, 11)	5.6 (3.1, 9.6)	5.1 (2.6, 7.4)	5.4 (2, 7.8)	6 (3.3, 9.4)
min - max	0 -- 16	0 -- 25	0 -- 19	0 -- 21	0 -- 14	0 -- 15	0 -- 25
Neoplasm Cum. Incidence (%) (pat.)							
mean ± sd	0.56 ± 0.91	0.73 ± 1.3	0.76 ± 1.2	0.46 ± 0.87	0.41 ± 0.97	0.51 ± 1.3	0.56 ± 1.1
median (Q1, Q3)	0 (0, 0.92)	0 (0, 1.5)	0 (0, 1.5)	0 (0, 0.65)	0 (0, 0)	0 (0, 0.14)	0 (0, 0.77)
min - max	0 -- 3.5	0 -- 9.6	0 -- 4.2	0 -- 4.4	0 -- 5.2	0 -- 6.7	0 -- 9.6
Nervous syst. Cum. Incidence (%) (pat.)							
mean ± sd	3.6 ± 2.5	2.7 ± 2.5	2.9 ± 2.9	2.7 ± 2.7	1.9 ± 2.1	2.2 ± 2.6	2.6 ± 2.6
median (Q1, Q3)	4.1 (0, 5.2)	2.9 (0, 4.5)	2.9 (0, 4.7)	2.3 (0, 4.7)	1.6 (0, 3.3)	1.9 (0, 3.9)	2.4 (0, 4.3)
min - max	0 -- 8.4	0 -- 12	0 -- 14	0 -- 11	0 -- 9.7	0 -- 11	0 -- 14
Ear.mastoid Cum. Incidence (%) (pat.)							
mean ± sd	0.97 ± 1.2	0.72 ± 1.1	0.85 ± 1.4	0.85 ± 1.3	0.83 ± 1.2	0.96 ± 1.5	0.84 ± 1.3
median (Q1, Q3)	0.57 (0, 1.7)	0 (0, 1.3)	0 (0, 1.5)	0 (0, 1.5)	0 (0, 1.5)	0 (0, 1.9)	0 (0, 1.5)

min - max	0 -- 4.5	0 -- 4.8	0 -- 7.6	0 -- 7.8	0 -- 5.2	0 -- 8.3	0 -- 8.3
Eye.adnexa Cum. Incidence (%) (pat.)							
mean ± sd	1.4 ± 1.6	1.8 ± 2	1.8 ± 2	1.6 ± 2	1.5 ± 1.9	1.4 ± 1.8	1.6 ± 1.9
median (Q1, Q3)	1.2 (0, 2.9)	1.3 (0, 3.2)	1.2 (0, 3.3)	0 (0, 3)	0.9 (0, 2.4)	0 (0, 2.9)	0.86 (0, 2.9)
min - max	0 -- 5.1	0 -- 8.2	0 -- 9.6	0 -- 8.4	0 -- 9.6	0 -- 6.8	0 -- 9.6
Pregn.condition Cum. Incidence (%) (pat.)							
mean ± sd	2.7 ± 2.8	3.1 ± 3.8	2.8 ± 3	2.5 ± 2.8	2.8 ± 3.4	2.4 ± 3.2	2.7 ± 3.2
median (Q1, Q3)	3 (0, 4.9)	0 (0, 6.4)	2.2 (0, 5)	1.9 (0, 4.8)	1.6 (0, 4.5)	0.74 (0, 3.9)	1.7 (0, 4.9)
min - max	0 -- 11	0 -- 14	0 -- 10	0 -- 12	0 -- 16	0 -- 14	0 -- 16
Psych.condition Cum. Incidence (%) (pat.)							
mean ± sd	7.1 ± 4.7	6.5 ± 5.2	6.9 ± 5.8	7.6 ± 6	5.2 ± 4.5	5.7 ± 5.8	6.5 ± 5.4
median (Q1, Q3)	6.8 (3.4, 8.6)	5.9 (2.7, 9.3)	5.9 (2, 11)	7 (2.9, 12)	4.5 (1.4, 8.4)	4.5 (0, 8.2)	5.6 (2.2, 10)
min - max	0 -- 19	0 -- 30	0 -- 38	0 -- 24	0 -- 21	0 -- 25	0 -- 38
Psych.prescrip. Cum. Incidence (%) (pat.)							
mean ± sd	1.5 ± 1.7	1.8 ± 2.7	2.4 ± 3	1.3 ± 2	0.95 ± 1.7	1.3 ± 2.6	1.5 ± 2.4
median (Q1, Q3)	0.93 (0, 3.1)	0 (0, 3.2)	1.3 (0, 3.8)	0 (0, 2.2)	0 (0, 1.4)	0 (0, 1.5)	0 (0, 2.4)
min - max	0 -- 6.5	0 -- 15	0 -- 14	0 -- 13	0 -- 9.7	0 -- 12	0 -- 15
Respiratory syst. Cum. Incidence (%) (pat.)							
mean ± sd	14 ± 7.3	14 ± 7.8	9.8 ± 9.1	6.6 ± 5.3	9.3 ± 6.9	12 ± 7.6	10 ± 7.7
median (Q1, Q3)	13 (10, 18)	14 (8.7, 20)	8.1 (1.9, 15)	6 (2.4, 10)	8.6 (3.7, 14)	12 (5.3, 17)	9.4 (3.8, 15)
min - max	0 -- 40	0 -- 39	0 -- 35	0 -- 23	0 -- 29	0 -- 29	0 -- 40
Genitourinary syst. Cum. Incidence (%) (pat.)							
mean ± sd	2.6 ± 2.1	3.4 ± 4.5	3 ± 2.7	3.2 ± 2.7	2.7 ± 2.7	2.8 ± 3	3 ± 3.1
median (Q1, Q3)	2.9 (0, 4.1)	3.1 (0, 4.9)	3.3 (0, 5)	3.1 (0, 5)	2.3 (0, 4.5)	2.5 (0, 3.9)	2.8 (0, 4.8)
min - max	0 -- 7.7	0 -- 40	0 -- 9.5	0 -- 12	0 -- 11	0 -- 16	0 -- 40

6.2 Supplementary Table S2: Sociodemographic characteristics of patients and occupancy in refugee centres per month, number of cases of 21 indicators per month as well as incidence proportions of 21 indicators per month, 2018 – 2023, N = 102,967 refugees.

Table S2: Sociodemographic characteristics of patients and occupancy in refugee centres per month, number of cases of 21 indicators per month as well as incidence proportions of 21 indicators per month, 2018 – 2023, N = 102,967 refugees.

Variable	2018	2019	2020	2021	2022	2023	Total
Centre-months	10	70	34	47	60	18	239
cluster							
REC	9 (90%)	55 (79%)	24 (71%)	22 (47%)	27 (45%)	8 (44%)	145 (61%)
REG	1 (10%)	15 (21%)	10 (29%)	25 (53%)	33 (55%)	10 (56%)	94 (39%)
Socio-demographic characteristics							
% male (pat.)							
mean ± sd	6.4 ± 1	6.5 ± 1.2	5.3 ± 1.2	5.5 ± 0.83	5.9 ± 1.2	7.3 ± 0.61	6 ± 1.2
median (Q1, Q3)	6.2 (5.6, 6.8)	6.6 (5.5, 7.3)	5.6 (4.5, 5.8)	5.7 (4.8, 6)	6 (5.5, 6.6)	7.3 (7, 7.7)	6 (5.3, 6.9)
min - max	5.4 -- 9	3.5 -- 9	2.9 -- 8.5	3.7 -- 7.6	2.4 -- 8.6	6.4 -- 8.8	2.4 -- 9
% adults (pat.)							
mean ± sd	8.1 ± 1	8.3 ± 1	7.8 ± 0.99	7.6 ± 0.83	7.7 ± 0.98	8.3 ± 0.49	7.9 ± 0.97
median (Q1, Q3)	8.4 (7.2, 8.8)	8.5 (7.6, 8.9)	7.7 (7.2, 8.7)	7.8 (7, 8.1)	7.8 (7.3, 8.5)	8.5 (7.9, 8.7)	8 (7.3, 8.7)
min - max	6.4 -- 9.4	6.1 -- 10	5.7 -- 9.5	5.9 -- 9	4.6 -- 9.2	7.4 -- 9	4.6 -- 10
% Nigerian pat.							
mean ± sd	0.16 ± 0.13	0.29 ± 0.22	0.074 ± 0.05	0.029 ± 0.052	0.028 ± 0.035	0.028 ± 0.029	0.12 ± 0.17
median (Q1, Q3)	0.14 (0.054, 0.29)	0.29 (0.069, 0.39)	0.075 (0.038, 0.11)	0.02 (0, 0.038)	0.021 (0, 0.038)	0.023 (0, 0.036)	0.04 (0.0092, 0.15)
min - max	0 -- 0.37	0 -- 0.92	0 -- 0.18	0 -- 0.35	0 -- 0.18	0 -- 0.093	0 -- 0.92
% Afghani pat.							
mean ± sd	0.11 ± 0.12	0.086 ± 0.11	0.2 ± 0.12	0.36 ± 0.19	0.19 ± 0.13	0.26 ± 0.11	0.2 ± 0.17
median (Q1, Q3)	0.065 (0, 0.15)	0.038 (0, 0.15)	0.18 (0.068, 0.27)	0.35 (0.17, 0.53)	0.16 (0.1, 0.25)	0.27 (0.2, 0.32)	0.17 (0.055, 0.29)
min - max	0 -- 0.39	0 -- 0.39	0 -- 0.44	0 -- 0.77	0 -- 0.52	0.055 -- 0.46	0 -- 0.77
% Syrian pat.							
mean ± sd	0.046 ± 0.035	0.037 ± 0.043	0.058 ± 0.083	0.066 ± 0.13	0.061 ± 0.06	0.082 ± 0.059	0.056 ± 0.077
median (Q1, Q3)	0.052 (0, 0.071)	0.03 (0, 0.062)	0.019 (0, 0.11)	0 (0, 0.046)	0.048 (0, 0.1)	0.079 (0.048, 0.1)	0.033 (0, 0.09)
min - max	0 -- 0.095	0 -- 0.16	0 -- 0.33	0 -- 0.5	0 -- 0.25	0 -- 0.23	0 -- 0.5
% Iraqi pat.							
mean ± sd	0.1 ± 0.051	0.049 ± 0.044	0.053 ± 0.053	0.026 ± 0.039	0.031 ± 0.037	0.01 ± 0.017	0.04 ± 0.045
median (Q1, Q3)	0.1 (0.071, 0.14)	0.049 (0, 0.082)	0.033 (0, 0.11)	0 (0, 0.046)	0.019 (0, 0.049)	0 (0, 0.016)	0.022 (0, 0.071)
min - max	0 -- 0.18	0 -- 0.14	0 -- 0.15	0 -- 0.16	0 -- 0.16	0 -- 0.062	0 -- 0.18
% Turkish pat.							
mean ± sd	0.05 ± 0.049	0.04 ± 0.042	0.051 ± 0.046	0.02 ± 0.026	0.088 ± 0.12	0.11 ± 0.094	0.055 ± 0.076
median (Q1, Q3)	0.038 (0, 0.08)	0.034 (0, 0.066)	0.038 (0, 0.081)	0 (0, 0.043)	0.057 (0, 0.13)	0.078 (0.057, 0.15)	0.038 (0, 0.079)
min - max	0 -- 0.14	0 -- 0.13	0 -- 0.18	0 -- 0.075	0 -- 0.51	0 -- 0.39	0 -- 0.51
patients							
N	10	70	34	47	60	18	239
mean ± sd	160 ± 183	228 ± 221	219 ± 170	227 ± 196	265 ± 261	275 ± 233	237 ± 220
median (Q1, Q3)	98 (80, 136)	130 (102, 194)	208 (91, 269)	168 (97, 223)	188 (142, 276)	199 (143, 303)	155 (105, 249)
min - max	68 -- 675	46 -- 934	62 -- 755	36 -- 880	70 -- 1390	88 -- 924	36 -- 1390
Cumulative Incidence (Incidence proportions) in %							
Disability Cum. Incidence (%) (pat.)							
mean ± sd	0.43 ± 1.4	1.1 ± 1.8	1.5 ± 2.3	2.1 ± 3.4	1.8 ± 2.6	1.7 ± 2.5	1.5 ± 2.5
median (Q1, Q3)	0 (0, 0)	0 (0, 2.3)	0 (0, 2.5)	0 (0, 3)	0.56 (0, 2.9)	0 (0, 2.7)	0 (0, 2.7)
min - max	0 -- 4.3	0 -- 6	0 -- 6.9	0 -- 14	0 -- 13	0 -- 6.6	0 -- 14
Skin Cum. Incidence (%) (pat.)							

mean ± sd	2.8 ± 3.1	4 ± 3.1	6.1 ± 3.8	5.1 ± 3.1	5.1 ± 3.2	8.1 ± 5	5 ± 3.6
median (Q1, Q3)	1.9 (0, 5.3)	4 (0, 6.4)	7.1 (3.8, 8.5)	4.7 (3.2, 6.7)	4.7 (3.3, 7)	7.9 (5.6, 9.6)	4.8 (3, 7.3)
min - max	0 -- 7.5	0 -- 13	0 -- 14	0 -- 12	0 -- 15	0 -- 22	0 -- 22
Cons.ext.causes Cum. Incidence (%) (pat.)							
mean ± sd	2.7 ± 3.3	4.4 ± 3.7	5.1 ± 3.9	4.5 ± 3.4	4.5 ± 3.1	4.1 ± 2.8	4.5 ± 3.4
median (Q1, Q3)	1 (0, 5.5)	4.7 (0, 6.9)	4.9 (1.8, 7.8)	4.2 (2, 6.6)	3.8 (2.2, 6.1)	4.3 (2.7, 6.2)	4.5 (1.6, 6.6)
min - max	0 -- 8.8	0 -- 15	0 -- 12	0 -- 12	0 -- 12	0 -- 9.1	0 -- 15
Digestive syst. Cum. Incidence (%) (pat.)							
mean ± sd	5.6 ± 2.6	7.4 ± 5.1	9.4 ± 4.3	10 ± 4.3	10 ± 5.5	11 ± 5.9	9.2 ± 5.1
median (Q1, Q3)	6.3 (3.3, 7.4)	7.9 (3.7, 11)	10 (6.7, 13)	10 (7.6, 12)	9 (6.5, 14)	12 (6.5, 15)	9.2 (5.9, 12)
min - max	2.2 -- 9.1	0 -- 22	0 -- 17	0 -- 23	0 -- 28	0 -- 21	0 -- 28
Blood Cum. Incidence (%) (pat.)							
mean ± sd	0.65 ± 1.5	0.54 ± 1.1	1.4 ± 1.7	0.7 ± 1.2	0.39 ± 0.71	0.36 ± 0.69	0.64 ± 1.2
median (Q1, Q3)	0 (0, 0)	0 (0, 0)	0.62 (0, 2.3)	0 (0, 1.4)	0 (0, 0.49)	0 (0, 0)	0 (0, 1.3)
min - max	0 -- 4.4	0 -- 4.2	0 -- 5	0 -- 4.7	0 -- 2.7	0 -- 1.7	0 -- 5
Inf.diseases Cum. Incidence (%) (pat.)							
mean ± sd	8 ± 5.1	8.4 ± 7	9.8 ± 5.8	9 ± 5	12 ± 6.9	12 ± 6.3	10 ± 6.5
median (Q1, Q3)	7.7 (5, 13)	8.2 (3, 12)	9.3 (6.4, 15)	9.9 (5.3, 13)	12 (7.4, 15)	14 (6, 16)	9.6 (5.1, 14)
min - max	0 -- 15	0 -- 25	0 -- 24	0 -- 18	0 -- 34	1.2 -- 22	0 -- 34
Inf.notify Cum. Incidence (%) (pat.)							
mean ± sd	0.4 ± 1.3	0.71 ± 1.3	0.96 ± 1.4	0.68 ± 1.4	0.94 ± 1.2	0.43 ± 0.86	0.76 ± 1.3
median (Q1, Q3)	0 (0, 0)	0 (0, 1.6)	0 (0, 1.7)	0 (0, 1.3)	0 (0, 2.1)	0 (0, 0)	0 (0, 1.6)
min - max	0 -- 4	0 -- 5	0 -- 5	0 -- 7.7	0 -- 4.7	0 -- 2.6	0 -- 7.7
Circulatory syst. Cum. Incidence (%) (pat.)							
mean ± sd	1.3 ± 2.2	2.8 ± 2.7	4.1 ± 2.9	3.9 ± 2.8	4 ± 2.8	3.3 ± 2.5	3.5 ± 2.8
median (Q1, Q3)	0 (0, 2.9)	2.8 (0, 5.1)	4 (2.6, 6.2)	3.7 (2, 5.6)	3.4 (2, 5.6)	3 (1.7, 4.9)	3.3 (1.1, 5.6)
min - max	0 -- 5.6	0 -- 7.9	0 -- 12	0 -- 12	0 -- 11	0 -- 8.9	0 -- 12
Hypertension Cum. Incidence (%) (pat.)							
mean ± sd	2.3 ± 3.8	1.7 ± 2.2	1.6 ± 2.2	1.8 ± 2.4	2.4 ± 2.5	2.5 ± 3	2 ± 2.4
median (Q1, Q3)	0 (0, 3.7)	0 (0, 3.8)	0 (0, 3.6)	0 (0, 3.6)	1.8 (0, 4.8)	1.5 (0, 3.1)	0 (0, 3.8)
min - max	0 -- 12	0 -- 8.8	0 -- 6	0 -- 8.3	0 -- 7.3	0 -- 8.9	0 -- 12
Metabolic Cum. Incidence (%) (pat.)							
mean ± sd	2.1 ± 2.2	1.9 ± 2.4	3.1 ± 2.6	3.4 ± 3.1	3.4 ± 2.3	2.3 ± 2.2	2.8 ± 2.6
median (Q1, Q3)	1.5 (0, 4.1)	0 (0, 4)	3.4 (0, 4.8)	3.1 (0, 4.9)	3.3 (2, 4.4)	2.1 (0, 3.7)	2.8 (0, 4.5)
min - max	0 -- 5.3	0 -- 7.5	0 -- 7	0 -- 11	0 -- 9.6	0 -- 6.9	0 -- 11
Diabetes Cum. Incidence (%) (pat.)							
mean ± sd	0.25 ± 0.8	0.63 ± 1.2	0.95 ± 1.2	1.3 ± 1.7	1.7 ± 1.7	1.3 ± 1.3	1.1 ± 1.5
median (Q1, Q3)	0 (0, 0)	0 (0, 0.97)	0 (0, 2)	0 (0, 2.4)	1.7 (0, 3)	1.1 (0, 2.4)	0 (0, 2.3)
min - max	0 -- 2.5	0 -- 4	0 -- 3.7	0 -- 6.1	0 -- 6.1	0 -- 3.7	0 -- 6.1
Musculoskelet. syst. Cum. Incidence (%) (pat.)							
mean ± sd	5.4 ± 3.4	6.9 ± 5	8.3 ± 4.7	7.4 ± 4.6	6.3 ± 2.9	6.8 ± 3.4	7 ± 4.3
median (Q1, Q3)	5 (2.9, 8.5)	7.5 (1.8, 10)	8.6 (5.5, 11)	6.1 (4.7, 9.4)	6 (4.3, 8.2)	6.9 (5.6, 9.9)	6.8 (4.3, 9.7)
min - max	0 -- 11	0 -- 19	0 -- 19	0 -- 21	0 -- 13	0 -- 13	0 -- 21
Neoplasm Cum. Incidence (%) (pat.)							
mean ± sd	0.21 ± 0.66	0.41 ± 0.86	1.2 ± 1.5	0.92 ± 1.3	0.51 ± 1.2	0.41 ± 0.97	0.64 ± 1.2
median (Q1, Q3)	0 (0, 0)	0 (0, 0)	0 (0, 2.7)	0 (0, 1.9)	0 (0, 0)	0 (0, 0)	0 (0, 1.1)
min - max	0 -- 2.1	0 -- 3.2	0 -- 4.2	0 -- 4.4	0 -- 5.2	0 -- 2.9	0 -- 5.2
Nervous syst. Cum. Incidence (%) (pat.)							
mean ± sd	2.3 ± 3	2.5 ± 2.6	3.4 ± 1.9	3.8 ± 2.5	2.5 ± 2.2	2.6 ± 2	2.9 ± 2.4
median (Q1, Q3)	0 (0, 5)	2.7 (0, 4.4)	3.7 (3, 4.5)	4 (2.3, 5.5)	2.5 (0, 3.5)	3.1 (0, 3.6)	3 (0, 4.4)

min - max	0 -- 7.4	0 -- 9.4	0 -- 7.4	0 -- 8.3	0 -- 9.7	0 -- 7.4	0 -- 9.7
Ear.mastoid Cum. Incidence (%) (pat.)							
mean ± sd	0.45 ± 1	0.83 ± 1.3	1.3 ± 1.8	1.1 ± 1.4	1 ± 1.2	1.3 ± 1.3	1 ± 1.4
median (Q1, Q3)	0 (0, 0)	0 (0, 1.7)	0 (0, 2.1)	0 (0, 2.2)	0 (0, 1.8)	1.3 (0, 2.2)	0 (0, 2)
min - max	0 -- 2.9	0 -- 4.8	0 -- 7.6	0 -- 4.8	0 -- 3.7	0 -- 3.9	0 -- 7.6
Eye.adnexa Cum. Incidence (%) (pat.)							
mean ± sd	0.62 ± 1.3	1.9 ± 2.2	1.9 ± 2.1	1.8 ± 2.1	1.8 ± 2	1.7 ± 1.8	1.8 ± 2.1
median (Q1, Q3)	0 (0, 0)	0.85 (0, 3.5)	0.74 (0, 3.5)	1.4 (0, 3.3)	1.5 (0, 2.7)	1.6 (0, 3)	1.4 (0, 3.3)
min - max	0 -- 3.3	0 -- 7.5	0 -- 5.8	0 -- 7.4	0 -- 9.6	0 -- 5.5	0 -- 9.6
Pregn.condition Cum. Incidence (%) (pat.)							
mean ± sd	0.99 ± 2.3	2.2 ± 3.5	4.8 ± 3.3	4.8 ± 2.9	3.1 ± 3.1	1.3 ± 1.6	3.2 ± 3.3
median (Q1, Q3)	0 (0, 0)	0 (0, 5.1)	4.8 (2.7, 7.5)	5.2 (2.7, 6.5)	2.8 (0, 4.6)	0 (0, 3)	2.7 (0, 5.7)
min - max	0 -- 7	0 -- 12	0 -- 10	0 -- 12	0 -- 11	0 -- 4.3	0 -- 12
Psych.condition Cum. Incidence (%) (pat.)							
mean ± sd	7.4 ± 4.4	6.7 ± 5.2	9.6 ± 5	9.9 ± 4.8	6.6 ± 4.2	6.7 ± 4.5	7.7 ± 5
median (Q1, Q3)	5.9 (4.4, 9.6)	6.2 (2.7, 9.3)	11 (5.5, 13)	10 (5.4, 13)	5.7 (3.8, 8.9)	6 (3.4, 8.6)	7.1 (4.3, 12)
min - max	2.9 -- 17	0 -- 22	0 -- 19	0 -- 21	0 -- 18	0 -- 17	0 -- 22
Psych.prescrip. Cum. Incidence (%) (pat.)							
mean ± sd	0.87 ± 2.1	1.9 ± 3.2	3.6 ± 3.3	2.2 ± 2.6	0.63 ± 1	0.73 ± 1.4	1.7 ± 2.7
median (Q1, Q3)	0 (0, 0)	0 (0, 2.9)	3.1 (0.4, 6.7)	1.6 (0, 2.8)	0 (0, 1.2)	0 (0, 0.92)	0 (0, 2.7)
min - max	0 -- 6.5	0 -- 15	0 -- 11	0 -- 13	0 -- 3.5	0 -- 4.2	0 -- 15
Respiratory syst. Cum. Incidence (%) (pat.)							
mean ± sd	13 ± 7.2	13 ± 8.4	12 ± 9.8	8.3 ± 5.6	12 ± 6.7	14 ± 6.1	12 ± 7.7
median (Q1, Q3)	11 (7.7, 20)	13 (6.9, 20)	9.4 (3.3, 18)	8.2 (3.9, 11)	11 (6.2, 16)	16 (11, 18)	11 (6.3, 17)
min - max	4.4 -- 24	0 -- 39	0 -- 33	0 -- 21	0 -- 27	0 -- 22	0 -- 39
Genitourinary syst. Cum. Incidence (%) (pat.)							
mean ± sd	1 ± 1.7	2.1 ± 2.3	4.4 ± 2.6	4.6 ± 2.4	2.8 ± 2.3	2.4 ± 1.7	3.1 ± 2.5
median (Q1, Q3)	0 (0, 2.2)	1.1 (0, 4.3)	4.8 (2.9, 6.2)	4.9 (3, 6.1)	2.7 (0, 4.9)	3 (0, 3.6)	3.3 (0, 5)
min - max	0 -- 4.1	0 -- 6.1	0 -- 8.9	0 -- 12	0 -- 7	0 -- 5.5	0 -- 12
time							
mean ± sd	2.5 ± 0.71	9.4 ± 3.4	20 ± 2.8	34 ± 3.6	46 ± 3.4	54 ± 1.2	28 ± 17
median (Q1, Q3)	3 (2, 3)	9.5 (6, 12)	20 (18, 22)	34 (31, 37)	45 (43, 49)	54 (52, 55)	29 (12, 43)
min - max	1 -- 3	4 -- 15	16 -- 26	28 -- 39	40 -- 51	52 -- 55	1 -- 55
occupancy							
mean ± sd	287 ± 228	393 ± 314	455 ± 339	367 ± 271	495 ± 537	566 ± 406	431 ± 386
median (Q1, Q3)	224 (161, 297)	266 (212, 426)	341 (223, 567)	296 (180, 464)	314 (248, 474)	386 (279, 792)	292 (220, 497)
min - max	93 -- 891	88 -- 1421	114 -- 1516	62 -- 1197	124 -- 3110	197 -- 1533	62 -- 3110
% male (occup.)							
mean ± sd	7.1 ± 1	7 ± 1.1	5.9 ± 1.4	6.2 ± 0.84	6.4 ± 1.1	7.6 ± 0.9	6.6 ± 1.2
median (Q1, Q3)	7 (6.1, 7.8)	7.3 (6.2, 7.8)	6.4 (4.6, 6.7)	6.3 (5.6, 6.8)	6.7 (6, 7.1)	7.4 (6.7, 8.5)	6.6 (6, 7.5)
min - max	5.9 -- 9	4.2 -- 9.2	3.3 -- 8.7	4.8 -- 7.9	3.3 -- 8.5	6.3 -- 8.9	3.3 -- 9.2
% adults (occup.)							
mean ± sd	7.9 ± 0.74	7.9 ± 0.84	7.5 ± 0.94	7.2 ± 0.68	7.1 ± 0.91	7.8 ± 0.86	7.5 ± 0.92
median (Q1, Q3)	8 (7.2, 8.5)	7.8 (7.3, 8.7)	7.7 (6.7, 8.1)	7.2 (6.6, 7.7)	7 (6.6, 7.8)	7.5 (7.2, 8.6)	7.5 (6.9, 8.1)
min - max	6.9 -- 9.1	6.2 -- 9.4	5.8 -- 9.5	5.3 -- 8.5	5 -- 8.6	6.5 -- 9.2	5 -- 9.5
Disability Cum. Incidence (%) (occup.)							
mean ± sd	0.33 ± 1	0.65 ± 1	0.85 ± 1.5	1.6 ± 2.6	1 ± 1.8	0.93 ± 1.4	0.96 ± 1.7
median (Q1, Q3)	0 (0, 0)	0 (0, 1.2)	0 (0, 1.4)	0 (0, 2.6)	0.2 (0, 1.4)	0 (0, 1.9)	0 (0, 1.4)
min - max	0 -- 3.3	0 -- 3.5	0 -- 5.5	0 -- 11	0 -- 11	0 -- 3.8	0 -- 11
Skin Cum. Incidence (%) (occup.)							
mean ± sd	1.3 ± 1.6	2.2 ± 2	2.9 ± 2.1	3.2 ± 2.4	2.8 ± 1.8	4 ± 3	2.8 ± 2.2
median (Q1, Q3)	0.87 (0, 2)	1.9 (0, 3.6)	2.9 (1.5, 4.2)	2.7 (1.7, 4.3)	2.8 (1.7, 4.1)	3.6 (2.9, 4.8)	2.6 (1.3, 4)

min - max	0 -- 5.1	0 -- 6.4	0 -- 8.4	0 -- 11	0 -- 6.7	0 -- 13	0 -- 13
Cons.ext.causes Cum. Incidence (%) (occup.)							
mean ± sd	1.6 ± 2.2	2.2 ± 1.9	2.6 ± 2.4	3.1 ± 2.7	2.5 ± 2	2 ± 1.5	2.5 ± 2.2
median (Q1, Q3)	0.68 (0, 2.6)	2.3 (0, 3.6)	2.3 (0.72, 3.2)	2.8 (1, 4.5)	2 (1.1, 3.9)	1.8 (0.97, 3.3)	2.2 (0.83, 3.8)
min - max	0 -- 6.5	0 -- 8	0 -- 9	0 -- 10	0 -- 8.1	0 -- 4.6	0 -- 10
Digestive syst. Cum. Incidence (%) (occup.)							
mean ± sd	2.9 ± 1.3	3.7 ± 3	4.5 ± 2.4	6.5 ± 3.3	5.7 ± 3.2	5.4 ± 2.9	5 ± 3.1
median (Q1, Q3)	3.1 (2.2, 3.2)	3.4 (1.7, 5.3)	4.6 (2.9, 5.5)	6.3 (4.4, 8.2)	5 (3.5, 7)	6.6 (2.2, 7.4)	4.7 (2.8, 6.9)
min - max	0.77 -- 5.3	0 -- 11	0 -- 10	0 -- 14	0 -- 16	0 -- 9.4	0 -- 16
Blood Cum. Incidence (%) (occup.)							
mean ± sd	0.34 ± 0.72	0.36 ± 0.71	0.66 ± 0.82	0.44 ± 0.79	0.23 ± 0.44	0.18 ± 0.36	0.37 ± 0.68
median (Q1, Q3)	0 (0, 0)	0 (0, 0)	0.25 (0, 1.1)	0 (0, 0.94)	0 (0, 0.19)	0 (0, 0)	0 (0, 0.6)
min - max	0 -- 1.8	0 -- 2.8	0 -- 2.9	0 -- 3.1	0 -- 1.6	0 -- 1	0 -- 3.1
Inf.diseases Cum. Incidence (%) (occup.)							
mean ± sd	4.4 ± 3.1	4.4 ± 3.8	4.8 ± 3.4	5.8 ± 4	6.9 ± 4.3	6.1 ± 3.7	5.5 ± 4
median (Q1, Q3)	4.1 (1.9, 5.8)	3.2 (0.83, 7.9)	4.1 (2.7, 6.4)	5.1 (2.8, 8.3)	5.9 (4.1, 8.4)	5.4 (2.5, 9.1)	4.9 (2.5, 8)
min - max	0 -- 11	0 -- 12	0 -- 14	0 -- 14	0 -- 18	0.49 -- 13	0 -- 18
Inf.notify Cum. Incidence (%) (occup.)							
mean ± sd	0.3 ± 0.96	0.44 ± 0.77	0.51 ± 0.83	0.44 ± 0.83	0.55 ± 0.76	0.25 ± 0.53	0.46 ± 0.78
median (Q1, Q3)	0 (0, 0)	0 (0, 0.78)	0 (0, 0.88)	0 (0, 0.84)	0 (0, 1)	0 (0, 0)	0 (0, 0.87)
min - max	0 -- 3	0 -- 2.7	0 -- 3.8	0 -- 3.3	0 -- 3	0 -- 1.7	0 -- 3.8
Circulatory syst. Cum. Incidence (%) (occup.)							
mean ± sd	0.88 ± 1.6	1.6 ± 1.7	2 ± 1.7	2.5 ± 2	2.3 ± 1.7	1.8 ± 1.5	2 ± 1.8
median (Q1, Q3)	0 (0, 1.3)	1.4 (0, 2.5)	1.5 (1.1, 2.8)	1.9 (1.1, 3.8)	2 (1.1, 3.3)	1.5 (0.54, 3)	1.6 (0.51, 3)
min - max	0 -- 4.3	0 -- 5.3	0 -- 7	0 -- 7.2	0 -- 6.7	0 -- 5.1	0 -- 7.2
Hypertension Cum. Incidence (%) (occup.)							
mean ± sd	1.9 ± 2.9	1.1 ± 1.6	0.81 ± 1.2	1.3 ± 1.7	1.4 ± 1.4	1.3 ± 1.6	1.2 ± 1.6
median (Q1, Q3)	0 (0, 3.1)	0 (0, 2.3)	0 (0, 1.9)	0 (0, 2.3)	0.97 (0, 2.7)	0.75 (0, 2.1)	0 (0, 2.2)
min - max	0 -- 8.6	0 -- 7.1	0 -- 4.3	0 -- 4.8	0 -- 4.6	0 -- 5.1	0 -- 8.6
Metabolic Cum. Incidence (%) (occup.)							
mean ± sd	0.95 ± 1.1	1.1 ± 1.5	1.6 ± 1.5	2.2 ± 2.3	2 ± 1.5	1.2 ± 1.3	1.6 ± 1.7
median (Q1, Q3)	0.51 (0, 1.8)	0 (0, 2.1)	1.5 (0, 2.6)	1.7 (0, 2.9)	1.8 (1.1, 2.4)	0.86 (0, 2.1)	1.4 (0, 2.4)
min - max	0 -- 3.1	0 -- 4.8	0 -- 5.2	0 -- 7.9	0 -- 7	0 -- 4.2	0 -- 7.9
Diabetes Cum. Incidence (%) (occup.)							
mean ± sd	0.19 ± 0.6	0.37 ± 0.68	0.49 ± 0.64	0.9 ± 1.2	0.98 ± 1	0.69 ± 0.74	0.66 ± 0.92
median (Q1, Q3)	0 (0, 0)	0 (0, 0.6)	0 (0, 0.97)	0 (0, 1.6)	0.91 (0, 1.6)	0.45 (0, 1.2)	0 (0, 1.2)
min - max	0 -- 1.9	0 -- 2.2	0 -- 2	0 -- 4.2	0 -- 4.4	0 -- 2.2	0 -- 4.4
Musculoskelet. syst. Cum. Incidence (%) (occup.)							
mean ± sd	3.1 ± 2.3	3.5 ± 3	4.1 ± 3.1	4.9 ± 3.7	3.6 ± 2	3.3 ± 1.9	3.9 ± 2.9
median (Q1, Q3)	3.1 (1.4, 4.4)	3.1 (0.62, 6.2)	3.9 (2.1, 5.1)	3.8 (2.7, 6.5)	3.2 (2.4, 4.5)	3.4 (2.1, 4.2)	3.3 (2, 5.1)
min - max	0 -- 8	0 -- 11	0 -- 14	0 -- 16	0 -- 11	0 -- 7.1	0 -- 16
Neoplasm Cum. Incidence (%) (occup.)							
mean ± sd	0.16 ± 0.5	0.3 ± 0.63	0.59 ± 0.81	0.59 ± 0.78	0.27 ± 0.66	0.2 ± 0.49	0.38 ± 0.69
median (Q1, Q3)	0 (0, 0)	0 (0, 0)	0 (0, 1.2)	0 (0, 1.3)	0 (0, 0)	0 (0, 0)	0 (0, 0.64)
min - max	0 -- 1.6	0 -- 2.6	0 -- 2.5	0 -- 2.2	0 -- 3.1	0 -- 1.8	0 -- 3.1
Nervous syst. Cum. Incidence (%) (occup.)							
mean ± sd	1 ± 1.5	1.4 ± 1.6	1.6 ± 0.93	2.3 ± 1.8	1.4 ± 1.3	1.3 ± 0.99	1.6 ± 1.5
median (Q1, Q3)	0 (0, 1.8)	1 (0, 2.4)	1.7 (1.4, 2.1)	2 (1, 3.6)	1.2 (0, 1.9)	1.2 (0, 2)	1.5 (0, 2.3)

min - max	0 -- 3.8	0 -- 6.9	0 -- 4	0 -- 7.6	0 -- 6.1	0 -- 3.1	0 -- 7.6
Ear.mastoid Cum. Incidence (%) (occup.)							
mean ± sd	0.25 ± 0.54	0.45 ± 0.65	0.63 ± 0.85	0.74 ± 1	0.56 ± 0.71	0.61 ± 0.61	0.56 ± 0.78
median (Q1, Q3)	0 (0, 0)	0 (0, 1)	0 (0, 1.1)	0 (0, 1.1)	0 (0, 1)	0.66 (0, 1.2)	0 (0, 1.1)
min - max	0 -- 1.3	0 -- 2.1	0 -- 3.1	0 -- 3.8	0 -- 2.9	0 -- 1.6	0 -- 3.8
Eye.adnexa Cum. Incidence (%) (occup.)							
mean ± sd	0.38 ± 0.84	1 ± 1.2	0.98 ± 1.2	1 ± 1.2	1 ± 1.1	0.81 ± 0.85	0.98 ± 1.1
median (Q1, Q3)	0 (0, 0)	0.29 (0, 2)	0.47 (0, 1.7)	0.65 (0, 2.4)	0.94 (0, 1.6)	0.73 (0, 1.6)	0.69 (0, 1.7)
min - max	0 -- 2.5	0 -- 4.9	0 -- 4.2	0 -- 3.6	0 -- 4.5	0 -- 2.5	0 -- 4.9
Pregn.condition Cum. Incidence (%) (occup.)							
mean ± sd	0.66 ± 1.7	1.6 ± 2.6	2.3 ± 1.8	3 ± 2.1	2 ± 2.3	0.68 ± 0.89	2 ± 2.3
median (Q1, Q3)	0 (0, 0)	0 (0, 3.1)	2.4 (1.1, 3.3)	2.6 (1.4, 4.6)	1.3 (0, 2.9)	0 (0, 1.2)	1.2 (0, 3.2)
min - max	0 -- 5.3	0 -- 9	0 -- 6.6	0 -- 7.7	0 -- 8.5	0 -- 2.5	0 -- 9
Psych.condition Cum. Incidence (%) (occup.)							
mean ± sd	3.8 ± 1.9	3.5 ± 2.9	4.5 ± 2.7	6.2 ± 3.6	3.7 ± 2.8	3.4 ± 2.4	4.2 ± 3.1
median (Q1, Q3)	4.1 (1.8, 5.6)	2.9 (1.4, 5.5)	4.9 (2.6, 5.8)	5.7 (3, 9.1)	3.1 (2, 4.9)	3.4 (1.5, 5)	3.9 (1.9, 5.7)
min - max	1 -- 5.9	0 -- 12	0 -- 12	0 -- 14	0 -- 15	0 -- 9.4	0 -- 15
Psych.prescrip. Cum. Incidence (%) (occup.)							
mean ± sd	0.44 ± 0.93	0.9 ± 1.5	1.6 ± 1.4	1.2 ± 1.3	0.39 ± 0.66	0.32 ± 0.62	0.86 ± 1.3
median (Q1, Q3)	0 (0, 0)	0 (0, 1.4)	1.6 (0.2, 2.5)	1 (0, 1.9)	0 (0, 0.59)	0 (0, 0.41)	0 (0, 1.5)
min - max	0 -- 2.3	0 -- 5.8	0 -- 4.4	0 -- 6	0 -- 2.3	0 -- 1.8	0 -- 6
Respiratory syst. Cum. Incidence (%) (occup.)							
mean ± sd	6.8 ± 4.5	7.2 ± 5.9	5.5 ± 4.8	5.1 ± 3.6	6.5 ± 4.1	7 ± 3.6	6.4 ± 4.7
median (Q1, Q3)	6.3 (3.2, 7.7)	6 (2.9, 10)	4 (1.3, 10)	5.1 (2.2, 7.5)	6 (3.6, 9.3)	6.4 (5, 10)	5.6 (3, 9.4)
min - max	2.7 -- 18	0 -- 31	0 -- 14	0 -- 15	0 -- 17	0 -- 15	0 -- 31
Genitourinary syst. Cum. Incidence (%) (occup.)							
mean ± sd	0.67 ± 1.2	1.2 ± 1.5	2.1 ± 1.4	2.8 ± 1.7	1.6 ± 1.4	1.2 ± 0.9	1.7 ± 1.6
median (Q1, Q3)	0 (0, 1.4)	0.39 (0, 2.4)	2.2 (1.2, 2.8)	2.8 (1.8, 4.1)	1.5 (0, 2.6)	1.3 (0, 1.9)	1.7 (0, 2.8)
min - max	0 -- 3.1	0 -- 5.2	0 -- 5.3	0 -- 7.2	0 -- 5.1	0 -- 2.5	0 -- 7.2
Peri-pandemic Inc.							
mean ± sd	0 ± 0	0 ± 0	0.79 ± 0.41	1 ± 0	1 ± 0	1 ± 0	0.64 ± 0.48
median (Q1, Q3)	0 (0, 0)	0 (0, 0)	1 (1, 1)	1 (1, 1)	1 (1, 1)	1 (1, 1)	1 (0, 1)
min - max	0 -- 0	0 -- 0	0 -- 1	1 -- 1	1 -- 1	1 -- 1	0 -- 1
Peri-pandemic time trend							
mean ± sd	0 ± 0	0 ± 0	3.2 ± 2.6	17 ± 3.6	29 ± 3.4	37 ± 1.2	14 ± 14
median (Q1, Q3)	0 (0, 0)	0 (0, 0)	3 (1, 5)	17 (14, 20)	28 (26, 32)	37 (35, 38)	12 (0, 26)
min - max	0 -- 0	0 -- 0	0 -- 9	11 -- 22	23 -- 34	35 -- 38	0 -- 38

6.3 Supplementary Table S3: Sociodemographic characteristics of patients and occupancy in refugee centres per month, number of cases of 21 indicators per month as well as incidence proportions of 21 indicators per month, 2018 – 2023, N = 155,546 refugees.

Table S3: Sociodemographic characteristics of patients and occupancy in refugee centres per month, number of cases of 21 indicators per month as well as incidence proportions of 21 indicators per month, 2018 – 2023, N = 155,546 refugees.

Variable	2018	2019	2020	2021	2022	2023	Total
Centre-months cluster	17	88	55	89	97	32	378
REC	12 (71%)	67 (76%)	33 (60%)	39 (44%)	54 (56%)	20 (62%)	225 (60%)
REG	5 (29%)	21 (24%)	22 (40%)	50 (56%)	43 (44%)	12 (38%)	153 (40%)
% male (pat.)							
N	17	88	55	89	97	32	378
mean ± sd	6.2 ± 0.9	6.2 ± 1.3	5.6 ± 1.2	5.8 ± 1.2	5.7 ± 1.3	6.6 ± 1.4	5.9 ± 1.3
median (Q1, Q3)	6.1 (5.5, 6.4)	6.3 (5.3, 7.2)	5.6 (4.6, 6.4)	6 (5, 6.7)	6 (5, 6.5)	7.1 (6.4, 7.4)	6 (5.3, 6.9)
min - max	5.3 -- 9	2.3 -- 9	2.9 -- 8.7	2 -- 8.5	2.4 -- 8.6	3.1 -- 8.8	2 -- 9
% adults (pat.)							
N	17	88	55	89	97	32	378
mean ± sd	8.2 ± 0.94	8.2 ± 0.93	7.9 ± 1	7.7 ± 0.85	7.6 ± 0.92	8 ± 0.91	7.9 ± 0.95
median (Q1, Q3)	8.3 (7.4, 8.8)	8.4 (7.7, 8.9)	7.9 (7.3, 8.8)	7.9 (7.2, 8.3)	7.7 (7.1, 8.3)	8.2 (7.7, 8.6)	7.9 (7.3, 8.6)
min - max	6.4 -- 9.7	6.1 -- 10	4.8 -- 9.7	5.8 -- 9.3	4.6 -- 9.2	4.8 -- 9	4.6 -- 10
% Nigerian pat.							
N	17	88	55	89	97	32	378
mean ± sd	0.14 ± 0.11	0.27 ± 0.22	0.073 ± 0.057	0.029 ± 0.045	0.026 ± 0.033	0.031 ± 0.031	0.096 ± 0.15
median (Q1, Q3)	0.12 (0.054, 0.2)	0.25 (0.068, 0.38)	0.074 (0.03, 0.1)	0.023 (0, 0.04)	0.02 (0, 0.035)	0.023 (0, 0.051)	0.038 (0, 0.093)
min - max	0 -- 0.37	0 -- 0.92	0 -- 0.29	0 -- 0.35	0 -- 0.18	0 -- 0.12	0 -- 0.92
% Afghani pat.							
N	17	88	55	89	97	32	378
mean ± sd	0.093 ± 0.098	0.096 ± 0.11	0.19 ± 0.12	0.27 ± 0.21	0.2 ± 0.14	0.21 ± 0.12	0.19 ± 0.16
median (Q1, Q3)	0.052 (0.035, 0.14)	0.051 (0, 0.21)	0.18 (0.068, 0.27)	0.17 (0.09, 0.45)	0.17 (0.1, 0.27)	0.21 (0.12, 0.28)	0.15 (0.061, 0.27)
min - max	0 -- 0.39	0 -- 0.39	0 -- 0.44	0 -- 0.84	0 -- 0.57	0 -- 0.46	0 -- 0.84
% Syrian pat.							
N	17	88	55	89	97	32	378
mean ± sd	0.066 ± 0.044	0.036 ± 0.042	0.068 ± 0.09	0.1 ± 0.13	0.061 ± 0.069	0.075 ± 0.091	0.068 ± 0.091
median (Q1, Q3)	0.069 (0.05, 0.083)	0.025 (0, 0.063)	0.022 (0, 0.12)	0.04 (0, 0.18)	0.038 (0, 0.1)	0.055 (0.0054, 0.098)	0.038 (0, 0.1)
min - max	0 -- 0.15	0 -- 0.16	0 -- 0.38	0 -- 0.56	0 -- 0.28	0 -- 0.47	0 -- 0.56
% Iraqi pat.							
N	17	88	55	89	97	32	378
mean ± sd	0.097 ± 0.041	0.044 ± 0.044	0.049 ± 0.049	0.042 ± 0.042	0.038 ± 0.055	0.0098 ± 0.017	0.042 ± 0.048
median (Q1, Q3)	0.093 (0.071, 0.12)	0.041 (0, 0.08)	0.04 (0, 0.1)	0.033 (0, 0.074)	0.019 (0, 0.049)	0 (0, 0.015)	0.027 (0, 0.073)
min - max	0 -- 0.18	0 -- 0.14	0 -- 0.15	0 -- 0.16	0 -- 0.32	0 -- 0.062	0 -- 0.32
% Turkish pat.							
N	17	88	55	89	97	32	378
mean ± sd	0.045 ± 0.039	0.036 ± 0.042	0.044 ± 0.042	0.03 ± 0.039	0.094 ± 0.14	0.15 ± 0.14	0.06 ± 0.095
median (Q1, Q3)	0.036 (0.022, 0.061)	0.023 (0, 0.059)	0.032 (0, 0.079)	0.022 (0, 0.048)	0.056 (0, 0.11)	0.087 (0.063, 0.21)	0.034 (0, 0.074)
min - max	0 -- 0.14	0 -- 0.14	0 -- 0.18	0 -- 0.19	0 -- 0.66	0 -- 0.61	0 -- 0.66
patients							
N	17	88	55	89	97	32	378
mean ± sd	258 ± 237	223 ± 214	233 ± 184	227 ± 175	270 ± 292	263 ± 216	242 ± 226

median (Q1, Q3)	136 (94, 356)	128 (101, 207)	192 (92, 284)	179 (108, 233)	177 (112, 276)	198 (123, 298)	162 (103, 274)
min - max	68 -- 734	29 -- 934	45 -- 810	36 -- 880	48 -- 1390	64 -- 924	29 -- 1390
Disability Cum. Incidence (%) (pat.)							
mean ± sd	1.4 ± 1.9	1 ± 1.6	1.5 ± 2.4	3.5 ± 4.4	1.7 ± 2.5	1.6 ± 2.6	1.9 ± 3
median (Q1, Q3)	0 (0, 2.1)	0 (0, 2.1)	0 (0, 2.2)	1.5 (0, 6.2)	0 (0, 2.8)	0 (0, 2.4)	0 (0, 3)
min - max	0 -- 4.9	0 -- 6	0 -- 7.3	0 -- 16	0 -- 13	0 -- 8.2	0 -- 16
Skin Cum. Incidence (%) (pat.)							
mean ± sd	3.3 ± 2.9	4.1 ± 3.1	5.8 ± 3.8	4.7 ± 3.2	4.8 ± 3.2	6 ± 4.9	4.8 ± 3.5
median (Q1, Q3)	4.2 (0, 5.3)	4 (0, 6.5)	5.9 (3.6, 8.2)	4.4 (2.4, 6.2)	4.5 (2.9, 6.7)	5.6 (2.5, 8.3)	4.6 (2.4, 6.9)
min - max	0 -- 7.5	0 -- 13	0 -- 16	0 -- 13	0 -- 15	0 -- 22	0 -- 22
Cons.ext.causes Cum. Incidence (%) (pat.)							
mean ± sd	3.3 ± 3	4.1 ± 3.5	4.6 ± 3.6	4.3 ± 3.2	4.1 ± 3.1	3.5 ± 2.9	4.1 ± 3.3
median (Q1, Q3)	3.9 (0, 6)	4.4 (0, 6.7)	4.7 (0, 6.8)	4 (2.2, 6.4)	3.6 (2, 6)	3.6 (0.59, 6.2)	4 (1.2, 6.4)
min - max	0 -- 8.8	0 -- 15	0 -- 12	0 -- 12	0 -- 12	0 -- 9.1	0 -- 15
Digestive syst. Cum. Incidence (%) (pat.)							
mean ± sd	6.4 ± 3	7.8 ± 5	9.3 ± 4.5	9.6 ± 4.5	9.8 ± 5.4	10 ± 5.6	9.1 ± 5
median (Q1, Q3)	6.8 (3.5, 7.9)	8.4 (3.9, 11)	10 (7.3, 13)	9.4 (7, 12)	9.3 (6, 13)	9.8 (6.4, 13)	9.1 (6.2, 12)
min - max	2 -- 12	0 -- 22	0 -- 17	0 -- 23	0 -- 28	0 -- 24	0 -- 28
Blood Cum. Incidence (%) (pat.)							
mean ± sd	0.8 ± 1.2	0.77 ± 1.6	1.4 ± 1.6	0.5 ± 1.1	0.48 ± 0.91	0.38 ± 0.8	0.69 ± 1.3
median (Q1, Q3)	0 (0, 1.4)	0 (0, 1.1)	1.2 (0, 2.6)	0 (0, 0)	0 (0, 0.99)	0 (0, 0)	0 (0, 1.3)
min - max	0 -- 4.4	0 -- 9.2	0 -- 5	0 -- 4.7	0 -- 5.2	0 -- 3.3	0 -- 9.2
Inf.diseases Cum. Incidence (%) (pat.)							
mean ± sd	9.2 ± 4.8	8.4 ± 6.7	9.4 ± 5.5	9 ± 5.1	11 ± 6.7	12 ± 5.6	9.6 ± 6.1
median (Q1, Q3)	9 (5.9, 14)	8.5 (3, 12)	9.1 (5.5, 14)	9.5 (5.3, 13)	9.7 (5.2, 14)	12 (6.5, 16)	9.3 (5.1, 14)
min - max	0 -- 17	0 -- 25	0 -- 24	0 -- 20	0 -- 34	1.2 -- 22	0 -- 34
Inf.notify Cum. Incidence (%) (pat.)							
mean ± sd	0.78 ± 1.4	0.79 ± 1.3	0.98 ± 1.4	0.66 ± 1.3	1 ± 1.5	0.44 ± 0.83	0.81 ± 1.4
median (Q1, Q3)	0 (0, 1.3)	0 (0, 1.7)	0 (0, 1.7)	0 (0, 0.89)	0 (0, 2.1)	0 (0, 0.59)	0 (0, 1.6)
min - max	0 -- 4	0 -- 6.1	0 -- 5	0 -- 7.7	0 -- 9	0 -- 2.6	0 -- 9
Circulatory syst. Cum. Incidence (%) (pat.)							
mean ± sd	2.7 ± 2.8	2.9 ± 2.7	4 ± 2.8	3.7 ± 2.9	3.9 ± 2.8	3.4 ± 3.2	3.5 ± 2.9
median (Q1, Q3)	2.2 (0, 5.6)	3.1 (0, 5.1)	4.1 (2.1, 6.1)	3.2 (1.8, 5.6)	3.5 (2, 5.6)	2.8 (0, 5.5)	3.5 (0, 5.6)
min - max	0 -- 6.9	0 -- 9.2	0 -- 12	0 -- 12	0 -- 11	0 -- 11	0 -- 12
Hypertension Cum. Incidence (%) (pat.)							
mean ± sd	2.5 ± 3	1.6 ± 2.1	1.7 ± 2	1.9 ± 2.4	2.2 ± 2.5	2.6 ± 3.1	2 ± 2.4
median (Q1, Q3)	3.1 (0, 3.5)	0 (0, 3.3)	0 (0, 3.5)	0.73 (0, 3.5)	1.3 (0, 4.1)	1.3 (0, 5)	0.71 (0, 3.7)
min - max	0 -- 12	0 -- 8.8	0 -- 6	0 -- 9.2	0 -- 7.3	0 -- 8.9	0 -- 12
Metabolic Cum. Incidence (%) (pat.)							
mean ± sd	3.4 ± 2.6	2 ± 2.5	3.2 ± 2.6	3.4 ± 2.7	3.2 ± 2.4	2.9 ± 2.5	2.9 ± 2.6
median (Q1, Q3)	4.1 (0, 4.4)	0 (0, 4.4)	3.7 (0, 5.4)	3.1 (1.4, 4.9)	3.3 (1.4, 4.5)	2.8 (0.59, 4.6)	3 (0, 4.7)
min - max	0 -- 8.1	0 -- 7.5	0 -- 8	0 -- 11	0 -- 9.6	0 -- 8.4	0 -- 11
Diabetes Cum. Incidence (%) (pat.)							
mean ± sd	1.2 ± 1.4	0.67 ± 1.2	1.1 ± 1.2	1.4 ± 1.7	1.5 ± 1.6	1.2 ± 1.4	1.2 ± 1.5
median (Q1, Q3)	0 (0, 2.5)	0 (0, 1.3)	1.2 (0, 2.2)	0.69 (0, 2.5)	1.1 (0, 2.8)	0.94 (0, 2.4)	0 (0, 2.3)
min - max	0 -- 3.8	0 -- 4	0 -- 3.7	0 -- 6.1	0 -- 6.1	0 -- 4.7	0 -- 6.1
Musculoskelet. syst. Cum. Incidence (%) (pat.)							
mean ± sd	6.5 ± 4.1	6.9 ± 4.8	7.6 ± 4.6	6.7 ± 4.5	5.7 ± 3.1	6 ± 3.4	6.6 ± 4.2
median (Q1, Q3)	5.6 (3.7, 9.7)	7.2 (3.1, 10)	7.7 (4.9, 11)	5.9 (4.3, 8.9)	5.6 (3.9, 7.8)	5.8 (3.3, 8.7)	6.2 (4, 9.4)

min - max	0 -- 14	0 -- 19	0 -- 19	0 -- 21	0 -- 13	0 -- 13	0 -- 21
Neoplasm Cum. Incidence (%) (pat.)							
mean ± sd	0.48 ± 0.93	0.63 ± 1.4	1.1 ± 1.4	0.65 ± 1.1	0.59 ± 1.3	0.67 ± 1.4	0.7 ± 1.3
median (Q1, Q3)	0 (0, 0)	0 (0, 0.67)	0 (0, 2.6)	0 (0, 1.3)	0 (0, 0)	0 (0, 0)	0 (0, 1.3)
min - max	0 -- 2.5	0 -- 9.6	0 -- 4.2	0 -- 4.4	0 -- 5.2	0 -- 5.7	0 -- 9.6
Nervous syst. Cum. Incidence (%) (pat.)							
mean ± sd	2.5 ± 2.7	2.8 ± 2.7	3.2 ± 2.1	3.2 ± 2.5	2.5 ± 2.2	2.3 ± 2.4	2.8 ± 2.4
median (Q1, Q3)	2.1 (0, 5)	3 (0, 4.5)	3.7 (1.4, 4.5)	3.5 (0, 5)	2.5 (0, 3.7)	2.7 (0, 3.7)	3 (0, 4.4)
min - max	0 -- 7.4	0 -- 12	0 -- 7.4	0 -- 8.4	0 -- 9.7	0 -- 9.7	0 -- 12
Ear.mastoid Cum. Incidence (%) (pat.)							
mean ± sd	0.65 ± 0.98	0.78 ± 1.2	1.3 ± 1.8	0.92 ± 1.4	0.94 ± 1.2	1.2 ± 1.3	0.95 ± 1.3
median (Q1, Q3)	0 (0, 1.3)	0 (0, 1.5)	0 (0, 2.1)	0 (0, 1.6)	0 (0, 1.7)	1.3 (0, 2.1)	0 (0, 1.8)
min - max	0 -- 2.9	0 -- 4.8	0 -- 7.6	0 -- 4.8	0 -- 5	0 -- 3.9	0 -- 7.6
Eye.adnexa Cum. Incidence (%) (pat.)							
mean ± sd	1.1 ± 1.4	1.7 ± 2.1	1.8 ± 1.9	1.4 ± 1.8	1.5 ± 1.8	1.6 ± 1.8	1.6 ± 1.9
median (Q1, Q3)	0 (0, 2.8)	0 (0, 3.2)	1.5 (0, 3.4)	0 (0, 2.9)	1.3 (0, 2.4)	1.2 (0, 3.1)	0.34 (0, 3)
min - max	0 -- 3.5	0 -- 7.5	0 -- 5.8	0 -- 7.4	0 -- 9.6	0 -- 5.9	0 -- 9.6
Pregn.condition Cum. Incidence (%) (pat.)							
mean ± sd	1.7 ± 2.5	2.7 ± 3.8	4.4 ± 3.1	4.6 ± 2.8	3.9 ± 3.5	2.5 ± 2.5	3.6 ± 3.3
median (Q1, Q3)	0 (0, 3.5)	0 (0, 5.6)	4.8 (2.2, 7.3)	4.9 (2.6, 6.3)	3.3 (0, 5.5)	2.3 (0, 4.2)	3.3 (0, 5.9)
min - max	0 -- 7	0 -- 14	0 -- 10	0 -- 12	0 -- 16	0 -- 9.4	0 -- 16
Psych.condition Cum. Incidence (%) (pat.)							
mean ± sd	7.6 ± 4.2	6.6 ± 5.1	9.4 ± 4.9	10 ± 5.2	7.2 ± 4.4	7.2 ± 5.1	8.1 ± 5
median (Q1, Q3)	7.4 (5.5, 8.6)	6.5 (2.8, 9.4)	9.7 (5.5, 14)	10 (5.3, 14)	6.3 (4.5, 9.6)	6.6 (3.7, 10)	7.6 (4.3, 12)
min - max	2.5 -- 17	0 -- 22	0 -- 19	0 -- 24	0 -- 21	0 -- 21	0 -- 24
Psych.prescrip. Cum. Incidence (%) (pat.)							
mean ± sd	1.1 ± 2	1.8 ± 3	3.7 ± 3.5	1.6 ± 2.4	1 ± 2	1.3 ± 2.4	1.7 ± 2.8
median (Q1, Q3)	0 (0, 0.96)	0 (0, 3.5)	2.7 (0.4, 6.7)	0.69 (0, 2)	0 (0, 1.2)	0 (0, 1.6)	0 (0, 2.7)
min - max	0 -- 6.5	0 -- 15	0 -- 14	0 -- 13	0 -- 9.7	0 -- 10	0 -- 15
Respiratory syst. Cum. Incidence (%) (pat.)							
mean ± sd	14 ± 5.8	14 ± 8.3	11 ± 9.3	8.3 ± 5.4	11 ± 7	14 ± 6.9	11 ± 7.6
median (Q1, Q3)	14 (10, 19)	14 (7.2, 20)	10 (3.3, 17)	8.2 (3.9, 11)	9.9 (5.4, 16)	15 (9.8, 18)	11 (5.4, 17)
min - max	4.4 -- 24	0 -- 39	0 -- 33	0 -- 23	0 -- 27	0 -- 27	0 -- 39
Genitourinary syst. Cum. Incidence (%) (pat.)							
mean ± sd	1.9 ± 2.2	2.5 ± 2.7	3.8 ± 2.6	4 ± 2.5	3.4 ± 2.7	3.6 ± 3.2	3.3 ± 2.7
median (Q1, Q3)	0 (0, 3.9)	2.5 (0, 4.6)	4.3 (1.3, 5.7)	4 (2.2, 5.6)	3.3 (0, 5.7)	3.1 (1.9, 3.9)	3.4 (0, 5.1)
min - max	0 -- 6	0 -- 11	0 -- 8.9	0 -- 12	0 -- 9.7	0 -- 16	0 -- 16
time							
mean ± sd	2.2 ± 0.75	9.8 ± 3.6	21 ± 3.2	34 ± 3.4	45 ± 3.6	54 ± 1.1	30 ± 16
median (Q1, Q3)	2 (2, 3)	10 (7, 13)	20 (18, 24)	35 (31, 37)	45 (42, 48)	54 (52, 54)	32 (14, 43)
min - max	1 -- 3	4 -- 15	16 -- 27	28 -- 39	40 -- 51	52 -- 55	1 -- 55
occupancy							
mean ± sd	414 ± 393	374 ± 303	405 ± 310	303 ± 278	513 ± 649	518 ± 457	411 ± 437
median (Q1, Q3)	288 (220, 403)	262 (194, 422)	276 (178, 555)	212 (126, 356)	272 (205, 462)	308 (224, 758)	259 (171, 462)
min - max	93 -- 1644	41 -- 1421	85 -- 1516	54 -- 1468	18 -- 3110	106 -- 1754	18 -- 3110
% male (occup.)							
mean ± sd	6.9 ± 0.95	6.7 ± 1.2	6.2 ± 1.4	6.3 ± 1	6.2 ± 1.4	6.9 ± 1.6	6.4 ± 1.3
median (Q1, Q3)	6.8 (6.1, 7.5)	6.8 (6, 7.7)	6.4 (4.8, 7.2)	6.5 (5.9, 7)	6.6 (5.5, 7.1)	7.1 (6.5, 7.9)	6.6 (5.8, 7.3)
min - max	5.7 -- 9	3.4 -- 9.2	3.3 -- 9.3	2.6 -- 8.9	2.9 -- 8.9	2.7 -- 8.9	2.6 -- 9.3
% adults (occup.)							
mean ± sd	7.9 ± 0.63	7.9 ± 0.8	7.7 ± 1	7.2 ± 0.8	7.1 ± 0.95	7.8 ± 0.97	7.5 ± 0.94
median (Q1, Q3)	8 (7.5, 8.4)	7.9 (7.3, 8.6)	7.8 (6.8, 8.3)	7.2 (6.6, 7.8)	7.2 (6.6, 7.8)	7.8 (7.1, 8.6)	7.5 (6.9, 8.1)

min - max	6.9 -- 9.1	6.2 -- 9.4	5.8 -- 9.5	4.2 -- 9	5 -- 9.4	5.6 -- 9.2	4.2 -- 9.5
Disability Cum. Incidence (%) (occup.)							
mean ± sd	1.1 ± 1.5	0.66 ± 1	1.1 ± 1.9	4.1 ± 5.9	1.3 ± 3.3	0.86 ± 1.5	1.7 ± 3.7
median (Q1, Q3)	0 (0, 1.9)	0 (0, 1.5)	0 (0, 1.8)	1 (0, 6.5)	0 (0, 1.4)	0 (0, 1.5)	0 (0, 1.9)
min - max	0 -- 4	0 -- 3.5	0 -- 9.5	0 -- 25	0 -- 28	0 -- 5.2	0 -- 28
Skin Cum. Incidence (%) (occup.)							
mean ± sd	2.3 ± 2.8	2.4 ± 2.2	3.4 ± 2.7	4.3 ± 3.8	2.8 ± 1.9	3.2 ± 2.7	3.2 ± 2.8
median (Q1, Q3)	1.8 (0, 2.9)	2 (0, 3.8)	3 (1.6, 4.8)	3.2 (1.7, 6)	2.8 (1.4, 4.2)	3.4 (1.5, 4)	2.7 (1.4, 4.4)
min - max	0 -- 9.5	0 -- 9.9	0 -- 13	0 -- 17	0 -- 6.8	0 -- 13	0 -- 17
Cons.ext.causes Cum. Incidence (%) (occup.)							
mean ± sd	2.5 ± 2.8	2.2 ± 2	2.8 ± 2.6	4.1 ± 3.6	2.5 ± 2.4	1.8 ± 1.5	2.8 ± 2.7
median (Q1, Q3)	1.9 (0, 4.2)	2.2 (0, 3.7)	2.4 (0, 4.4)	3.5 (1.3, 6.9)	2 (1, 3.5)	1.6 (0.38, 3.2)	2.3 (0.38, 4.2)
min - max	0 -- 8.9	0 -- 8	0 -- 9	0 -- 16	0 -- 17	0 -- 4.7	0 -- 17
Digestive syst. Cum. Incidence (%) (occup.)							
mean ± sd	4.6 ± 4.3	4.3 ± 3.3	5.5 ± 3.9	9.3 ± 8.6	6.4 ± 5.4	5.6 ± 2.8	6.3 ± 5.9
median (Q1, Q3)	3.1 (2.4, 5)	3.7 (1.8, 6.8)	5.1 (2.9, 6.9)	7.4 (4.4, 10)	5.1 (3.5, 7.5)	6.4 (2.8, 7.6)	5.3 (2.9, 8.1)
min - max	0.77 -- 18	0 -- 12	0 -- 15	0 -- 46	0 -- 44	0 -- 10	0 -- 46
Blood Cum. Incidence (%) (occup.)							
mean ± sd	0.6 ± 0.8	0.47 ± 0.84	0.9 ± 1.1	0.33 ± 0.74	0.27 ± 0.5	0.22 ± 0.53	0.43 ± 0.79
median (Q1, Q3)	0 (0, 1.1)	0 (0, 1.1)	0.5 (0, 1.7)	0 (0, 0)	0 (0, 0.46)	0 (0, 0)	0 (0, 0.68)
min - max	0 -- 2.1	0 -- 3.4	0 -- 4.4	0 -- 3.6	0 -- 2.4	0 -- 2.5	0 -- 4.4
Inf.diseases Cum. Incidence (%) (occup.)							
mean ± sd	7.2 ± 8.7	4.8 ± 4.4	5.7 ± 4.7	9.3 ± 9.8	6.9 ± 6.2	6.4 ± 3.3	6.8 ± 6.8
median (Q1, Q3)	4.3 (3.5, 6.7)	3.4 (1.2, 8.1)	5 (2.7, 7.5)	7.3 (3.3, 11)	5.2 (3.6, 7.9)	5.8 (4.3, 9)	5.2 (2.8, 8.7)
min - max	0 -- 38	0 -- 21	0 -- 25	0 -- 45	0 -- 50	0.49 -- 13	0 -- 50
Inf.notify Cum. Incidence (%) (occup.)							
mean ± sd	0.71 ± 1.4	0.53 ± 0.88	0.63 ± 0.92	0.57 ± 1.1	0.67 ± 1.4	0.28 ± 0.53	0.58 ± 1.1
median (Q1, Q3)	0 (0, 0.67)	0 (0, 1)	0 (0, 1.1)	0 (0, 0.84)	0 (0, 1)	0 (0, 0.23)	0 (0, 0.97)
min - max	0 -- 4.4	0 -- 3.7	0 -- 3.8	0 -- 4.8	0 -- 11	0 -- 1.7	0 -- 11
Circulatory syst. Cum. Incidence (%) (occup.)							
mean ± sd	2 ± 2.4	1.7 ± 1.8	2.5 ± 2.2	3.8 ± 4.9	2.5 ± 2.4	2 ± 2.2	2.6 ± 3.1
median (Q1, Q3)	1.3 (0, 3.2)	1.4 (0, 3.3)	2.2 (1, 4)	2.5 (0.95, 4.5)	2 (1.1, 3.5)	1.4 (0, 3.3)	1.9 (0, 3.7)
min - max	0 -- 8	0 -- 7.1	0 -- 8.6	0 -- 25	0 -- 17	0 -- 8.6	0 -- 25
Hypertension Cum. Incidence (%) (occup.)							
mean ± sd	2.1 ± 2.4	1.1 ± 1.6	1.2 ± 1.6	2.1 ± 3.5	1.3 ± 1.5	1.5 ± 1.9	1.5 ± 2.3
median (Q1, Q3)	1.4 (0, 3.1)	0 (0, 2.2)	0 (0, 2.2)	0.63 (0, 3)	0.86 (0, 2.5)	0.7 (0, 2.8)	0.48 (0, 2.5)
min - max	0 -- 8.6	0 -- 7.1	0 -- 6.1	0 -- 16	0 -- 7.3	0 -- 6.9	0 -- 16
Metabolic Cum. Incidence (%) (occup.)							
mean ± sd	2.2 ± 2.1	1.2 ± 1.7	2.2 ± 2.3	3.1 ± 2.7	2.1 ± 2.6	1.8 ± 1.8	2.1 ± 2.4
median (Q1, Q3)	1.8 (0, 3.3)	0 (0, 2.3)	1.8 (0, 3.2)	2.4 (1, 4.9)	1.8 (0.65, 2.6)	1.5 (0.22, 2.5)	1.7 (0, 3.1)
min - max	0 -- 7	0 -- 6.5	0 -- 8.7	0 -- 12	0 -- 22	0 -- 7.8	0 -- 22
Diabetes Cum. Incidence (%) (occup.)							
mean ± sd	0.86 ± 1.1	0.45 ± 0.86	0.79 ± 1.1	1.4 ± 1.9	1 ± 1.9	0.75 ± 1	0.91 ± 1.5
median (Q1, Q3)	0 (0, 1.9)	0 (0, 0.88)	0.52 (0, 1.2)	0.63 (0, 2.3)	0.4 (0, 1.5)	0.35 (0, 1.2)	0 (0, 1.4)
min - max	0 -- 3.2	0 -- 4.1	0 -- 4.8	0 -- 9.3	0 -- 17	0 -- 4.3	0 -- 17
Musculoskelet. syst. Cum. Incidence (%) (occup.)							
mean ± sd	4.3 ± 3.1	3.8 ± 3	4.5 ± 3.5	6.2 ± 4.9	3.6 ± 2.8	3.3 ± 2.1	4.4 ± 3.7
median (Q1, Q3)	3.5 (1.7, 5.7)	3.1 (1.3, 6.4)	4 (2.1, 5.8)	4.6 (2.7, 9.3)	3.1 (2.2, 4.5)	3.1 (2.1, 4.2)	3.6 (2, 6.1)

min - max	0 -- 11	0 -- 11	0 -- 15	0 -- 27	0 -- 22	0 -- 10	0 -- 27
Neoplasm Cum. Incidence (%) (occup.)							
mean ± sd	0.42 ± 0.83	0.42 ± 0.81	0.74 ± 0.97	0.51 ± 0.86	0.34 ± 0.73	0.35 ± 0.75	0.46 ± 0.83
median (Q1, Q3)	0 (0, 0)	0 (0, 0.65)	0 (0, 1.4)	0 (0, 1)	0 (0, 0)	0 (0, 0)	0 (0, 0.84)
min - max	0 -- 2.5	0 -- 3.3	0 -- 3.5	0 -- 3.5	0 -- 3.1	0 -- 2.7	0 -- 3.5
Nervous syst. Cum. Incidence (%) (occup.)							
mean ± sd	1.9 ± 2.5	1.6 ± 1.7	2 ± 1.7	2.9 ± 2.8	1.7 ± 3.5	1.3 ± 1.5	2 ± 2.6
median (Q1, Q3)	1.1 (0, 3.1)	1.3 (0, 2.7)	1.8 (0.5, 2.5)	2.6 (0, 4.6)	1.3 (0, 2)	1.2 (0, 2.1)	1.5 (0, 2.8)
min - max	0 -- 8.9	0 -- 6.9	0 -- 7.3	0 -- 12	0 -- 33	0 -- 6.1	0 -- 33
Ear.mastoid Cum. Incidence (%) (occup.)							
mean ± sd	0.48 ± 0.76	0.45 ± 0.65	0.73 ± 0.99	0.74 ± 1.2	0.52 ± 0.72	0.69 ± 0.79	0.6 ± 0.88
median (Q1, Q3)	0 (0, 1)	0 (0, 1)	0 (0, 1.4)	0 (0, 1.2)	0 (0, 1)	0.58 (0, 1.3)	0 (0, 1.1)
min - max	0 -- 2.5	0 -- 2.1	0 -- 3.4	0 -- 5.3	0 -- 3.4	0 -- 2.8	0 -- 5.3
Eye.adnexa Cum. Incidence (%) (occup.)							
mean ± sd	0.93 ± 1.3	0.98 ± 1.2	1.1 ± 1.2	1 ± 1.4	0.86 ± 1	0.9 ± 1	0.97 ± 1.2
median (Q1, Q3)	0 (0, 1.6)	0 (0, 1.9)	1 (0, 2)	0 (0, 2.1)	0.67 (0, 1.5)	0.55 (0, 1.7)	0.19 (0, 1.7)
min - max	0 -- 3.8	0 -- 4.9	0 -- 4.2	0 -- 5.3	0 -- 4.5	0 -- 3.4	0 -- 5.3
Pregn.condition Cum. Incidence (%) (occup.)							
mean ± sd	1.7 ± 3.2	1.9 ± 2.7	2.7 ± 2.4	4.6 ± 4.6	2.5 ± 2.5	1.7 ± 1.9	2.8 ± 3.3
median (Q1, Q3)	0 (0, 2.7)	0 (0, 3.5)	2.5 (0.71, 3.9)	3.1 (2, 6.3)	1.8 (0, 3.7)	1.1 (0, 3.2)	2.1 (0, 4.1)
min - max	0 -- 12	0 -- 10	0 -- 9.3	0 -- 21	0 -- 13	0 -- 6	0 -- 21
Psych.condition Cum. Incidence (%) (occup.)							
mean ± sd	5.1 ± 4	3.8 ± 3.3	5.8 ± 4.6	9.3 ± 6.9	4.9 ± 5.6	4.3 ± 3.7	5.8 ± 5.5
median (Q1, Q3)	4.3 (2.9, 5.8)	3 (1.3, 5.6)	4.9 (3.3, 7.5)	7.9 (4.2, 12)	3.9 (2.6, 5.8)	3.7 (1.8, 6)	4.5 (2.3, 7.3)
min - max	1 -- 18	0 -- 12	0 -- 20	0 -- 35	0 -- 50	0 -- 19	0 -- 50
Psych.prescrip. Cum. Incidence (%) (occup.)							
mean ± sd	1.3 ± 2.8	1 ± 1.7	2.1 ± 2.4	0.98 ± 1.3	0.55 ± 1	0.83 ± 2	1 ± 1.7
median (Q1, Q3)	0 (0, 0.77)	0 (0, 1.4)	1.5 (0.2, 2.8)	0.52 (0, 1.6)	0 (0, 0.67)	0 (0, 0.75)	0 (0, 1.6)
min - max	0 -- 11	0 -- 5.8	0 -- 9.8	0 -- 6	0 -- 4.4	0 -- 9.5	0 -- 11
Respiratory syst. Cum. Incidence (%) (occup.)							
mean ± sd	9.5 ± 7.4	7.7 ± 6.4	6.9 ± 7.4	7.4 ± 6	7.4 ± 11	7.3 ± 3.9	7.5 ± 7.6
median (Q1, Q3)	7.3 (5.2, 8.7)	6.6 (3.1, 10)	4.9 (1.5, 10)	5.8 (3.1, 10)	5.6 (3.4, 8.6)	6.6 (4.3, 10)	6 (3.2, 10)
min - max	2.7 -- 32	0 -- 31	0 -- 38	0 -- 32	0 -- 100	0 -- 16	0 -- 100
Genitourinary syst. Cum. Incidence (%) (occup.)							
mean ± sd	1.7 ± 2.6	1.5 ± 1.7	2.2 ± 1.7	3.5 ± 3.1	2 ± 1.9	2.2 ± 2.1	2.3 ± 2.3
median (Q1, Q3)	0 (0, 2.2)	1 (0, 2.8)	2.2 (0.9, 3)	3.2 (1.8, 4.3)	1.6 (0, 3)	1.8 (1, 2.5)	2 (0, 3.4)
min - max	0 -- 9.5	0 -- 6.7	0 -- 7.6	0 -- 22	0 -- 8.1	0 -- 8.6	0 -- 22
Peri-pandemic Inc.							
mean ± sd	0 ± 0	0 ± 0	0.8 ± 0.4	1 ± 0	1 ± 0	1 ± 0	0.69 ± 0.46
median (Q1, Q3)	0 (0, 0)	0 (0, 0)	1 (1, 1)	1 (1, 1)	1 (1, 1)	1 (1, 1)	1 (0, 1)
min - max	0 -- 0	0 -- 0	0 -- 1	1 -- 1	1 -- 1	1 -- 1	0 -- 1
Peri-pandemic time trend							
mean ± sd	0 ± 0	0 ± 0	3.8 ± 3.1	17 ± 3.4	28 ± 3.6	37 ± 1.1	15 ± 13
median (Q1, Q3)	0 (0, 0)	0 (0, 0)	3 (1, 7)	18 (14, 20)	28 (25, 31)	37 (36, 38)	15 (0, 26)
min - max	0 -- 0	0 -- 0	0 -- 10	11 -- 22	23 -- 34	35 -- 38	0 -- 38
