# Supplementary material for: Impact of the COVID-19 pandemic on incident diagnoses in German refugee centres 2018 to 2023
Source: Nat Commun. 2025 Jul 24;16:6808. doi: 10.1038/s41467-025-61876-x (PMC12290057; doi:10.1038/s41467-025-61876-x)
Supplement: Supplementary file 2 — Reporting Summary [file 41467_2025_61876_MOESM2_ESM.pdf]

nature portfolio

Corresponding author(s): Kayvan Bozorgmehr

Last updated by author(s): Jun 9, 2025

Reporting Summary

Nature Portfolio wishes to improve the reproducibility of the work that we publish. This form provides structure for consistency and transparency in reporting. For further information on Nature Portfolio policies, see our Editorial Policies and the Editorial Policy Checklist.

Statistics

For all statistical analyses, confirm that the following items are present in the figure legend, table legend, main text, or Methods section.

n/a	Confirmed	
☐	☒	The exact sample size (*n*) for each experimental group/condition, given as a discrete number and unit of measurement
☐	☒	A statement on whether measurements were taken from distinct samples or whether the same sample was measured repeatedly
☐	☒	The statistical test(s) used AND whether they are one- or two-sided<br>*Only common tests should be described solely by name; describe more complex techniques in the Methods section.*
☐	☒	A description of all covariates tested
☐	☒	A description of any assumptions or corrections, such as tests of normality and adjustment for multiple comparisons
☐	☒	A full description of the statistical parameters including central tendency (e.g. means) or other basic estimates (e.g. regression coefficient) AND variation (e.g. standard deviation) or associated estimates of uncertainty (e.g. confidence intervals)
☐	☒	For null hypothesis testing, the test statistic (e.g. *F*, *t*, *r*) with confidence intervals, effect sizes, degrees of freedom and *P* value noted<br>*Give P values as exact values whenever suitable.*
☒	☐	For Bayesian analysis, information on the choice of priors and Markov chain Monte Carlo settings
☐	☒	For hierarchical and complex designs, identification of the appropriate level for tests and full reporting of outcomes
☒	☐	Estimates of effect sizes (e.g. Cohen's *d*, Pearson's *r*), indicating how they were calculated

Our web collection on statistics for biologists contains articles on many of the points above.

Software and code

Policy information about availability of computer code

Data collection	Refugee Care Manager (Ref.Care) Version 1.1.8 (www.refcare.org)
Data analysis	The analyses were conducted in R version 4.2.1 using packages glmmTMB for fitting mixed-effects models. The original R output can be found in Appendix. The full code can be requested by contacting the corresponding author (Kayvan Bozorgmehr, refcare.allmed@med.uni-heidelberg.de)

For manuscripts utilizing custom algorithms or software that are central to the research but not yet described in published literature, software must be made available to editors and reviewers. We strongly encourage code deposition in a community repository (e.g. GitHub). See the Nature Portfolio guidelines for submitting code & software for further information.

Data

Policy information about availability of data

All manuscripts must include a data availability statement. This statement should provide the following information, where applicable:

- Accession codes, unique identifiers, or web links for publicly available datasets
- A description of any restrictions on data availability
- For clinical datasets or third party data, please ensure that the statement adheres to our policy

The datasets generated and/or analysed during the current study are not publicly available due to the data-use and -access (DUAC) regulations of the Pri.CareNet Consortium. The generated and analysed datasets are available for scientific purposes from the Pri.CareNet Consortium upon request by contacting the

spokesperson (Kayvan Bozorgmehr, refcare.allmed@med.uni-heidelberg.de). Data provision is subject to written request with a specification of detailed research questions, a draft analysis plan, the cooperation with at least one consortium member, as well as clearance by the Consortium's DUAC based on criteria of feasibility and ethical considerations. Source data are provided with this paper.

Research involving human participants, their data, or biological material

Policy information about studies with human participants or human data. See also policy information about sex, gender (identity/presentation), and sexual orientation and race, ethnicity and racism.

Reporting on sex and gender	Sex is based on data coded in routine medical records (based on pysicians' coding), gender was not captured or available. Sex was considered in the analyses as co-variable in regression models. Differential effects of the pandemic between men and women were not analysed. Sex-stratified descriptive data of all outcomes are available.
Reporting on race, ethnicity, or other socially relevant groupings	No variables of race or ethnicity were used. Country of origin, i.e. nationality recorded in the medical records system, was used in the regression models to adjust for differences in incident diagnoses that may be attributable to compositional changes of the underlying refugee poopulation in a centre, and less to the analysed exposure (COVID-19 pandemic). Nationalities included in the analyses were restricted to those with the most frequent share among the refugee population across all centres.<br>Other socially relevant grouping: we use the social category "refugee" in our analysis, acknowledging that this category subsumes a heterogenous population. We further use this term as "umbrella term" for persons who are registered in the centres and live their with different residence status as asylum claimants, asylum seekers, resettlement refugees, accepted refugees, or tolerated individuals. As our data is based on medical records data, no information was available for the different residence status to allow for more nuanced dis-agrgegation.
Population characteristics	See above
Recruitment	Individuals are included in the medical records if they seek care in one of the on-site medical clinicals established for refugees, so that data are "utilisation data" and data of individuals who did not seek care is not included. Individuals may also seek care outside of reception centres, but this usually requires approval and in case of serious conditions data of referrals are recorded in RefCare in the refugee centers as well to ensure ongoing treatment.
Ethics oversight	The study uses de-individualised anonymous (aggregate) data, generated from a federated data analysis methodology performed on individual-level clinical data with the result of anonymous counts. The methodology and implementation of the approach for federated data analysis has been reviewed and approved by the Review Board of the Technology and Methods Platform for Networked Medical Research (TMF e.V.).

Note that full information on the approval of the study protocol must also be provided in the manuscript.

Field-specific reporting

Please select the one below that is the best fit for your research. If you are not sure, read the appropriate sections before making your selection.

☐ Life sciences ☒ Behavioural & social sciences ☐ Ecological, evolutionary & environmental sciences

For a reference copy of the document with all sections, see nature.com/documents/nr-reporting-summary-flat.pdf

Behavioural & social sciences study design

All studies must disclose on these points even when the disclosure is negative.

Study description	Study type: multi-centre observational prospective (open) cohort study analysed in a quasi-experimantal (interrupted time series) design
Research sample	Refugees in German refugee reception centres in three German federal states. Descriptive soci-demographics: see Table 1 in manuscript. Sample is representative to the general population of refugees in Germany with respect to age, sex, and distribution of nationalities. Furthermore, allocation to states occurs on a quasi-random manner based on administrative quota, which is why it is very likely that there is no systematic difference refugee characteristics or underlying morbidity between different states. But sites and states may differ with respect to contexts and pandemic measures, which is why we considered this level as random effect in the analysis. The study is based on pre-exisiting datasets obtained and generated through the PriCareNet Surveillance Network, which covers three federal states in Germany. This is why no additional sites could be considered, especially for the historical data.
Sampling strategy	No sample size calculation was performed as the data is based on an existing dataset. The analysis was conducted within the framework of PriCarenet, a health surveillance network21. PriCarenet is overseen by the University Hospital Heidelberg and comprises healthcare providers operating healthcare facilities on-site as of October 2018. Since December 2023, these facilities are distributed across 24 state-level registration and reception centres, along with one district-level accommodation centre for refugees in Germany. These 25 centres are situated in the German states of Baden-Wuerttemberg, Bavaria, and Hamburg. These states collectively host approximately 30% of the asylum-seeking population in Germany, as determined by administrative quotas 43. Within PriCarenet, healthcare providers are equipped with a customized Electronic Health Record (EHR) system known as Refugee Care Manager (RefCare). RefCare not only includes standard medical record-keeping features but also incorporates a built-in health surveillance module (Table 2). The surveillance module comprises an automated analysis of locally stored medical routine data using predefined indicators. The indicators are constructed using diagnosis categories based on International Classification of Diseases

(ICD-10-GM Version 2021) and drug prescriptions based on the Anatomic Therapeutic Classification (ATC 2023) as defined and outlined in Table 3, and operationalized through a standardised analysis script . To protect data anonymity, any observations with counts less than 3 are adjusted to 0. More detailed information about the surveillance infrastructure in PriCarenet, and the local analysis of indicators can be found in previous reports .
The data used in this paper covers the time span from October 2018 to April 2023. The facilities included in this study joined the surveillance network at different dates (Supplementary chapter 3.). Some centres have since departed from the network due to closures or changes in healthcare providers, but still contributed their anonymous health surveillance data for the purpose of this study. Provided data consequently varies per centre (Supplementary chapter 3.).
RefCare is used by health professionals, who are the data holders of the individual-level patient data in on-site health care facilities. The respective authorities in the three federal states are responsible for immigration data, and are data holders of the occupancy data, i.e. the sociodemographic information of the refugee centres' inhabitants.

Data collection	see above (under Sampling strategy)
Timing	The data used in this paper covers the time span from October 2018 to April 2023. The facilities included in this study joined the surveillance network at different dates (Supplementary chapter 3.). Some centres have since departed from the network due to closures or changes in healthcare providers, but still contributed their anonymous health surveillance data for the purpose of this study. Provided data consequently varies per centre (Supplementary chapter 3.).
Data exclusions	See methods page 12/13 and Figure 8: We matched the EHR data with the monthly occupancy data for each centre, wherever possible (Figure 8). In 64 cases, the occupancy count was lower than the number of patients (i.e., n_occ<n_pat). This occurrence is reasonable in situations where refugee centres experience a rapid turnover of individuals, such as a high influx of new arrivals and frequent transfers. In such instances, individuals may seek on-site healthcare services but stay within the centres for only a brief period, leading to a temporary misalignment between occupancy figures and the number of patients receiving healthcare services. These observations were excluded for the main analysis which resulted in a total of 314 centre-months between October 2018 and April 2023 of 21 centres (with 〖mean(n)〗_pat)=243, sd(n_pat)=240, 〖mean(n)〗_occ)=459 and sd(n_occ)=462; subset 2).<br>In 75 cases, the sum of the reported strata counts (female/male x adult/children) did not equal the reported total occupancy. Therefore, we repeated the main analysis on subset 3 (sensitivity analysis 2), where the occupancy totals equal the totals in occupancy age-and sex-strata AND n_occ≥n_pat. (Supplementary chapter 2.2.). Furthermore, in sensitivity analysis 3, we repeated the main analysis again (which was performed on subset 2), but instead used subset 4 of the linked data which contained no restrictions, i.e. all observations of the linked dataset (Supplementary chapter 2.3.).
Non-participation	n/a
Randomization	Allocation of refugees to refugee centres: occurs in a quasi-random fashion based on administrative quota (outside of the control of researchers and refugees alike, no self-selection into centres possible with few exceptions). Allocation of refugees/centres to exposure: based on a temporal cut-off related to the onset of the COVID-19 pandemic, see methods page 13: covid: coded 0 for pre-covid time points and 1 for post-covid time points (0: < March 2020, 1: ≥ March 2020). This variable captures the impact of the COVID-19 pandemic in peri-pandemic time periods, with pre-pandemic time periods used as reference. Systematic differences in pre- and post-covid time periods between refugees (with respect to their socio-demographic and morbidity) is unlikely because of the qusio-random allocation procedure to refugee centres. We minimised the risk for residual confounding by adjusting for sex, age, and nationality (amongst others) in our regression models.

Reporting for specific materials, systems and methods

We require information from authors about some types of materials, experimental systems and methods used in many studies. Here, indicate whether each material, system or method listed is relevant to your study. If you are not sure if a list item applies to your research, read the appropriate section before selecting a response.

Materials & experimental systems

n/a	Involved in the study	
☒	☐	Antibodies
☒	☐	Eukaryotic cell lines
☒	☐	Palaeontology and archaeology
☒	☐	Animals and other organisms
☒	☐	Clinical data
☒	☐	Dual use research of concern
☒	☐	Plants

Methods

n/a	Involved in the study	
☒	☐	ChIP-seq
☒	☐	Flow cytometry
☒	☐	MRI-based neuroimaging

Plants

Seed stocks	*Report on the source of all seed stocks or other plant material used. If applicable, state the seed stock centre and catalogue number. If plant specimens were collected from the field, describe the collection location, date and sampling procedures.*
Novel plant genotypes	*Describe the methods by which all novel plant genotypes were produced. This includes those generated by transgenic approaches, gene editing, chemical/radiation-based mutagenesis and hybridization. For transgenic lines, describe the transformation method, the number of independent lines analyzed and the generation upon which experiments were performed. For gene-edited lines, describe the editor used, the endogenous sequence targeted for editing, the targeting guide RNA sequence (if applicable) and how the editor was applied.*
Authentication	*Describe any authentication procedures for each seed stock used or novel genotype generated. Describe any experiments used to assess the effect of a mutation and, where applicable, how potential secondary effects (e.g. second site T-DNA insertions, mosiacism, off-target gene editing) were examined.*
